# Development of a Second-Generation RARα Selective Antagonist as an Orally Bioavailable, Effective, Safe, and Reversible Male Contraceptive

**DOI:** 10.1021/acs.jmedchem.6c00011

**Published:** 2026-05-11

**Authors:** Rui Shi, Kristen John, Xuan Qin, Ehfazul Haque, Taimeng Liang, Narsihmulu Cheryala, Feng Li, Henry L. Wong, Gunda I. Georg

**Affiliations:** † Department of Medicinal Chemistry and Institute for Therapeutics Discovery and Development, College of Pharmacy, 5635 University of Minnesota, 717 Delaware Street SE, Minneapolis, Minnesota 55414, United States; ‡ Center for Drug Discovery, Department of Pathology and Immunology, NMR and Drug Metabolism Core, Advanced Technology Cores, Department of Biochemistry and Molecular Pharmacology, 3989 Baylor College of Medicine, One Baylor Plaza, MS: BCM330, Houston, Texas 77030, United States; § Department of Chemistry, University of Minnesota, 207 Pleasant Street, SE, Minneapolis, Minnesota 55455-0431, United States

## Abstract

We report the design and SAR studies of benzopyran-, benzofuran-, and benzothiophene-derived inhibitors of the retinoic acid receptor alpha (RARα) for male contraception. SAR studies identified critical features influencing activity, such as the optimal positioning of antagonism moieties and substituents, leading to the discovery of (*S*)-4-(5-(2,8-dimethyl-5-(*p*-tolyl)-2*H*-chromen-3-yl)-1*H*-pyrrol-2-yl)­benzoic acid (compound **23**). Compound **23** is a highly potent RARα inhibitor (IC_50_ = 0.051 nM) with excellent selectivity (>1650-fold over RARβ and >1960-fold over RARγ) and ADMET properties. Compound **23** is orally bioavailable and reduces sperm counts in mice for a full male contraceptive effect with a minimum effective dose between 0.3 and 1 mg/kg/day, with complete recovery after drug continuation. Using imaging mass spectrometry, we analyzed the spatial distribution of compound **23** in mouse seminiferous tubules. Compound **23** does not cross the blood–testis barrier and therefore must exert its activity during the initial phases of spermatogenesis.

## Graphical abstract

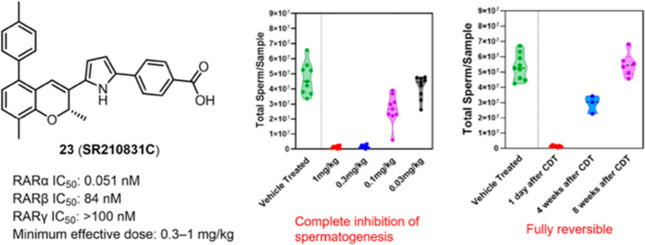

## Introduction

Modern contraceptive methods have significantly advanced family planning and reduced maternal health risks, yet the burden of contraception continues to fall disproportionately on women. While female contraceptives are numerous and varied, male options remain limited to condoms and vasectomy. Condoms, although accessible and reversible, have a typical-use failure rate of 13%, while vasectomy is a surgical and often irreversible procedure.^1,2^ As a result, unintended pregnancy rates remain high, affecting 93% of pregnancies in low-income countries, 67% in middle-income countries, and 34% in high-income countries, with nearly half of them ending in abortion.^3^ This stark imbalance underscores the urgent need for effective reversible male contraceptive options.^4^

Testosterone and its esters have been investigated as male contraceptive agents; however, testosterone alone is insufficient to suppress spermatogenesis fully and exhibits variable efficacy among different ethnic groups.^5,6^ Additional reported side effects include weight gain, acne, mood disturbances, and pain at injection sites.^7^ Moreover, approximately 2.2% of men fail to respond to testosterone-based regimens, indicating the presence of “nonresponders.”^7^ Recent efforts have focused on developing testosterone analogs such as 7-α-11β-dimethyl-19-nortestosterone undecanoate (**DMAU**) and 11-β-methyl-19-nortestosterone-17β-dodecylcarbonate (**11βMNTDC**), which are currently in clinical trials for oral, subcutaneous, or intramuscular administration.^8^ These compounds function as prodrugs that are metabolized in vivo to active forms capable of binding both androgen and progesterone receptors, thereby enhancing gonadotropin suppression to achieve more consistent azoospermia while minimizing adverse effects. Although combining testosterone with progestins improves efficacy, prolonged use of testosterone still poses risks, including cardiotoxicity,^9^ liver damage,^10^ and erythrocytosis.^11^ A transdermal gel delivering segesterone acetate (nestorone) and testosterone has shown encouraging results in recent clinical trials, with effective suppression of sperm production and full reversibility. Side effects were reported in only a minority of participants.^12^

Due to the limitations associated with hormonal male contraceptives and the need to expand contraceptive choices, significant efforts have been focused on developing nonhormonal alternatives that reduce the risk of side effects, health complications, and systemic toxicity. Several nonhormonal targets are currently under investigation, with some approaches showing promise in preclinical models.^13^ One such target is the epididymal protease inhibitor (EPPIN), a male-specific protein present on the surface of human sperm. Administration of the small molecule **EP055**, which inhibits EPPIN, resulted in complete and reversible suppression of sperm motility in cynomolgus rhesus monkeys.^14^ Another promising target is soluble adenylyl cyclase (sAC), an enzyme critical for sperm motility. In preclinical studies, sAC inhibitors produced reversible, on-demand contraceptive effects following intraperitoneal injection.^15^ The natural product triptonide has also demonstrated strong contraceptive efficacy in multiple animal models, including nonhuman primates;^13^ however, its known toxicity necessitates further safety evaluation before clinical advancement.

Retinoic acid (RA), the endogenous metabolite of vitamin A and β-carotene, plays a vital role in spermatogenesis by maintaining the integrity of the blood-testis barrier, promoting spermatogonial differentiation, and enabling spermiation.^16^ RA exerts its biological effects through two classes of nuclear receptors: retinoic acid receptors (RARs) and retinoid X receptors (RXRs). RARs are primarily activated by all-trans-retinoic acid (**ATRA**) (**1**) (Figure 1), while RXRs are selectively activated by 9-cis-retinoic acid. Of the three RAR isoforms (α, β, and γ), both RARα and RARγ are essential for male fertility. Knockout studies show that mice lacking either RARα or RARγ are sterile. In contrast, RARβ-deficient males remain fertile, underscoring the importance of RARα and RARγ as attractive targets for nonhormonal male contraception.^17–19^ Although retinoic acid signaling regulates diverse biological processes, historical safety concerns largely stem from its essential role during development and from the effects of broad perturbation of the retinoid pathway. Adult RARα-deficient mice that survive postnatally are otherwise viable, with male sterility representing the most consistent adult phenotype. In addition, in a transgenic reporter mouse model of RAR signaling, activity was more significantly reduced in the testes of vitamin A-deficient mice than in any other organ.^20^ These observations identify RARα as a mechanistically validated contraceptive target in adults.^21^ Pharmacologic blockade of RA signaling using pan-RAR antagonist, **BMS-189453** (**2**) (Figure 1), has resulted in reversible infertility in rodent models.^22^ However, the lack of isoform selectivity and agonist activity at RARβ may contribute to off-target effects, limiting its clinical viability. In this context, RARα selectivity represents a rational risk-mitigation strategy to limit engagement of retinoid signaling outside the testis while retaining contraceptive efficacy. Detailed analyses of mouse RARα-deficient testes reveal multiple defects in spermatogenesis, including a temporary arrest of step 8–9 spermatids during the initial wave, delayed progression of pachytene/leptotene (PL/L) spermatocytes in the second wave, and their accumulation in the third wave.^23^ Additional abnormalities include disrupted cellular associations due to asynchronous germ cell development, reduced germ cell proliferation, increased apoptosis in elongating spermatids, misorientation of step 8–9 spermatids, entrapment within Sertoli cells, and impaired spermiation at stage VIII.^17,21,23–25^ These defects are predominantly observed during stages VIII–IX of the seminiferous epithelial cycle, aligning with the peak expression of RARα.^26^ A diagrammatic representation of the spermatogenic cycle illustrating these abnormalities in RARα-deficient mice was provided by Wolgemuth and Chung.^26^ Importantly, pairwise BLAST alignment of human and mouse RARα revealed 98.48% sequence identity in the DNA-binding domain and 98.23% identity in the ligand-binding domain, indicating strong cross-species conservation of receptor function and supporting the translational relevance of rodent pharmacology. Despite this genetic and mechanistic validation, early RARα-selective antagonists such as **BMS-189532** (**3**) (Figure 1) and **BMS-189614** (**4**) (Figure 1) demonstrated limited in vivo efficacy, primarily due to poor pharmacokinetic properties and inadequate testicular exposure.^27^ These compounds, characterized by a dihydronaphthalene scaffold and an amide linker, failed to elicit sufficient contraceptive effects in vivo.

**Figure 1:**
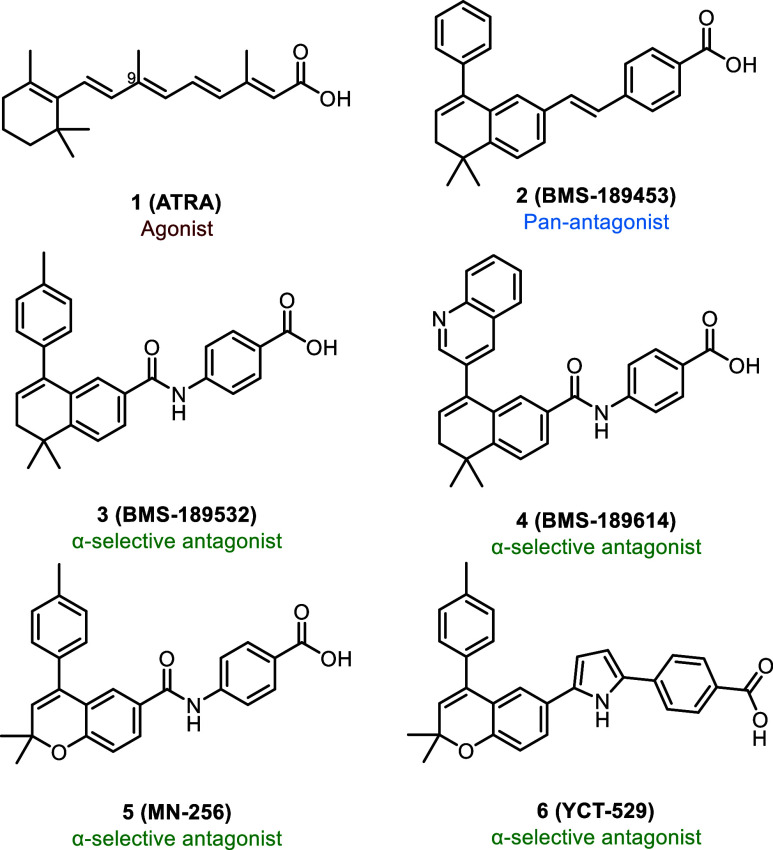
Structures of retinoic acid and RAR antagonists.

To address these limitations, our group previously reported **MN-256** (**5**) (Figure 1), a chromene-based RARα-selective antagonist.^28^ **MN-256** exhibits potent antagonistic activity and induces distinct yet modest effects on spermatogenesis, while it failed to prevent fertility in mating studies. Further structure–activity relationship (SAR) optimization led to the development of **YCT-529** (**6**) (Figure 1), a pyrrole-linked RARα-selective antagonist with high potency (IC_50_ = 6.8 nM), >550-fold selectivity over RARβ and RARγ, excellent oral bioavailability, and metabolic stability.^29^ In CD-1 mice, daily oral administration at 10 mg/kg for 4 weeks rendered 99% of males infertile, with full fertility restored 6 weeks after treatment cessation.^29^ In cynomolgus macaques, dosing at 0.5–7.5 mg/kg/day induced rapid, reversible, severe oligospermia or azoospermia without systemic or hormonal toxicity (over a total dosing duration of 108 days and recovery phase of 147 days), confirming a favorable safety margin.^29^ These data supported clinical translation: YourChoice Therapeutics completed a single-ascending-dose Phase 1a first-in-human study in June 2024,^30^ demonstrating favorable safety, tolerability, and pharmacokinetics; a multicenter Phase 1*b*/2a repeat-dose escalation trial launched in September 2024 and is currently enrolling up to 66 participants to evaluate 28- and 90 day oral regimens and semen-parameter pharmacodynamics.^31^ Collectively, these milestones position **YCT-529** as the leading nonhormonal male contraceptive candidate in clinical development.

Given the success of **YCT-529** and its analogs as selective RARα antagonists for male contraception, subsequent efforts in this study were directed toward the development of alternative scaffolds for RARα-selective antagonists. SAR analysis of **YCT-529** identified the pyrrole linker as a critical structural element, conferring both high potency and strong isoform selectivity. However, extensive SAR exploration around the **YCT-529** scaffold revealed limited tolerance to structural modification,^32^ thereby limiting opportunities to further optimize physicochemical and pharmacokinetic properties. However, these findings solidified the pyrrole motif as an optimal linker, motivating the search for new scaffolds that retain this key interaction while offering additional flexibility for medicinal chemistry optimization.

To expand chemical space, we explored two previously reported pyrrole-linked retinoids that have been characterized as RARα-selective agonists (Figure 2).^33,34^ These compounds showed significant promise as templates for developing antagonists. Building on the established SAR framework, bulky aromatic substitutions on the bicyclic core were identified as a strategy to shift retinoid behavior from agonism to antagonism.^35^ This modification aligns with findings that steric clashes induced by such substitutions disrupt receptor activation, transforming agonists into antagonists.

**Figure 2:**
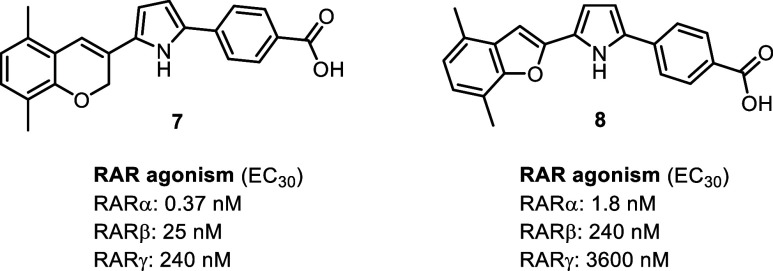
Structures and activity of known pyrrole-based RARα-selective agonists.

This planned approach aims to leverage both the structural advantages of pyrrole linkers and the functional transformation achievable through targeted core modifications. By combining these strategies, we seek to generate novel RARα-selective antagonists with enhanced pharmacological profiles, further advancing the field of nonhormonal male contraception.

## Results

The agonist and antagonist activities of the synthesized compounds were evaluated in GeneBLAzer reporter cell assays against human RARα, RARβ, and RARγ. In this system, cells express recombinant RAR ligand-binding domains fused to a Gal4 DNA-binding domain, which drives expression of the β-lactamase reporter gene upon receptor activation. β-Lactamase activity was measured using the cell-permeable FRET substrate CCF4-AM. Increasing agonist concentrations increases β-lactamase expression, reducing the FRET signal and enabling determination of agonist EC_50_ values. For antagonist evaluation, cells were stimulated with all-trans-retinoic acid (ATRA) at its EC_80_ concentration in the presence of increasing concentrations of test compounds. Under these assay conditions, antagonist activity could be assessed up to 100 nM, which was sufficient to evaluate RARα selectivity relative to RARβ and RARγ.

### Rational Design of RARα-Selective Antagonists Based on Compound **7**

Compound **7** is an RARα-selective agonist featuring a pyrrole linker and a terminal benzoic acid, similar to **YCT-529**, but with a distinct benzopyran bicyclic core that effectively occupies the hydrophobic pocket of the RARα ligand-binding domain. Compound **7** demonstrates remarkable potency, with a subnanomolar EC_30_ for RARα, along with 67-fold selectivity over RARβ and 648-fold selectivity over RARγ.^30^ These results highlight the potential of the benzopyran core and pyrrole linker combination in achieving high potency and selectivity.

To convert compound **7** from an agonist into an antagonist, we introduced a bulky aromatic substituent on the benzopyran ring (Table 1). Structural studies have shown that agonist binding stabilizes helix 12 (H12) of the RAR ligand-binding domain (LBD) in its active conformation, facilitating coactivator recruitment and downstream transcriptional activation.^36,37^ In contrast, antagonists feature bulky substituents on the hydrophobic core (Figure 1), which drive H12 into an inactive conformation, preventing coactivator binding and thereby repressing gene expression.^36^ Our design leverages this mechanistic insight by introducing targeted steric hindrance within the LBD, a strategy known to promote antagonism. This single modification aims to maintain the high potency and RARα selectivity of compound **7**’s scaffold while functionally shifting its activity toward antagonism.

**Table 1:**
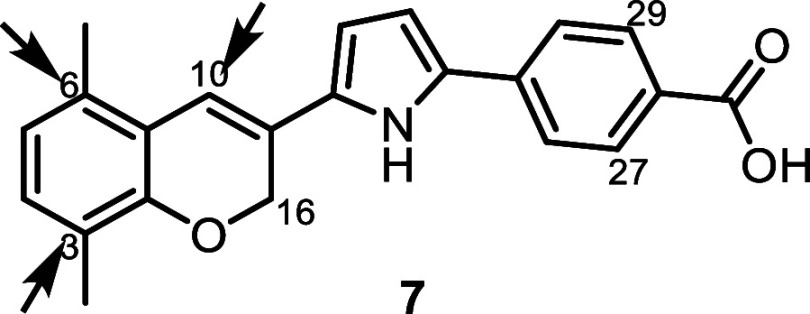

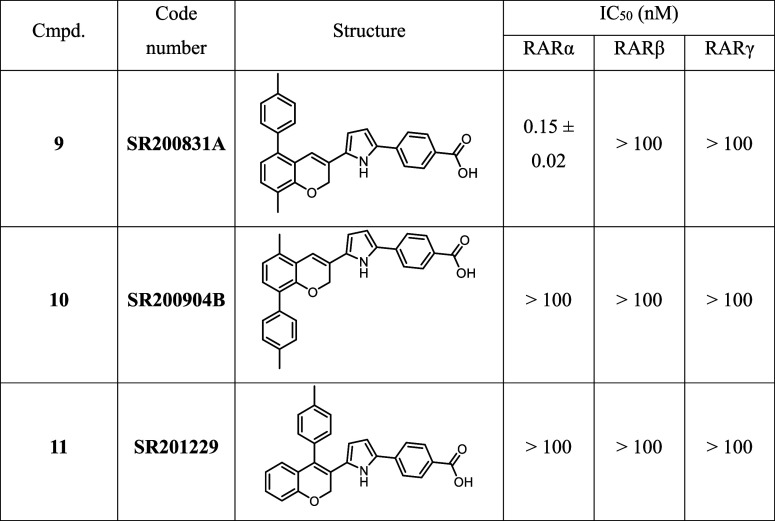
In Vitro Antagonism Activity of Analogs of **7** Carrying an Antagonism Moiety^a^

a *n* = 3 independent experiments.

### SAR Exploration of Analogs of **7**

There are three potential positions for incorporating the antagonism moiety. To determine the optimal position and assess its impact on antagonistic activity and selectivity, we designed, synthesized, and evaluated three analogs: **9**, **10**, and **11** (Table 1). Among the three, incorporation of the antagonism moiety at the C3 and C10 positions produced compounds without antagonistic activity on RARs (Table 1). In contrast, incorporation at the C6 position yielded a highly potent and selective RARα antagonist, with an IC_50_ of 0.15 nM and more than 667-fold selectivity over RARβ and RARγ. These results highlight the critical importance of the position of incorporation of the antagonism moiety for benzopyran-based RAR antagonists, with the **C6** position being exclusively preferred for achieving potent and selective activity.

We subsequently performed molecular modeling to predict the binding conformation of **9** with RARα using Schrödinger’s Maestro suite. As anticipated, the docking conformation revealed that the incorporated antagonism moiety extended into the pocket created by steric clashes with helix H12, thereby conferring antagonistic activity (Figure 3). The benzopyran scaffold effectively occupied the hydrophobic pocket, forming van der Waals interactions with surrounding hydrophobic residues, thereby providing high binding affinity. Additionally, the NH group on the pyrrole linker established a critical interaction with the unique S232 residue in RARα, ensuring selectivity over RARβ and RARγ. The distal benzoic acid group contributed to binding affinity and structural anchoring by forming key hydrogen bonds and a salt bridge with S287 and R276. These interactions collectively stabilized the compound in the correct binding conformation within the RARα ligand-binding domain, facilitating its potent and selective antagonistic activity.

**Figure 3:**
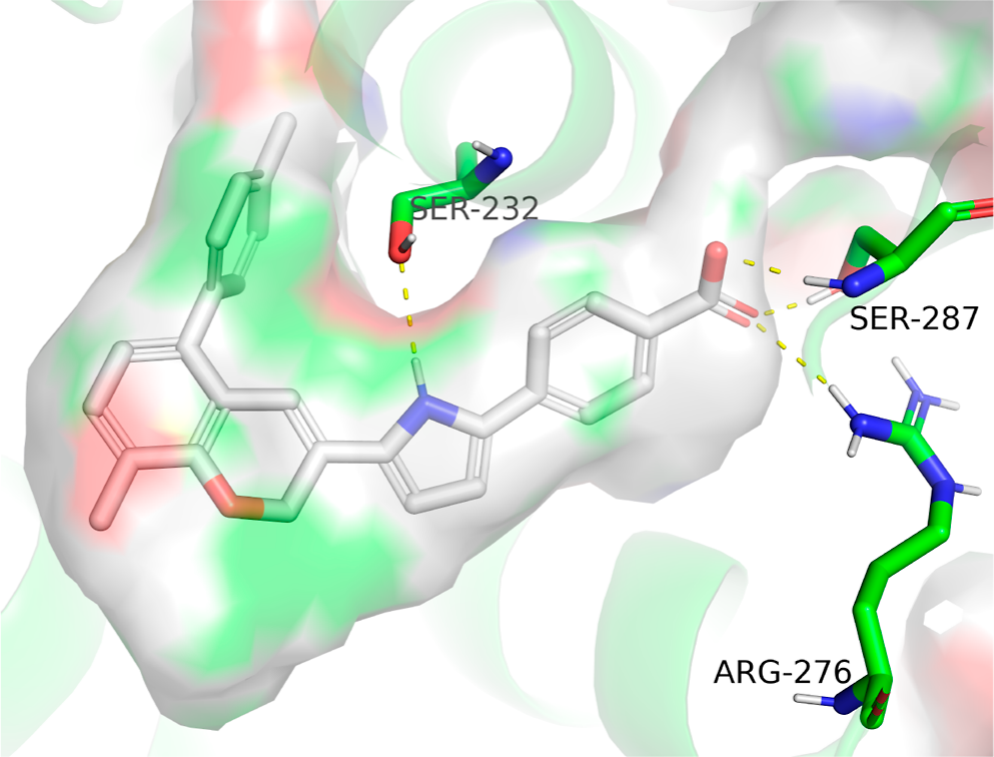
Predicted binding pose of **9** with RARα (PDB: 1dkf). The surface of the ligand-binding pocket is displayed. Polar interactions between **9** and RARα are shown with yellow dashed lines.

Analyzing the predicted binding conformation of **9** with RARα revealed several positions suitable for further compound optimization, including the antagonism moiety, the benzopyran core, and the benzoic acid group. Given the critical role of the pyrrole linker in driving both activity and selectivity, we chose to retain this structural feature. Subsequent derivatization and SAR studies were therefore focused on optimizing the other identified positions to enhance the compound’s potency, selectivity, and overall pharmacological profile.

The first position targeted for optimization was the antagonism moiety. Given the general hydrophobic nature of RAR binders, which typically feature a carboxylic acid terminal group, RAR antagonists often exhibit high hydrophobicity at positions distant from the carboxylic acid. This characteristic can lead to potential PK and physicochemical disadvantages, including low solubility, poor bioavailability, and high plasma protein binding. To mitigate these liabilities, we explored introducing heteroaromatic functionality into the antagonistic moiety. One functional group previously validated as an effective antagonism moiety is the quinoline group, as utilized in the known RARα-selective antagonist **BMS-189614**.^36^ Accordingly, we designed and synthesized **12**, featuring a quinoline antagonistic moiety (Table 2). Compound **12** demonstrated an IC_50_ of 1.6 nM against RARα, without measurable activity against RARβ and RARγ at concentrations up to 100 nM (Table 2). These results indicate that the quinoline group is well-tolerated within the benzopyran-based RARα-selective antagonist scaffold. However, **12** exhibited approximately 10-fold lower activity compared to its toluene counterpart, **9**, suggesting a trade-off between improved physicochemical properties and reduced potency.

**Table 2:**
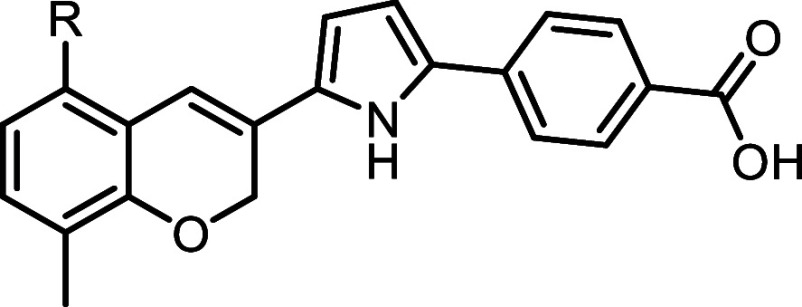

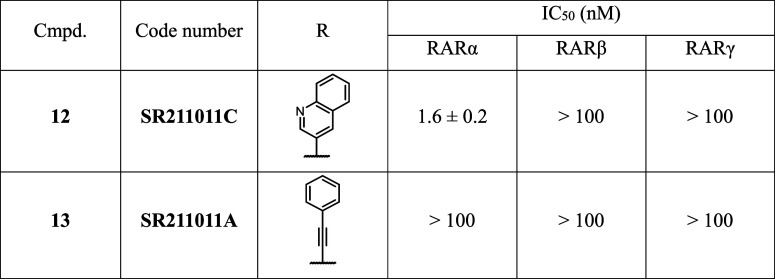
In Vitro Antagonism Activity of Analogs of **7** with Antagonism Moiety Derivatizations^a^

a *n* = 3 independent experiments.

Another strategy we pursued was to convert the benzopyran antagonist effect into inverse agonism. The compound **BMS493** is known to act as an RARα inverse agonist by stabilizing the β-strand S3 conformation through van der Waals interactions between its phenylacetylene group and RARα residues Ile396 and Leu398.^36^ This stabilization promotes the recruitment of transcriptional corepressors via the CoRNR1 (Co-Repressor for Nuclear Receptors 1) motif, a conserved peptide sequence in nuclear receptor corepressors such as NCoR and SMRT that mediates high-affinity binding to receptors in their inactive conformation. Engagement of the CoRNR1 motif suppresses basal transcriptional activity, thereby producing an inverse agonistic effect. Inspired by this mechanism, we introduced a phenylacetylene group to the benzopyran scaffold, designing and synthesizing **13** (Table 2). However, **13** exhibited no activity against the three RAR subtypes at concentrations up to 100 nM (Table 2). This lack of activity suggests that the phenylacetylene group is not compatible with the benzopyran scaffold, potentially due to differences in the binding conformation between the amide-based scaffold of **BMS493** and the pyrrole-based scaffold used in **13**.

We next explored derivatizations at the ortho position of the benzoic acid group. Molecular modeling of the binding conformation of **9** with RARα revealed a small pocket adjacent to this position, suggesting an opportunity to introduce modifications that could provide additional nonpolar interactions, thereby enhancing binding affinity (Figure 3). Additionally, we aimed to investigate the impact of altering the stereoelectronic properties of the benzoic acid group on antagonist activity and selectivity. Changes in electron density at this position could modulate the acidity of the carboxylic acid group, potentially affecting the compound’s physicochemical properties, including solubility, permeability, and metabolic stability. These derivatizations aimed to balance improvements in binding affinity, activity, and selectivity with enhanced drug-like properties.

We first investigated electron-withdrawing halogen substitutions, specifically chloro and fluoro groups, at the ortho position of the benzoic acid group (Table 3). Both monochloro and monofluoro substitutions were well-tolerated, yielding analogs with comparable activity against RARα while maintaining selectivity over RARβ and RARγ. Among these, **15**, featuring a monofluoro substitution, exhibited the highest activity, with an IC_50_ of 0.11 nM. In contrast, difluoro substitution at both ortho positions resulted in a 5-fold decrease in RARα activity compared to the monofluoro counterpart, indicating monohalogen substitution is preferred. The preference for monohalogen substitution at the ortho position is consistent with docking models that suggest the presence of a small, spatially restricted pocket adjacent to one side of the benzoic acid group, which can accommodate a single halogen substituent but not dual substitution without a steric penalty.

**Table 3:**
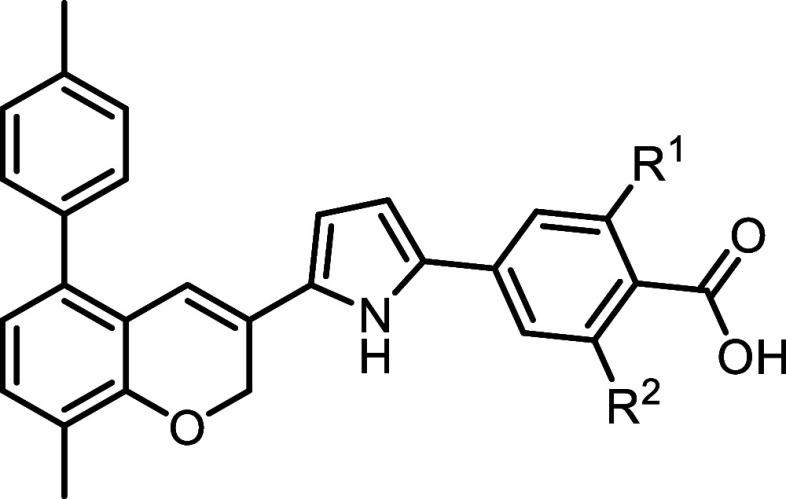
In Vitro Antagonism Activity of Analogs of **7** with Benzoic Acid Derivatizations^a^

				IC_50_ (nM)		
cmpd.	code number	*R* ^1^	*R* ^2^	RARα	RARβ	RARγ
**14**	**SR211205A**	Cl	H	0.16 ± 0.01	>100	>100
**15**	**SR210620A**	F	H	0.11 ± 0.02	>100	>100
**16**	**SR210620B**	F	F	0.55 ± 0.03	>100	>100
**17**	**SR211205B**	OH	H	0.50 ± 0.05	>100	>100
**18**	**SR210331C**	OMe	H	42 ± 8	>100	>100

a *n* = 3 independent experiments.

On the other hand, electron-donating substituents, such as a hydroxyl group, reduced activity against RARα (Table 3). For example, **17**, featuring a hydroxyl group at the ortho position, exhibited an IC_50_ of 0.50 nM. Larger electron-donating substituents, such as a methoxy group, further reduced activity, with an IC_50_ of 42 nM. These results indicate that smaller ortho substituents are preferred and that electron-withdrawing groups are more favorable, likely because they enhance polarization of the carboxylic acid and strengthen key polar interactions with conserved RARα residues such as Ser287 and Arg276, whereas electron-donating and bulkier substituents weaken these interactions and are less well tolerated.

Molecular modeling of the binding conformation of **9** with RARα identified an empty hydrophobic pocket near the pyran ring (Figure 3). To exploit this pocket and introduce additional van der Waals interactions to enhance binding affinity, we introduced hydrophobic substitutions at the R1 and R2 positions (Table 4). Initially, larger substituents were introduced at the R1 position, including CF_3_ (**19**), ethyl (**20**), and isopropyl (**21**). Among these, the smaller CF_3_ and ethyl substitutions showed activity against RARα comparable to **9**. In contrast, the larger isopropyl substitution resulted in approximately a 5-fold reduction in RARα activity. This result aligns with the molecular modeling data, which indicate that the empty hydrophobic pocket is primarily adjacent to the pyran oxygen and the R2 position, leaving limited space for substitutions at the R1 position. Consequently, larger substitutions at R1 are less favored due to steric constraints within the binding pocket. To address these constraints at the R1 position, we explored a smaller fluoro substitution. The resulting compound, **22**, demonstrated improved activity against RARα, with an IC_50_ of 0.072 nM. Furthermore, **22** exhibited no activity against RARβ and RARγ at concentrations up to 100 nM, indicating exceptionally high potency and selectivity for RARα.

**Table 4:**
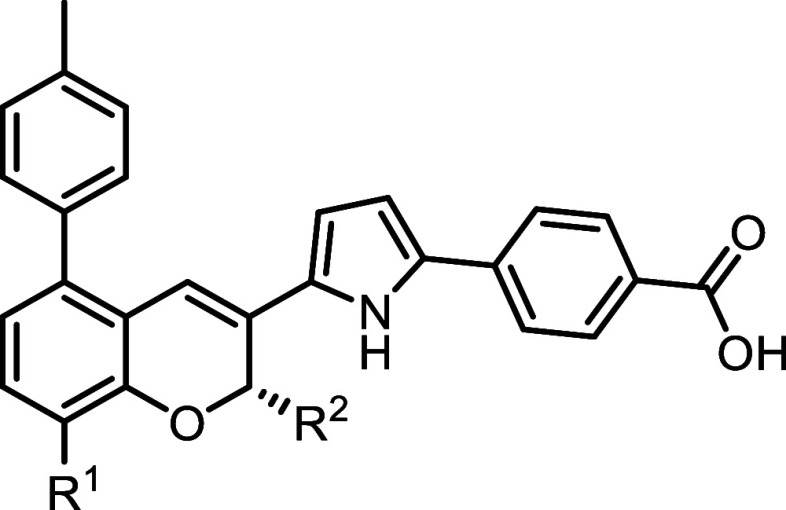
In Vitro Antagonism Activity of Analogs of **7** with Benzopyran Derivatizations^a^

				IC_50_ (nM)		
cmpd.	code number	*R* ^1^	*R* ^2^	RARα	RARβ	RARγ
**19**	**SR230329A**	CF_3_	H	0.18 ± 0.05	>100	>100
**20**	**SR211003B**	Et	H	0.15 ± 0.02	>100	>100
**21**	**SR210331F**	i-Pr	H	0.79 ± 0.09	>100	>100
**22**	**SR210505A**	F	H	0.072 ± 0.007	>100	>100
**23**	**SR210831C**	Me	Me	0.051 ± 0.006	84 ± 14	>100

a *n* = 3 independent experiments.

Another strategy was to introduce a methyl group at the R2 position to provide additional hydrophobic interactions with an adjacent unoccupied pocket. The resulting compound **23** exhibited remarkable activity against RARα, with an IC_50_ of 0.051 nM (Table 4). Additionally, **23** demonstrated outstanding selectivity, with 1647-fold selectivity over RARβ and more than 1960-fold selectivity over RARγ. These results highlight the success of effectively occupying the empty hydrophobic pocket near the pyran ring, significantly enhancing antagonism activity.

With the SAR information obtained, we concluded that fluoro substitution at the R1 position, methyl substitution at the R2 position, and monofluoro substitution at the ortho position of the benzoic acid group are beneficial for the benzopyran scaffold’s RARα activity (Table 5). Thus, we combined these features to evaluate their synergistic effects on RARα activity.

**Table 5:**
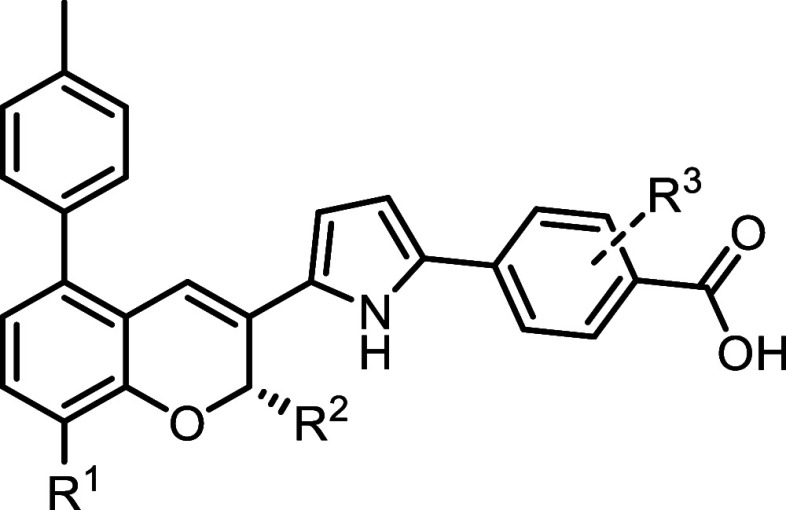
In Vitro Antagonism Activity of Analogs of **7** with Mixed Derivatizations^a^

					IC_50_ (nM)		
cmpd.	code number	*R* ^1^	*R* ^2^	*R* ^3^	RARα	RARβ	RARγ
**24**	**SR230210A**	F	H	*o*-F	0.081 ± 0.020	>100	>100
**25**	**SR231215C**	Me	Me	*o*-F	0.056 ± 0.006	16 ± 4	>100
**26**	**SR231215A**	F	Me	H	0.020 ± 0.003	4.0 ± 1.2	>100
**27**	**SR231215B**	F	Me	*o*-F	0.021 ± 0.004	2.6 ± 0.3	>100
**28**	**SR230329B**	CF_3_	H	*o*-F	0.19 ± 0.05	>100	>100
**29**	**SR230329C**	CF_3_	H	dual-*o*-F	2.8 ± 0.5	>100	>100

a *n* = 3 independent experiments.

We first designed and synthesized **24**, which incorporated a fluoro substitution at the R1 position and a monofluoro substitution at the benzoic acid ortho position (Table 5). **24** exhibited comparable IC_50_ values against RARα to its nonsubstituted counterpart (**22**) while maintaining a lack of activity against RARβ and RARγ at concentrations up to 100 nM.

Next, we combined a methyl substitution at the R2 position with a monofluoro substitution on the benzoic acid ortho position, resulting in **25** (Table 5). This analog retained similar activity and selectivity as its nonsubstituted counterpart (**23**) but showed significantly reduced RARβ selectivity, from 1647-fold to 285-fold.

Combining fluoro substitution at the R1 position with methyl substitution at the R2 position yielded compound **26**, which showed improved activity against RARα, with an IC_50_ of 0.020 nM (Table 5). However, compound **26** exhibited a 200-fold reduction in selectivity relative to RARβ. Further derivatization with a monofluoro group at the benzoic acid ortho position (**27**) did not affect its RARα activity or selectivity.

To address the potential metabolic liability of the pyran methyl group, we investigated CF_3_ substitutions at the R1 position, combined with a fluoro substitution at the benzoic acid ortho position (Table 5). The resulting compounds **28** and **29** were active and selective RARα antagonists. Among them, the monosubstituted analog **28** showed higher activity, with an IC_50_ of 0.19 nM.

### SAR Exploration of Analogs of **8**

Compound **8** (Figure 2) is a pyrrole-based RARα agonist with high potency (EC_30_ = 1.8 nM) and strong isoform selectivity. Building on the established strategy of converting RARα agonists to antagonists by introducing a bulky aromatic substituent, we evaluated this approach on the benzofuran scaffold of **8** to assess positional tolerance and SAR.

Two positions on the benzofuran ring, C20 and C23, were identified as viable sites for incorporation of an antagonism moiety (Table 6). Introduction of a toluene substituent at C20 (compounds **30** and **31**) did not confer antagonistic activity against any RAR subtype. In contrast, placement of the antagonism moiety at C23 produced active RARα antagonists (compounds **32** and **33**). Notably, compound **33**, bearing an additional para-methyl substituent on the toluene group, exhibited enhanced potency (IC_50_ = 0.55 nM) and retained greater than 180-fold selectivity over RARβ and RARγ (Table 6).

**Table 6:**
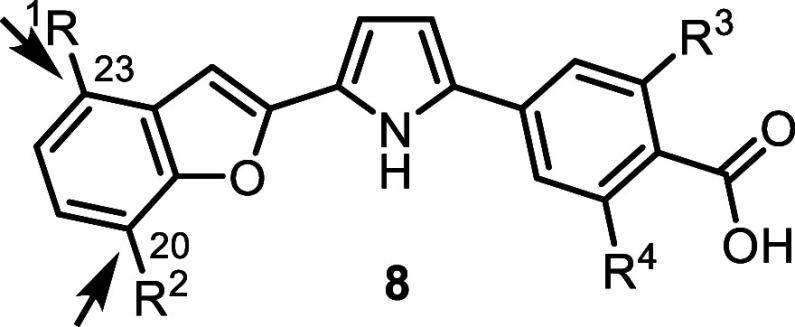

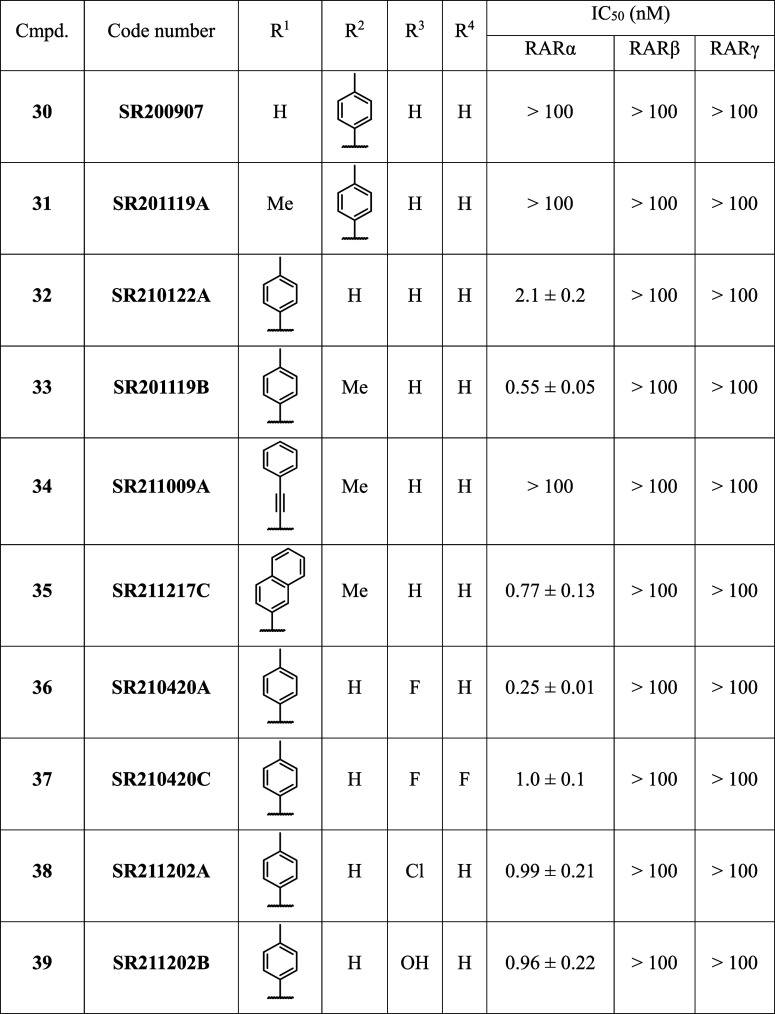
In Vitro Antagonism Activity of Analogs of **8** Carrying an Antagonism Moiety^a^

a *n* = 3 independent experiments.

Docking of compound **33** to RARα revealed a binding mode largely consistent with that of compound **9**, including extension of the antagonism moiety toward helix H12 to induce antagonism, occupation of the hydrophobic pocket by the bicyclic scaffold, a key interaction between the pyrrole NH and the RARα-specific residue S232, and anchoring of the distal benzoic acid through interactions with S287 and R276 (Figure 4).

**Figure 4:**
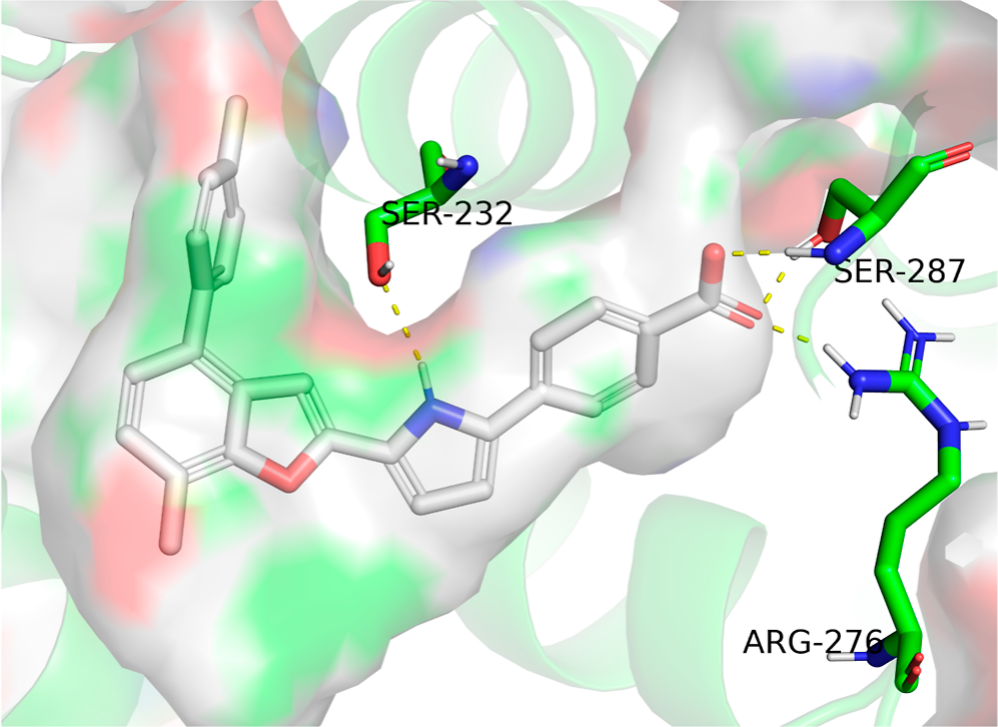
Predicted binding pose of **33** with RARα (PDB: 1dkf). The surface of the ligand-binding pocket is shown. Polar interactions between **33** and RARα are shown with yellow dashed lines.

Given the similar predicted binding conformations of compounds **33** and **9** within RARα, we applied an analogous modification strategy targeting the antagonism moiety, bicyclic core, and benzoic acid group (Table 6). These modifications produced SAR trends that closely paralleled those observed for the benzopyran series, underscoring the robustness of the design strategy across scaffolds. Unlike the benzopyran scaffold, a quinoline substitution on benzofuran could not be accessed synthetically in this series; therefore, a naphthalene group was introduced as an alternative. Notably, the naphthalene analog **35** retained activity comparable to its toluene counterpart **33**, in contrast to the quinoline substitution in the benzopyran scaffold, which led to a pronounced loss of activity, further supporting the highly hydrophobic nature of the antagonism-moiety binding pocket. Among this series, the *ortho*-fluoro-substituted analog **36** exhibited the highest activity, with an IC_50_ of 0.25 nM against RARα and no measurable activity against RARβ or RARγ at concentrations up to 100 nM.

We next explored the derivatization of the benzofuran core, identifying benzothiophene as a promising alternative because of its high structural similarity to benzofuran and its extensive use in drug discovery. Benzothiophene is a well-established moiety found in numerous approved drugs.^38^ Based on these attributes, we designed and synthesized benzothiophene-based analogs **40** and **41**, with the antagonism moiety incorporated at **C20** and **C23**, respectively (Table 7).

**Table 7:**
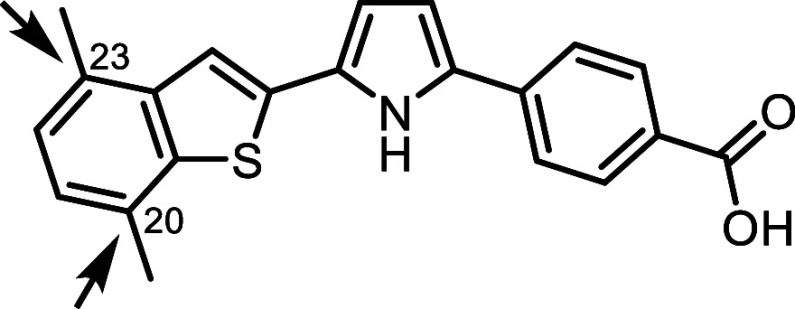

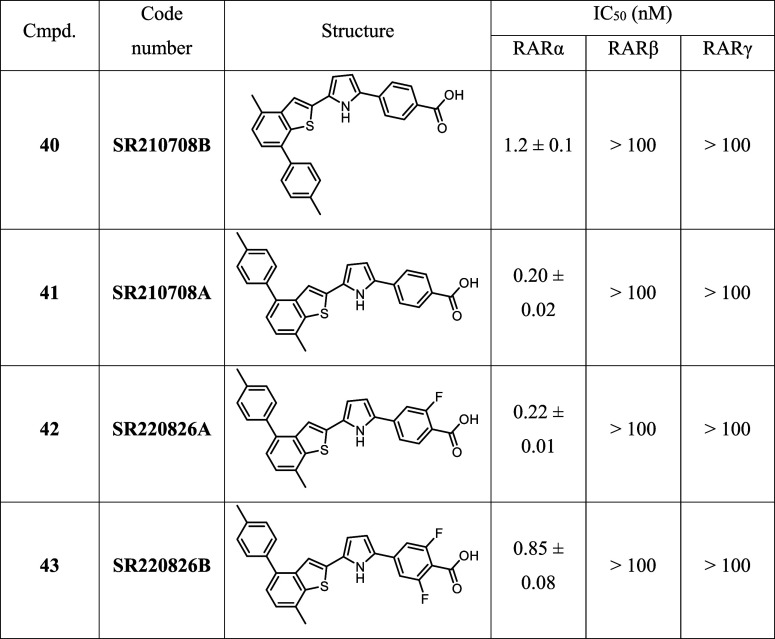
In Vitro Antagonism Activity of Analogs of **8** with Benzothiophene Derivatizations^a^

a *n* = 3 independent experiments.

In contrast to the benzofuran series, where effective antagonism was observed only for a single substitution orientation, the benzothiophene scaffold tolerated inversion of the heterocyclic ring orientation while retaining RARα binding, antagonist activity, and selectivity, indicating greater permissiveness for this scaffold within the receptor. The C23-incorporated analog **41** exhibited significantly higher activity, with an IC_50_ of 0.20 nM and over 500-fold selectivity against RARβ and RARγ. Further modifications of **41** were explored using the *ortho*-fluoro substitution strategy on the benzoic acid moiety. Monofluoro substitution (**42**) was well-tolerated, maintaining RARα activity, whereas difluoro substitution (**43**) resulted in a 4-fold reduction in activity.

Lastly, hydrophobic substitutions at the R1 position of **33** were explored, including ethyl (**44**), isopropyl (**45**), and trifluoromethyl (**48**) groups (Table 8). All three modifications enhanced RARα activity relative to the methyl parent compound **33** while preserving high selectivity over RARβ and RARγ. Among these, the trifluoromethyl analog **48** showed the greatest potency, with an IC_50_ of 0.10 nM (Table 8).

**Table 8:**
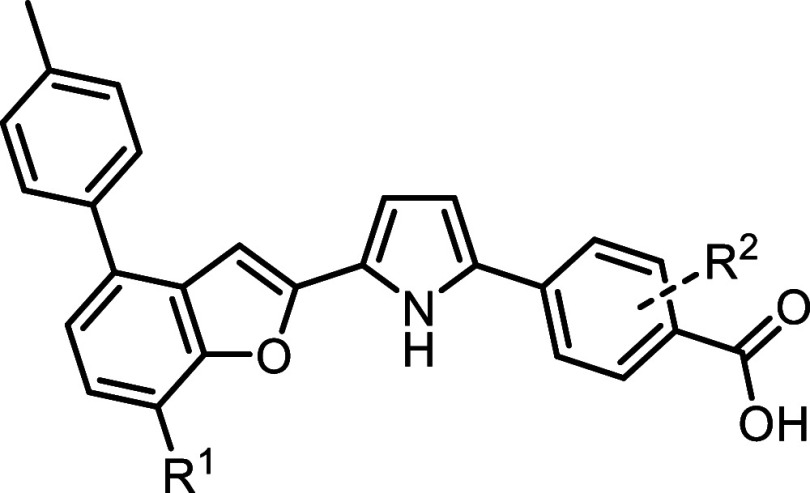
In Vitro Antagonism Activity of Analogs of **8** with Benzofuran Derivatizations^a^

				IC_50_ (nM)		
cmpd.	code number	*R* ^1^	*R* ^2^	RARα	RARβ	RARγ
**44**	**SR211003A**	Et	-	0.20 ± 0.02	>100	>100
**45**	**SR210401A**	iPr	-	0.18 ± 0.02	>100	>100
**46**	**SR220908A**	iPr	*o*-F	0.14 ± 0.03	>100	>100
**47**	**SR220908B**	iPr	dual-*o*-F	0.25 ± 0.04	>100	>100
**48**	**SR230329D**	CF_3_	-	0.10 ± 0.02	>100	>100
**49**	**SR230329E**	CF_3_	*o*-F	0.094 ± 0.014	>100	>100
**50**	**SR230329F**	CF_3_	dual-*o*-F	0.62 ± 0.06	>100	>100

a *n* = 3 independent experiments.

We then incorporated fluoro substitutions at the ortho position of the benzoic acid moiety and observed a consistent pattern: monofluoro substitution increased activity, whereas difluoro substitution decreased activity (Table 8). The combination of a trifluoromethyl substitution at the R1 position and monofluoro substitution on the benzoic acid moiety (**49**) resulted in the highest activity, with an IC_50_ of 0.094 nM and over 1063-fold selectivity against RARβ and RARγ.

### ADMET Evaluation of Benzopyran, Benzofuran, and Benzothiophene Analogs

With the SAR data in hand, we identified key structural modifications that significantly enhance RARα activity and selectivity across the benzopyran, benzofuran, and benzothiophene scaffolds, leading to the selection of **22**, **23**, **42**, and **46** for further evaluation. These compounds demonstrated exceptional potency and selectivity. The previously reported RARα antagonist **YCT-529** exhibited an IC_50_ of 1.2 nM against RARα, with substantially weaker activity against RARβ (>430 nM) and RARγ (370 nM) under the same assay conditions.^32^ In comparison, compounds **22**, **23**, **42**, and **46** show markedly improved potency and selectivity, underscoring the impact of the structural optimizations described herein.

Transitioning from SAR studies to ADMET profiling enables us to evaluate the drug-like properties of these potent and selective compounds, including pharmacokinetics and safety, which are essential for advancing the most promising candidates toward preclinical development. The ADMET evaluations were carried out by Pharmaron. To improve kinetic solubility, the analogues were converted to their sodium salts by reaction with 1 equiv of sodium methoxide.

The liver microsome metabolic stability tests revealed that **22**, **23**, **42**, and **46** (tested as sodium salts) exhibited high stability. Among the benzopyran analogues, **23** demonstrated greater stability in both human and mouse liver microsomes, while **22** was more prone to liver microsomal metabolism in both species (Table 9). The benzothiophene analogue **42** showed no detectable metabolism in either human or mouse liver microsomes, highlighting its exceptional stability (Table 9). Similarly, the benzofuran analogue **46** showed no metabolism in human liver microsomes and low metabolism in mouse liver microsomes, with a half-life (*T*_1/2_) of 400 min, indicating its favorable metabolic stability (Table 9).

**Table 9:** Metabolic Stability of Test Compounds in Pooled Human and Male Mouse Liver Microsomes

cmpd.	species	*T*_1/2_ (min)	CL_int_ (μL/min/mg protein)	scaled-up CL_int_ (mL/min/kg)
**22**	human	175	7.93	9.94
	mouse	216	6.40	28.0
**23**	human	3800	0.36	0.46
	mouse	∞	0.00	0.00
**42**	human	∞	0.00	0.00
	mouse	∞	0.00	0.00
**46**	human	∞	0.00	0.00
	mouse	401	3.46	15.1

To develop a practical male contraceptive therapy, ensuring user compliance is crucial, and a daily oral pill is the preferred route of administration, mirroring the success of female contraceptives. To assess the oral bioavailability of the selected compounds, we examined their sodium salts for Caco-2 permeability. As shown in Table 10, the benzothiophene analogue **42** and the benzofuran analogue **46** exhibited low Caco-2 permeability and high efflux ratios, making them less desirable for further development. In contrast, benzopyran compounds **22** and **23** demonstrated moderate Caco-2 permeability, with **23** showing a particularly low efflux ratio, indicating its suitability for further development as an oral contraceptive.

**Table 10:** Permeability Results of Test Compounds in Caco-2 Cells

cmpd.	P_app__(A‑B)_ (10^–6^ cm/s)	P_app__(B‑A)_ (10^–6^ cm/s)	efflux ratio	recovery (%) AP-BL	recovery (%) BL-AP
**22**	0.50	1.52	3.04	71.1	102
**23**	0.68	1.11	1.64	55.3	80.0
**42**	<0.17	1.29	>7.74	<63.3	91.0
**46**	<0.24	0.75	>3.11	<62.8	87.5

Next, we conducted a more comprehensive ADMET evaluation of **23** (Table 11). The compound demonstrated high hepatocyte stability, with half-lives of 123 and 64 min in human and mouse hepatocytes, respectively. To improve its kinetic solubility, **23** was converted to its sodium salt by reaction with 1 equiv of sodium methoxide. This modification significantly enhanced solubility in PBS at physiological pH, achieving a maximum concentration of 78 μM.

**Table 11:**
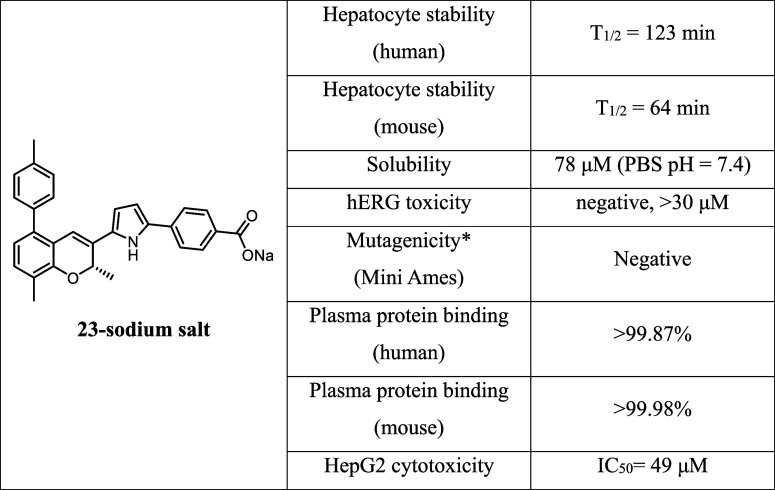
Further ADMET Evaluations of the Sodium Salt of **23**

a Test strains: TA98, TA100; exogenous metabolic activation: presence and absence of S9.

For toxicity evaluation, **23** underwent hERG inhibition testing to assess the risk of cardiovascular toxicity, mini-Ames testing for mutagenicity, and HepG2 viability testing for liver toxicity. The compound raised no toxicity concerns in any of these assays. However, it exhibited very high plasma protein binding in both human and mouse plasma, with over 99% bound. This is consistent with other RAR antagonists, such as **YCT-529**, which also possess highly lipophilic scaffolds and terminal carboxylic acid groups. Despite its high plasma protein binding, **YCT-529** has demonstrated significant in vivo efficacy for inducing male contraceptive effects. This may be attributed to factors such as plasma protein-facilitated transport to target tissues and its high potency as an RARα antagonist, ensuring sufficient free drug to achieve the desired pharmacological effect.

On the basis of its favorable ADMET profile, **23** met our preliminary drug development criteria, and we advanced it to in vivo studies for further evaluation.

We next examined the pharmacokinetic profile of **23** in CD1 mice (Figure 5). The sodium salt of **23** was administered orally at 10 mg/kg, and plasma and testis concentrations were measured at 0.25, 0.5, 1, 2, 4, 8, 16, and 24 h postdosing. The compound exhibited high oral bioavailability, with a maximum plasma concentration of 4992 ng/mL, exceeding 10 μM. Additionally, **23** demonstrated strong testis distribution, reaching a peak concentration of 2441 ng/g, indicating sufficient exposure in the target tissue. The compound also exhibited a prolonged retention time, with a half-life of approximately 12 h in both plasma and testis, supporting its potential for sustained pharmacological activity. At the same 10 mg/kg oral dose, the previously reported RARα antagonist **YCT-529** achieved markedly lower exposure, with plasma and testis *C*_max_ values each below 1000 ng/mL (or ng/g).^29^ Although unbound concentrations cannot be reliably calculated due to high plasma protein binding, the substantially higher total plasma and testis exposure observed for compound **23** indicates improved oral bioavailability and tissue distribution relative to **YCT-529**, supporting its advancement for in vivo evaluation.

**Figure 5:**
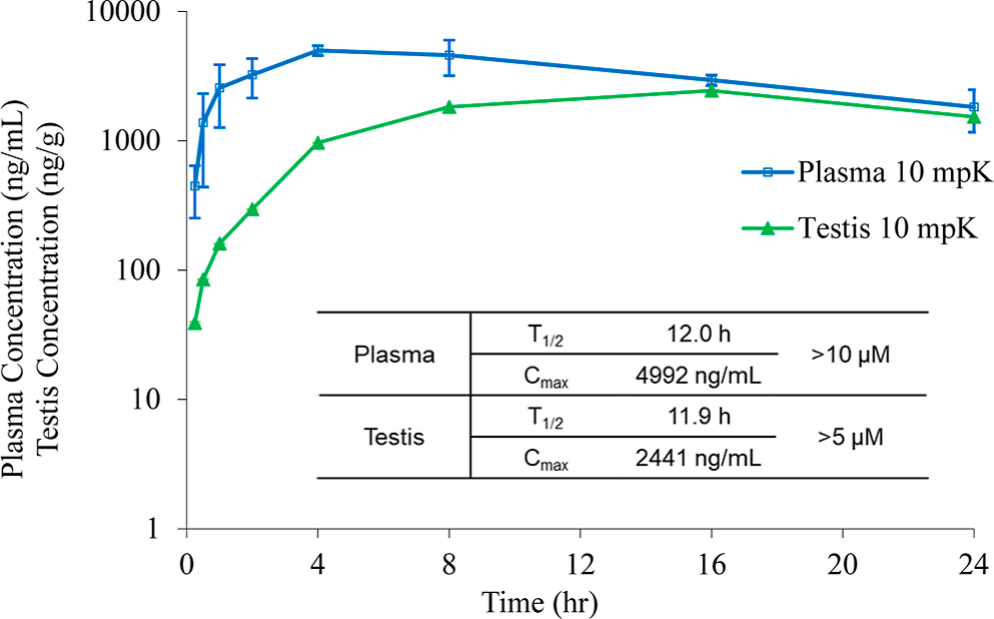
Pharmacokinetic study of **23**. The sodium salt of analog **23** was orally administered at a dose of 10 mg/kg to CD1 mice.

### In Vivo Efficacy Evaluation of **23**

Given the acceptable pharmacokinetic profile of **23**, we evaluated its in vivo effects as a male contraceptive. To determine whether **23** elicited the characteristic effects on spermatogenesis observed in Rara^–/–^ mice and in mice treated with **BMS-189453**,^17,39^ an initial pilot experiment was conducted. Compound **23** was administered orally as its sodium salt at 10 mg/kg/day for 28 days (*n* = 7) in CD1 mice. One day after cessation of drug treatment (CDT), we observed a significant reduction in sperm counts (Figure 6A), with treated mice showing counts of 2.6 ± 0.9 × 10^6^, compared with the standard control of 55.1 ± 8.5 × 10^6^, indicating a more than 20-fold decrease. Additionally, testicular weight significantly decreased (Figure 6B), with treated mice exhibiting weights of 205 ± 8 mg compared with the control group’s 266 ± 11 mg. These reductions in sperm count and testicular weight align with the effects seen in Rara^–/–^ mice and in those treated with **BMS-189453**. Importantly, there was no significant change in body weight after CDT (Figure 6C), indicating the absence of systemic toxicity. These results highlight the potential of **23** as an effective and well-tolerated male contraceptive.

**Figure 6:**
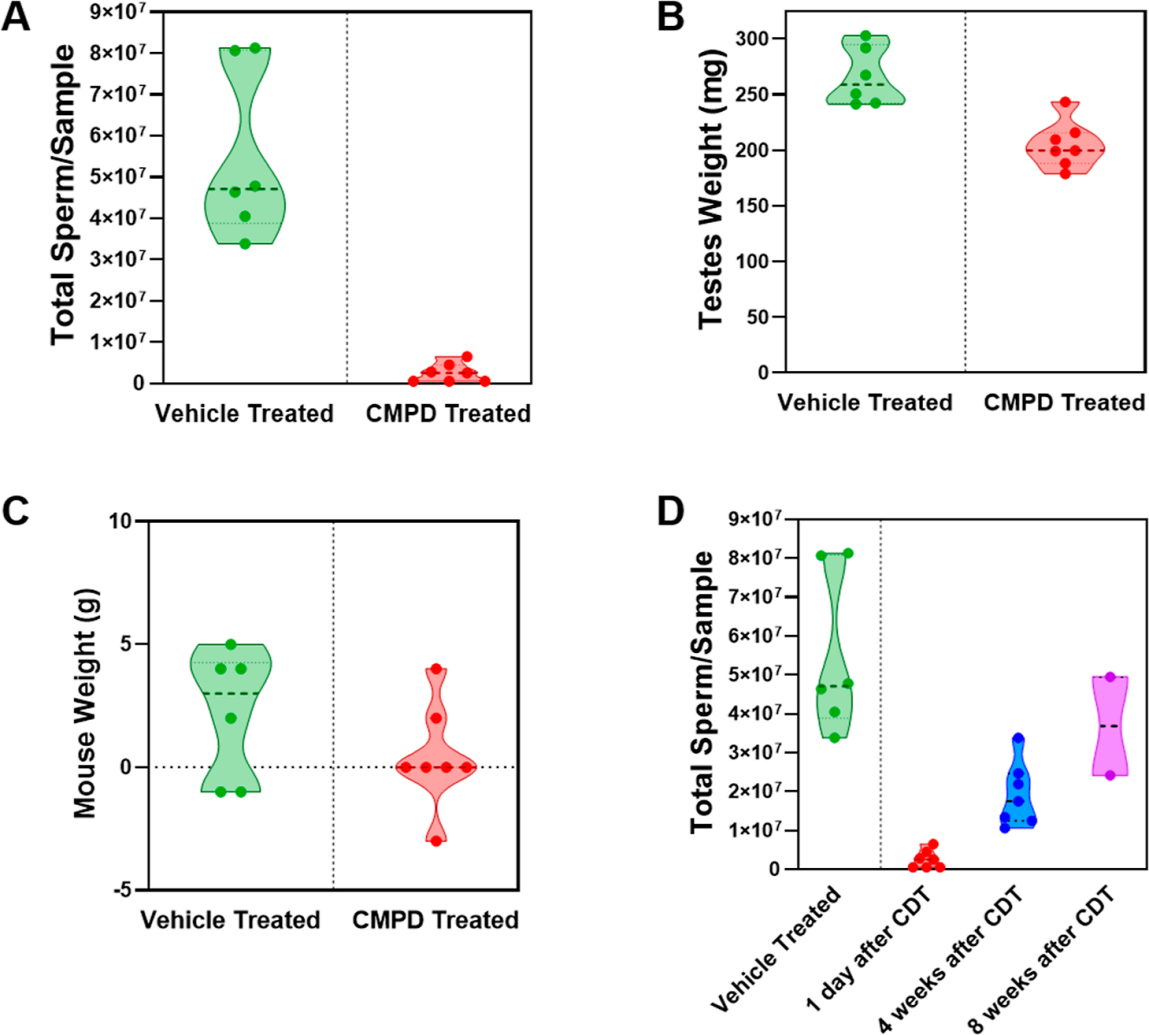
In vivo evaluation of **23**’s contraceptive effect. CD1 mice were treated with the sodium salt of **23** orally at 10 mg/kg/day for 28 days. Total sperm count (A), testis weight (B), and mouse weight change (C) in treated mice after CDT were compared with those in vehicle control mice. (D) Sperm counts were measured 1 day after CDT and compared with those after 4 and 8 weeks of the washout phase.

A key requirement for male contraceptive development is the reversibility of its effects, following treatment withdrawal. To assess the reversibility of the **23**-induced inhibition of spermatogenesis, we conducted a washout study. Mice were treated with **23** at 10 mg/kg/day for 28 days, as in the initial experiments, and were monitored after CDT. Testis samples were collected at 4 weeks and 8 weeks post-CDT to evaluate sperm recovery. As illustrated in Figure 6D, sperm counts showed a time-dependent recovery over the 8 week washout period, with approximately 40% recovery at 4 weeks CDT and full recovery by 8 weeks post-CDT. This aligns with the spermatogenesis cycle in mice, which takes approximately 35 days^40^ and confirms that the inhibition of spermatogenesis caused by **23** at 10 mg/kg/day is reversible.

To determine the minimum effective dose of **23** required to induce male contraception, a dose-response study was conducted. Mice were treated with **23** at doses of 10, 3, 1, and 0.3 mg/kg/day for 28 days. Complete inhibition of spermatogenesis was observed in all groups except the 0.3 mg/kg/day group, which showed a slight reduction in the inhibitory effect (Figure 7A). To further characterize the dose-response relationship, additional studies were performed with doses ranging from 1 mg/kg/day to 0.03 mg/kg/day. A dose-dependent effect on spermatogenesis was observed within this range with no inhibitory effect detected at 0.03 mg/kg/day (Figure 7B). Complete inhibition of spermatogenesis was observed in the 1 mg/kg and 0.3 mg/kg groups, while a reduced inhibitory effect was noted in the 0.1 mg/kg group (Figure 7B). On the basis of these findings, we concluded that the minimum effective dose of **23** for inducing full male contraceptive effects lies between 0.3 and 1 mg/kg/day. This represents a significant improvement over that of **YCT-529**, which has a minimum effective dose of approximately 7.5 mg/kg/day. The 7.5- to 25-fold increase in potency suggests a lower required dose for clinical use and a potentially larger therapeutic window.

**Figure 7:**
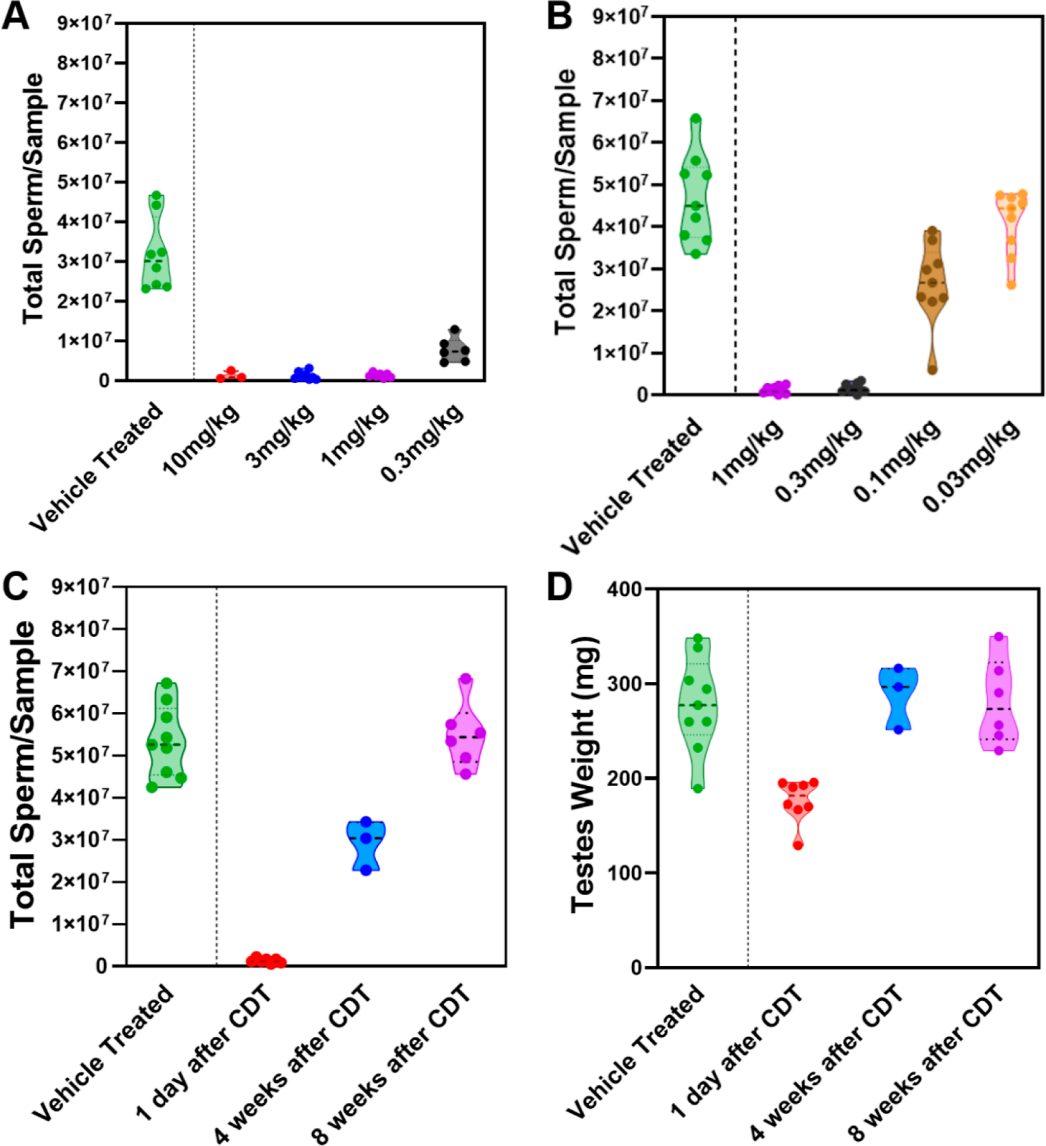
Dose-response evaluation of **23**’s spermatogenesis inhibitory effect. CD1 mice were treated with the sodium salt of **23** orally at 0.3–10 mg/kg/day (A) or 0.03–1 mg/kg/day (B) for 28 days. Sperm counts were measured 1 day after cessation of drug treatment (CDT) and compared with those of the vehicle control group. Sperm counts (C) and testis weight (D) of mice treated at 1 mg/kg/day were measured 1 day after CDT and compared with counts after 4 and 8 weeks of the washout phase.

More comprehensive reversibility studies were performed in mice treated with compound **23** at 1 mg/kg/day, as shown in Figure 7C,D. Consistent with findings in the 10 mg/kg/day group, sperm counts showed a time-dependent recovery over the 8 week washout period, with partial recovery at 4 weeks CDT and complete recovery by week 8. Notably, testicular weight had already returned to baseline by 4 weeks post-CDT, preceding the full recovery of sperm counts. These findings reinforce the reversibility of the contraceptive effect of compound **23** and demonstrate that testicular recovery occurs in advance of complete sperm-count restoration.

### Testicular Drug Spatial Distribution of Compound **23**

Given the impressive in vivo activity of compound **23**, we wanted to explore the mechanism by which this analogue is reducing sperm counts. **ATRA** is required for the conversion of undifferentiated spermatogonia type A_a1_ to differentiated A_1_ spermatogonia,^23^ a process that takes place before the blood–testis barrier (BTB).^41^ **ATRA** is also required for spermiogenesis, which involves the initiation of spermatid elongation and the release of spermatozoa from the Sertoli cells into the seminiferous tubule.^23^ Inhibiting this process would require the RAR inhibitor to cross the BTB.^41^ **ATRA** controls both processes by regulating the expression of downstream signaling genes. While some reports have implicated **ATRA** in the initiation of meiosis, a recent report showed that RA is not required for its initiation, progression, or completion.^42^ We employed imaging mass spectrometry (IMS) technology to map the distribution of compound **23** in mouse testis tissue. Three groups were used in these studies, including mouse testis collected at 0 h (control), 8 h, and 16 h post-treatment at an oral dose of 10 mg/kg. In the control group, compound **23** was not detected (Figure 8A). The concentration level of **23** increased with time (Figure 8B,C). Overlaid images (last two columns of Figure 8B,C) indicated that compound **23** did not cross the BTB and is mainly distributed outside of the seminiferous tubules. Thus, the effect of compound **23** should take place during the conversion of undifferentiated spermatogonia type Aa1 to differentiated A1 spermatogonia.

**Figure 8:**
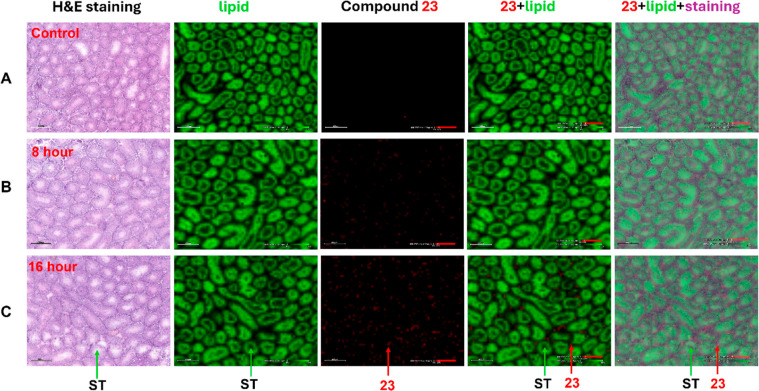
Time-dependent spatial distribution of compound **23** in transverse sectioned mouse testis. The IMS analysis was performed with a Thermo Q Exactive Orbitrap MS mounted with a Spectroglyph MALDI Injector, and the images were visualized using SCiLS Lab (Bruker). Compound ion ([M-H]^−^, *m*/*z* 434.1767 ± 10 ppm) and a lipid ([M-H]^−^, *m*/*z* 809.5189 ± 10 ppm) were extracted. (A) Control group. (B) 8 h post dose. (C) 16 h post dose. The columns, in order, are Hematoxylin and Eosin (H&E) staining, lipid outlining of seminiferous tubules (ST), compound **23**, compound **23** with lipid, and compound **23** with lipid plus staining. Image of H & E staining (purple), lipid (green), and **23** (red). Scale bar, 400 μm.

### Chemistry

We initiated the synthesis of the benzopyran scaffold with **10**, featuring an antagonism moiety on the C3 position (Table 1). As shown in Scheme 1, the synthesis began with a magnesium chloride-mediated aldehyde incorporation, converting the commercial 2-bromo-5-methylphenol (**51**) into benzaldehyde **52**. Compound **52** was then reacted with acrylaldehyde to undergo cyclization, forming benzopyran aldehyde **53**. Compound **53** was further reacted with methyl 4-acryloylbenzoate, catalyzed by the organocatalyst 3-benzyl-5-(2-hydroxyethyl)-4-methylthiazol-3-ium chloride, yielding diketone **54**. A Paal–Knorr reaction was employed to cyclize diketone **21** into pyrrole **55**. Finally, a one-pot reaction was conducted, starting with a Suzuki cross-coupling to incorporate the toluene antagonism moiety, followed by saponification with additional lithium hydroxide and water, producing the final compound **10**.

**Scheme 1:**
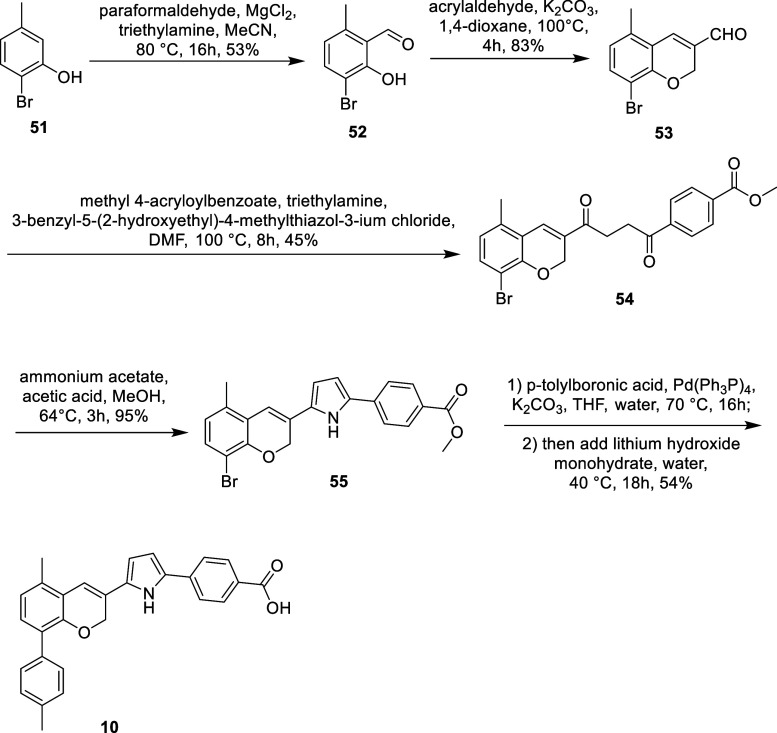
Preparation of Benzopyran Analog **10**

To streamline the synthesis of benzopyran analogues bearing an antagonism moiety at the C6 position, we adopted a Suzuki coupling-based synthetic scheme. This approach divides the compound into two modular building blocks: the pyrrole core (Scheme 2) and the benzopyran core (Scheme 3). These two fragments were cross-coupled via a Suzuki reaction, enabling more efficient derivatization and exploration of structural variations.

**Scheme 2:**
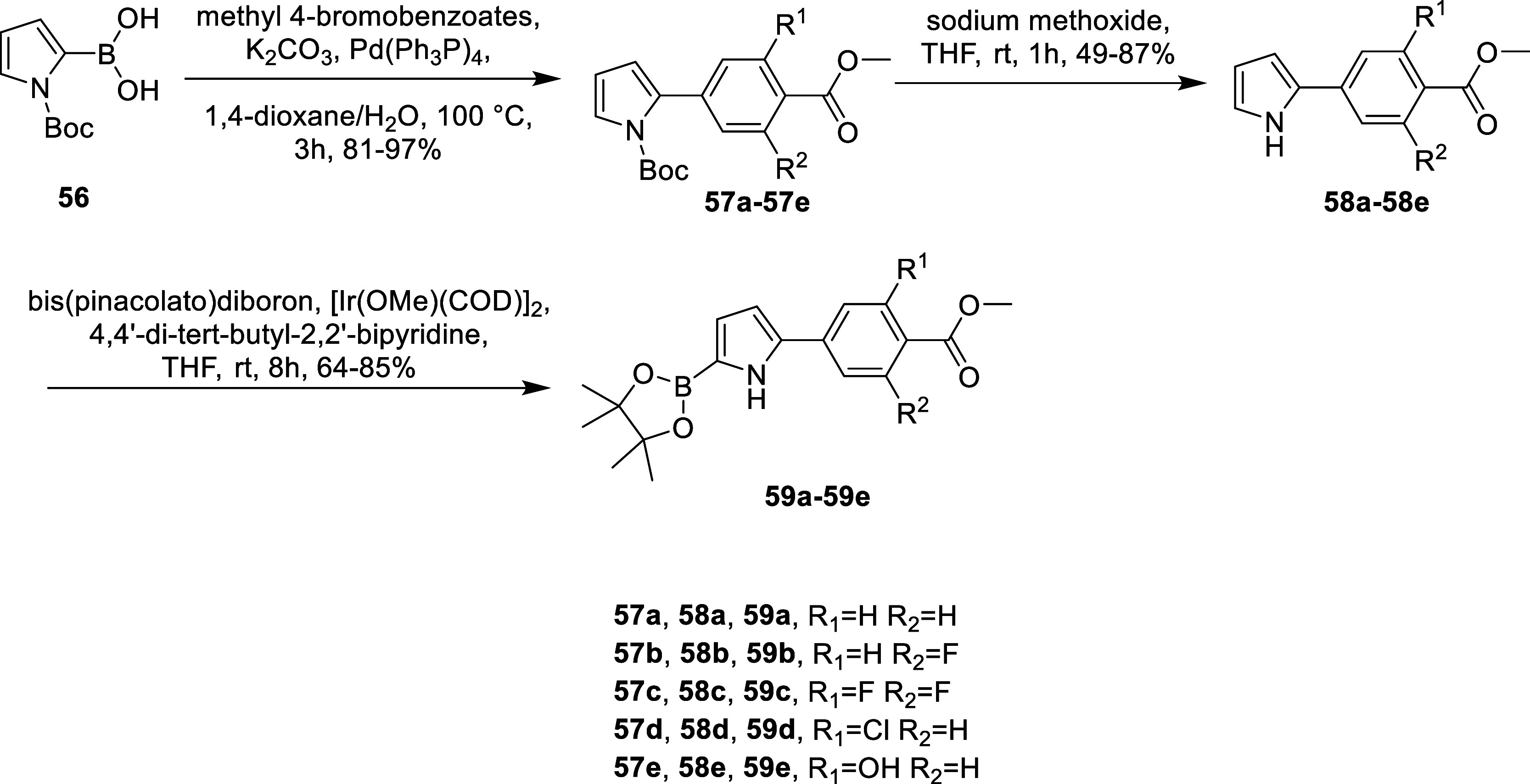
General Synthetic Scheme for the Pyrrole Building Blocks

**Scheme 3:**
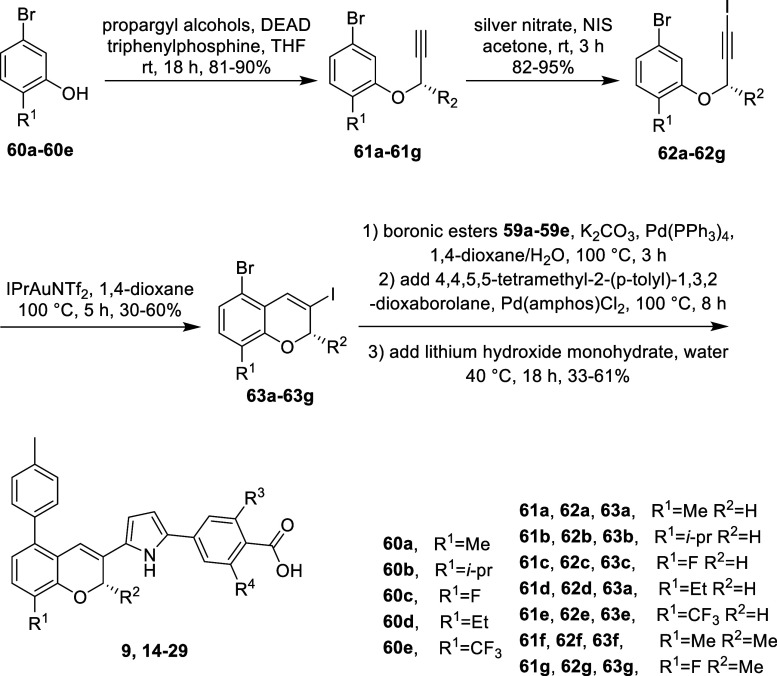
General Synthetic Scheme for Benzopyran Analogs with an Antagonism Moiety on C6

For the pyrrole building blocks, the synthesis begins with a Suzuki coupling reaction between *N*-Boc-pyrrole-2-boronate and ortho-substituted 4-bromobenzoate esters, yielding intermediates **57a**-**57e**. These intermediates undergo mild Boc deprotection using sodium methoxide to produce free pyrrole intermediates **58a**-**58e**. Subsequently, iridium-catalyzed C–H activation is employed to achieve C5 borylation of the pyrrole, yielding pyrrole boronic esters **59a**-**59e** (Scheme 2).

For the synthesis of the benzopyran building blocks and subsequently the final compounds, the synthesis began with a Mitsunobu reaction to produce the alkyne ether intermediates **61a**-**61g**. These intermediates were subsequently iodinated using silver nitrate as a catalyst and NIS as the iodine source, yielding intermediates **62a**-**62g**. The iodinated intermediates were then cyclized to form benzopyrans **63a**-**63g**, catalyzed by IPrAuNTf_2_. In a one-pot reaction, the benzopyran intermediates underwent Suzuki coupling with pyrrole boronic acid pinacol esters **59a**-**59e** to generate disubstituted pyrroles. Excess toluene boronic acid pinacol ester and additional palladium catalyst were then added to the reaction mixture to incorporate the antagonism moiety. Finally, saponification was achieved by adding lithium hydroxide and water, producing the final unprotected RAR antagonist compounds (Scheme 3).

For **12** and **13**, featuring phenylacetylene and quinoline as the antagonism moieties instead of toluene, their syntheses are outlined in Scheme 4. Benzopyran **63a** was first cross-coupled with pyrrole boronic acid pinacol ester **59a**, yielding disubstituted pyrrole intermediate **64**. Intermediate **64** was then subjected to a Sonogashira reaction with phenylacetylene, followed by saponification to remove the methyl ester and yield **13**. Alternatively, intermediate **64** underwent a Suzuki reaction with a quinoline boronic acid pinacol ester. Lithium hydroxide and additional water were added to the reaction mixture to demethylate the methyl ester, which yielded **12**.

**Scheme 4:**
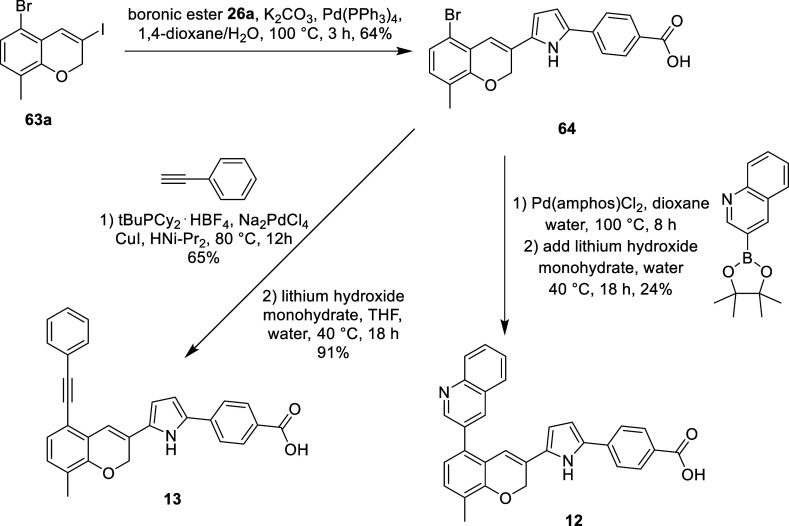
Preparation of Benzopyran Analogs **13** and **12**

For compound **11**, featuring the toluene antagonism moiety incorporated at the C10 position (Table 3.1), the synthesis began with a one-pot reaction to form a phenyl ether by reacting alcohol **65** with diphenyliodonium trifluoromethanesulfonate. It was then followed by cyclization using NIS and the Lewis acid BF_3_·OEt_2_,^43^ yielding the benzopyran intermediate **66**. Intermediate **66** was then cross-coupled with pyrrole boronic acid pinacol ester **59a** to form intermediate **67**, which underwent saponification to deprotect the methyl ester, resulting in the final compound, **11** (Scheme 5).

**Scheme 5:**
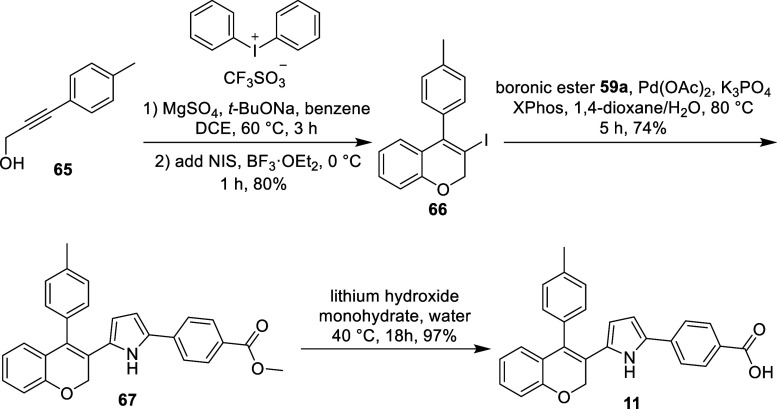
Preparation of Benzopyran Analog **11**

For the benzofuran and benzothiophene analogs, the synthesis began with a nucleophilic substitution reaction, yielding intermediates **69a**-**69i**. These intermediates were then cyclized under acidic conditions to produce benzofuran and benzothiophene cores, **70a**-**70i**. A Suzuki reaction was subsequently employed to introduce the antagonism moiety, yielding intermediates **71a**-**71j**. The intermediates **71a**-**71j** were then iodinated to form **72a**-**72j**. Finally, these iodinated intermediates underwent a one-pot Suzuki coupling with pyrrole boronic acid pinacol esters **59a**-**59e**, followed by saponification, to yield the final compounds (Scheme 6).

**Scheme 6:**
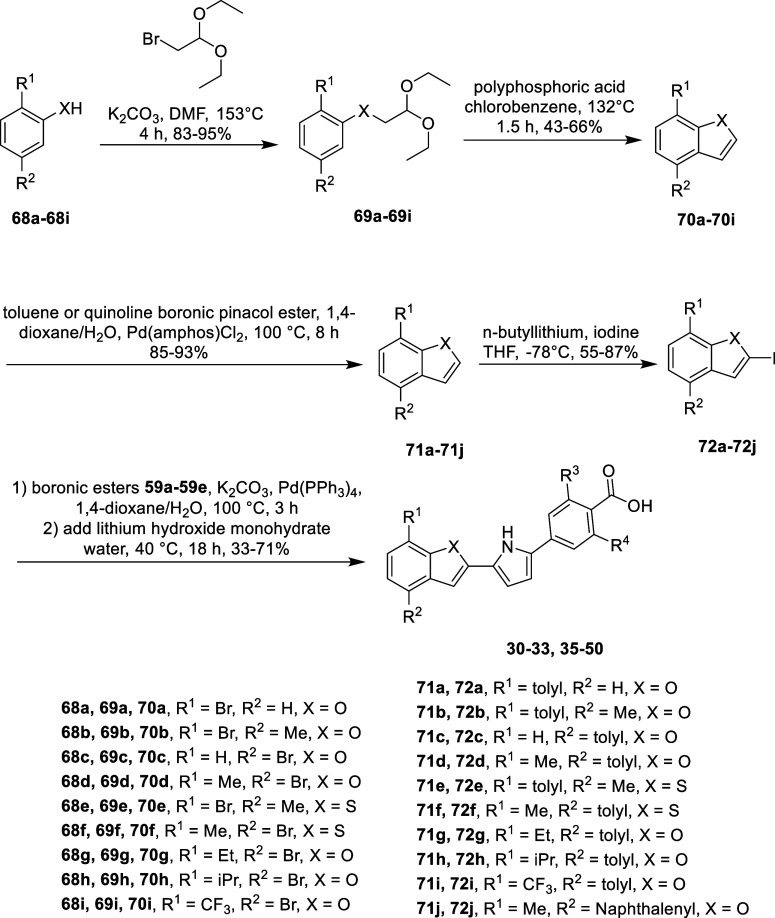
General Synthetic Scheme for Benzofuran and Benzothiophene Analogs

For the synthesis of **34**, we began with a Sonogashira reaction to convert bromobenzofuran **70d** into intermediate **73**. Intermediate **73** was then iodinated to give intermediate **74**. The subsequent Suzuki coupling of intermediate **74** with pyrrole boronic ester **59a** afforded intermediate **75**. Finally, intermediate **75** was subjected to saponification, yielding the final compound **34** (Scheme 7).

**Scheme 7:**
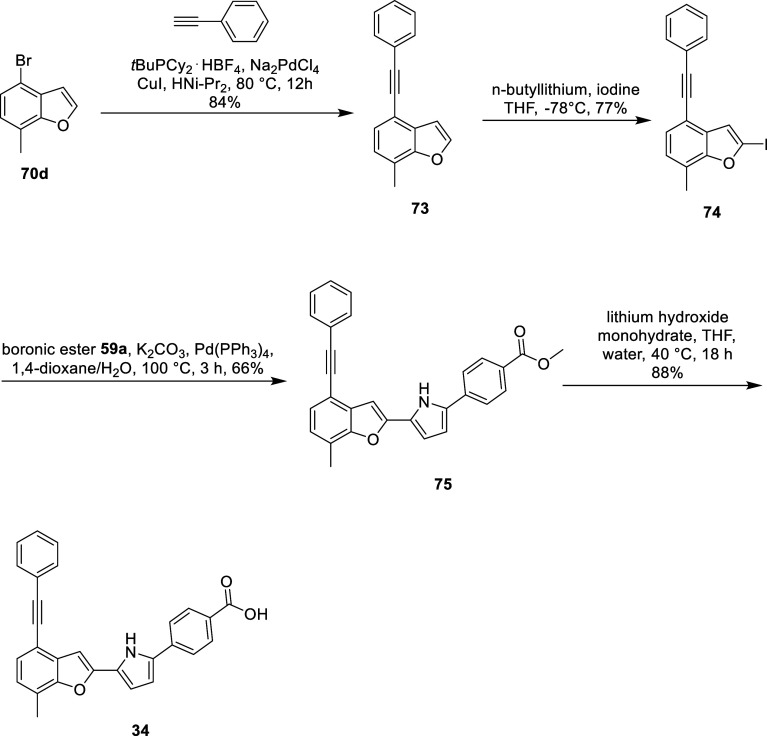
Synthesis of **34**

## Discussion and Conclusions

We report the rational design, synthesis, and evaluation of novel retinoic acid receptor alpha (RARα)-selective antagonists with benzopyran, benzofuran, and benzothiophene scaffolds for the development of male contraceptives. Building on the success of **YCT-529**, the first oral, effective, and reversible RARα-selective male contraceptive, this work was motivated by the recognition that the **YCT-529** scaffold is limited in its tolerance to structural modification, thereby constraining further optimization of potency, selectivity, and pharmacokinetic properties. Accordingly, we pursued structurally distinct second-generation scaffolds designed to retain RARα selectivity while offering greater medicinal chemistry flexibility, and improved developability.

SAR studies identified critical features influencing activity, such as the optimal positioning of the antagonism moiety and substituent patterns compatible with the RARα binding pocket. Among the benzopyran series, compound **23** emerged as a lead with exceptional potency (IC_50_ = 0.051 nM) and pronounced selectivity (>1650-fold over RARβ and >1960-fold over RARγ), representing a substantial improvement in both activity and selectivity relative to **YCT-529** under the same assay conditions. Benzofuran and benzothiophene analogs such as **42** and **46** further demonstrated that high potency and selectivity could be achieved across multiple scaffolds, underscoring the robustness of the design strategy.

Extensive ADMET profiling revealed that **23** possesses high metabolic stability, favorable solubility, and no systemic toxicity. Its pharmacokinetic studies in CD1 mice confirmed high oral bioavailability, strong testis-specific distribution, and a prolonged half-life, supporting its potential for sustained contraceptive effects. In vivo efficacy studies demonstrated significant reversible inhibition of spermatogenesis, with a minimum effective dose of 0.3–1 mg/kg/day, representing a 7.5- to 25-fold improvement in potency over **YCT-529**. We also analyzed the spatial distribution of compound **23** in mouse seminiferous tubules using imaging mass spectrometry technology. We found that **23** does not cross the BTB and exerts its activity during the conversion of undifferentiated spermatogonia type Aa1 to differentiated A1 spermatogonia. Collectively, these results establish compound **23** as a highly promising second-generation RARα-selective antagonist with superior potency, selectivity, and developability, and further validate selective RARα antagonism as a viable and translational strategy for male contraception.

## Experimental Section

### Docking Studies

An SD file containing the structures of the retinoid analogs was generated in ChemDraw and imported into Maestro, Schrodinger’s molecular modeling suite. Within the project workspace, bond orders were assigned, hydrogens were added, and all ionization states were generated using Epik (pH 5.0–9.0), allowing desalting and tautomerization while retaining chiralities (a maximum of 32 states per ligand). Only the lowest energy ring conformation per ligand was retained for docking. For protein preparation, the PDB file (1dkf) was imported with mixed hydrogen display. Preprocessing involved assigning bond orders, adding hydrogens, creating zero-order bonds to metals, forming disulfide bonds, and removing water molecules beyond 5 Å from the ligand-binding site. Epik was used to generate protonation states, and the sample water orientation was optimized. A restrained minimization was performed using the OPLS 2001 force field. Next, Receptor Grid Generation was performed by selecting the cocrystallized ligand 2b as the reference binding site. Glide Receptor Grid Generation was performed with a van der Waals scaling factor of 1.0 and a partial charge cutoff of 0.25, softening the potential for nonpolar receptor regions. Finally, Ligand Docking was conducted using Glide in standard precision (SP) mode, with nitrogen inversion and ring conformation sampling enabled. Nonplanar amide conformations were biased with penalties applied, and Epik state penalties were incorporated. A maximum of 10 poses per ligand was allowed, followed by postdocking minimization. The PDB files for the docking studies with SR200831A and SR201119B are provided in the Supporting Information.

### RAR Transactivation Assay

The antagonism and agonism potency of test compounds of RARα, RARβ, and RARγ was evaluated using the GeneBLAzer RAR-UAS-bla HEK 293T cell-based reporter assay (Thermo Scientific, Waltham, MA). These HEK 293T cells stably express a recombinant human RAR subtype fused to a GAL4 DNA-binding domain, along with a beta-lactamase reporter gene under the control of an upstream activator sequence (UAS). When an agonist binds to the ligand-binding domain (LBD) of the GAL4-RAR fusion protein, it activates transcription of the beta-lactamase reporter gene. The level of beta-lactamase expression was quantified by measuring cleavage of the FRET substrate CCF4-AM. For antagonist testing, 0.8 nM of **ATRA** in DMSO (final 0.1%) was added to control and test compound wells using an Echo acoustic nanoliter dispenser (Labcyte, San Jose, CA). Test and reference compounds were added in an 8-point dose-response format in triplicate. For agonist testing, compounds were added in an 8-point dose-response format using an Echo without **ATRA**. RARα, RARβ, and RARγ cells were serum-starved for 16–24 h before assay initiation, and 40 μL of cell suspension (2.5 × 10^5^ cells/mL) was added per well. Plates were incubated at 37 °C in a 5% CO_2_ incubator for 16–24 h. Following incubation, 8 μL of beta-lactamase detection reagent containing CCF4-AM was added, and plates were incubated at room temperature for 2 h. Fluorescence was measured using a SpectraMax M2e plate reader (Molecular Devices, San Jose, CA) with excitation at 410 nm and emission at 460 and 520 nm. After background subtraction, the 460/520 emission ratio was calculated, and IC_50_ and EC_50_ values were determined using a four-parameter logistic equation in GraphPad Prism 8.0. Mean ± SEM values were calculated from the geometric mean of the log IC_50_ and EC_50_ values.

### Animal Studies

All animal experiments were performed in accordance with protocols evaluated and approved by the University of Minnesota Institutional Animal Care and Use Committee (IACUC Protocol 2503-42812A).

Animals and Housing: Male CD1 mice (8–10 weeks, Envigo, Indianapolis) were used in all experiments. Animals were housed 2 or 3 per ventilated cage with corncob bedding, nesting material, and free access to food and water. Rooms were maintained at 21–23 °C, 40–60% humidity, with a 12 h light/dark cycle.

#### Oral Dosing Regimens: 10 mg/kg/day Study

In the initial efficacy study, mice were treated with compound **23** (10 mg/kg/day, oral gavage) or vehicle once daily for 28 days. The washout arm was treated at the same time as the initial cohort, with subsets of animals sacrificed at day 29 and others maintained for an additional 4 weeks post treatment. Group sizes were: day 29—vehicle (*n* = 6), compound 23 (*n* = 9); 4 week washout—vehicle (*n* = 6), compound **23** (*n* = 8).

#### Dose Response Studies (10, 3, 1, 0.3 mg/kg/day)

A high-range dose–response study was conducted with daily oral gavage for 28 days. Group sizes were vehicle (*n* = 9), 10 mg/kg (*n* = 3), 3 mg/kg (*n* = 9), 1 mg/kg (*n* = 9), and 0.3 mg/kg (*n* = 9).

#### Dose–response Studies (1, 0.3, 0.1, 0.03 mg/kg/day)

A low-range dose–response study was conducted with daily oral dosing for 28 days. Group sizes were vehicle (*n* = 9), 1 mg/kg (*n* = 9), 0.3 mg/kg (*n* = 9), 0.1 mg/kg (*n* = 9), and 0.03 mg/kg (*n* = 9). An additional 4 week washout cohort was included for the 1 mg/kg group (*n* = 9) and the vehicle group (*n* = 9).

#### Reversibility Study at 1 mg/kg/day

Reversibility was further evaluated using washout animals from the 1, 0.3, 0.1, and 0.03 mg/kg/day study. Mice treated with 1 mg/kg/day (*n* = 9) and vehicle (*n* = 9) for 28 days were monitored for recovery post-treatment.

At 4 weeks post-treatment: 1 mg/kg (*n* = 3), vehicle (*n* = 3).
At 8 weeks post-treatment: 1 mg/kg (*n* = 6), vehicle (*n* = 6).

### Tissue Collection

At designated end points (day 29, 4 weeks post-treatment, or 8 weeks post-treatment), mice were euthanized via CO_2_ asphyxiation followed by cervical dislocation. Testes and epididymides were dissected; testes were weighed immediately after dissection.

### Sperm Collection and Fixation

Sperm was collected from the cauda epididymis using a swim-out method. 1.0 mL of prewarmed Witten’s medium (37 °C) was placed into sterile tubes. Epididymides were dissected and transferred into the medium. Each cauda was finely minced with microscissors, and the tissue was incubated at 37 °C for 30 min to allow sperm to swim out. After incubation, 100 μL of sperm suspension was transferred to a separate tube containing 900 μL of 10% neutral buffered formalin (final concentration 1%) to fix the sperm for counting. Fixed samples were stored at 4 °C until analysis.

### Sperm Count Analysis

Sperm suspensions were diluted as appropriate and loaded into a Neubauer hemocytometer. Sperm was counted in five large squares per chamber under phase-contrast microscopy. Concentrations (sperm/mL) were calculated as.

Sperm concentration = mean count per square x 10^4^ x dilution factor.
The total sperm count per cauda was obtained by multiplying the sperm concentration by the incubation volume.

### Data Analysis

Testis weight, sperm counts, and body weights were expressed as mean ± SD. Comparisons between multiple dose groups were analyzed using one-way ANOVA with Tukey’s post hoc test. Reversibility cohorts were analyzed using a one-way ANOVA across time points. A *p*-value <0.05 was considered statistically significant.

### Testis Spatial Distribution of Compound **23** Using MALDI Imaging Mass Spectrometry (MALDI-IMS)

#### Materials

Indium tin oxide (ITO)-coated glass slides (25 × 75 × 1.1 mm, 70–100 ohms) were obtained from Delta Technologies (Loveland, CO). Water, acetonitrile, and eosin were obtained from Fisher Scientific (Waltham, MA). Hematoxylin, 1,5-diaminonaphthalene (DAN), and Entellan were obtained from Sigma-Aldrich (St. Louis, MO).

#### Cryo-Sectioning of Tissues and Matrix Deposition

The tissues were attached to a Leica CM3050 S cryostat (Leica Biosystems, Deer Park, IL) with water, then sectioned at 14 μm thickness at −20 °C. The testis samples were sectioned in both transverse and longitudinal orientations to obtain the maximum surface area for MALDI-IMS analysis. Sections from the control, 8 h, and 16 h groups of the same orientation were attached to the same ITO slide to avoid the effects of interslide variation.

The slides were removed from the −80 °C freezer and promptly placed in a desiccator for 20 min to reduce the condensation of atmospheric water on their surfaces. Tissue sections were coated with a matrix solution containing 10 mg/mL DAN in 70% aqueous acetonitrile using an HTX M5 Sprayer (HTX Technologies LLC, Carrboro, NC). The spraying conditions were as follows: nitrogen gas pressure, 10 psi; nozzle temperature, 65 °C; tray temperature, 40 °C; pass count, 4; spray pattern, CC; pump flow rate, 0.1 mL/min; nozzle velocity, 1250 mm/min; track spacing, 2.5 mm; drying time, 10 s.

#### MALDI-IMS Analysis

The testis distribution of **23** was visualized using a combination of a Q-Exactive Hybrid Quadrupole-Orbitrap mass spectrometer (Thermo Fisher Scientific, Waltham, MA) and an elevated-pressure MALDI ion source with a dual-ion funnel interface (Spectroglyph LLC, Richland, WA). An attached Q-switched frequency-tripled Nd: YLF laser (349 nm wavelength) was used at a repetition rate of 500 Hz and a pulse energy of 1.2 to 1.3 μJ. The high-pressure ion funnel was maintained at 7.4 to 7.5 Torr, and the low-pressure ion funnel was maintained at 1.6 to 1.8 Torr. Spectroglyph MALDI injector 1.3 software was used to acquire the images at a spatial resolution of 40 μm. The mass spectrometer was operated in negative-ion mode over the mass range *m*/*z* 100 to 1000, with a mass resolution of 70,000.

After IMS data acquisition, the slides were stained using a standard hematoxylin and eosin (H&E) protocol. The residual DAN matrix was removed by submerging in 70% ethanol (3 min). Then for 3 min, sections were rinsed in successive ethanol washes (100%, 96%, 96%, 70%, 70%) and deionized H_2_O. Hematoxylin staining was applied for 3 min, followed by a gentle 3 min wash with running tap water. Eosin staining was applied for 30 s and rinsed gently with running tap water for 3 min. The staining was completed with an ethanol wash for 1 min and a xylene wash for 30 s. Optical images were captured with a Zeiss Axioscan 7 microscopy scanner (Oberkochen, Germany) at 20× magnification (0.173 μm/pixel).

SCiLS Laboratory software version 2025b (SCiLS GmbH, Bremen, Germany) was used for processing IMS data, visualization, and coregistration of H&E-stained optical images. Mass spectral data were imported into the SCiLS software, followed by baseline correction (convolution algorithm) and root-mean-square normalization. Feature images were generated for **23** ([M-H]^−^) and lipid ion species [PE (36:2)] within a mass error of ±10 ppm.

### General Chemistry

All chemicals and reagents were obtained from commercial sources and used as received unless otherwise specified. Reactions were carried out in anhydrous solvents, which were degassed with nitrogen and passed through activated alumina or molecular sieves. Reaction progress was monitored by thin-layer chromatography (TLC) on silica gel 60 F_254_ plates, visualized under UV light at 254 and 365 nm. Compounds were purified by flash column chromatography. The purity of final compounds was determined by HPLC/MS (solvent system from Fisher Scientific International, Inc.), and mass spectrometric analysis was performed on an Agilent InfinityLab LC/MSD iQ instrument in positive-ion mode. The purity of all tested compounds was >95%, except for compound **14**, which had a purity of 92%. Microwave-assisted reactions were conducted using an Initiator+ (Biotage, San Jose, CA) with Biotage microwave reaction vials. All NMR spectroscopy experiments were performed on a Bruker Avance II 400/100 MHz instrument equipped with a BBO broadband probe, and spectra were processed by using MestReNova software (Mestrelab Research S.L.). Chemical shifts are reported in ppm and referenced to residual solvent peaks: CHCl_3_ in CDCl_3_ at 7.26 ppm for ^1^H NMR and 77.16 ppm for ^13^C NMR; DMSO in DMSO-*d*_6_ at 2.50 ppm for ^1^H NMR and 39.52 ppm for ^13^C NMR. Coupling constants (*J*) are given in hertz (Hz), and splitting patterns are designated as singlet (s), doublet (d), triplet (t), quartet (q), and broad singlet (br s).

### Synthesis of Benzopyran Analogs

#### 3-Bromo-2-hydroxy-6-methylbenzaldehyde (**52**)

2-Bromo-5-methylphenol (**51**) (5.0 g, 27 mmol) was dissolved in MeCN (100 mL) at room temperature under nitrogen, and then magnesium chloride (7.6 g, 80 mmol) was added in one portion, followed by triethylamine (9.3 mL, 67 mmol). The mixture was stirred at room temperature for 20 min before paraformaldehyde (4.0 g, 130 mmol) was added in one portion. The mixture was then heated to 80 °C and stirred overnight. The mixture was cooled to room temperature, then quenched with aqueous HCl (1N, 80 mL), and extracted with ethyl acetate. The organic layer was dried over anhydrous MgSO_4_, then purified by flash chromatography using mixtures of hexanes, and ethyl acetate to afford 3-bromo-2-hydroxy-6-methylbenzaldehyde (3.0 g, 53% yield) as a pale-yellow solid. ^1^H NMR (400 MHz, DMSO-*d*_6_) δ: 12.36 (s, 1H), 10.27 (s, 1H), 7.76 (d, *J* = 8.1 Hz, 1H), 6.82–6.77 (m, 1H), 2.57 (s, 3H).

#### 8-Bromo-5-methyl-2*H*-chromene-3-carbaldehyde (**53**)

3-Bromo-2-hydroxy-6-methylbenzaldehyde (**52**) (2.0 g, 9.3 mmol) was dissolved in 1,4-dioxane (100 mL) at room temperature. K_2_CO_3_ (1.5 g, 11 mmol) was added portion-wise, followed by acrylaldehyde (0.78 mL, 11 mmol). The mixture was then heated to 100 °C and stirred for 4 h. The mixture was cooled to room temperature, then quenched with water (70 mL), and extracted with ethyl acetate. The organic layer was dried over anhydrous MgSO_4_, then purified by flash chromatography using mixtures of hexanes and ethyl acetate to afford 8-bromo-5-methyl-2*H*-chromene-3-carbaldehyde (2.0 g, 83% yield) as a pale-yellow solid. ^1^H NMR (400 MHz, DMSO-*d*_6_) δ: 12.36 (s, 1H), 10.27 (s, 1H), 7.76 (d, *J* = 8.1 Hz, 1H), 6.87–6.77 (m, 1H), 2.57 (s, 3H).

#### Methyl 4-(4-(8-bromo-5-methyl-2*H*-chromen-3-yl)-4-oxobutanoyl)­benzoate (**54**)

8-Bromo-5-methyl-2*H*-chromene-3-carbaldehyde (**53**) (1.0 g, 4.0 mmol), methyl 4-acryloylbenzoate (0.90 g, 4.7 mmol), 3-benzyl-5-(2-hydroxyethyl)-4-methylthiazol-3-ium chloride (0.53 g, 2.0 mmol), and triethylamine (0.66 mL, 4.7 mmol) were dissolved in *N,N*-dimethylformamide (10 mL). The mixture was then heated to 100 °C and stirred for 30 min. The mixture was cooled to room temperature, then quenched with water (30 mL), and extracted with ethyl acetate. The organic layer was washed with water and brine, and then dried over anhydrous MgSO_4_, purified by flash chromatography using mixtures of hexanes and ethyl acetate to afford methyl 4-(4-(8-bromo-5-methyl-2*H*-chromen-3-yl)-4-oxobutanoyl)­benzoate (0.79 g, 45% yield) as a white solid. ^1^H NMR (400 MHz, CDCl_3_) δ: 8.11–7.96 (m, 4H), 7.56 (d, *J* = 1.4 Hz, 1H), 7.30 (d, *J* = 8.2 Hz, 1H), 6.63 (dd, *J* = 8.2, 0.8 Hz, 1H), 5.00 (d, *J* = 1.3 Hz, 2H), 3.89 (s, 3H), 3.35 (dd, *J* = 6.8, 5.7 Hz, 2H), 3.21 (t, *J* = 6.3 Hz, 2H), 2.33 (s, 3H).

#### Methyl 4-(5-(8-bromo-5-methyl-2*H*-chromen-3-yl)-1*H*-pyrrol-2-yl)­benzoate (**55**)

Methyl 4-(4-(8-bromo-5-methyl-2*H*-chromen-3-yl)-4-oxobutanoyl)­benzoate (**54**) (510 mg, 1.2 mmol), ammonium acetate (880 mg, 12 mmol), and acetic acid (0.66 mL, 12 mmol) were dissolved in MeOH (3 mL). After stirring at reflux for 3 h, the reaction mixture was cooled to room temperature and quenched with water, extracted with EtOAc, washed with brine, dried over anhydrous MgSO_4_, and concentrated. The resulting crude was purified by flash chromatography using mixtures of hexanes and ethyl acetate to afford methyl 4-(5-(8-bromo-5-methyl-2*H*-chromen-3-yl)-1*H*-pyrrol-2-yl)­benzoate (0.46 g, 95% yield) as a yellow solid. ^1^H NMR (400 MHz, CDCl_3_) δ: 8.69 (s, 1H), 8.09 (d, *J* = 8.3 Hz, 2H), 7.63 (d, *J* = 8.2 Hz, 2H), 7.24 (d, *J* = 8.1 Hz, 1H), 6.76–6.66 (m, 3H), 6.42 (t, *J* = 3.1 Hz, 1H), 5.13 (s, 2H), 3.96 (s, 3H), 2.40 (s, 3H).

#### 4-(5-(5-Methyl-8-(*p*-tolyl)-2*H*-chromen-3-yl)-1*H*-pyrrol-2-yl)­benzoic Acid (10)

Methyl 4-(5-(8-bromo-5-methyl-2*H*-chromen-3-yl)-1*H*-pyrrol-2-yl)­benzoate (**55**) (150 mg, 0.35 mmol), *p*-tolylboronic acid (53 mg, 0.39 mmol), Pd­(Ph_3_P)_4_ (41 mg, 0.035 mmol), K_2_CO_3_ (98 mg, 0.71 mmol) were dissolved in THF/water (10/1, 5 mL). This mixture was stirred at 70 °C for 8 h under nitrogen. After completion of the reaction, lithium hydroxide monohydrate (150 mg, 3.5 mmol) was added, and the mixture was stirred for an additional 18 h at 40 °C. The resulting mixture was cooled to room temperature and acidified with HCl (1M) to pH 1, followed by extraction with ethyl acetate. The organic layer was washed with brine and dried over anhydrous MgSO_4_, then purified by flash chromatography using mixtures of hexanes and ethyl acetate to afford 4-(5-(5-methyl-8-(*p*-tolyl)-2*H*-chromen-3-yl)-1*H*-pyrrol-2-yl)­benzoic acid (80 mg, 54% yield) as a yellow solid. ^1^H NMR (400 MHz, DMSO-*d*_6_) δ: 12.85 (s, 1H), 11.45 (t, *J* = 2.5 Hz, 1H), 8.01–7.93 (m, 2H), 7.90 (d, *J* = 8.6 Hz, 2H), 7.44–7.37 (m, 2H), 7.34 (d, *J* = 1.2 Hz, 1H), 7.22 (d, *J* = 7.9 Hz, 2H), 7.04 (d, *J* = 7.8 Hz, 1H), 6.88 (d, *J* = 7.9 Hz, 1H), 6.79 (dd, *J* = 3.8, 2.3 Hz, 1H), 6.50 (dd, *J* = 3.8, 2.3 Hz, 1H), 4.95 (d, *J* = 1.1 Hz, 2H), 2.44 (s, 3H), 2.34 (s, 3H). ^13^C NMR (101 MHz, DMSO-*d*_6_) δ: 167.0, 149.5, 136.0, 135.8, 134.7, 133.1, 133.0, 131.0, 129.8, 128.9, 128.5, 128.0, 127.8, 126.2, 124.1, 123.8, 123.2, 122.7, 111.9, 110.3, 109.4, 64.5, 20.7, 18.5. *m*/*z* (ES^+^) [M + H]^+^ = 422.2.

#### General Synthetic Method for **57a**–**57e**

(1-(*Tert*-butoxycarbonyl)-1*H*-pyrrol-2-yl)­boronic acid **56** (1.0 equiv), methyl 4-bromobenzoates (1.0 equiv), Pd­(Ph_3_P)_4_ (0.05 equiv), K_2_CO_3_ (2.0 equiv) were dissolved in 1,4-dioxane/water (10/1, 0.2 M). This mixture was stirred at 100 °C for 4 h under nitrogen. The organic solvent was removed under reduced pressure. The residue was partitioned between ethyl ether and water. The resulting organic layer was dried over anhydrous MgSO_4_, then purified by flash chromatography using mixtures of hexanes and ethyl acetate to afford *tert*-butyl 2-(4-(methoxycarbonyl)­phenyl)-1*H*-pyrrole-1-carboxylates **57a**-**57e**.

#### *Tert*-butyl 2-(4-(Methoxycarbonyl)­phenyl)-1*H*-pyrrole-1-carboxylate (**57a**)

Oil (96% yield). ^1^H NMR (400 MHz, CDCl_3_) δ: 8.05–8.00 (m, 2H), 7.44–7.39 (m, 2H), 7.38 (dd, *J* = 3.2, 1.9 Hz, 1H), 6.28–6.22 (m, 2H), 3.93 (s, 3H), 1.37 (s, 9H).

#### *Tert*-butyl 2-(3-Fluoro-4-(methoxycarbonyl)­phenyl)-1*H*-pyrrole-1-carboxylate (**57b**)

Oil (90% yield). ^1^H NMR (400 MHz, CDCl_3_) δ: 7.92 (t, *J* = 7.9 Hz, 1H), 7.39 (dd, *J* = 3.3, 1.8 Hz, 1H), 7.21–7.11 (m, 2H), 6.29 (dd, *J* = 3.3, 1.8 Hz, 1H), 6.25 (t, *J* = 3.3 Hz, 1H), 3.94 (s, 3H), 1.41 (s, 9H).

#### *Tert*-butyl 2-(3,5-Difluoro-4-(methoxycarbonyl)­phenyl)-1*H*-pyrrole-1-carboxylate (**57c**)

Oil (97% yield). ^1^H NMR (400 MHz, CDCl_3_) δ: 7.38 (dd, *J* = 3.2, 1.7 Hz, 1H), 6.99–6.91 (m, 2H), 6.29 (dd, *J* = 3.4, 1.8 Hz, 1H), 6.24 (t, *J* = 3.3 Hz, 1H), 3.95 (s, 3H), 1.45 (s, 9H).

#### *Tert*-butyl 2-(3-Chloro-4-(methoxycarbonyl)­phenyl)-1*H*-pyrrole-1-carboxylate (**57d**)

Oil (85% yield). ^1^H NMR (400 MHz, CDCl_3_) δ: 7.85 (d, *J* = 8.0 Hz, 1H), 7.45 (d, *J* = 1.6 Hz, 1H), 7.39 (dd, *J* = 3.3, 1.8 Hz, 1H), 7.30 (dd, *J* = 8.1, 1.7 Hz, 1H), 6.28 (dd, *J* = 3.4, 1.7 Hz, 1H), 6.25 (t, *J* = 3.3 Hz, 1H), 3.94 (s, 3H), 1.40 (s, 9H).

#### *Tert*-butyl 2-(3-Hydroxy-4-(methoxycarbonyl)­phenyl)-1*H*-pyrrole-1-carboxylate (**57e**)

Oil (81% yield). ^1^H NMR (400 MHz, CDCl_3_) δ: 10.75 (s, 1H), 7.79 (d, *J* = 8.3 Hz, 1H), 7.36 (dd, *J* = 3.4, 1.7 Hz, 1H), 6.97 (d, *J* = 1.6 Hz, 1H), 6.87 (dd, *J* = 8.2, 1.7 Hz, 1H), 6.28 (dd, *J* = 3.3, 1.8 Hz, 1H), 6.23 (t, *J* = 3.3 Hz, 1H), 3.96 (s, 3H), 1.41 (s, 9H).

#### General Synthetic Method for **58a**–**58e**

To a solution of *tert*-butyl 2-(4-(methoxycarbonyl)­phenyl)-1*H*-pyrrole-1-carboxylates **57a**-**57e** (1.0 equiv) in THF (0.2 M) was added sodium methoxide solution (25 wt %, 1.5 equiv) and kept stirring at room temperature for 30 min. The mixture was then quenched with water and extracted with an EtOAc. The organic layer was washed with brine, dried with anhydrous MgSO_4_, and concentrated. The resulting crude was purified by flash chromatography using mixtures of hexanes and ethyl acetate to afford methyl 4-(1*H*-pyrrol-2-yl)­benzoates **58a**-**58e**.

#### Methyl 4-(1*H*-Pyrrol-2-yl)­benzoate (**58a**)

White solid (87% yield). ^1^H NMR (400 MHz, CDCl_3_) δ: 8.55 (s, 1H), 8.06–7.98 (m, 2H), 7.57–7.44 (m, 2H), 6.93 (td, *J* = 2.7, 1.4 Hz, 1H), 6.66 (ddd, *J* = 3.9, 2.7, 1.4 Hz, 1H), 6.33 (dt, *J* = 3.5, 2.5 Hz, 1H), 3.92 (s, 3H).

#### Methyl 2-Fluoro-4-(1*H*-pyrrol-2-yl)­benzoate (**58b**)

White solid (53% yield). ^1^H NMR (400 MHz, CDCl_3_) δ: 8.54 (s, 1H), 7.94 (t, *J* = 8.0 Hz, 1H), 7.28 (dd, *J* = 8.2, 1.7 Hz, 1H), 7.20 (dd, *J* = 12.3, 1.8 Hz, 1H), 6.95 (td, *J* = 2.7, 1.4 Hz, 1H), 6.71–6.65 (m, 1H), 6.34 (q, *J* = 2.8 Hz, 1H), 3.93 (s, 3H).

#### Methyl 2,6-Difluoro-4-(1*H*-pyrrol-2-yl)­benzoate (**58c**)

White solid (49% yield). ^1^H NMR (400 MHz, CDCl_3_) δ: 8.54 (s, 1H), 7.06–6.98 (m, 2H), 6.94 (td, *J* = 2.8, 1.4 Hz, 1H), 6.65 (ddd, *J* = 3.9, 2.7, 1.4 Hz, 1H), 6.33 (dt, *J* = 3.6, 2.6 Hz, 1H), 3.94 (s, 3H).

#### Methyl 2-Chloro-4-(1*H*-pyrrol-2-yl)­benzoate (**58d**)

White solid (80% yield). ^1^H NMR (400 MHz, CDCl_3_) δ: 8.51 (s, 1H), 7.89 (d, *J* = 8.2 Hz, 1H), 7.53 (d, *J* = 1.8 Hz, 1H), 7.38 (dd, *J* = 8.2, 1.8 Hz, 1H), 6.94 (td, *J* = 2.7, 1.4 Hz, 1H), 6.66 (ddd, *J* = 3.9, 2.7, 1.4 Hz, 1H), 6.33 (dt, *J* = 3.7, 2.6 Hz, 1H), 3.93 (s, 3H).

#### Methyl 2-Hydroxy-4-(1*H*-pyrrol-2-yl)­benzoate (**58e**)

White solid (71% yield). ^1^H NMR (400 MHz, CDCl_3_) δ: 10.84 (s, 1H), 8.53 (s, 1H), 7.80 (d, *J* = 8.2 Hz, 1H), 7.04–6.98 (m, 2H), 6.92 (td, *J* = 2.7, 1.4 Hz, 1H), 6.66 (dt, *J* = 3.6, 2.6 Hz, 1H), 6.32 (dt, *J* = 3.6, 2.6 Hz, 1H), 3.95 (s, 3H).

#### General Synthetic Method for **59a**–**59e**

Methyl 4-(1*H*-pyrrol-2-yl)­benzoates **58a**–**58e** (1.0 equiv), 4,4,4′,4′,5,5,5′,5′-octamethyl-2,2′-bi­(1,3,2-dioxaborolane) (0.55 equiv), [Ir­(OMe)­(COD)]_2_ (0.015 equiv), 4,4′-di-*tert-*butyl-2,2′-bipyridine (0.03 equiv) were dissolved in dry THF (0.2 M) and then stirred for 8 h at room temperature. The solvent was removed under reduced pressure, and the residue was partitioned between ethyl acetate and water. The resulting organic layer was dried over anhydrous MgSO_4_ and then purified by flash chromatography using mixtures of hexanes and ethyl acetate to afford methyl 4-(5-(4,4,5,5-tetramethyl-1,3,2-dioxaborolan-2-yl)-1*H*-pyrrol-2-yl)­benzoates **59a**–**59e**.

#### Methyl 4-(5-(4,4,5,5-Tetramethyl-1,3,2-dioxaborolan-2-yl)-1*H*-pyrrol-2-yl)­benzoate (**59a**)

White solid (79% yield). ^1^H NMR (400 MHz, CDCl_3_) δ: 8.96 (s, 1H), 8.08–7.98 (m, 2H), 7.65–7.56 (m, 2H), 6.89 (dd, *J* = 3.7, 2.4 Hz, 1H), 6.69 (dd, *J* = 3.7, 2.5 Hz, 1H), 3.92 (s, 3H), 1.35 (s, 12H).

#### Methyl 2-Fluoro-4-(5-(4,4,5,5-tetramethyl-1,3,2-dioxaborolan-2-yl)-1*H*-pyrrol-2-yl)­benzoate (**59b**)

White solid (76% yield). ^1^H NMR (400 MHz, CDCl_3_) δ: 8.96 (s, 1H), 7.94 (td, *J* = 7.9, 2.6 Hz, 1H), 7.39–7.26 (m, 2H), 6.88 (dd, *J* = 3.7, 2.4 Hz, 1H), 6.69 (dd, *J* = 3.7, 2.5 Hz, 1H), 3.93 (s, 3H), 1.34 (s, 12H).

#### Methyl 2,6-Difluoro-4-(5-(4,4,5,5-tetramethyl-1,3,2-dioxaborolan-2-yl)-1*H*-pyrrol-2-yl)­benzoate (**59c**)

White solid (85% yield). ^1^H NMR (400 MHz, CDCl_3_) δ: 8.90 (s, 1H), 7.13–7.06 (m, 2H), 6.87 (dd, *J* = 3.8, 2.3 Hz, 1H), 6.66 (dd, *J* = 3.7, 2.5 Hz, 1H), 3.95 (s, 3H), 1.34 (s, 12H).

#### Methyl 2-Chloro-4-(5-(4,4,5,5-tetramethyl-1,3,2-dioxaborolan-2-yl)-1*H*-pyrrol-2-yl)­benzoate (**59d**)

White solid (78% yield). ^1^H NMR (400 MHz, CDCl_3_) δ: 8.93 (s, 1H), 7.89 (d, *J* = 8.2 Hz, 1H), 7.60 (d, *J* = 1.7 Hz, 1H), 7.45 (dd, *J* = 8.2, 1.8 Hz, 1H), 6.88 (dd, *J* = 3.7, 2.4 Hz, 1H), 6.68 (dd, *J* = 3.7, 2.6 Hz, 1H), 3.93 (s, 3H), 1.35 (s, 12H).

#### Methyl 2-Hydroxy-4-(5-(4,4,5,5-tetramethyl-1,3,2-dioxaborolan-2-yl)-1*H*-pyrrol-2-yl)­benzoate (**59e**)

White solid (64% yield). ^1^H NMR (400 MHz, CDCl_3_) δ: 10.81 (s, 1H), 8.96 (s, 1H), 7.82 (d, *J* = 8.3 Hz, 1H), 7.12 (d, *J* = 1.8 Hz, 1H), 7.07 (dd, *J* = 8.3, 1.8 Hz, 1H), 6.87 (dd, *J* = 3.7, 2.4 Hz, 1H), 6.69 (dd, *J* = 3.7, 2.5 Hz, 1H), 3.95 (s, 3H), 1.34 (s, 12H).

#### General Synthetic Method for **61a**–**61g**

5-Bromo-2-methylphenol derivatives **60a**–**60e** (1.0 equiv), prop-2-yn-1-ol derivatives (1.2 equiv), and triphenylphosphine (1.1 equiv) were dissolved in THF (0.2 M). The solution was cooled to 0 °C, and diethyl azodicarboxylate (1.2 equiv) was added dropwise via syringe. The ice bath was removed, and the mixture was stirred under argon for 18 h. The mixture was then quenched with water and extracted with EtOAc. The organic layer was washed with brine, dried over anhydrous MgSO_4_, and concentrated. The resulting crude was purified by flash chromatography using mixtures of hexanes and ethyl acetate to afford **61a**–**61g**.

#### 4-Bromo-1-methyl-2-(prop-2-yn-1-yloxy)­benzene (**61a**)

White solid (81% yield). ^1^H NMR (400 MHz, CDCl_3_) δ: 7.09–6.99 (m, 3H), 4.70 (d, *J* = 2.4 Hz, 2H), 2.54 (t, *J* = 2.4 Hz, 1H), 2.18 (s, 3H).

#### 4-Bromo-1-isopropyl-2-(prop-2-yn-1-yloxy)­benzene (**61b**)

White solid (83% yield). ^1^H NMR (400 MHz, CDCl_3_) δ: 7.24 (dd, *J* = 7.4, 2.3 Hz, 1H), 7.08 (ddd, *J* = 8.6, 4.0, 2.3 Hz, 1H), 6.98 (dd, *J* = 10.8, 8.7 Hz, 1H), 4.76 (d, *J* = 2.4 Hz, 2H), 2.58 (t, *J* = 2.4 Hz, 1H).

#### 4-Bromo-1-fluoro-2-(prop-2-yn-1-yloxy)­benzene (**61c**)

Oil (89% yield). ^1^H NMR (400 MHz, CDCl_3_) δ: 7.24 (dd, *J* = 7.4, 2.3 Hz, 1H), 7.08 (ddd, *J* = 8.6, 4.0, 2.3 Hz, 1H), 6.98 (dd, *J* = 10.8, 8.7 Hz, 1H), 4.76 (d, *J* = 2.4 Hz, 2H), 2.58 (t, *J* = 2.4 Hz, 1H).

#### 4-Bromo-1-ethyl-2-(prop-2-yn-1-yloxy)­benzene (**61d**)

Oil (90% yield). ^1^H NMR (400 MHz, CDCl_3_) δ: 7.09–7.05 (m, 2H), 7.02 (d, *J* = 8.5 Hz, 1H), 4.70 (d, *J* = 2.4 Hz, 2H), 2.60 (q, *J* = 7.5 Hz, 2H), 2.53 (t, *J* = 2.4 Hz, 1H), 1.17 (t, *J* = 7.5 Hz, 3H).

#### 4-Bromo-2-(prop-2-yn-1-yloxy)-1-(trifluoromethyl)­benzene (**61e**)

Oil (86% yield). ^1^H NMR (400 MHz, CDCl_3_) δ: 7.45 (d, *J* = 8.3 Hz, 1H), 7.32 (t, *J* = 1.2 Hz, 1H), 7.22 (ddd, *J* = 8.3, 1.8, 0.9 Hz, 1H), 4.80 (d, *J* = 2.4 Hz, 2H), 2.59 (t, *J* = 2.4 Hz, 1H).

#### (*S*)-4-Bromo-2-(but-3-yn-2-yloxy)-1-methylbenzene (**61f**)

Oil (81% yield). ^1^H NMR (400 MHz, CDCl_3_) δ: 7.16 (d, *J* = 1.7 Hz, 1H), 7.04–6.98 (m, 2H), 4.81 (qd, *J* = 6.6, 2.0 Hz, 1H), 2.50 (d, *J* = 2.0 Hz, 1H), 2.17 (s, 3H), 1.68 (d, *J* = 6.6 Hz, 3H).

#### (*S*)-4-Bromo-2-(but-3-yn-2-yloxy)-1-fluorobenzene (**61g**)

Oil (88% yield). ^1^H NMR (400 MHz, CDCl_3_) δ: 7.29 (dd, *J* = 7.4, 2.4 Hz, 1H), 7.08 (ddd, *J* = 8.8, 4.0, 2.4 Hz, 1H), 6.97 (dd, *J* = 10.7, 8.7 Hz, 1H), 4.88 (qd, *J* = 6.6, 2.1 Hz, 1H), 2.56–2.52 (m, 1H), 1.70 (dd, *J* = 6.6, 0.8 Hz, 3H).

### General Synthetic Method for **62a**–**62g**

4-Bromo-2-(prop-2-yn-1-yloxy)-1-methylbenzene derivatives **61a**–**61g** (1.0 equiv), NIS (1.15 equiv), and silver nitrate (0.1 equiv) were suspended in acetone for 3 h. The mixture was filtered, and the filtrate was concentrated under reduced pressure and then purified by flash chromatography using mixtures of hexanes and ethyl acetate to afford **62a**–**62g**.

#### 4-Bromo-2-((3-iodoprop-2-yn-1-yl)­oxy)-1-methylbenzene (**62a**)

White solid (90% yield). ^1^H NMR (400 MHz, CDCl_3_) δ: 7.02 (d, *J* = 7.5 Hz, 3H), 4.83 (s, 2H), 2.17 (s, 3H).

#### 4-Bromo-2-((3-iodoprop-2-yn-1-yl)­oxy)-1-isopropylbenzene (**62b**)

White solid (84% yield). ^1^H NMR (400 MHz, CDCl_3_) δ: 7.14–7.01 (m, 3H), 4.83 (s, 2H), 3.27 (hept, *J* = 7.0 Hz, 1H), 1.18 (d, *J* = 6.9 Hz, 6H).

#### 4-Bromo-1-fluoro-2-((3-iodoprop-2-yn-1-yl)­oxy)­benzene (**62c**)

White solid (89% yield). ^1^H NMR (400 MHz, CDCl_3_) δ: 7.20 (dd, *J* = 7.4, 2.3 Hz, 1H), 7.09 (ddd, *J* = 8.6, 4.0, 2.3 Hz, 1H), 6.97 (dd, *J* = 10.8, 8.6 Hz, 1H), 4.88 (s, 2H).

#### 4-Bromo-1-ethyl-2-((3-iodoprop-2-yn-1-yl)­oxy)­benzene (**62d**)

Oil (82% yield). ^1^H NMR (400 MHz, CDCl_3_) δ: 7.11–6.98 (m, 3H), 4.83 (s, 2H), 2.59 (q, *J* = 7.5 Hz, 2H), 1.16 (t, *J* = 7.5 Hz, 3H).

#### 4-Bromo-2-((3-iodoprop-2-yn-1-yl)­oxy)-1-(trifluoromethyl)­benzene (**62e**)

Oil (87% yield). ^1^H NMR (400 MHz, CDCl_3_) δ: 7.44 (dd, *J* = 8.3, 0.7 Hz, 1H), 7.29 (dd, *J* = 1.7, 0.8 Hz, 1H), 7.22 (ddt, *J* = 8.4, 1.6, 0.8 Hz, 1H), 4.93 (s, 2H).

#### (*S*)-4-Bromo-2-((4-iodobut-3-yn-2-yl)­oxy)-1-methylbenzene (**62f**)

White solid (95% yield). ^1^H NMR (400 MHz, CDCl_3_) δ: 7.12 (d, *J* = 1.7 Hz, 1H), 7.06–6.97 (m, 2H), 4.90 (q, *J* = 6.5 Hz, 1H), 2.16 (s, 3H), 1.66 (d, *J* = 6.6 Hz, 3H).

#### (*S*)-4-Bromo-1-fluoro-2-((4-iodobut-3-yn-2-yl)­oxy)­benzene (**62g**)

White solid (85% yield). ^1^H NMR (400 MHz, CDCl_3_) δ: 7.26 (dd, *J* = 7.4, 2.2 Hz, 1H), 7.08 (ddd, *J* = 8.7, 4.1, 2.3 Hz, 1H), 6.96 (dd, *J* = 10.8, 8.7 Hz, 1H), 4.97 (q, *J* = 6.5 Hz, 1H), 1.68 (d, *J* = 6.6 Hz, 3H).

### General Synthetic Method for **63a**–**63g**

4-Bromo-2-((3-iodoprop-2-yn-1-yl)­oxy)-1-methylbenzene derivatives **62a**–**62g** (1.0 equiv) and IPrAuNTf_2_ (0.05 equiv) were dissolved in 1,4-dioxane. The resulting solution was stirred at 100 °C for 5 h under nitrogen. After the reaction was finished, the solvent was removed under reduced pressure, and the crude product was purified by flash chromatography using hexanes as eluent to afford **63a**–**63g**.

#### 5-Bromo-3-iodo-8-methyl-2*H*-chromene (**63a**)

Oil (53% yield). ^1^H NMR (400 MHz, CDCl_3_) δ: 7.35 (t, *J* = 1.6 Hz, 1H), 7.01 (d, *J* = 8.1 Hz, 1H), 6.86 (d, *J* = 8.1 Hz, 1H), 4.86 (d, *J* = 1.7 Hz, 2H), 2.11 (s, 3H).

#### 5-Bromo-3-iodo-8-isopropyl-2*H*-chromene (**63b**)

Oil (39% yield). ^1^H NMR (400 MHz, CDCl_3_) δ: 7.37 (t, *J* = 1.6 Hz, 1H), 7.08 (d, *J* = 8.4 Hz, 1H), 6.95 (d, *J* = 8.4 Hz, 1H), 4.83 (d, *J* = 1.6 Hz, 2H), 3.15 (hept, *J* = 6.9 Hz, 1H), 1.17 (d, *J* = 6.9 Hz, 6H).

#### 5-Bromo-8-fluoro-3-iodo-2*H*-chromene (**63c**)

Oil (51% yield). ^1^H NMR (400 MHz, CDCl_3_) δ: 7.34 (q, *J* = 1.7 Hz, 1H), 7.04 (dd, *J* = 8.8, 4.4 Hz, 1H), 6.86 (dd, *J* = 10.0, 8.8 Hz, 1H), 4.91 (d, *J* = 1.8 Hz, 2H).

#### 5-Bromo-8-ethyl-3-iodo-2*H*-chromene (**63d**)

Oil (44% yield). ^1^H NMR (400 MHz, CDCl_3_) δ: 7.36 (t, *J* = 1.6 Hz, 1H), 7.05 (d, *J* = 8.2 Hz, 1H), 6.89 (d, *J* = 8.2 Hz, 1H), 4.84 (d, *J* = 1.6 Hz, 2H), 2.52 (q, *J* = 7.5 Hz, 2H), 1.14 (t, *J* = 7.5 Hz, 3H).

#### 5-Bromo-3-iodo-8-(trifluoromethyl)-2*H*-chromene (**63e**)

Oil (60% yield). ^1^H NMR (400 MHz, CDCl_3_) δ: 7.39 (t, *J* = 1.7 Hz, 1H), 7.25 (d, *J* = 5.5 Hz, 1H), 7.19 (d, *J* = 8.5 Hz, 1H), 4.96 (d, *J* = 1.6 Hz, 2H).

#### (*S*)-5-Bromo-3-iodo-2,8-dimethyl-2*H*-chromene (**63f**)

Oil (30% yield). ^1^H NMR (400 MHz, CDCl_3_) δ: 7.29 (d, *J* = 0.6 Hz, 1H), 7.00 (d, *J* = 8.1 Hz, 1H), 6.87 (dd, *J* = 8.1, 0.8 Hz, 1H), 5.08–5.02 (m, 1H), 2.11 (d, *J* = 0.8 Hz, 3H), 1.43 (d, *J* = 6.5 Hz, 3H).

#### (*S*)-5-Bromo-8-fluoro-3-iodo-2-methyl-2*H*-chromene (**63g**)

Oil (59% yield). ^1^H NMR (400 MHz, CDCl_3_) δ: 7.29 (d, *J* = 1.6 Hz, 1H), 7.03 (dd, *J* = 8.8, 4.4 Hz, 1H), 6.87 (dd, *J* = 10.0, 8.9 Hz, 1H), 5.12 (q, *J* = 6.6 Hz, 1H), 1.50 (d, *J* = 6.6 Hz, 3H).

### General Synthetic Method for **9** and **14**–**29**

5-Bromo-3-iodo-2*H*-chromenes **63a**-**63g** (1.0 equiv), methyl 4-(5-(4,4,5,5-tetramethyl-1,3,2-dioxaborolan-2-yl)-1*H*-pyrrol-2-yl)­benzoates **59a**-**59e** (1.1 equiv), Pd­(PPh_3_)_4_ (0.1 equiv), K_2_CO_3_ (3.0 equiv) were dissolved in 1,4-dioxane/water (10/1, 10 mL). This mixture was stirred at 100 °C for 3 h under nitrogen. *p*-Tolylboronic acid (1.5 equiv) and Pd­(amphos)­Cl_2_ (0.1 equiv) were added and stirred at 100 °C for 8 h under nitrogen. After completion of the reaction, lithium hydroxide monohydrate (10 equiv) in water (1 mL) was added, and the mixture was stirred for an additional 18 h at 40 °C. The resulting mixture was cooled to room temperature and acidified with HCl (1M) to pH 1, then extracted with ethyl acetate. The organic layer was washed with brine, dried over anhydrous MgSO_4_, and purified by flash chromatography using mixtures of hexanes and ethyl acetate to afford the desired products.

#### 4-(5-(8-Methyl-5-(*p*-tolyl)-2*H*-chromen-3-yl)-1*H*-pyrrol-2-yl)­benzoic Acid (9)

Yellow solid (33% yield). ^1^H NMR (400 MHz, DMSO-*d*_6_) δ: 12.80 (s, 1H), 11.43 (s, 1H), 7.91 (d, *J* = 8.1 Hz, 2H), 7.82 (d, *J* = 8.3 Hz, 2H), 7.31 (d, *J* = 2.0 Hz, 4H), 7.06 (d, *J* = 7.7 Hz, 1H), 7.01 (s, 1H), 6.83 (d, *J* = 7.8 Hz, 1H), 6.79–6.73 (m, 1H), 6.47–6.41 (m, 1H), 5.03 (s, 2H), 2.39 (s, 3H), 2.21 (s, 3H). ^13^C NMR (101 MHz, DMSO-*d*_6_) δ: 167.0, 151.5, 136.7, 136.6, 136.3, 136.0, 132.9, 131.5, 129.7, 129.4, 129.1 (d, *J* = 5.5 Hz), 127.6, 123.8, 123.1, 122.9, 122.2, 120.5, 113.5, 110.1, 109.5, 64.8, 20.7, 15.4. *m*/*z* (ES^+^) [M + H]^+^ = 422.2.

#### 2-Chloro-4-(5-(8-methyl-5-(*p*-tolyl)-2*H*-chromen-3-yl)-1*H*-pyrrol-2-yl)­benzoic Acid (14)

Yellow solid (45% yield). ^1^H NMR (400 MHz, DMSO-*d*_6_) δ: 11.45 (t, *J* = 2.4 Hz, 1H), 7.91 (d, *J* = 1.7 Hz, 1H), 7.80 (d, *J* = 8.3 Hz, 1H), 7.74–7.69 (m, 1H), 7.30 (s, 4H), 7.06 (d, *J* = 7.8 Hz, 1H), 6.98 (s, 1H), 6.85–6.79 (m, 2H), 6.43 (dd, *J* = 3.9, 2.3 Hz, 1H), 5.00 (s, 2H), 2.37 (s, 3H), 2.19 (s, 3H). *m*/*z* (ES^+^) [M + H]^+^ = 456.1.

#### 2-Fluoro-4-(5-(8-methyl-5-(*p*-tolyl)-2*H*-chromen-3-yl)-1*H*-pyrrol-2-yl)­benzoic Acid (15)

Yellow solid (41% yield). ^1^H NMR (400 MHz, DMSO-*d*_6_) δ: 13.03 (s, 1H), 11.43 (d, *J* = 2.6 Hz, 1H), 7.84 (t, *J* = 8.2 Hz, 1H), 7.74–7.59 (m, 2H), 7.31 (s, 4H), 7.08 (d, *J* = 7.8 Hz, 1H), 7.01 (s, 1H), 6.89–6.80 (m, 2H), 6.45 (dd, *J* = 3.8, 2.2 Hz, 1H), 5.02 (s, 2H), 2.39 (s, 3H), 2.21 (s, 3H). ^13^C NMR (101 MHz, DMSO-*d*_6_) δ: 164.7, 163.1, 160.6, 151.5, 138.1 (d, *J* = 9.8 Hz), 136.8, 136.6, 136.4, 132.4, 132.1, 131.6 (d, *J* = 2.4 Hz), 129.3 (d, *J* = 7.9 Hz), 129.1, 123.0 (d, *J* = 3.4 Hz), 122.2, 120.4, 119.5, 115.4, 115.3, 114.0, 111.4, 111.2, 110.5, 110.2, 64.7, 20.7, 15.4. *m*/*z* (ES^+^) [M + H]^+^ = 440.2.

#### 2,6-Difluoro-4-(5-(8-methyl-5-(*p*-tolyl)-2*H*-chromen-3-yl)-1*H*-pyrrol-2-yl)­benzoic Acid (16)

Yellow solid (34% yield). ^1^H NMR (400 MHz, DMSO-*d*_6_) δ: 11.38 (t, *J* = 2.5 Hz, 1H), 7.61–7.52 (m, 2H), 7.29 (s, 4H), 7.06 (d, *J* = 7.8 Hz, 1H), 6.98 (s, 1H), 6.90 (dd, *J* = 3.9, 2.3 Hz, 1H), 6.83 (d, *J* = 7.7 Hz, 1H), 6.44 (dd, *J* = 3.9, 2.3 Hz, 1H), 5.00 (s, 2H), 2.37 (s, 3H), 2.19 (s, 3H). ^13^C NMR (101 MHz, DMSO-*d*_6_) δ: 162.0, 161.5 (d, *J* = 8.2 Hz), 159.0 (d, *J* = 8.1 Hz), 151.6, 136.8 (d, *J* = 8.2 Hz), 136.6, 136.4 (d, *J* = 5.7 Hz), 132.3, 130.8 (d, *J* = 2.9 Hz), 129.4 (d, *J* = 4.4 Hz), 129.1, 123.0, 122.8, 122.2, 120.3, 114.2, 111.1, 110.2, 107.8, 107.0, 106.7, 64.7, 20.7, 15.4. *m*/*z* (ES^+^) [M + H]^+^ = 458.2.

#### 2-Hydroxy-4-(5-(8-methyl-5-(*p*-tolyl)-2*H*-chromen-3-yl)-1*H*-pyrrol-2-yl)­benzoic Acid (17)

Yellow solid (45% yield). ^1^H NMR (400 MHz, DMSO-*d*_6_) δ: 11.37 (t, *J* = 2.5 Hz, 1H), 7.71 (d, *J* = 8.4 Hz, 1H), 7.36 (d, *J* = 1.7 Hz, 1H), 7.32–7.24 (m, 5H), 7.06 (d, *J* = 7.8 Hz, 1H), 7.02 (s, 1H), 6.82 (d, *J* = 7.7 Hz, 1H), 6.77 (dd, *J* = 3.8, 2.3 Hz, 1H), 6.42 (dd, *J* = 3.8, 2.3 Hz, 1H), 4.99 (s, 2H), 2.38 (s, 3H), 2.19 (s, 3H). *m*/*z* (ES^+^) [M + H]^+^ = 438.2.

#### 2-Methoxy-4-(5-(8-methyl-5-(*p*-tolyl)-2*H*-chromen-3-yl)-1*H*-pyrrol-2-yl)­benzoic Acid (18)

Yellow solid (40% yield). ^1^H NMR (400 MHz, DMSO-*d*_6_) δ: 11.36 (t, *J* = 2.5 Hz, 1H), 7.66 (d, *J* = 8.0 Hz, 1H), 7.38–7.25 (m, 6H), 7.05 (d, *J* = 7.8 Hz, 1H), 6.93 (s, 1H), 6.82 (d, *J* = 7.8 Hz, 1H), 6.77 (dd, *J* = 3.8, 2.3 Hz, 1H), 6.41 (dd, *J* = 3.9, 2.2 Hz, 1H), 5.01 (s, 2H), 3.86 (s, 3H), 2.36 (s, 3H), 2.19 (s, 3H). ^13^C NMR (101 MHz, DMSO-*d*_6_) δ: 166.7, 159.0, 151.4, 136.8, 136.7, 136.6, 136.3, 132.9, 131.6, 131.3, 129.4, 129.1 (d, *J* = 10.4 Hz), 123.3, 123.0, 122.2, 120.4, 117.7, 115.8, 113.5, 109.9, 109.6, 107.8, 64.8, 55.9, 20.7, 15.4. *m*/*z* (ES^+^) [M + H]^+^ = 452.2.

#### 4-(5-(5-(*p*-Tolyl)-8-(trifluoromethyl)-2*H*-chromen-3-yl)-1*H*-pyrrol-2-yl)­benzoic Acid (19)

Yellow solid (61% yield). ^1^H NMR (400 MHz, DMSO-*d*_6_) δ: 11.51 (d, *J* = 2.5 Hz, 1H), 7.93–7.88 (m, 2H), 7.81 (d, *J* = 8.5 Hz, 2H), 7.47 (d, *J* = 8.2 Hz, 1H), 7.43–7.33 (m, 4H), 7.10–7.01 (m, 2H), 6.77 (dd, *J* = 3.9, 2.3 Hz, 1H), 6.52 (dd, *J* = 3.8, 2.3 Hz, 1H), 5.13 (s, 2H), 2.40 (s, 3H). *m*/*z* (ES^+^) [M + H]^+^ = 476.1.

#### 4-(5-(8-Ethyl-5-(*p*-tolyl)-2*H*-chromen-3-yl)-1*H*-pyrrol-2-yl)­benzoic Acid (20)

Yellow solid (39% yield). ^1^H NMR (400 MHz, DMSO-*d*_6_) δ: 11.41 (t, *J* = 2.4 Hz, 1H), 7.92–7.86 (m, 2H), 7.85–7.76 (m, 2H), 7.36–7.24 (m, 4H), 7.07 (d, *J* = 7.8 Hz, 1H), 6.99 (s, 1H), 6.85 (d, *J* = 7.8 Hz, 1H), 6.75 (dd, *J* = 3.8, 2.3 Hz, 1H), 6.43 (dd, *J* = 3.8, 2.2 Hz, 1H), 4.99 (s, 2H), 2.60 (q, *J* = 7.5 Hz, 2H), 2.37 (s, 3H), 1.17 (t, *J* = 7.5 Hz, 3H). *m*/*z* (ES^+^) [M + H]^+^ = 436.2.

#### 4-(5-(8-Isopropyl-5-(*p*-tolyl)-2*H*-chromen-3-yl)-1*H*-pyrrol-2-yl)­benzoic Acid (21)

Yellow solid (47% yield). ^1^H NMR (400 MHz, DMSO-*d*_6_) δ: 11.41 (t, *J* = 2.4 Hz, 1H), 7.89 (d, *J* = 8.5 Hz, 2H), 7.80 (d, *J* = 8.5 Hz, 2H), 7.34–7.24 (m, 4H), 7.12 (d, *J* = 8.0 Hz, 1H), 7.00 (s, 1H), 6.88 (d, *J* = 7.9 Hz, 1H), 6.75 (dd, *J* = 3.8, 2.3 Hz, 1H), 6.43 (dd, *J* = 3.8, 2.3 Hz, 1H), 4.98 (s, 2H), 3.30–3.21 (m, 1H), 2.37 (s, 3H), 1.21 (d, *J* = 6.8 Hz, 6H). ^13^C NMR (101 MHz, DMSO-*d*_6_) δ: 167.1, 150.5, 136.6, 136.6, 136.3, 136.0, 133.6, 132.9, 131.5, 129.7, 129.4, 129.1, 127.6, 124.6, 123.8, 123.4, 122.6, 120.8, 113.6, 110.2, 109.5, 78.9, 64.7, 26.3, 22.4, 20.7. *m*/*z* (ES^+^) [M + H]^+^ = 450.2.

#### 4-(5-(8-Fluoro-5-(*p*-tolyl)-2*H*-chromen-3-yl)-1*H*-pyrrol-2-yl)­benzoic Acid (22)

Yellow solid (51% yield). ^1^H NMR (400 MHz, DMSO-*d*_6_) δ: 12.82 (s, 1H), 11.50 (t, *J* = 2.5 Hz, 1H), 7.96–7.88 (m, 2H), 7.88–7.79 (m, 2H), 7.32 (s, 4H), 7.14 (dd, *J* = 10.5, 8.5 Hz, 1H), 7.03 (d, *J* = 1.6 Hz, 1H), 6.96–6.88 (m, 1H), 6.78 (dd, *J* = 3.8, 2.3 Hz, 1H), 6.49 (dd, *J* = 3.8, 2.3 Hz, 1H), 5.09 (d, *J* = 1.1 Hz, 2H), 2.40 (s, 3H). ^13^C NMR (101 MHz, DMSO-*d*_6_) δ: 167.0, 150.7, 148.2, 140.6, 140.4, 136.6, 135.9, 135.8, 134.5 (d, *J* = 3.2 Hz), 133.4, 130.9, 129.7, 129.4, 129.2, 127.8, 124.4, 123.9, 123.4 (d, *J* = 2.4 Hz), 122.5 (d, *J* = 7.0 Hz), 114.6, 114.4, 112.5, 110.8, 109.5, 64.9, 20.7. *m*/*z* (ES^+^) [M + H]^+^ = 426.1.

#### (*S*)-4-(5-(2,8-Dimethyl-5-(*p*-tolyl)-2*H*-chromen-3-yl)-1*H*-pyrrol-2-yl)­benzoic Acid (23)

Yellow solid (38% yield). ^1^H NMR (400 MHz, CDCl_3_) δ: 8.46 (s, 1H), 8.12–8.05 (m, 2H), 7.60–7.49 (m, 2H), 7.32 (q, *J* = 8.0 Hz, 4H), 7.07 (d, *J* = 7.7 Hz, 1H), 6.85 (d, *J* = 7.7 Hz, 1H), 6.68 (dd, *J* = 3.8, 2.6 Hz, 1H), 6.56 (s, 1H), 6.38–6.32 (m, 1H), 5.41 (q, *J* = 6.5 Hz, 1H), 2.46 (s, 3H), 2.28 (s, 3H), 1.53 (d, *J* = 6.5 Hz, 3H). *m*/*z* (ES^+^) [M + H]^+^ = 436.2.

#### 2-Fluoro-4-(5-(8-fluoro-5-(*p*-tolyl)-2*H*-chromen-3-yl)-1*H*-pyrrol-2-yl)­benzoic Acid (24)

Yellow solid (55% yield). ^1^H NMR (400 MHz, DMSO-*d*_6_) δ: 11.48 (t, *J* = 2.5 Hz, 1H), 7.83 (t, *J* = 8.2 Hz, 1H), 7.73–7.56 (m, 2H), 7.31 (s, 4H), 7.14 (dd, *J* = 10.5, 8.5 Hz, 1H), 7.01 (d, *J* = 1.5 Hz, 1H), 6.90 (dd, *J* = 8.6, 5.0 Hz, 1H), 6.86 (dd, *J* = 3.9, 2.4 Hz, 1H), 6.49 (dd, *J* = 3.9, 2.3 Hz, 1H), 5.11–5.03 (m, 2H), 2.38 (s, 3H). *m*/*z* (ES^+^) [M + H]^+^ = 444.1.

#### (*S*)-4-(5-(2,8-Dimethyl-5-(*p*-tolyl)-2*H*-chromen-3-yl)-1*H*-pyrrol-2-yl)-2-fluorobenzoic Acid (25)

Yellow solid (46% yield). ^1^H NMR (400 MHz, DMSO-*d*_6_) δ: 11.36 (s, 1H), 7.81 (t, *J* = 8.2 Hz, 1H), 7.71–7.57 (m, 2H), 7.30 (s, 4H), 7.07 (d, *J* = 7.9 Hz, 1H), 6.95 (s, 1H), 6.83 (dd, *J* = 3.8, 2.3 Hz, 1H), 6.80 (d, *J* = 7.7 Hz, 1H), 6.43 (dd, *J* = 3.9, 2.3 Hz, 1H), 5.46 (q, *J* = 6.4 Hz, 1H), 2.38 (s, 3H), 2.19 (s, 3H), 1.39 (d, *J* = 6.4 Hz, 3H). *m*/*z* (ES^+^) [M + H]^+^ = 454.2.

#### (*S*)-4-(5-(8-Fluoro-2-methyl-5-(*p*-tolyl)-2*H*-chromen-3-yl)-1*H*-pyrrol-2-yl)­benzoic Acid (26)

Yellow solid (45% yield). ^1^H NMR (400 MHz, DMSO-*d*_6_) δ: 11.43 (s, 1H), 7.91–7.85 (m, 2H), 7.84–7.77 (m, 2H), 7.31 (s, 4H), 7.13 (dd, *J* = 10.5, 8.5 Hz, 1H), 6.96 (d, *J* = 1.7 Hz, 1H), 6.87 (dd, *J* = 8.5, 4.9 Hz, 1H), 6.76 (dd, *J* = 3.8, 2.3 Hz, 1H), 6.48 (dd, *J* = 3.9, 2.3 Hz, 1H), 5.53 (q, *J* = 6.4 Hz, 1H), 2.38 (s, 3H), 1.43 (d, *J* = 6.4 Hz, 3H). *m*/*z* (ES^+^) [M + H]^+^ = 440.2.

#### (*S*)-2-Fluoro-4-(5-(8-fluoro-2-methyl-5-(*p*-tolyl)-2*H*-chromen-3-yl)-1*H*-pyrrol-2-yl)­benzoic Acid (27)

Yellow solid (33% yield). ^1^H NMR (400 MHz, DMSO-*d*_6_) δ: 11.42 (t, *J* = 2.5 Hz, 1H), 7.82 (t, *J* = 8.2 Hz, 1H), 7.72–7.56 (m, 2H), 7.31 (s, 4H), 7.15 (dd, *J* = 10.5, 8.5 Hz, 1H), 6.96 (d, *J* = 1.6 Hz, 1H), 6.90–6.83 (m, 2H), 6.49 (dd, *J* = 3.9, 2.3 Hz, 1H), 5.53 (q, *J* = 6.4 Hz, 1H), 2.38 (s, 3H), 1.43 (d, *J* = 6.5 Hz, 3H). *m*/*z* (ES^+^) [M + H]^+^ = 458.2.

#### 2-Fluoro-4-(5-(5-(*p*-tolyl)-8-(trifluoromethyl)-2*H*-chromen-3-yl)-1*H*-pyrrol-2-yl)­benzoic Acid (28)

Yellow solid (52% yield). ^1^H NMR (400 MHz, DMSO-*d*_6_) δ: 11.44 (t, *J* = 2.5 Hz, 1H), 7.83–7.71 (m, 1H), 7.67–7.53 (m, 2H), 7.43 (d, *J* = 8.2 Hz, 1H), 7.35–7.27 (m, 4H), 7.05–6.96 (m, 2H), 6.81 (dd, *J* = 3.9, 2.3 Hz, 1H), 6.47 (dd, *J* = 3.9, 2.2 Hz, 1H), 5.08 (s, 2H), 2.35 (s, 3H). *m*/*z* (ES^+^) [M + H]^+^ = 494.1.

#### 2,6-Difluoro-4-(5-(5-(*p*-tolyl)-8-(trifluoromethyl)-2*H*-chromen-3-yl)-1*H*-pyrrol-2-yl)­benzoic Acid (29)

Yellow solid (52% yield). ^1^H NMR (400 MHz, DMSO-*d*_6_) δ: 11.37 (s, 1H), 7.47 (d, *J* = 8.2 Hz, 1H), 7.43–7.33 (m, 6H), 7.06 (d, *J* = 8.2 Hz, 1H), 7.00 (s, 1H), 6.79–6.72 (m, 1H), 6.49 (dd, *J* = 3.9, 2.3 Hz, 1H), 5.12 (s, 2H), 2.40 (s, 3H). *m*/*z* (ES^+^) [M + H]^+^ = 512.1.

#### Methyl 4-(5-(5-Bromo-8-methyl-2*H*-chromen-3-yl)-1*H*-pyrrol-2-yl)­benzoate (64)

5-Bromo-3-iodo-2H-chromene **63a** (520 mg, 1.5 mmol), methyl 4-(5-(4,4,5,5-tetramethyl-1,3,2-dioxaborolan-2-yl)-1*H*-pyrrol-2-yl)­benzoate **26a** (490 mg, 1.5 mmol), Pd­(PPh_3_)_4_ (170 mg, 150 μmol), K_2_CO_3_ (510 mg, 3.7 mmol) were dissolved in 1,4-dioxane/water (10/1, 10 mL). This mixture was stirred at 100 °C for 3 h under nitrogen. After the reaction was complete, the solvent was removed under reduced pressure, and the crude product was purified by flash chromatography using hexanes and EtOAc as eluents to afford methyl 4-(5-(5-bromo-8-methyl-2H-chromen-3-yl)-1*H*-pyrrol-2-yl)­benzoate (**64**) (410 mg, 64% yield) as a yellow solid. ^1^H NMR (400 MHz, CDCl_3_) δ: 8.73 (s, 1H), 8.11–8.03 (m, 2H), 7.65–7.57 (m, 2H), 7.07 (d, *J* = 8.1 Hz, 1H), 6.88–6.81 (m, 2H), 6.69 (dd, *J* = 3.9, 2.5 Hz, 1H), 6.40 (dd, *J* = 3.8, 2.5 Hz, 1H), 5.03 (d, *J* = 1.3 Hz, 2H), 3.94 (s, 3H), 2.18 (s, 3H). ^13^C NMR (101 MHz, CDCl_3_) δ: 166.7, 152.2, 135.8, 133.4, 130.4, 130.3, 130.3, 128.1, 124.9, 124.9, 124.6, 123.6, 122.1, 118.5, 113.1, 110.5, 109.7, 65.6, 52.1, 15.4.

#### 4-(5-(8-Methyl-5-(quinolin-3-yl)-2*H*-chromen-3-yl)-1*H*-pyrrol-2-yl)­benzoic Acid (12)

Methyl 4-(5-(5-bromo-8-methyl-2*H*-chromen-3-yl)-1*H*-pyrrol-2-yl)­benzoate (**64**) (150 mg, 350 μmol), 3-(4,4,5,5-tetramethyl-1,3,2-dioxaborolan-2-yl)­quinoline (110 mg, 420 μmol), Pd­(amphos)­Cl_2_ (25 mg, 35 μmol), K_2_CO_3_ (98 mg, 710 μmol) were dissolved in 1,4-dioxane/water (10/1, 10 mL). This mixture was stirred at 100 °C for 8 h under nitrogen. After completion of the reaction, lithium hydroxide monohydrate (150 mg, 3.5 mmol) in water (1 mL) was added, and the mixture was stirred for an additional 18 h at 40 °C. The resulting mixture was cooled to room temperature and acidified with HCl (1M) to pH 1, followed by extraction with ethyl acetate. The organic layer was washed with brine and dried over anhydrous MgSO_4_, then purified by flash chromatography using mixtures of hexanes and ethyl acetate to afford 4-(5-(8-methyl-5-(quinolin-3-yl)-2*H*-chromen-3-yl)-1*H*-pyrrol-2-yl)­benzoic acid (**12**) (39 mg, 24% yield) as a yellow solid. ^1^H NMR (400 MHz, DMSO-*d*_6_) δ: 11.33 (t, *J* = 2.5 Hz, 1H), 8.94 (d, *J* = 2.3 Hz, 1H), 8.40 (d, *J* = 2.2 Hz, 1H), 8.15–8.07 (m, 2H), 7.89–7.77 (m, 3H), 7.77–7.69 (m, 2H), 7.66 (ddd, *J* = 8.1, 6.8, 1.2 Hz, 1H), 7.16 (d, *J* = 7.8 Hz, 1H), 7.04 (s, 1H), 7.00 (d, *J* = 7.7 Hz, 1H), 6.74 (dd, *J* = 3.9, 2.3 Hz, 1H), 6.48 (dd, *J* = 3.8, 2.3 Hz, 1H), 5.07 (s, 2H), 2.25 (s, 3H). *m*/*z* (ES^+^) [M + H]^+^ = 459.2.

#### 4-(5-(8-Methyl-5-(phenylethynyl)-2*H*-chromen-3-yl)-1*H*-pyrrol-2-yl)­benzoic Acid (13)

To a reaction vial, methyl 4-(5-(5-bromo-8-methyl-2*H*-chromen-3-yl)-1*H*-pyrrol-2-yl)­benzoate (**64**) (150 mg, 350 μmol), ethynylbenzene (43 mg, 420 μmol), *tert*-butyldicyclohexylphosphonium tetrafluoroborate (39 mg, 110 μmol), sodium tetrachloropalladate­(II) (17 mg, 57 μmol), and copper­(I) iodide (8.1 mg, 42 μmol) were added under nitrogen, followed by addition of diisopropylamine (10 mL). The mixture was kept stirring at 80 °C for 16 h. After the reaction was completed, it was cooled to room temperature and filtered to remove the solids. The resulting filtrate was then concentrated in vacuo and purified by flash chromatography using mixtures of hexanes and ethyl acetate to afford methyl 4-(5-(8-methyl-5-(phenylethynyl)-2*H*-chromen-3-yl)-1*H*-pyrrol-2-yl)­benzoate (100 mg, 65% yield) as a yellow solid. ^1^H NMR (400 MHz, CDCl_3_) δ: 8.67 (s, 1H), 8.05 (d, *J* = 8.4 Hz, 2H), 7.58 (t, *J* = 8.6 Hz, 4H), 7.44–7.36 (m, 3H), 7.09–7.03 (m, 2H), 6.96 (d, *J* = 7.8 Hz, 1H), 6.70 (dd, *J* = 3.8, 2.5 Hz, 1H), 6.41 (dd, *J* = 3.8, 2.5 Hz, 1H), 5.08 (d, *J* = 1.4 Hz, 2H), 3.93 (s, 3H), 2.24 (s, 3H). Methyl 4-(5-(8-methyl-5-(phenylethynyl)-2*H*-chromen-3-yl)-1*H*-pyrrol-2-yl)­benzoate (103 mg, 231 μmol) was then dissolved in THF (8 mL) and water (2 mL), and lithium hydroxide monohydrate (150 mg, 3.5 mmol) was added. The reaction was stirred for 18 h at 40 °C. The resulting mixture was cooled to room temperature and acidified with HCl (1M) to pH 1, followed by extraction with ethyl acetate. The organic layer was washed with brine and dried over anhydrous MgSO_4_, then purified by flash chromatography using mixtures of hexanes and ethyl acetate to afford 4-(5-(8-methyl-5-(phenylethynyl)-2*H*-chromen-3-yl)-1*H*-pyrrol-2-yl)­benzoic acid (**13**) (91 mg, 91% yield) as a yellow solid. ^1^H NMR (400 MHz, DMSO-*d*_6_) δ: 11.73 (t, *J* = 2.4 Hz, 1H), 7.99–7.86 (m, 4H), 7.73–7.65 (m, 2H), 7.51–7.39 (m, 4H), 7.06 (d, *J* = 7.8 Hz, 1H), 7.01 (d, *J* = 7.9 Hz, 1H), 6.81 (dd, *J* = 3.9, 2.3 Hz, 1H), 6.54 (dd, *J* = 3.9, 2.3 Hz, 1H), 5.08 (d, *J* = 1.1 Hz, 2H), 2.18 (s, 3H). *m*/*z* (ES^+^) [M + H]^+^ = 432.2.

#### 3-Iodo-4-(*p*-tolyl)-2*H*-chromene (66)

To a mixture of 3-(*p*-tolyl)­prop-2-yn-1-ol (**65**) (200 mg, 1.4 mmol), MgSO_4_ (33 mg, 0.27 mmol), and *t*-BuONa (150 mg, 1.5 mmol) in a mixture of benzene and DCE (1:1, 5.0 mL) was added diphenyliodonium trifluoromethanesulfonate (650 mg, 1.5 mmol). The obtained mixture was stirred for 3 h at 60 °C under an argon atmosphere. Then, NIS (340 mg, 1.5 mmol) and BF_3_·OEt_2_ (186 μL, 1.50 mmol) were added at 0 °C, and the obtained mixture was stirred for 1 h at 0 °C. Saturated Na_2_SO_3_ aqueous solution (10 mL) was added to the reaction mixture, and the product was extracted with ethyl acetate. The organic layer was dried over MgSO_4_ and concentrated. The resulting crude was purified by flash chromatography using mixtures of hexanes and ethyl acetate to afford 3-iodo-4-(*p*-tolyl)-2*H*-chromene (380 mg, 80% yield) as a pale-yellow solid. ^1^H NMR (400 MHz, CDCl_3_) δ: 7.25 (d, *J* = 3.5 Hz, 2H), 7.15 (td, *J* = 7.8, 1.6 Hz, 1H), 7.12–7.07 (m, 2H), 6.85 (dd, *J* = 8.1, 1.2 Hz, 1H), 6.77 (td, *J* = 7.6, 1.2 Hz, 1H), 6.65 (dd, *J* = 7.7, 1.6 Hz, 1H), 5.06 (s, 2H), 2.42 (s, 3H).

#### Methyl 4-(5-(4-(*p*-Tolyl)-2*H*-chromen-3-yl)-1*H*-pyrrol-2-yl)­benzoate (67)

3-Iodo-4-(*p*-tolyl)-2*H*-chromene (**66**) (470 mg, 1.4 mmol), methyl 4-(5-(4,4,5,5-tetramethyl-1,3,2-dioxaborolan-2-yl)-1*H*-pyrrol-2-yl)­benzoate (**59a**) (490 mg, 1.5 mmol), K_3_PO_4_ (570 mg, 2.7 mmol), Pd­(OAc)_2_ (30 mg, 0.14 mmol), and XPhos (73 mg, 0.27 mmol) were dissolved in deoxygenated dioxane/water (10/1, 10 mL). The reaction mixture was stirred at 80 °C for 5 h under nitrogen. The solvent was removed, and the crude product was purified by flash chromatography using hexanes and ethyl acetate to afford methyl 4-(5-(4-(*p*-tolyl)-2*H*-chromen-3-yl)-1*H*-pyrrol-2-yl)­benzoate (420 mg, 73% yield) as a yellow solid. ^1^H NMR (400 MHz, CDCl_3_) δ: 7.93–7.83 (m, 2H), 7.63 (s, 1H), 7.48 (d, *J* = 7.8 Hz, 2H), 7.36–7.28 (m, 2H), 7.12 (td, *J* = 7.6, 1.6 Hz, 1H), 6.95–6.88 (m, 3H), 6.83 (td, *J* = 7.5, 1.2 Hz, 1H), 6.74 (dd, *J* = 7.8, 1.6 Hz, 1H), 6.53 (dd, *J* = 3.9, 2.6 Hz, 1H), 6.36 (dd, *J* = 3.9, 2.6 Hz, 1H), 5.15 (s, 2H), 3.91 (s, 3H), 2.58 (s, 3H).

#### 4-(5-(4-(*p*-Tolyl)-2*H*-chromen-3-yl)-1*H*-pyrrol-2-yl)­benzoic Acid (11)

The mixture of LiOH·H_2_O (420 mg, 10 mmol) and 4-(5-(4-(*p*-tolyl)-2*H*-chromen-3-yl)-1*H*-pyrrol-2-yl)­benzoate (**67**) (420 mg, 1.0 mmol) in THF (10 mL) and H_2_O (2 mL) was stirred at 40 °C for 18 h. The resulting mixture was cooled to room temperature and acidified with HCl (1M) to pH 1, followed by extraction with ethyl acetate. The organic layer was washed with brine and dried over anhydrous MgSO_4_, then purified by flash chromatography using mixtures of hexanes and ethyl acetate to afford 4-(5-(4-(*p*-tolyl)-2*H*-chromen-3-yl)-1*H*-pyrrol-2-yl)­benzoic acid (400 mg, 97% yield) as a yellow solid. ^1^H NMR (400 MHz, DMSO-*d*_6_) δ: 12.82 (s, 1H), 10.39 (s, 1H), 7.88 (d, *J* = 8.4 Hz, 2H), 7.66–7.54 (m, 2H), 7.35 (d, *J* = 7.7 Hz, 2H), 7.14 (dd, *J* = 7.8, 6.1 Hz, 3H), 6.93 (dd, *J* = 8.1, 1.2 Hz, 1H), 6.84 (td, *J* = 7.5, 1.2 Hz, 1H), 6.62 (dd, *J* = 7.8, 1.6 Hz, 1H), 6.55 (dd, *J* = 3.9, 2.4 Hz, 1H), 5.55 (dd, *J* = 3.9, 2.3 Hz, 1H), 5.21 (s, 2H), 2.42 (s, 3H). *m*/*z* (ES^+^) [M + H]^+^ = 408.2.

### Synthesis of Benzofuran and Benzothiophene Analogs

#### General Synthetic Method (3A) for **69a**–**69i**

To a stirred suspension containing **68a**–**68i** (1.0 equiv) and potassium carbonate (1.1 equiv) in DMF (0.2M), bromoacetaldehyde diethyl acetal (1.1 equiv) was added dropwise. The mixture was refluxed for 4 h. After the mixture was cooled, the precipitate was filtered off, and the solvent was evaporated under reduced pressure. The residue was purified by flash chromatography using mixtures of hexanes and ethyl acetate to afford corresponding products **69a**–**69i**.

#### General Synthetic Method (3B) for **70a**–**70i**

A stirred mixture of polyphosphoric acid (∼5 g) and chlorobenzene (8 mL) was heated to reflux, and a solution of **69a**–**69i** (1.0 equiv) in chlorobenzene (3 mL) was added dropwise over 10 min. The reaction mixture was maintained at reflux for 1.5 h, then cooled to room temperature. Aqueous 1 M NaOH (20 mL) was added, followed by extraction with ether. The organic layer was washed with water and brine, then dried over anhydrous MgSO_4_. The solvent was evaporated under reduced pressure, and the crude residue was purified by flash chromatography using hexanes/ethyl acetate mixtures to afford the corresponding products **70a**–**70i**.

#### General Synthetic Method (3C) for **71a**–**71j**

**70a**–**70i** (1.0 equiv), boronic acid pinacol esters (1.1 equiv), Pd­(amphos)­Cl_2_ (0.2 equiv), K_2_CO_3_ (2.0 equiv) were dissolved in 1,4-dioxane/water (10/1, 0.2 M). This mixture was stirred at 100 °C for 8 h under nitrogen. After the completion of the reaction, the reaction was cooled to room temperature and extracted with ethyl acetate. The organic layer was washed with brine, dried over anhydrous MgSO_4_, and purified by flash chromatography using mixtures of hexanes and ethyl acetate to afford the corresponding products **71a**–**71i**.

#### General Synthetic Method (3D) for **72a**–**72j**

To a solution of **71a**–**71j** (1.0 equiv) in THF (0.2 M) was added *n*-BuLi (1.7 M, 1.1 equiv) at −78 °C. After stirring at the same temperature for 30 min, a solution of iodine (1.1 equiv) in THF (0.5 M) was added dropwise. The mixture warmed up gradually to room temperature and was stirred for 1.5 h. The reaction was diluted with ethyl acetate (20 mL). Excess I_2_ was reduced with a 10% Na_2_S_2_O_7_ solution. The organic layer was washed with brine, dried over MgSO_4,_ and concentrated under reduced pressure. Purification by flash column chromatography afforded **72a**–**72j**.

#### General Synthetic Method (3E) for **30**–**33** and **35**–**50**

**72a**–**72j** (1.0 equiv), methyl 4-(5-(4,4,5,5-tetramethyl-1,3,2-dioxaborolan-2-yl)-1*H*-pyrrol-2-yl)­benzoates **59a**–**59e** (1.1 equiv), Pd­(PPh_3_)_4_ (0.1 equiv), K_2_CO_3_ (3.0 equiv) were dissolved in 1,4-dioxane/water (10/1, 10 mL). This mixture was stirred at 100 °C for 3 h under nitrogen. After completion of the reaction, lithium hydroxide monohydrate (10 equiv) in 1 mL of water was added, and the mixture was stirred for an additional 18 h at 40 °C. The resulting mixture was cooled to room temperature and acidified with HCl (1M) to pH 1, followed by extraction with ethyl acetate. The organic layer was washed with brine, dried over anhydrous MgSO_4_, and purified by flash chromatography using mixtures of hexanes and ethyl acetate to afford the desired products.

### Representative Synthesis: 44

#### 4-Bromo-2-(2,2-diethoxyethoxy)-1-ethylbenzene (**69g**)

5-Bromo-2-ethylphenol (**68g**) was used as the starting material and subjected to general synthetic method 3A. The reaction afforded 4-bromo-2-(2,2-diethoxyethoxy)-1-ethylbenzene (**69g**) as a colorless oil (600 mg, 86% yield). ^1^H NMR (400 MHz, CDCl_3_) δ: 7.06–6.98 (m, 2H), 6.96 (d, *J* = 1.7 Hz, 1H), 4.85 (t, *J* = 5.3 Hz, 1H), 3.98 (d, *J* = 5.3 Hz, 2H), 3.77 (dq, *J* = 9.4, 7.1 Hz, 2H), 3.64 (dq, *J* = 9.4, 7.0 Hz, 2H), 2.59 (q, *J* = 7.5 Hz, 2H), 1.25 (t, *J* = 7.0 Hz, 6H), 1.17 (t, *J* = 7.5 Hz, 3H). ^13^C NMR (101 MHz, CDCl_3_) δ: 156.9, 131.9, 130.1, 123.7, 119.5, 114.7, 100.4, 77.2, 68.8, 62.6, 31.6, 23.0, 15.3, 13.9.

#### 4-Bromo-7-ethylbenzofuran (**70g**)

4-Bromo-2-(2,2-diethoxyethoxy)-1-ethylbenzene (**69g**) was used as a starting material and subjected to general synthetic method 3B. The reaction afforded 4-bromo-7-ethylbenzofuran (**70g**) as a colorless oil (280 mg, 66% yield). ^1^H NMR (400 MHz, CDCl_3_) δ: 7.66 (d, *J* = 2.2 Hz, 1H), 7.31 (d, *J* = 7.9 Hz, 1H), 7.00 (d, *J* = 7.9 Hz, 1H), 6.80 (d, *J* = 2.2 Hz, 1H), 2.89 (qd, *J* = 7.6, 0.7 Hz, 2H), 1.32 (t, *J* = 7.6 Hz, 3H). ^13^C NMR (101 MHz, CDCl_3_) δ: 153.28, 144.94, 128.39, 127.24, 125.75, 124.50, 111.20, 106.97, 77.22, 22.65, 14.04.

#### 7-Ethyl-4-(*p*-tolyl)­benzofuran (**71g**)

4-Bromo-7-ethylbenzofuran (**70g**) was used as a starting material and subjected to general synthetic method 3C. The reaction afforded 7-ethyl-4-(*p*-tolyl)­benzofuran (**71g**) as a white solid (271 mg, 92% yield). ^1^H NMR (400 MHz, CDCl_3_) δ: 7.68 (d, *J* = 2.2 Hz, 1H), 7.57–7.51 (m, 2H), 7.31 (s, 2H), 7.27 (d, *J* = 2.6 Hz, 1H), 7.20 (d, *J* = 7.6 Hz, 1H), 6.96 (d, *J* = 2.2 Hz, 1H), 3.00 (q, *J* = 7.6 Hz, 2H), 2.44 (s, 3H), 1.40 (t, *J* = 7.6 Hz, 3H). ^13^C NMR (101 MHz, CDCl_3_) δ: 144.7, 137.4, 136.8, 132.8, 129.4, 129.3, 128.2, 126.8, 126.7, 123.6, 122.3, 106.3, 22.8, 21.1, 14.1.

#### 7-Ethyl-2-iodo-4-(*p*-tolyl)­benzofuran (**72g**)

7-Ethyl-4-(*p*-tolyl)­benzofuran (**71g**) was used as a starting material and subjected to general synthetic method 3D. The reaction afforded 7-ethyl-2-iodo-4-(*p*-tolyl)­benzofuran (**72g**) as a white solid (346 mg, 83% yield). ^1^H NMR (400 MHz, CDCl_3_) δ: 7.48–7.42 (m, 2H), 7.31–7.17 (m, 3H), 7.14–7.07 (m, 2H), 2.95 (q, *J* = 7.6 Hz, 2H), 2.42 (s, 3H), 1.36 (t, *J* = 7.6 Hz, 3H). ^13^C NMR (101 MHz, CDCl_3_) δ: 157.2, 137.0, 136.9, 131.5, 129.4, 128.1, 127.1, 126.8, 126.0, 123.6, 122.6, 117.0, 95.5, 22.6, 21.1, 14.1.

#### 4-(5-(7-Ethyl-4-(*p*-tolyl)­benzofuran-2-yl)-1*H*-pyrrol-2-yl)­benzoic Acid (**44**)

7-Ethyl-2-iodo-4-(*p*-tolyl)­benzofuran (**72g**) and pyrrole boronic acid pinacol ester **59a** were used as starting materials and subjected to general synthetic method 3E. The reaction afforded 4-(5-(7-ethyl-4-(*p*-tolyl)­benzofuran-2-yl)-1*H*-pyrrol-2-yl)­benzoic acid (**44**) as a yellow solid (237 mg, 65% yield). ^1^H NMR (400 MHz, DMSO-*d*_6_) δ: 12.86 (s, 1H), 11.81 (t, *J* = 2.4 Hz, 1H), 7.97 (d, *J* = 8.6 Hz, 2H), 7.90 (d, *J* = 8.6 Hz, 2H), 7.60–7.54 (m, 2H), 7.44 (s, 1H), 7.36 (d, *J* = 7.9 Hz, 2H), 7.25 (d, *J* = 7.6 Hz, 1H), 7.18 (d, *J* = 7.7 Hz, 1H), 6.87 (dd, *J* = 3.8, 2.4 Hz, 1H), 6.77 (dd, *J* = 3.8, 2.2 Hz, 1H), 2.97 (q, *J* = 7.6 Hz, 2H), 2.40 (s, 3H), 1.36 (t, *J* = 7.6 Hz, 3H). ^13^C NMR (101 MHz, DMSO-*d*_6_) δ: 167.5, 152.8, 150.2, 137.2, 136.9, 136.3, 133.5, 131.8, 130.3, 129.9, 128.3, 128.3, 127.1, 125.9, 124.1, 123.9, 122.8, 110.1, 110.0, 99.3, 22.5, 21.2, 14.6. *m*/*z* (ES^+^) [M + H]^+^ = 422.2.

### Characterization for Other Final Compounds

#### 4-(5-(7-(*p*-Tolyl)­benzofuran-2-yl)-1*H*-pyrrol-2-yl)­benzoic Acid (**30**)

Yellow solid. ^1^H NMR (400 MHz, DMSO-*d*_6_) δ: 12.86 (s, 1H), 11.91 (d, *J* = 3.0 Hz, 1H), 7.97 (d, *J* = 8.2 Hz, 2H), 7.90 (d, *J* = 8.3 Hz, 2H), 7.86 (d, *J* = 7.9 Hz, 2H), 7.62 (d, *J* = 7.6 Hz, 1H), 7.46 (d, *J* = 7.6 Hz, 1H), 7.38 (d, *J* = 7.9 Hz, 2H), 7.32 (t, *J* = 7.6 Hz, 1H), 7.28 (s, 1H), 6.89–6.83 (m, 1H), 6.71 (dd, *J* = 3.7, 2.2 Hz, 1H), 2.40 (s, 3H). *m*/*z* (ES^+^) [M + H]^+^ = 394.1.

#### 4-(5-(4-Methyl-7-(*p*-tolyl)­benzofuran-2-yl)-1*H*-pyrrol-2-yl)­benzoic Acid (**31**)

Yellow solid. ^1^H NMR (400 MHz, DMSO-*d*_6_) δ: 12.85 (s, 1H), 11.89 (s, 1H), 7.97 (d, *J* = 8.6 Hz, 2H), 7.94–7.87 (m, 2H), 7.87–7.80 (m, 2H), 7.37 (dd, *J* = 7.9, 2.8 Hz, 3H), 7.33 (s, 1H), 7.14 (d, *J* = 7.7 Hz, 1H), 6.87 (dd, *J* = 3.8, 2.4 Hz, 1H), 6.71 (dd, *J* = 3.8, 2.2 Hz, 1H), 2.54 (s, 3H), 2.39 (s, 3H). ^13^C NMR (101 MHz, DMSO-*d*_6_) δ: 167.0, 150.3, 149.3, 136.7, 135.9, 133.0, 133.0, 129.9, 129.5, 129.3, 129.0, 127.8, 127.8, 125.5, 125.4, 124.0, 123.6, 122.6, 121.5, 109.7, 109.5, 98.2, 59.7, 20.7, 20.7, 18.1, 14.05. *m*/*z* (ES^+^) [M + H]^+^ = 408.2.

#### 4-(5-(4-(*p*-Tolyl)­benzofuran-2-yl)-1*H*-pyrrol-2-yl)­benzoic Acid (**32**)

Yellow solid. ^1^H NMR (400 MHz, DMSO-*d*_6_O) δ: 12.83 (s, 1H), 11.86 (t, *J* = 2.5 Hz, 1H), 7.96 (d, *J* = 8.6 Hz, 2H), 7.91 (d, *J* = 8.5 Hz, 2H), 7.64–7.52 (m, 3H), 7.45 (d, *J* = 0.9 Hz, 1H), 7.42–7.29 (m, 4H), 6.87 (dd, *J* = 3.8, 2.4 Hz, 1H), 6.78 (dd, *J* = 3.8, 2.2 Hz, 1H), 2.42 (s, 3H). ^13^C NMR (101 MHz, DMSO-*d*_6_) δ: 167.0, 154.0, 150.1, 136.8, 136.5, 135.8, 133.7, 133.1, 129.8, 129.4, 127.9, 127.9, 127.1, 125.2, 124.1, 123.6, 122.2, 109.8, 109.6, 109.5, 98.5, 20.7. *m*/*z* (ES^+^) [M + H]^+^ = 394.1.

#### 4-(5-(7-Methyl-4-(*p*-tolyl)­benzofuran-2-yl)-1*H*-pyrrol-2-yl)­benzoic Acid (**33**)

Yellow solid. ^1^H NMR (400 MHz, DMSO-*d*_6_) δ: 12.85 (s, 1H), 11.81 (t, *J* = 2.4 Hz, 1H), 7.97 (d, *J* = 8.5 Hz, 2H), 7.90 (d, *J* = 8.5 Hz, 2H), 7.61–7.54 (m, 2H), 7.45 (s, 1H), 7.37 (d, *J* = 7.8 Hz, 2H), 7.23 (d, *J* = 7.6 Hz, 1H), 7.19–7.13 (m, 1H), 6.87 (dd, *J* = 3.8, 2.4 Hz, 1H), 6.78 (dd, *J* = 3.7, 2.2 Hz, 1H), 2.56 (s, 3H), 2.41 (s, 3H). ^13^C NMR (101 MHz, DMSO-*d*_6_) δ: 167.0, 152.8, 149.8, 136.7, 136.4, 135.8, 133.0, 131.3, 129.8, 129.4, 127.9, 127.8, 126.5, 125.4, 125.0, 123.6, 122.1, 119.2, 109.6, 109.5, 98.9, 59.7, 20.7, 14.5, 14.0. *m*/*z* (ES^+^) [M + H]^+^ = 408.2.

#### 4-(5-(7-Methyl-4-(naphthalen-2-yl)­benzofuran-2-yl)-1*H*-pyrrol-2-yl)­benzoic Acid (**35**)

Yellow solid. ^1^H NMR (400 MHz, DMSO-*d*_6_) δ: 12.87 (s, 1H), 11.84 (t, *J* = 2.5 Hz, 1H), 8.21 (d, *J* = 1.8 Hz, 1H), 8.09 (dd, *J* = 15.6, 7.6 Hz, 2H), 8.04–7.99 (m, 1H), 7.96 (d, *J* = 8.5 Hz, 2H), 7.93–7.82 (m, 3H), 7.67–7.52 (m, 3H), 7.40 (d, *J* = 7.6 Hz, 1H), 7.23 (d, *J* = 7.6 Hz, 1H), 6.88 (dd, *J* = 3.8, 2.4 Hz, 1H), 6.81 (dd, *J* = 3.8, 2.2 Hz, 1H), 2.60 (s, 3H). *m*/*z* (ES^+^) [M + H]^+^ = 444.2.

#### 2-Fluoro-4-(5-(7-methyl-4-(*p*-tolyl)­benzofuran-2-yl)-1*H*-pyrrol-2-yl)­benzoic Acid (**36**)

Yellow solid. ^1^H NMR (400 MHz, DMSO-*d*_6_) δ: 13.06 (s, 1H), 11.83 (t, *J* = 2.5 Hz, 1H), 7.89 (t, *J* = 8.1 Hz, 1H), 7.79–7.67 (m, 2H), 7.60–7.53 (m, 2H), 7.43 (s, 1H), 7.36 (d, *J* = 7.9 Hz, 2H), 7.23 (d, *J* = 7.6 Hz, 1H), 7.17 (dd, *J* = 7.6, 0.9 Hz, 1H), 6.96 (dd, *J* = 3.8, 2.4 Hz, 1H), 6.79 (dd, *J* = 3.8, 2.2 Hz, 1H), 2.56 (s, 3H), 2.40 (s, 3H). ^13^C NMR (101 MHz, DMSO-*d*_6_) δ: 164.7, 164.7, 163.2, 160.7, 152.8, 149.6, 138.0, 137.9, 136.7, 136.4, 132.5, 131.8, 131.8, 131.4, 129.4, 127.8, 126.4, 125.9, 125.2, 122.2, 119.4, 119.2, 115.7, 115.6, 111.4, 111.1, 110.6, 109.7, 99.2, 20.7, 14.5. *m*/*z* (ES^+^) [M + H]^+^ = 426.1.

#### 2,6-Difluoro-4-(5-(7-methyl-4-(*p*-tolyl)­benzofuran-2-yl)-1*H*-pyrrol-2-yl)­benzoic Acid (**37**)

Yellow solid. ^1^H NMR (400 MHz, DMSO-*d*_6_) δ: 13.69 (s, 1H), 11.81 (t, *J* = 2.4 Hz, 1H), 7.70–7.61 (m, 2H), 7.59–7.52 (m, 2H), 7.43–7.33 (m, 3H), 7.23 (d, *J* = 7.5 Hz, 1H), 7.21–7.14 (m, 1H), 7.01 (dd, *J* = 3.8, 2.5 Hz, 1H), 6.79 (dd, *J* = 3.8, 2.2 Hz, 1H), 2.56 (s, 3H), 2.40 (s, 3H). ^13^C NMR (101 MHz, DMSO-*d*_6_) δ: 162.0, 159.1, 159.0, 152.9, 149.4, 136.6, 136.5, 131.4, 131.0, 129.4, 127.8, 126.3, 126.1, 125.3, 122.2, 119.3, 111.1, 109.7, 108.1, 107.0, 106.7, 99.4, 20.7, 14.5. *m*/*z* (ES^+^) [M + H]^+^ = 444.1.

#### 2-Chloro-4-(5-(7-methyl-4-(*p*-tolyl)­benzofuran-2-yl)-1*H*-pyrrol-2-yl)­benzoic Acid (**38**)

Yellow solid. ^1^H NMR (400 MHz, DMSO-*d*_6_) δ: 13.24 (s, 1H), 11.86 (t, *J* = 2.4 Hz, 1H), 7.99 (d, *J* = 1.7 Hz, 1H), 7.91–7.78 (m, 2H), 7.60–7.53 (m, 2H), 7.42 (s, 1H), 7.40–7.33 (m, 2H), 7.23 (d, *J* = 7.6 Hz, 1H), 7.17 (dd, *J* = 7.6, 0.9 Hz, 1H), 6.94 (dd, *J* = 3.8, 2.5 Hz, 1H), 6.79 (dd, *J* = 3.8, 2.2 Hz, 1H), 2.56 (d, *J* = 0.7 Hz, 3H), 2.40 (s, 3H). *m*/*z* (ES^+^) [M + H]^+^ = 442.1.

#### 2-Hydroxy-4-(5-(7-methyl-4-(*p*-tolyl)­benzofuran-2-yl)-1*H*-pyrrol-2-yl)­benzoic Acid (**39**)

Yellow solid. ^1^H NMR (400 MHz, DMSO-*d*_6_) δ: 13.87 (s, 1H), 11.78 (t, *J* = 2.5 Hz, 1H), 11.42 (s, 1H), 7.79 (d, *J* = 8.3 Hz, 1H), 7.60–7.54 (m, 2H), 7.50–7.41 (m, 2H), 7.40–7.32 (m, 3H), 7.23 (d, *J* = 7.6 Hz, 1H), 7.16 (dd, *J* = 7.6, 1.0 Hz, 1H), 6.89 (dd, *J* = 3.8, 2.5 Hz, 1H), 6.77 (dd, *J* = 3.8, 2.2 Hz, 1H), 2.55 (s, 3H), 2.40 (s, 3H). *m*/*z* (ES^+^) [M + H]^+^ = 424.1.

#### 4-(5-(4-Methyl-7-(*p*-tolyl)­benzo­[*b*]­thiophen-2-yl)-1*H*-pyrrol-2-yl)­benzoic Acid (**40**)

Yellow solid. ^1^H NMR (400 MHz, DMSO-*d*_6_) δ: 12.84 (s, 1H), 11.80 (t, *J* = 2.5 Hz, 1H), 7.98–7.88 (m, 5H), 7.62–7.56 (m, 2H), 7.36 (d, *J* = 7.9 Hz, 2H), 7.31–7.25 (m, 1H), 7.22 (d, *J* = 7.5 Hz, 1H), 6.80 (dd, *J* = 3.8, 2.4 Hz, 1H), 6.58 (dd, *J* = 3.8, 2.3 Hz, 1H), 2.64 (s, 3H), 2.40 (s, 3H). *m*/*z* (ES^+^) [M + H]^+^ = 424.1.

#### 4-(5-(7-Methyl-4-(*p*-tolyl)­benzo­[*b*]­thiophen-2-yl)-1*H*-pyrrol-2-yl)­benzoic Acid (**41**)

Yellow solid. ^1^H NMR (400 MHz, DMSO-*d*_6_) δ: 12.86 (s, 1H), 11.83 (t, *J* = 2.5 Hz, 1H), 7.97–7.84 (m, 5H), 7.52–7.46 (m, 2H), 7.38 (d, *J* = 7.8 Hz, 2H), 7.23 (q, *J* = 7.5 Hz, 2H), 6.81 (dd, *J* = 3.8, 2.3 Hz, 1H), 6.60 (dd, *J* = 3.8, 2.2 Hz, 1H), 2.53 (s, 3H), 2.42 (s, 3H). *m*/*z* (ES^+^) [M + H]^+^ = 424.1.

#### 2-Fluoro-4-(5-(7-methyl-4-(*p*-tolyl)­benzo­[*b*]­thiophen-2-yl)-1*H*-pyrrol-2-yl)­benzoic Acid (**42**)

Yellow solid. ^1^H NMR (400 MHz, DMSO-*d*_6_) δ: 13.08 (s, 1H), 11.83 (t, *J* = 2.5 Hz, 1H), 7.93–7.82 (m, 2H), 7.73 (dd, *J* = 12.9, 1.7 Hz, 1H), 7.68 (dd, *J* = 8.2, 1.7 Hz, 1H), 7.52–7.46 (m, 2H), 7.38 (d, *J* = 7.8 Hz, 2H), 7.29–7.19 (m, 2H), 6.90 (dd, *J* = 3.8, 2.4 Hz, 1H), 6.62 (dd, *J* = 3.8, 2.3 Hz, 1H), 2.53 (s, 3H), 2.42 (s, 3H). *m*/*z* (ES^+^) [M + H]^+^ = 442.1.

#### 2,6-Difluoro-4-(5-(7-methyl-4-(*p*-tolyl)­benzo­[*b*]­thiophen-2-yl)-1*H*-pyrrol-2-yl)­benzoic Acid (**43**)

Yellow solid. ^1^H NMR (400 MHz, DMSO-*d*_6_) δ: 13.68 (s, 1H), 11.80 (t, *J* = 2.5 Hz, 1H), 7.88 (s, 1H), 7.68–7.58 (m, 2H), 7.49 (d, *J* = 8.1 Hz, 2H), 7.38 (d, *J* = 7.8 Hz, 2H), 7.29–7.20 (m, 2H), 6.96 (dd, *J* = 3.9, 2.4 Hz, 1H), 6.62 (dd, *J* = 3.9, 2.2 Hz, 1H), 2.53 (s, 3H), 2.42 (s, 3H). *m*/*z* (ES^+^) [M + H]^+^ = 460.1.

#### 4-(5-(7-Isopropyl-4-(*p*-tolyl)­benzofuran-2-yl)-1*H*-pyrrol-2-yl)­benzoic Acid (**45**)

Yellow solid. ^1^H NMR (400 MHz, DMSO-*d*_6_) δ: 11.81 (t, *J* = 2.4 Hz, 1H), 8.01–7.94 (m, 2H), 7.90 (d, *J* = 8.6 Hz, 2H), 7.61–7.54 (m, 2H), 7.43 (s, 1H), 7.37 (d, *J* = 7.9 Hz, 2H), 7.29–7.18 (m, 2H), 6.87 (dd, *J* = 3.8, 2.4 Hz, 1H), 6.77 (dd, *J* = 3.8, 2.2 Hz, 1H), 3.51 (hept, *J* = 6.8 Hz, 1H), 2.41 (s, 3H), 1.40 (d, *J* = 6.9 Hz, 6H). ^13^C NMR (101 MHz, DMSO-*d*_6_) δ: 167.0, 151.7, 149.6, 136.7, 136.4, 135.8, 133.0, 131.3, 130.0, 129.8, 129.4, 127.8, 127.8, 126.8, 125.4, 123.6, 122.3, 121.1, 109.6, 109.5, 98.8, 27.9, 25.0, 22.6, 21.0, 20.7. *m*/*z* (ES^+^) [M + H]^+^ = 436.2.

#### 2-Fluoro-4-(5-(7-isopropyl-4-(*p*-tolyl)­benzofuran-2-yl)-1*H*-pyrrol-2-yl)­benzoic Acid (**46**)

Yellow solid. ^1^H NMR (400 MHz, DMSO-*d*_6_) δ: 13.03 (s, 1H), 11.76 (t, *J* = 2.5 Hz, 1H), 7.82 (t, *J* = 8.1 Hz, 1H), 7.72–7.60 (m, 2H), 7.52–7.46 (m, 2H), 7.35 (s, 1H), 7.29 (d, *J* = 7.9 Hz, 2H), 7.23–7.12 (m, 2H), 6.89 (dd, *J* = 3.8, 2.4 Hz, 1H), 6.71 (dd, *J* = 3.8, 2.2 Hz, 1H), 3.44 (hept, *J* = 6.9 Hz, 1H), 2.33 (s, 3H), 1.33 (d, *J* = 6.9 Hz, 6H). *m*/*z* (ES^+^) [M + H]^+^ = 454.2.

#### 2,6-Difluoro-4-(5-(7-isopropyl-4-(*p*-tolyl)­benzofuran-2-yl)-1*H*-pyrrol-2-yl)­benzoic Acid (**47**)

Yellow solid. ^1^H NMR (400 MHz, DMSO-*d*_6_) δ: 13.63 (s, 1H), 11.73 (d, *J* = 2.8 Hz, 1H), 7.58 (d, *J* = 10.0 Hz, 2H), 7.49 (d, *J* = 8.0 Hz, 2H), 7.34–7.26 (m, 3H), 7.18 (q, *J* = 7.8 Hz, 2H), 6.95 (dd, *J* = 3.8, 2.4 Hz, 1H), 6.71 (dd, *J* = 3.8, 2.2 Hz, 1H), 3.44 (p, *J* = 6.9 Hz, 1H), 2.33 (s, 3H), 1.33 (d, *J* = 6.9 Hz, 6H). *m*/*z* (ES^+^) [M + H]^+^ = 472.2.

#### 4-(5-(4-(*p*-Tolyl)-7-(trifluoromethyl)­benzofuran-2-yl)-1*H*-pyrrol-2-yl)­benzoic Acid (**48**)

Yellow solid. ^1^H NMR (400 MHz, DMSO-*d*_6_) δ: 12.83 (s, 1H), 11.86 (d, *J* = 2.6 Hz, 1H), 7.91 (d, *J* = 8.5 Hz, 2H), 7.83 (d, *J* = 8.5 Hz, 2H), 7.58 (dd, *J* = 8.1, 3.6 Hz, 3H), 7.50 (s, 1H), 7.42 (d, *J* = 7.9 Hz, 1H), 7.37 (d, *J* = 7.8 Hz, 2H), 6.82 (dd, *J* = 3.8, 2.4 Hz, 1H), 6.71 (dd, *J* = 3.8, 2.2 Hz, 1H), 2.37 (s, 3H). *m*/*z* (ES^+^) [M + H]^+^ = 462.1.

#### 2-Fluoro-4-(5-(4-(*p*-tolyl)-7-(trifluoromethyl)­benzofuran-2-yl)-1*H*-pyrrol-2-yl)­benzoic Acid (**49**)

Yellow solid. ^1^H NMR (400 MHz, DMSO-*d*_6_) δ: 13.07 (s, 1H), 11.87 (t, *J* = 2.5 Hz, 1H), 7.84 (t, *J* = 8.1 Hz, 1H), 7.72–7.54 (m, 5H), 7.48 (s, 1H), 7.42 (d, *J* = 7.9 Hz, 1H), 7.36 (d, *J* = 7.8 Hz, 2H), 6.91 (t, *J* = 3.1 Hz, 1H), 6.72 (dd, *J* = 3.9, 2.2 Hz, 1H), 2.36 (s, 3H). *m*/*z* (ES^+^) [M + H]^+^ = 480.1.

#### 2,6-Difluoro-4-(5-(4-(*p*-tolyl)-7-(trifluoromethyl)­benzofuran-2-yl)-1*H*-pyrrol-2-yl)­benzoic Acid (**50**)

Yellow solid. ^1^H NMR (400 MHz, DMSO-*d*_6_O) δ: 13.85 (s, 1H), 11.92 (t, *J* = 2.5 Hz, 1H), 7.70–7.58 (m, 5H), 7.54–7.46 (m, 2H), 7.43 (d, *J* = 7.9 Hz, 2H), 7.01 (dd, *J* = 3.9, 2.4 Hz, 1H), 6.78 (dd, *J* = 3.8, 2.2 Hz, 1H), 2.43 (s, 3H). *m*/*z* (ES^+^) [M + H]^+^ = 498.1.

#### 7-Methyl-4-(phenylethynyl)­benzofuran (**73**)

To a reaction vial, 4-bromo-7-methylbenzofuran (**70d**) (150 mg, 710 μmol), ethynylbenzene (94 μL, 850 μmol), *tert*-butyldicyclohexylphosphonium tetrafluoroborate (78 mg, 230 μmol), sodium tetrachloropalladate­(II) (34 mg, 110 μmol), and copper­(I) iodide (16 mg, 85 μmol) were added under nitrogen, followed by addition of diisopropylamine (10 mL). The mixture was kept stirring at 80 °C for 12 h. After the reaction was completed, it was cooled to room temperature and filtered to remove the solids. The resulting filtrate was then concentrated in vacuo and purified by flash chromatography using mixtures of hexanes and ethyl acetate to afford methyl 7-methyl-4-(phenylethynyl)­benzofuran (**73**) (140 mg, 84% yield) as a white solid. ^1^H NMR (400 MHz, CDCl_3_) δ: 7.68 (d, *J* = 2.2 Hz, 1H), 7.61–7.54 (m, 2H), 7.51–7.27 (m, 5H), 7.11–7.01 (m, 1H), 6.98 (d, *J* = 2.2 Hz, 1H), 2.55 (s, 3H). ^13^C NMR (101 MHz, CDCl_3_) δ: 153.5, 145.1, 131.6, 131.5, 128.8, 128.7, 128.3, 128.3, 128.2, 126.3, 126.3, 125.1, 123.4, 122.5, 113.3, 106.6, 91.6, 87.3, 77.2, 15.2.

#### 2-Iodo-7-methyl-4-(phenylethynyl)­benzofuran (**74**)

To a solution of 7-methyl-4-(phenylethynyl)­benzofuran (**73**) (140 mg, 590 μmol) in THF (5 mL) was added *n*-BuLi (1.6 M, 460 μL, 740 μmol) at −78 °C. After stirring at the same temperature for 30 min, a solution of iodine (190 mg, 740 μmol) in THF (2 mL) was added dropwise. The mixture was warmed to room temperature gradually and stirred for 1.5 h. The reaction was diluted with ethyl acetate (20 mL). Excess I_2_ was reduced with a 10% Na_2_S_2_O_7_ solution. The organic layer was washed with brine, dried over MgSO_4_, and concentrated under reduced pressure. Purification by flash column chromatography afforded 2-iodo-7-methyl-4-(phenylethynyl)­benzofuran (**74**) (160 mg, 77% yield) as a white solid. ^1^H NMR (400 MHz, CDCl_3_) δ: 7.61–7.56 (m, 2H), 7.43–7.29 (m, 4H), 7.20 (s, 1H), 7.02 (dd, *J* = 7.7, 1.0 Hz, 1H), 2.55 (s, 3H). ^13^C NMR (101 MHz, CDCl_3_) δ: 156.9, 131.6, 130.5, 128.3, 128.3, 126.6, 125.1, 123.2, 121.8, 117.3, 111.9, 96.3, 92.1, 86.8, 15.1.

#### Methyl 4-(5-(7-Methyl-4-(phenylethynyl)­benzofuran-2-yl)-1*H*-pyrrol-2-yl)­benzoate (**75**)

2-Iodo-7-methyl-4-(phenylethynyl)­benzofuran (**74**) (160 mg, 460 μmol), methyl 4-(5-(4,4,5,5-tetramethyl-1,3,2-dioxaborolan-2-yl)-1*H*-pyrrol-2-yl)­benzoate (**59a**) (220 mg, 680 μmol), K_3_CO_3_ (130 mg, 910 μmol), and Pd­(amphos)­Cl_2_ (32 mg, 46 μmol) were dissolved in deoxygenated dioxane/water (10/1, 10 mL). The reaction mixture was stirred at 100 °C for 3 h under nitrogen. The solvent was evaporated, and the crude was then purified by flash chromatography using hexanes and ethyl acetate to afford methyl 4-(5-(7-methyl-4-(phenylethynyl)­benzofuran-2-yl)-1*H*-pyrrol-2-yl)­benzoate (**75**) (130 mg, 66% yield) as a yellow solid. ^1^H NMR (400 MHz, CDCl_3_) δ: 8.99 (s, 1H), 8.12–8.05 (m, 2H), 7.68–7.55 (m, 4H), 7.42–7.31 (m, 4H), 7.03 (d, *J* = 12.0 Hz, 2H), 6.84–6.73 (m, 2H), 3.94 (s, 3H), 2.61 (s, 3H). ^13^C NMR (101 MHz, CDCl_3_) δ: 131.6, 130.4, 128.4, 128.2, 125.0, 123.4, 121.7, 110.4, 110.1, 99.5, 52.1, 15.3.

#### 4-(5-(7-Methyl-4-(phenylethynyl)­benzofuran-2-yl)-1*H*-pyrrol-2-yl)­benzoic Acid (**34**)

The mixture of LiOH·H_2_O (190 mg, 4.5 mmol) and 4-(5-(7-methyl-4-(phenylethynyl)­benzofuran-2-yl)-1*H*-pyrrol-2-yl)­benzoate (**75**) (130 mg, 300 μmol) in THF (10 mL) and H_2_O (2 mL) was stirred at 40 °C for 18 h. The resulting mixture was cooled to room temperature and acidified with HCl (1M) to pH 1, followed by extraction with ethyl acetate. The organic layer was washed with brine and dried over anhydrous MgSO_4_, then purified by flash chromatography using mixtures of hexanes and ethyl acetate to afford 4-(5-(7-methyl-4-(phenylethynyl)­benzofuran-2-yl)-1*H*-pyrrol-2-yl)­benzoic acid (**34**) (110 mg, 88% yield) as a yellow solid. ^1^H NMR (400 MHz, DMSO-*d*_6_) δ: 12.88 (s, 1H), 11.93 (t, *J* = 2.5 Hz, 1H), 8.02–7.96 (m, 2H), 7.96–7.89 (m, 2H), 7.68–7.64 (m, 2H), 7.55–7.39 (m, 4H), 7.34 (d, *J* = 7.7 Hz, 1H), 7.12 (dd, *J* = 7.6, 0.9 Hz, 1H), 6.89 (dd, *J* = 3.8, 2.4 Hz, 1H), 6.83 (dd, *J* = 3.8, 2.2 Hz, 1H), 2.55 (d, *J* = 5.0 Hz, 3H). ^13^C NMR (101 MHz, DMSO-*d*_6_) δ: 167.5, 152.5, 150.9, 136.2, 134.0, 131.8, 131.0, 130.3, 129.3, 129.2, 128.5, 126.8, 125.4, 125.4, 124.2, 122.9, 122.1, 111.7, 110.8, 110.2, 99.3, 92.2, 88.0, 15.2. *m*/*z* (ES^+^) [M + H]^+^ = 418.1.

## Supplementary Material









## Notes

Purity of compounds determined by UPLC MS (PDF)
Docking studies of SR200931A (PDB)
Docking studies of SR201119B (PDB)
Molecular formula strings of the compounds (CSV)

## Abbreviations USED

ADMET: absorption, distribution, metabolism, excretion, and Toxicity
ATRA: all-trans retinoic acid
Bu: butyl
CDT: Cessation of Drug Treatment
DAN: 1,5-diaminonaphthalene
DCE: 1,2-dichloroethane
DMAU: dimethandrolone undecanoate
DMF: dimethylformamide
DMSO: dimethyl sulfoxide
DNA: DNA
E: eosin
EC_30_: 30% maximal effective concentration
EC_50_: half maximal effective concentration
Et: ethyl
FRET: fluorescence resonance energy transfer
H: hematoxylin
HPLC: high-performance liquid chromatography
IC_50_: half maximal inhibitory concentration
ITO: indium tin oxide
LBD: ligand-binding domain
LC: liquid chromatography
*m*/*z*: mass-to-charge
MALDI-IMS: matrix-assisted laser desorption/ionization imaging mass spectrometry
Me: methyl
MS: mass spectrometry
NMR: nuclear magnetic resonance
PBS: phosphate-buffered saline
PDB: protein data bank
Ph: phenyl
PK: pharmacokinetic
RA: retinoic acid
RAR: retinoic acid receptor
RARα: retinoic acid receptor alpha
RARβ: retinoic acid receptor beta
RARγ: retinoic acid receptor gamma
SAR: structure–activity relationship
SEM: standard error of the mean
*T*_1/2_: half-life
THF: tetrahydrofuran
TLC: thin-layer chromatography
UPLC: ultraperformance liquid chromatography
UV: ultraviolet
