# Supplementary material for: Development of a Second-Generation RARα Selective Antagonist as an Orally Bioavailable, Effective, Safe, and Reversible Male Contraceptive
Source: J Med Chem. 2026 May 11;69(10):12144–70. doi: 10.1021/acs.jmedchem.6c00011 (PMC13224095; doi:10.1021/acs.jmedchem.6c00011)

Development of a Second-generation RARα Selective Antagonist

as an Orally Bioavailable, Effective, Safe, and Reversible Male Contraceptive

Rui Shi,[a] Kristen John,[a] Xuan Qin,[b] Ehfazul Haque,[a] Taimeng Liang,[c]

Narsihmulu Cheryala,[a] Feng Li,[b] Henry L. Wong,[a] and Gunda I. Georg[a*]

[a]*Department of Medicinal Chemistry and Institute for Therapeutics Discovery & Development, College of Pharmacy, University of Minnesota, 717 Delaware Street SE, Minneapolis, Minnesota 55414, USA*

[b]*Center for Drug Discovery, Department of Pathology and Immunology, NMR and Drug Metabolism Core, Advanced Technology Cores, Department of Biochemistry and Molecular Pharmacology, Baylor College of Medicine, One Baylor Plaza, MS: BCM330, Houston, Texas, 77030, USA*

[c]*Department of Chemistry, University of Minnesota, 207 Pleasant Street, SE, Minneapolis, MN 55455-0431, USA*

Email for Corresponding author: georg@umn.edu

Table of Contents

HPLC traces for all tested compounds Pages 2-137

Mass Analysis Report

SAMPLE INFORMATION

Sample Name: SR200831A
Acq Method Set: Col2_MeCN_H2O_NH4HCO3
Acquired: 1/29/2021 10:44:31 PM CST
InjVol: 7.50 uL

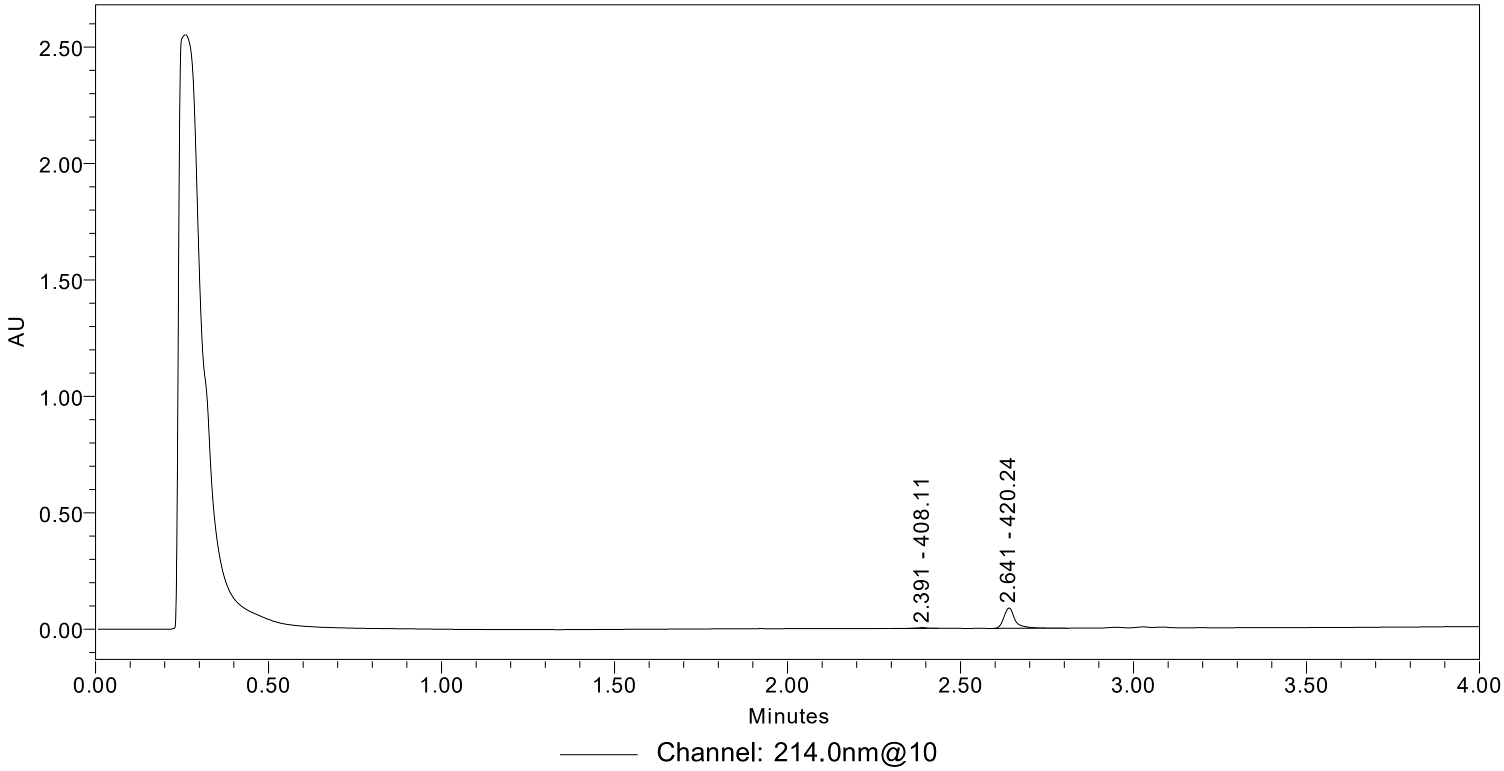

	RT	Area	% Area	Height	Base Peak (m/z)
1	2.391	7157	3.49	2784	408.11
2	2.641	198068	96.51	87075	420.24

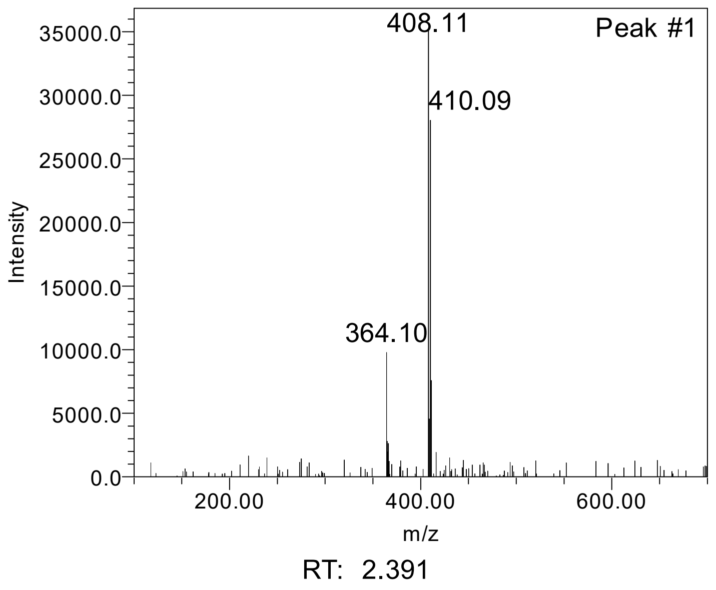

Peak #1
408.11
410.09
364.10
Intensity
35000.0
30000.0
25000.0
20000.0
15000.0
10000.0
5000.0
0.0
200.00
400.00
600.00
m/z
RT: 2.391

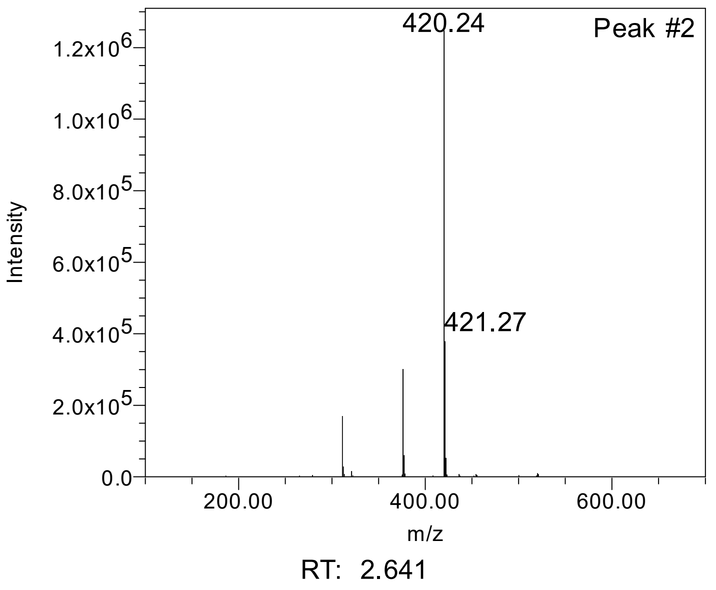

Peak #2
420.24
421.27
Intensity
1.2x10^6
1.0x10^6
8.0x10^5
6.0x10^5
4.0x10^5
2.0x10^5
0.0
200.00
400.00
600.00
m/z
RT: 2.641

Mass Analysis Report

SAMPLE INFORMATION

Sample Name: SR200831A
Acq Method Set: Col1_MeOH_H2O_NH4HCO3
Acquired: 1/30/2021 8:34:36 AM CST
InjVol: 7.50 uL

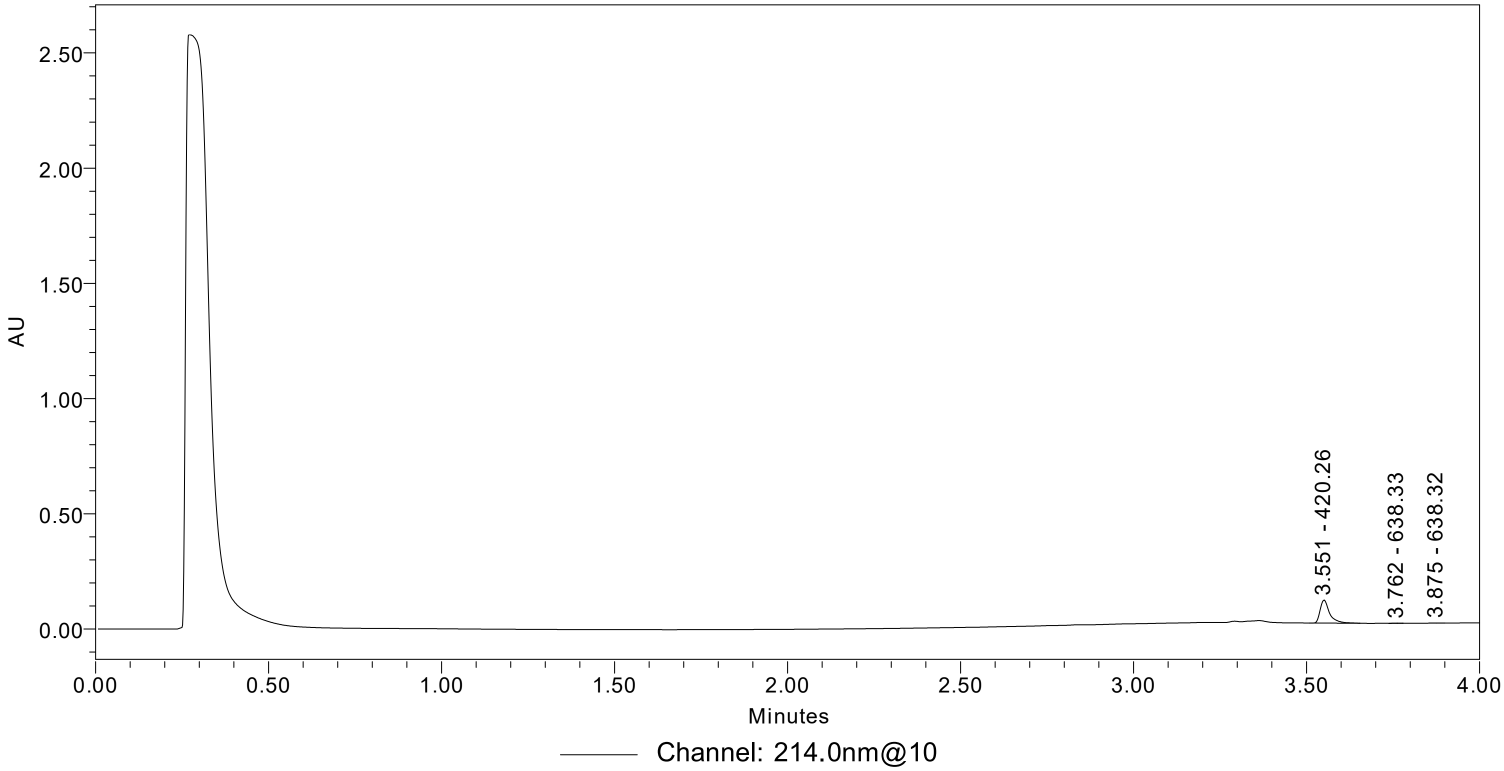

	RT	Area	% Area	Height	Base Peak (m/z)
1	3.551	190571	99.61	99586	420.26
2	3.762	320	0.17	335	638.33
3	3.875	433	0.23	407	638.32

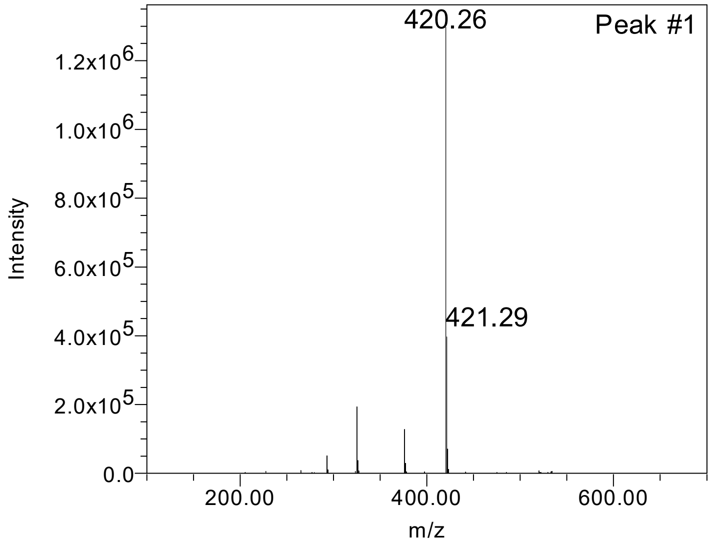

RT: 3.551

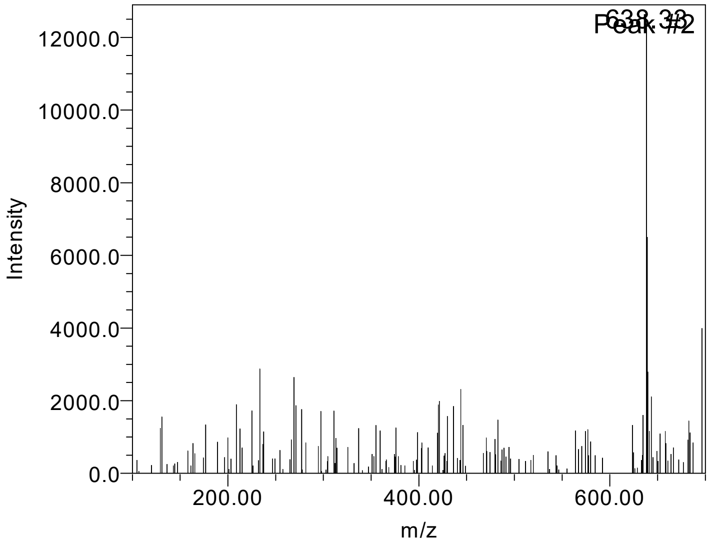

RT: 3.762

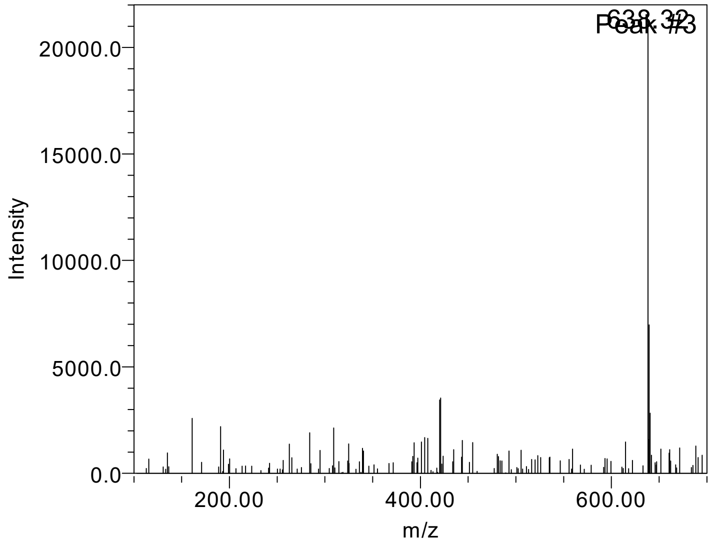

RT: 3.875

Mass Analysis Report

SAMPLE INFORMATION

Sample Name: SR200904B
Acq Method Set: Col2_MeCN_H2O_NH4HCO3
Acquired: 1/29/2021 10:51:36 PM CST
InjVol: 7.50 uL

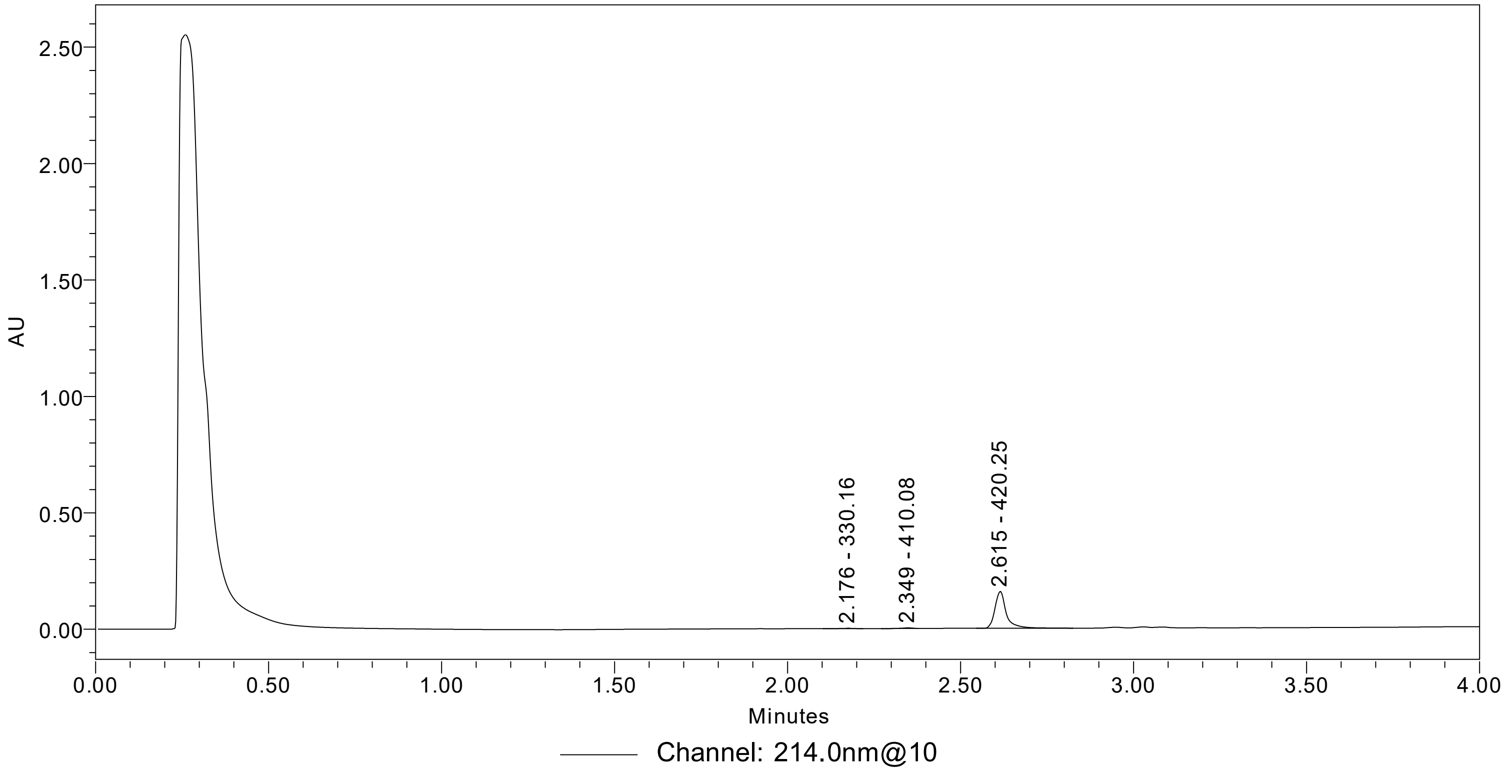

	RT	Area	% Area	Height	Base Peak (m/z)
1	2.176	3128	0.85	1271	330.16
2	2.349	5254	1.42	2521	410.08
3	2.615	360464	97.73	157778	420.25

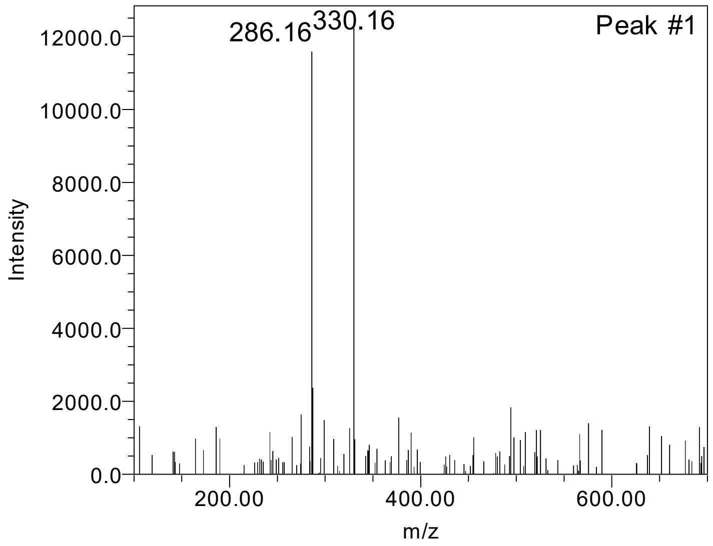

RT: 2.176

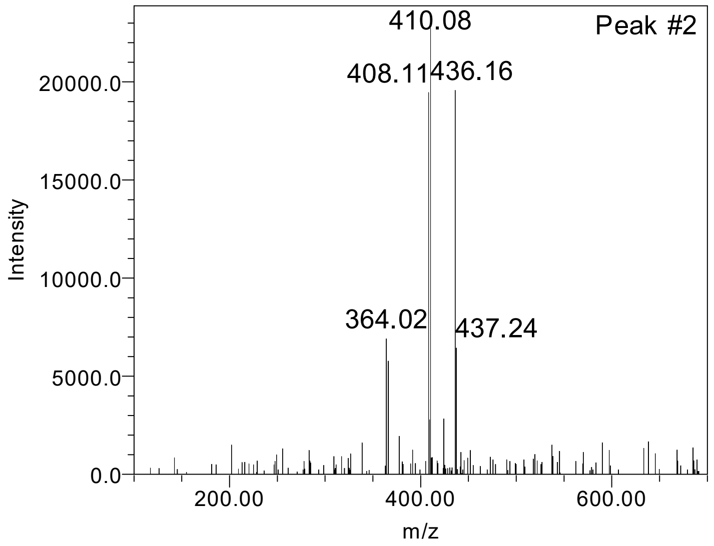

RT: 2.349

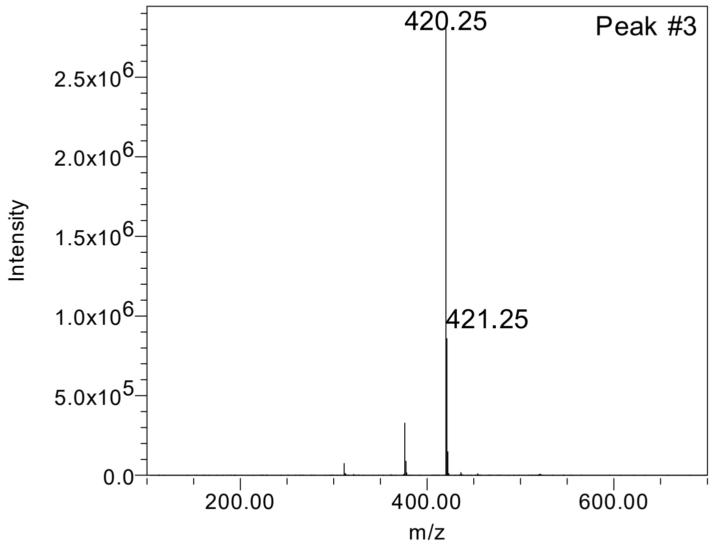

RT: 2.615

Mass Analysis Report

SAMPLE INFORMATION

Sample Name: SR200904B
Acq Method Set: Col1_MeOH_H2O_NH4HCO3
Acquired: 1/30/2021 8:41:41 AM CST
InjVol: 7.50 uL

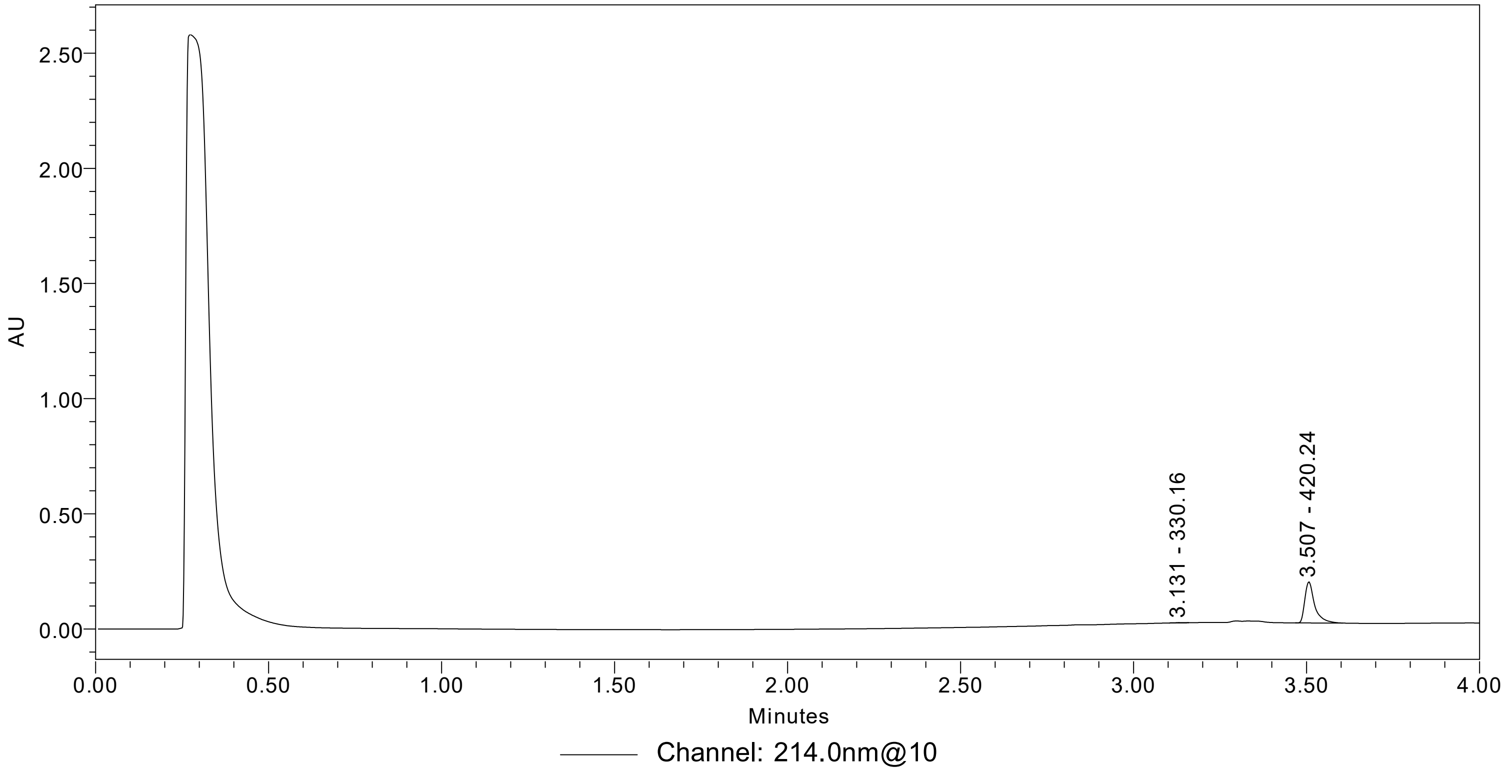

	RT	Area	% Area	Height	Base Peak (m/z)
1	3.131	1185	0.33	707	330.16
2	3.507	353100	99.67	177650	420.24

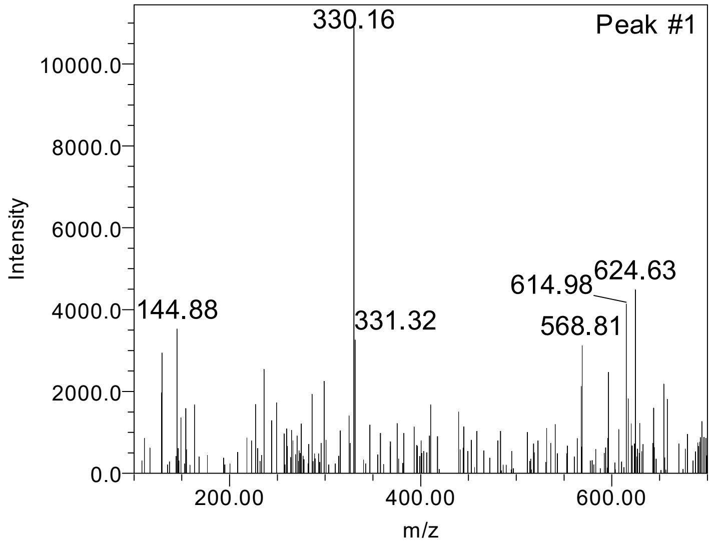

RT: 3.131

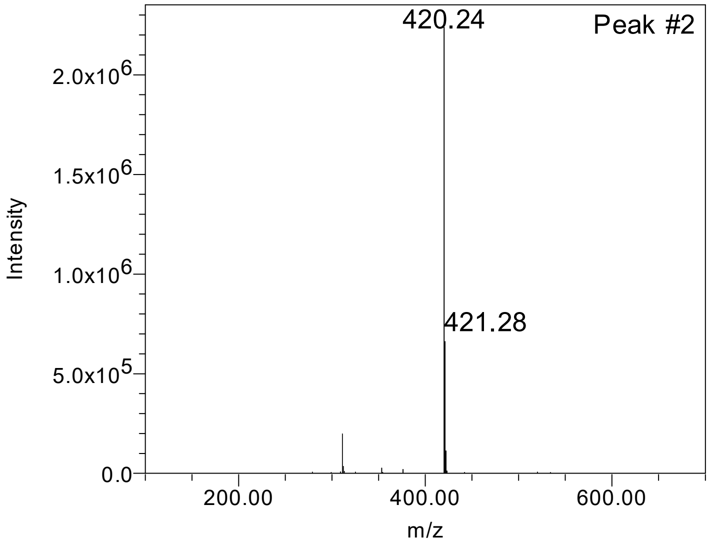

RT: 3.507

Mass Analysis Report

SAMPLE INFORMATION

Sample Name: SR201229
Acq Method Set: Col2_MeCN_H2O_NH4HCO3
Acquired: 1/29/2021 11:27:08 PM CST
InjVol: 7.50 uL

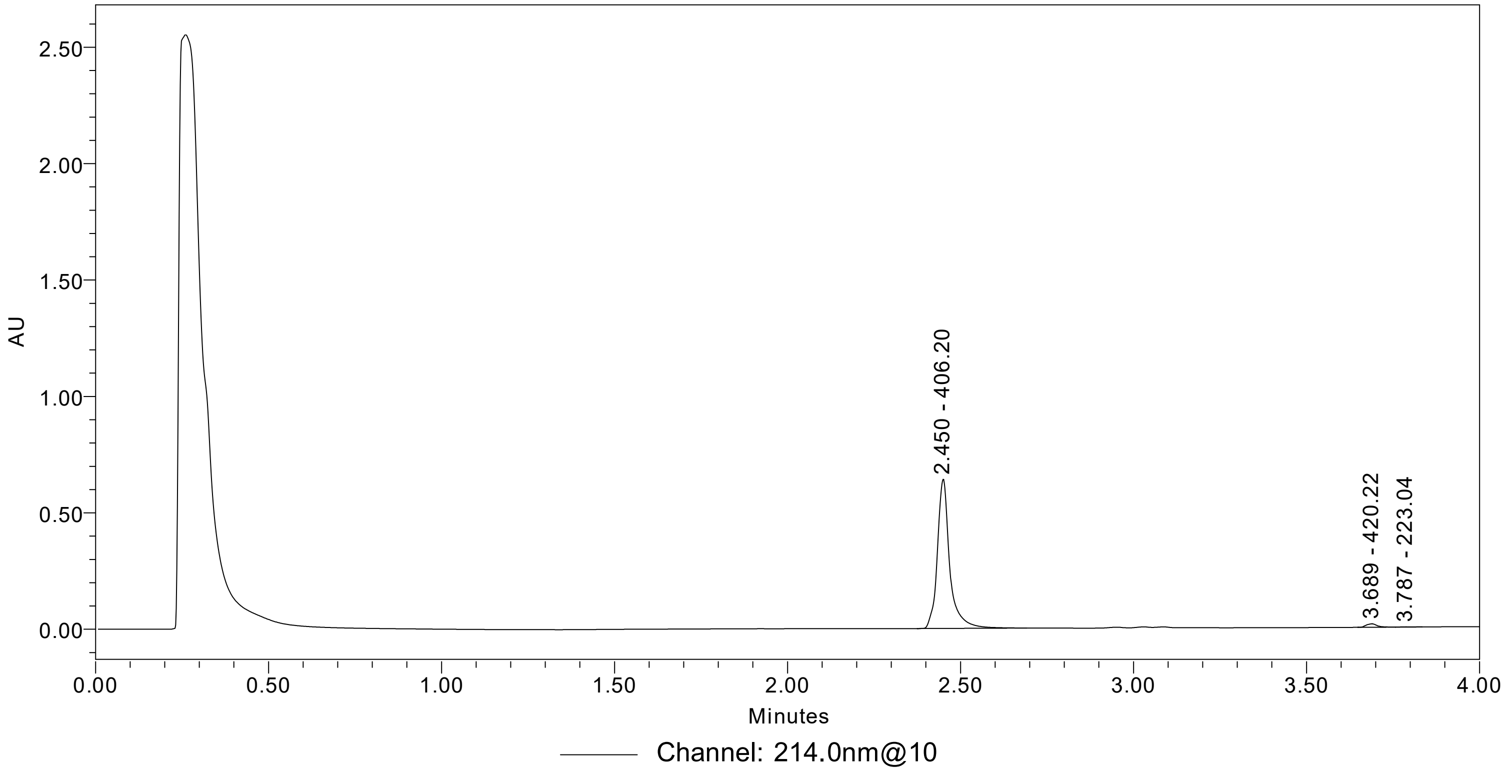

	RT	Area	% Area	Height	Base Peak (m/z)
1	2.450	1593985	97.89	640634	406.20
2	3.689	32874	2.02	14978	420.22
3	3.787	1476	0.09	760	223.04

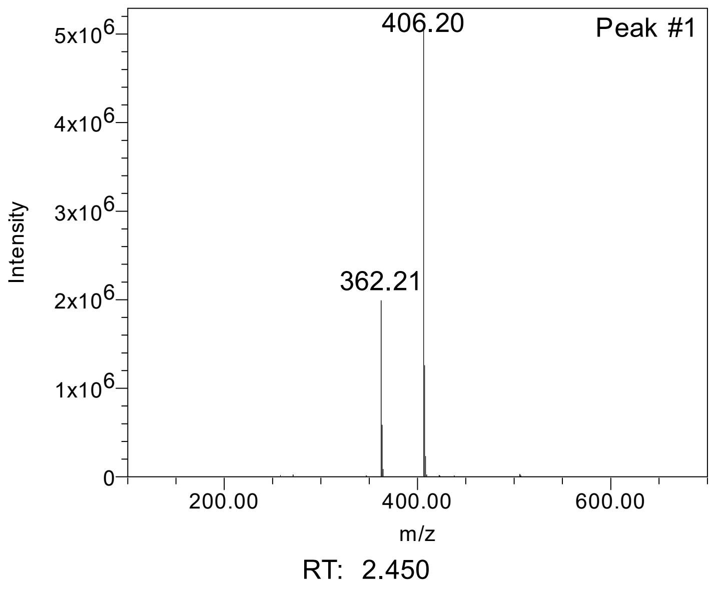
Peak #1
406.20
362.21
Intensity
m/z
RT: 2.450

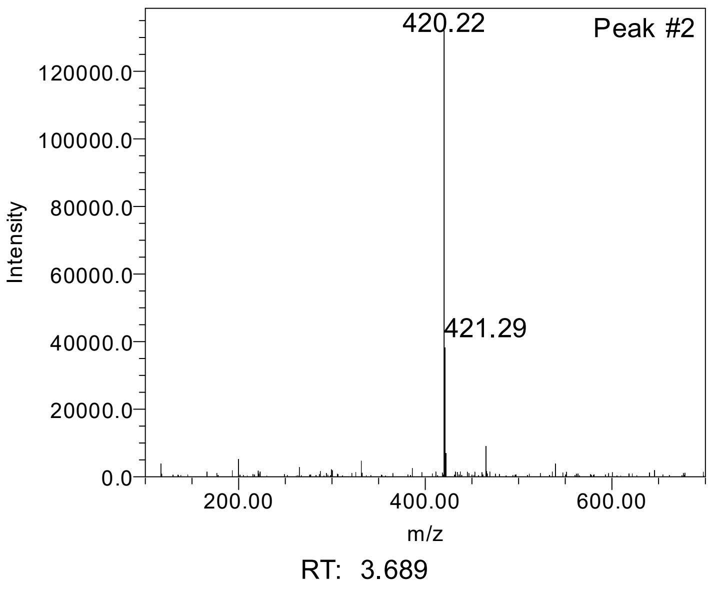
Peak #2
420.22
421.29
Intensity
m/z
RT: 3.689

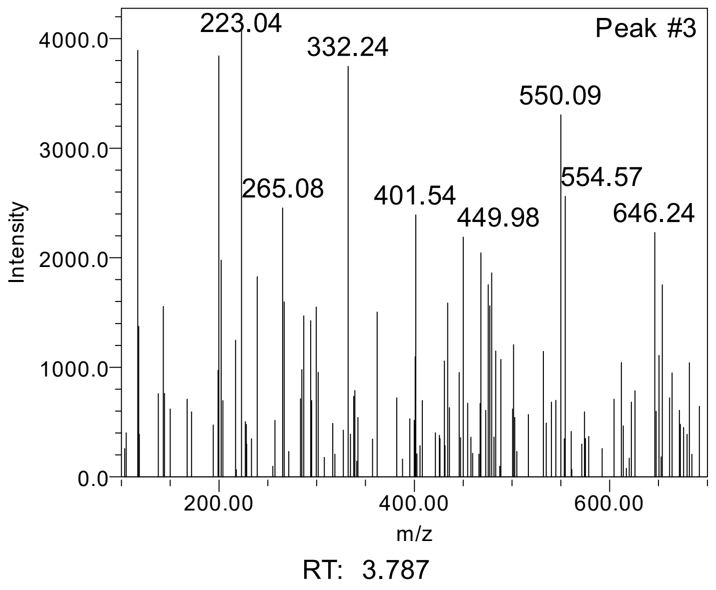
Peak #3
223.04
332.24
550.09
265.08
401.54
449.98
554.57
646.24
Intensity
m/z
RT: 3.787

Mass Analysis Report

SAMPLE INFORMATION

Sample Name: SR201229
Acq Method Set: Col1_MeOH_H2O_NH4HCO3

Acquired: 1/30/2021 9:17:13 AM CST
InjVol: 7.50 uL

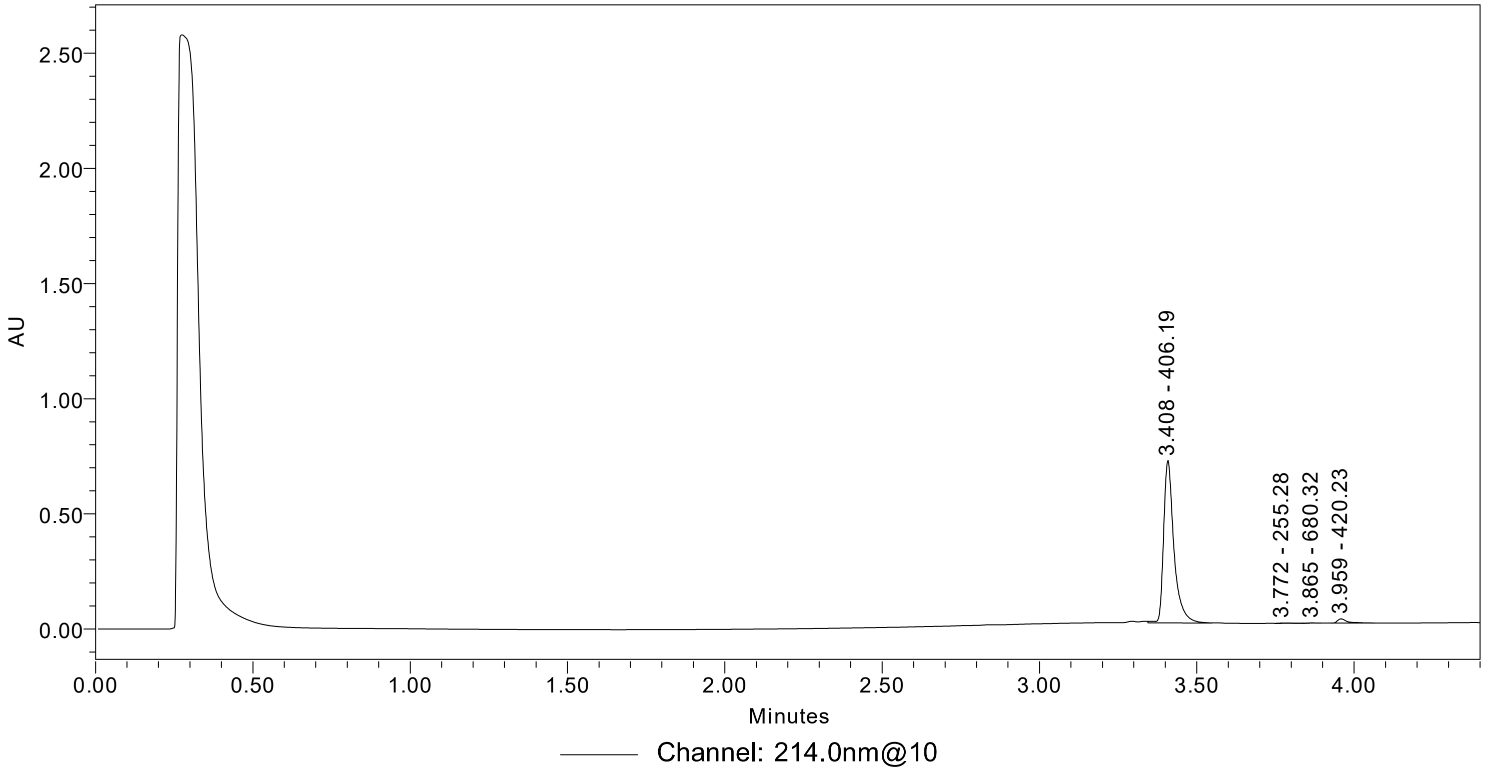

	RT	Area	% Area	Height	Base Peak (m/z)
1	3.408	1610069	97.46	704384	406.19
2	3.772	3033	0.18	1079	255.28
3	3.865	1437	0.09	1118	680.32
4	3.959	37442	2.27	17848	420.23

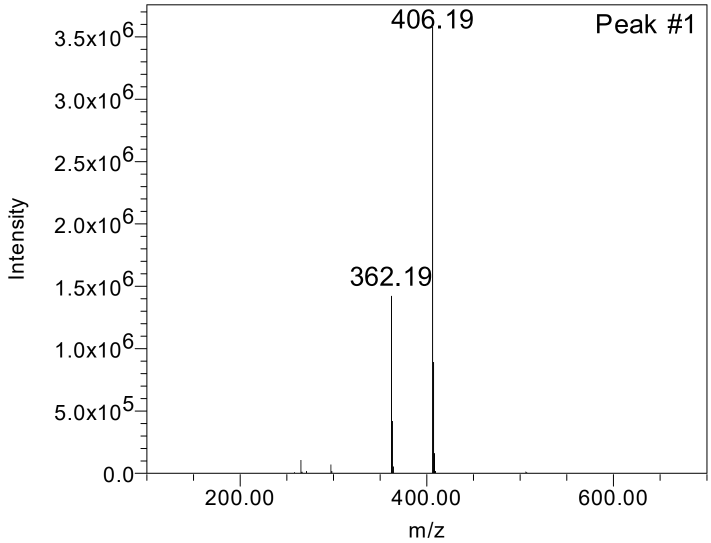

RT: 3.408

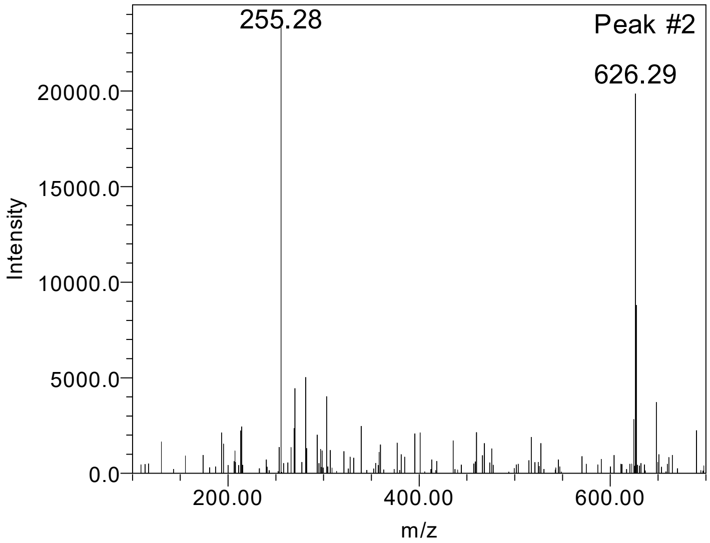

RT: 3.772

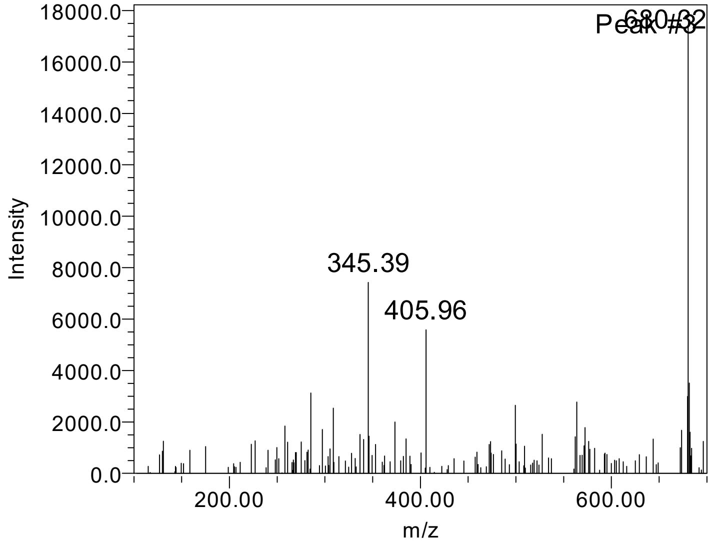

RT: 3.865

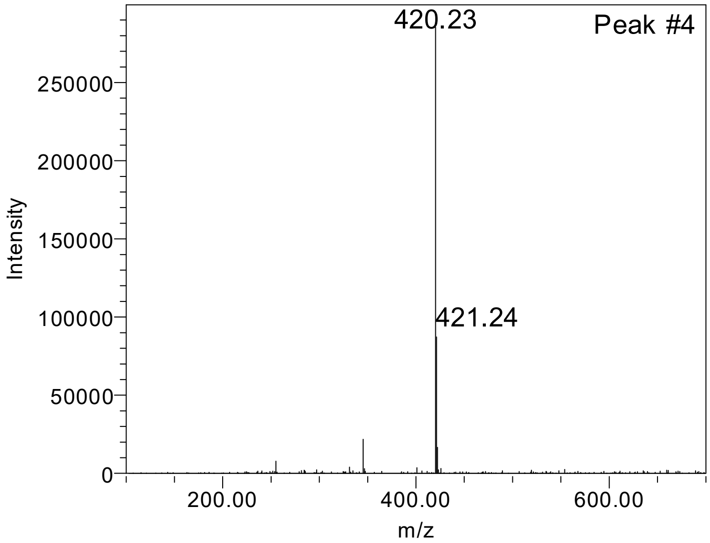

RT: 3.959

Mass Analysis Report

SAMPLE INFORMATION

Sample Name: SR211011C
Acq Method Set: Col2_MeCN_H2O_NH4HCO3
Acquired: 11/11/2021 12:59:47 PM CST
InjVol: 3.00 uL

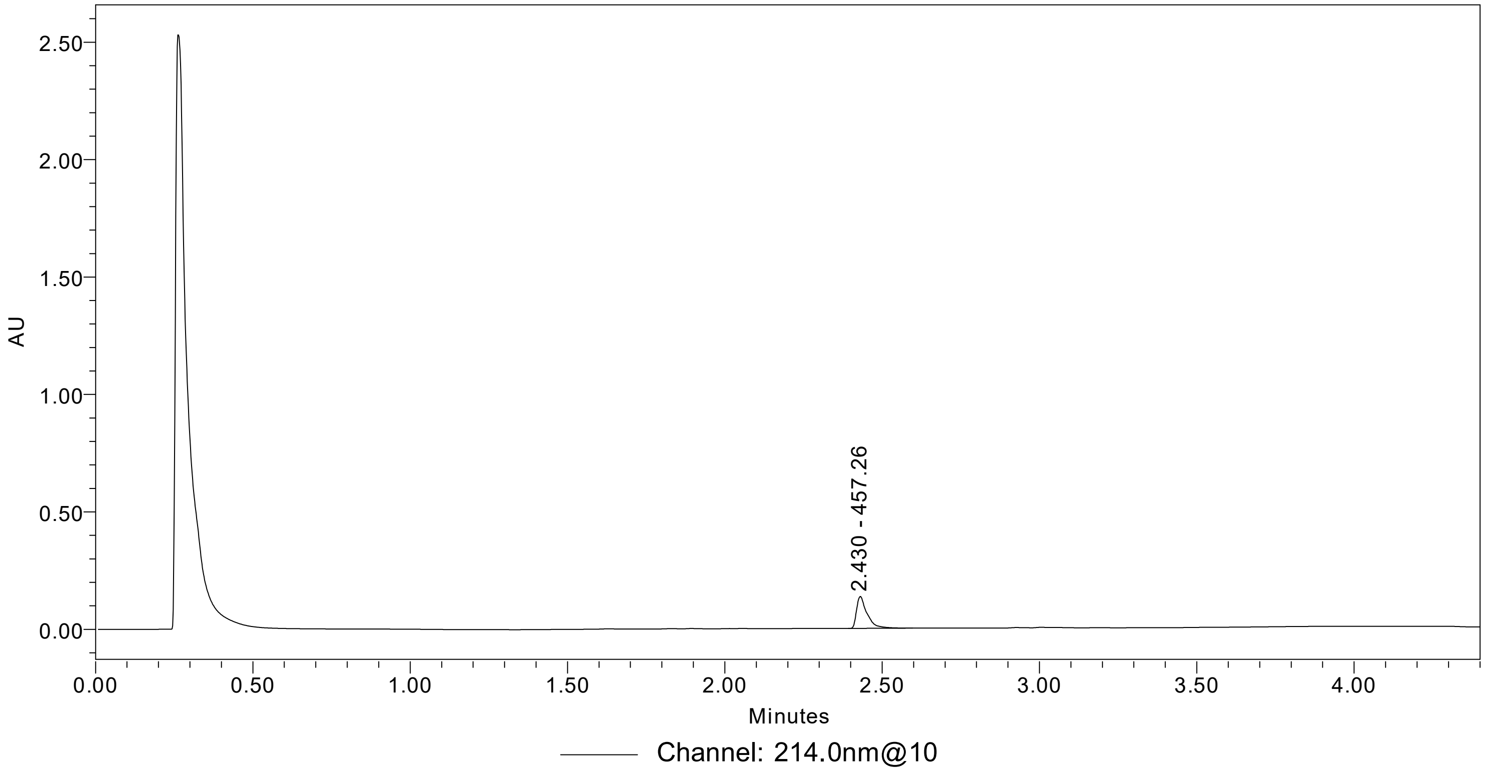

	RT	Area	% Area	Height	Base Peak (m/z)
1	2.430	314982	100.00	135807	457.26

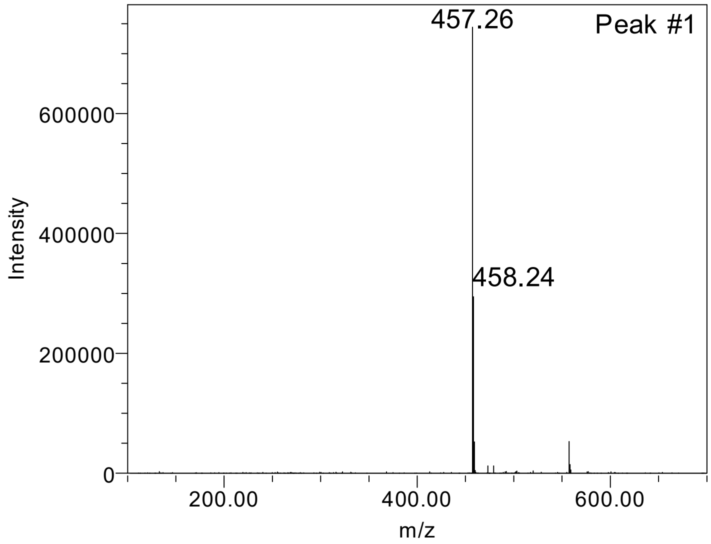

RT: 2.430

Mass Analysis Report

SAMPLE INFORMATION

Sample Name: SR211011C
Acq Method Set: Col1_MeOH_H2O_NH4HCO3
Acquired: 11/11/2021 4:50:41 PM CST
InjVol: 3.00 uL

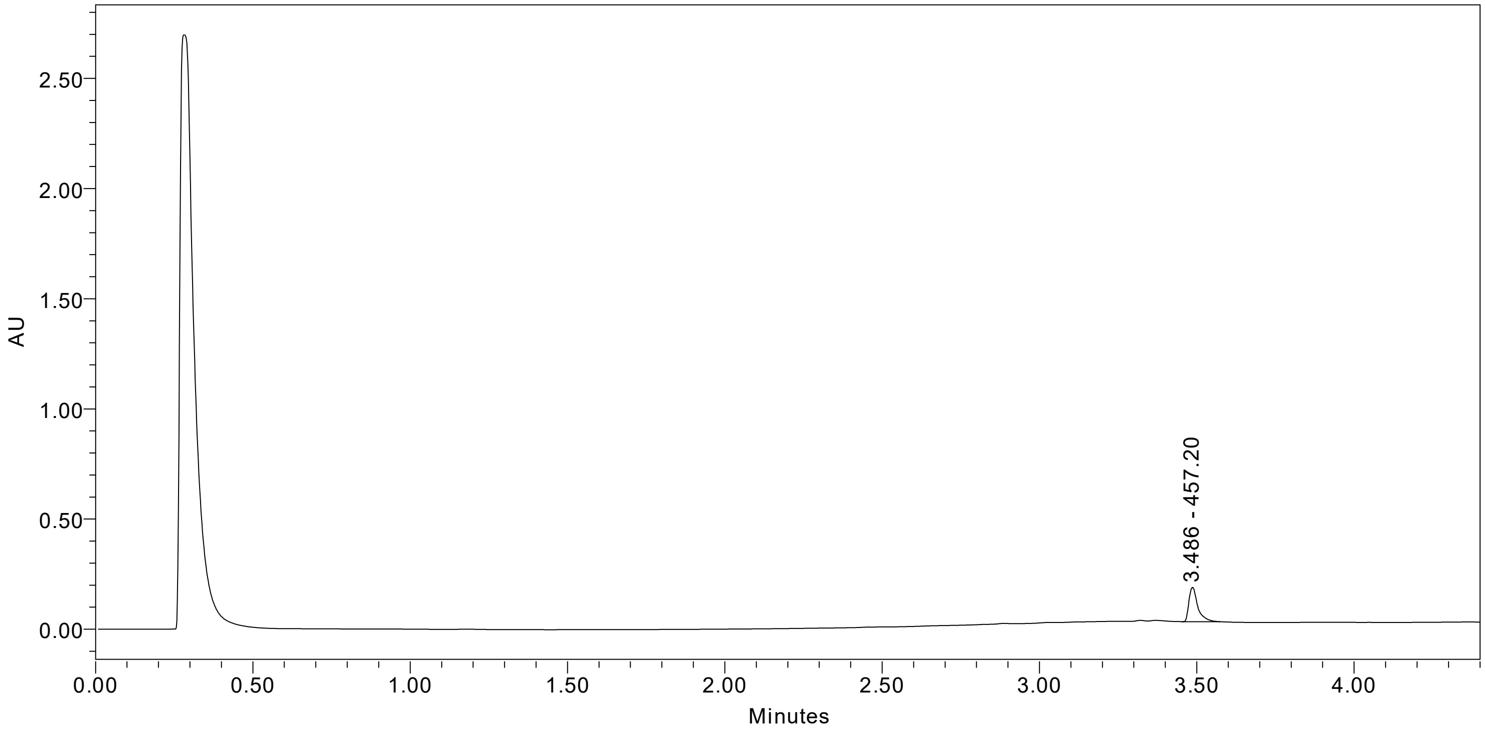

	RT	Area	% Area	Height	Base Peak (m/z)
1	3.486	303148	100.00	155294	457.20

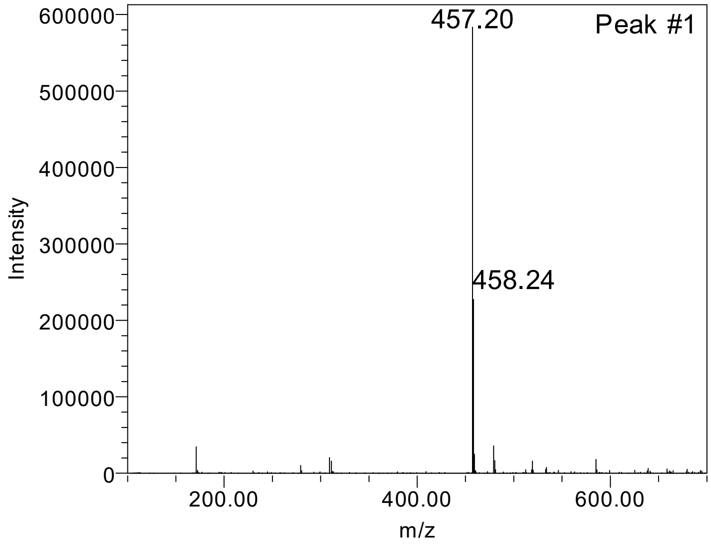

RT: 3.486

Mass Analysis Report

SAMPLE INFORMATION

Sample Name: SR211011A
Acq Method Set: Col2_MeCN_H2O_NH4HCO3
Acquired: 11/11/2021 12:52:47 PM CST
InjVol: 3.00 uL

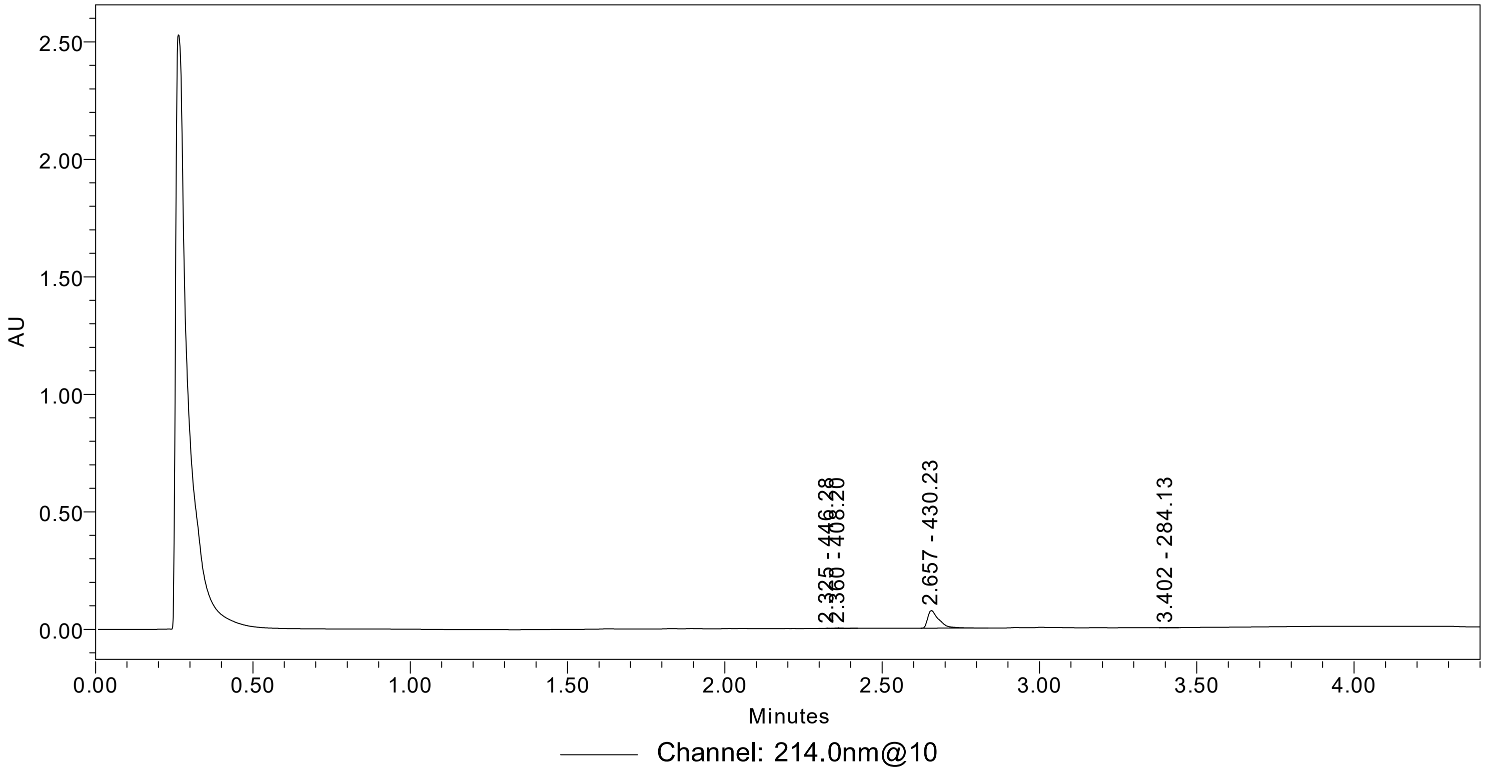

	RT	Area	% Area	Height	Base Peak (m/z)
1	2.325	778	0.41	614	446.28
2	2.360	4063	2.12	1911	408.20
3	2.657	186272	97.24	74718	430.23
4	3.402	450	0.23	274	284.13

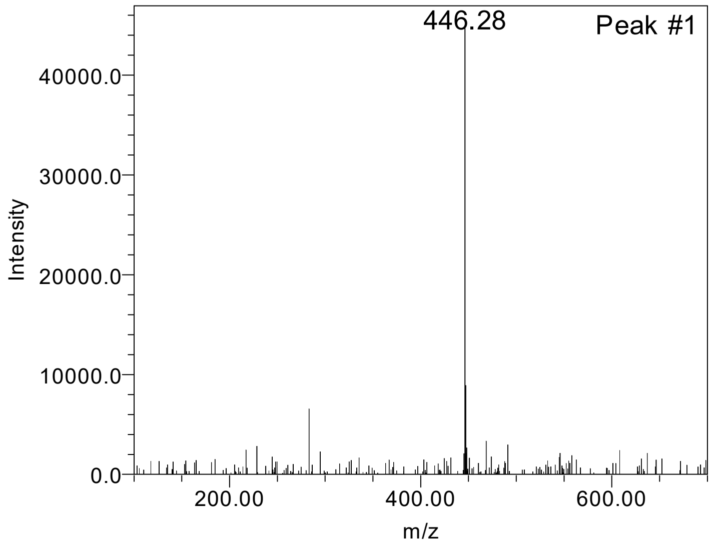

RT: 2.325

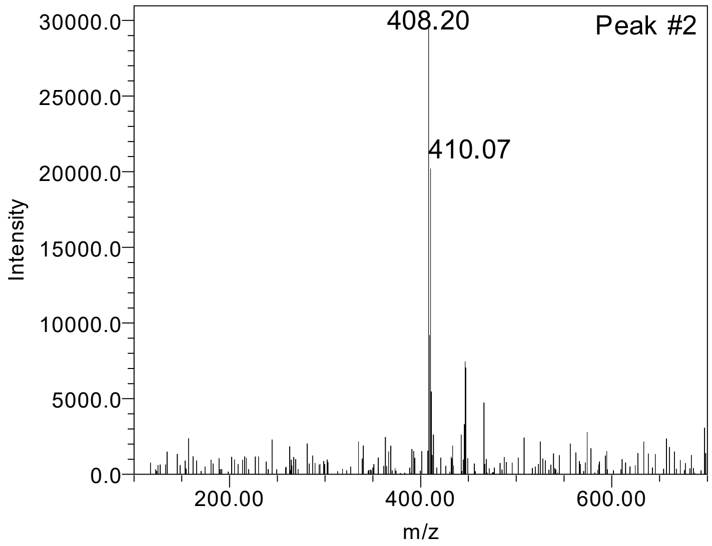

RT: 2.360

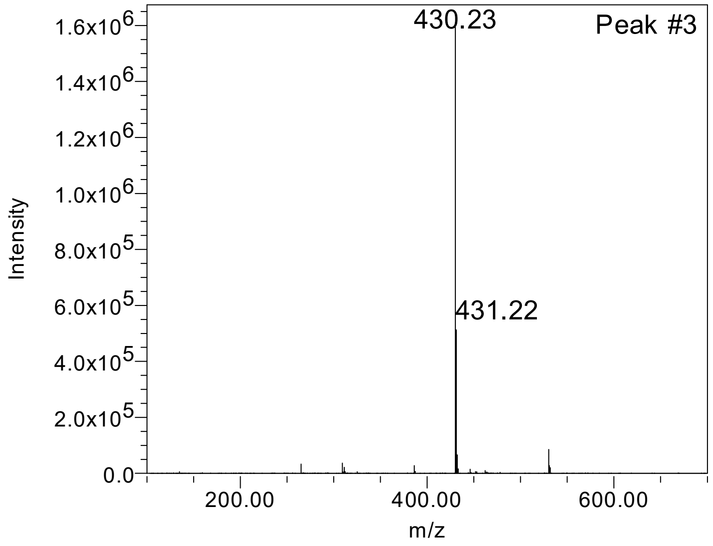

RT: 2.657

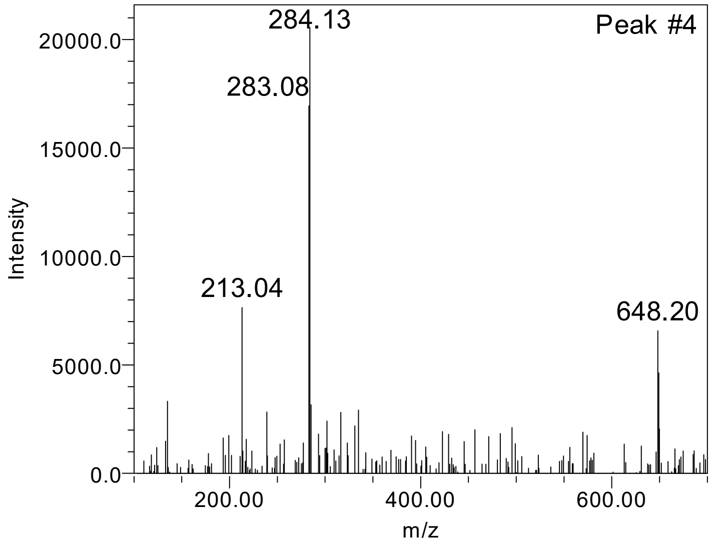

RT: 3.402

Mass Analysis Report

SAMPLE INFORMATION

Sample Name: SR211011A
Acq Method Set: Col1_MeOH_H2O_NH4HCO3
Acquired: 11/11/2021 4:43:39 PM CST
InjVol: 3.00 uL

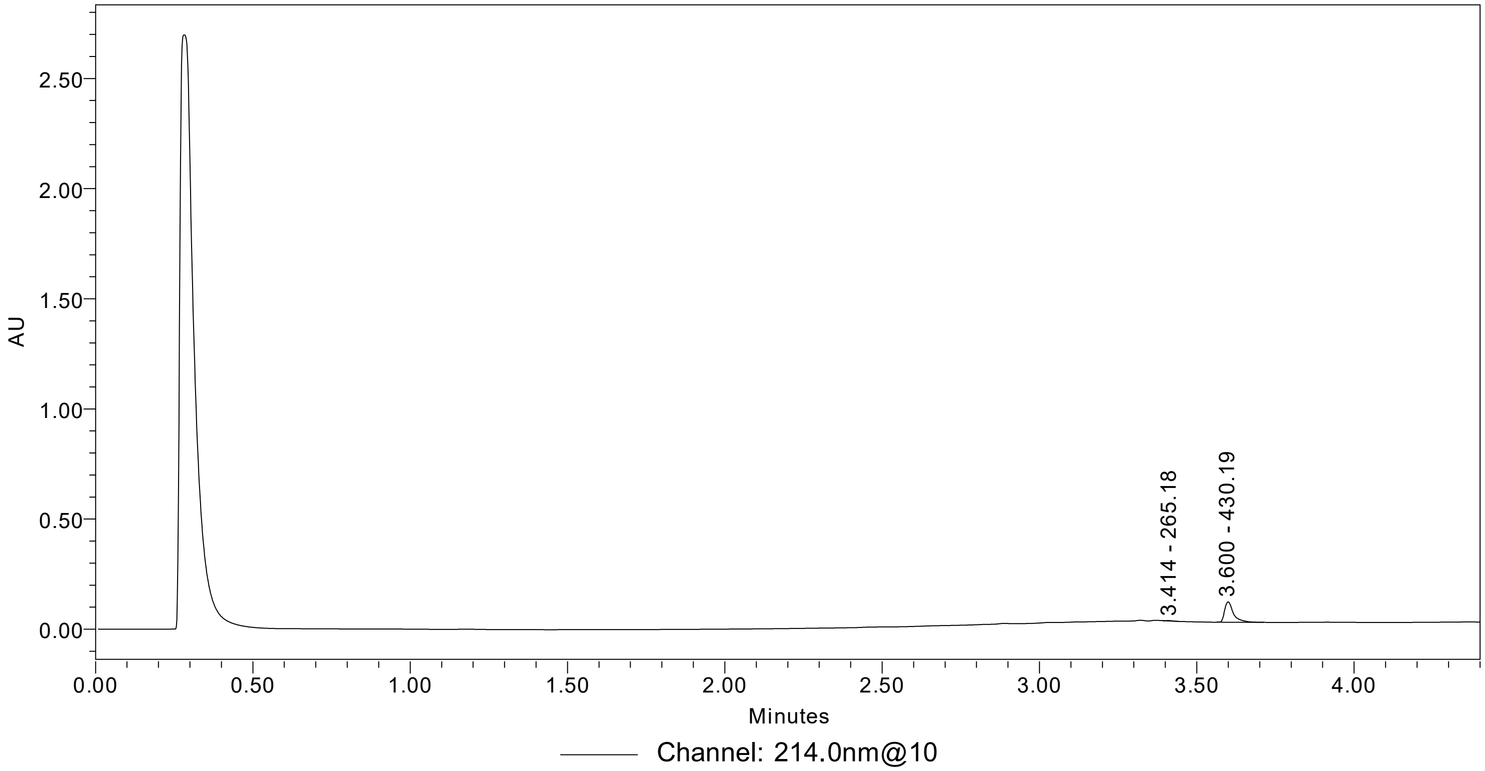

	RT	Area	% Area	Height	Base Peak (m/z)
1	3.414	1902	1.04	1425	265.18
2	3.600	180705	98.96	91935	430.19

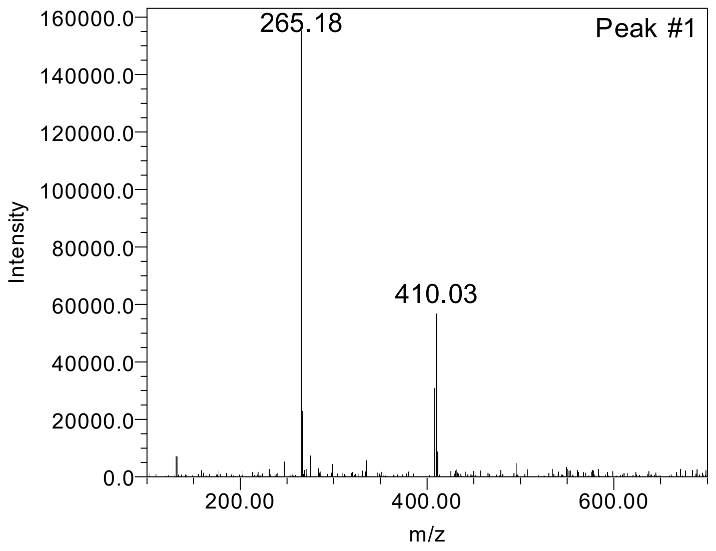

RT: 3.414

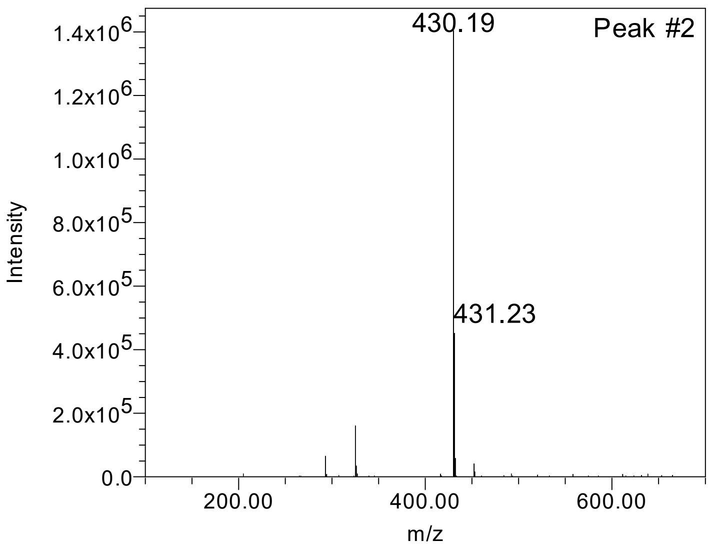

RT: 3.600

Mass Analysis Report

SAMPLE INFORMATION

Sample Name: SR211205A
Acq Method Set: Col2_MeCN_H2O_NH4HCO3
Acquired: 12/21/2021 8:13:05 PM CST
InjVol: 7.50 uL

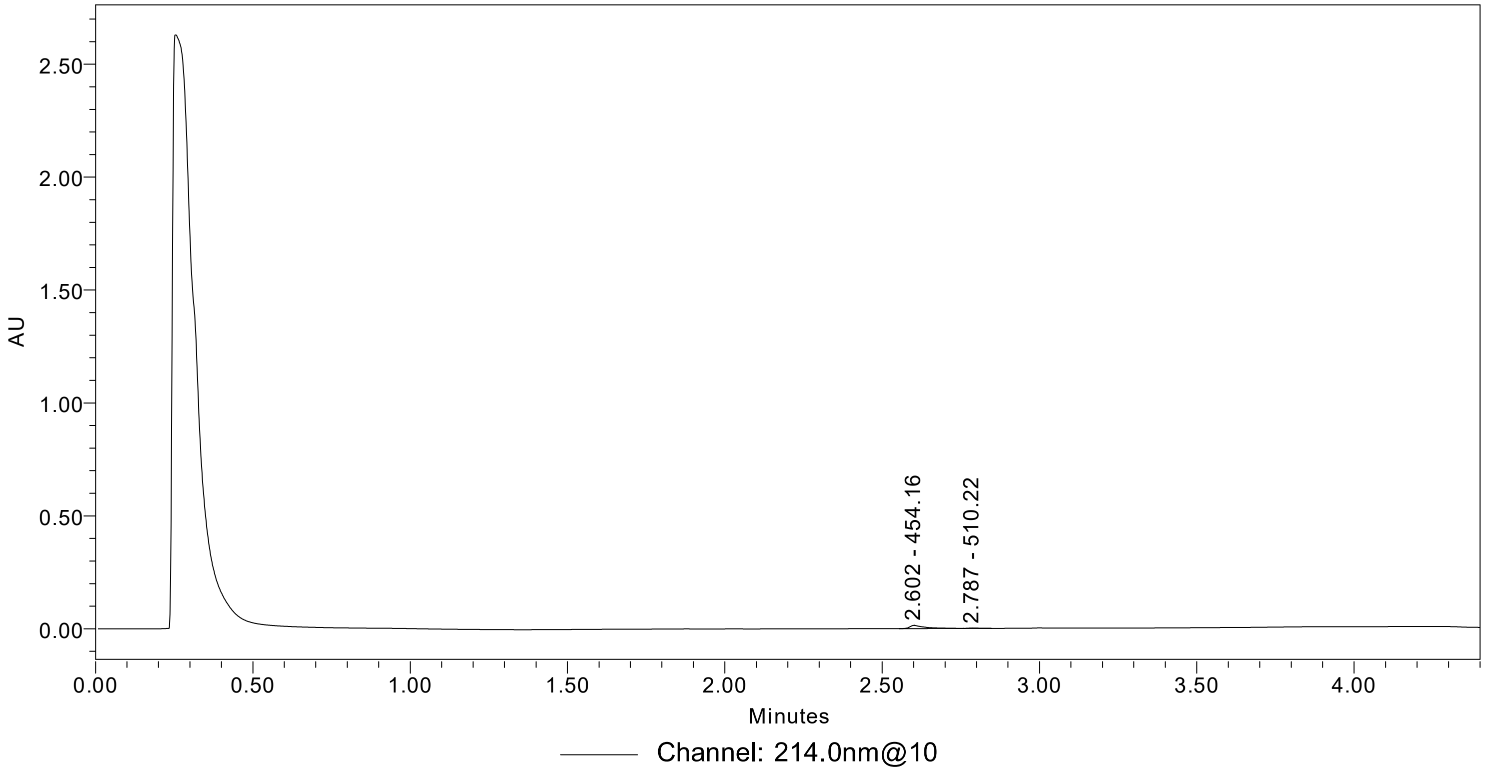

	RT	Area	% Area	Height	Base Peak (m/z)
1	2.602	47437	92.08	14169	454.16
2	2.787	4080	7.92	1744	510.22

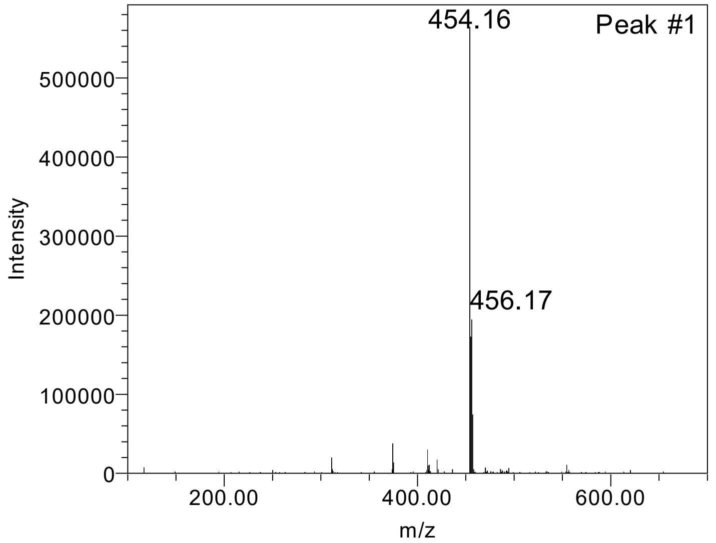

RT: 2.602

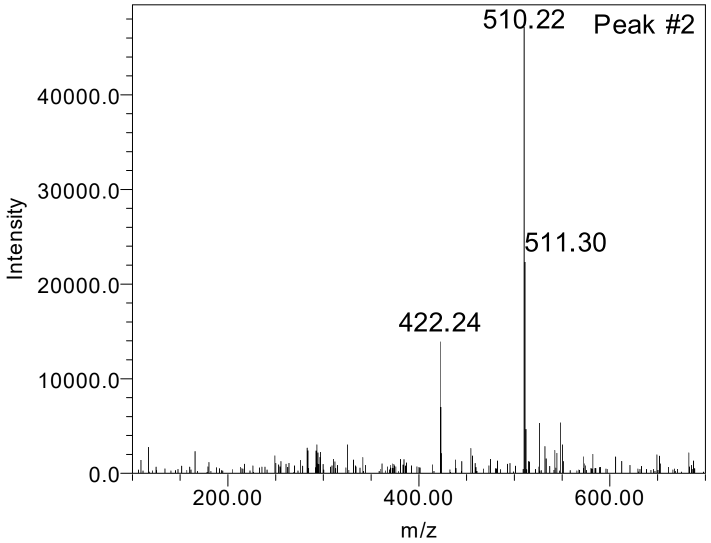

RT: 2.787

Mass Analysis Report

SAMPLE INFORMATION

Sample Name: SR211205A
Acq Method Set: Col1_MeOH_H2O_NH4HCO3
Acquired: 12/22/2021 2:59:11 AM CST
InjVol: 7.50 uL

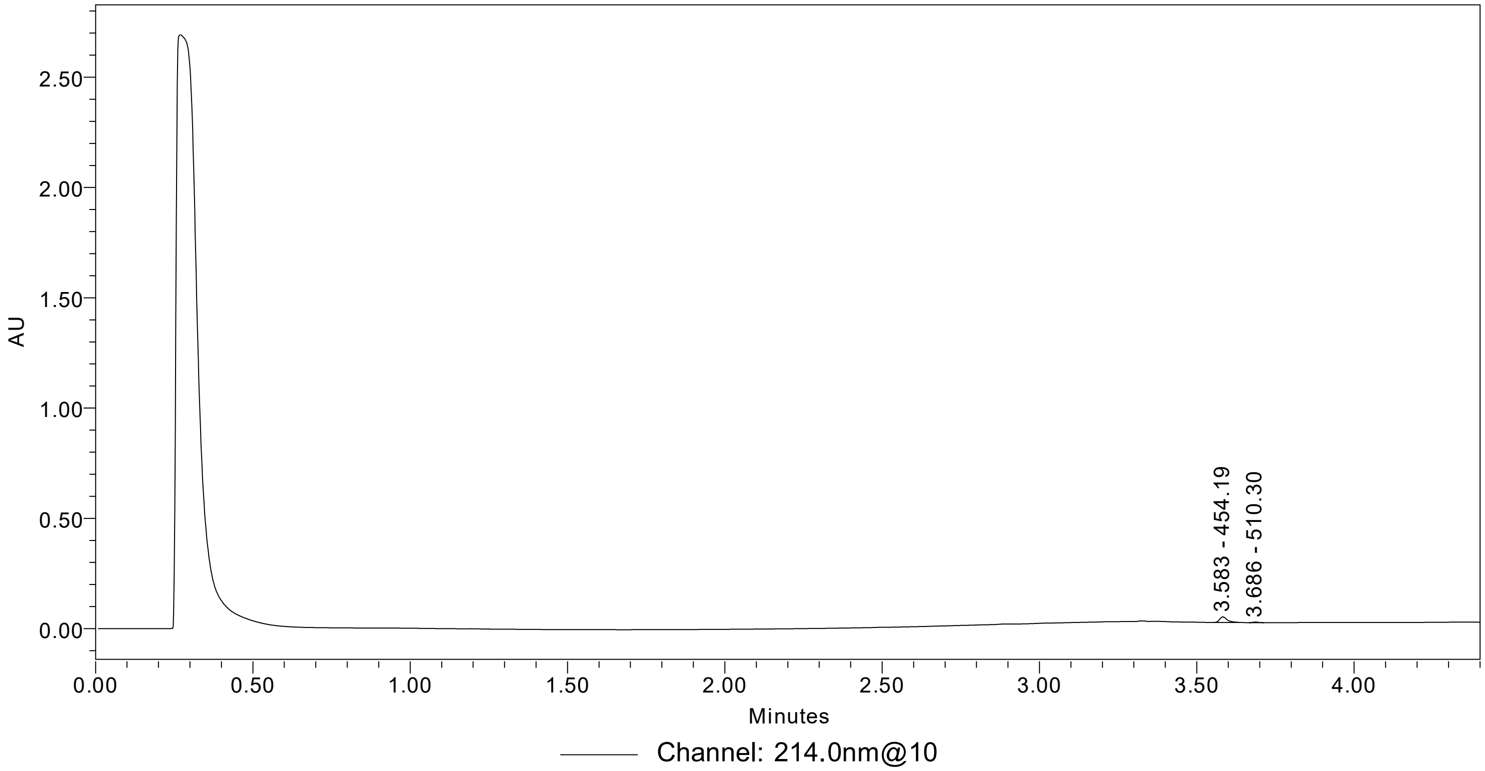

	RT	Area	% Area	Height	Base Peak (m/z)
1	3.583	43255	92.96	25296	454.19
2	3.686	3276	7.04	2629	510.30

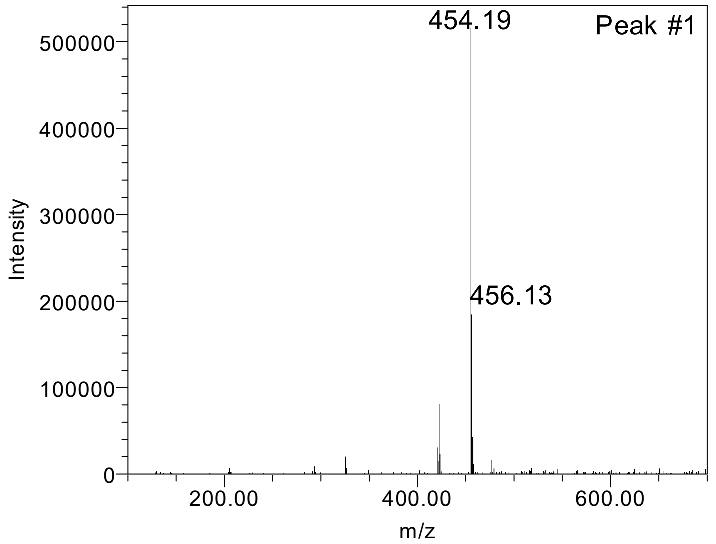

RT: 3.583

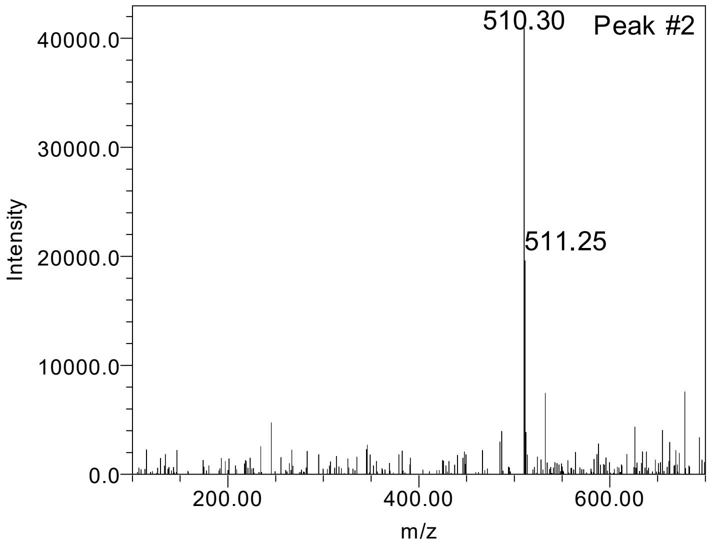

RT: 3.686

Mass Analysis Report

SAMPLE INFORMATION

Sample Name: SR210620A
Acq Method Set: Col2_MeCN_H2O_NH4HCO3
Acquired: 6/24/2021 7:21:39 PM CDT
InjVol: 7.50 uL

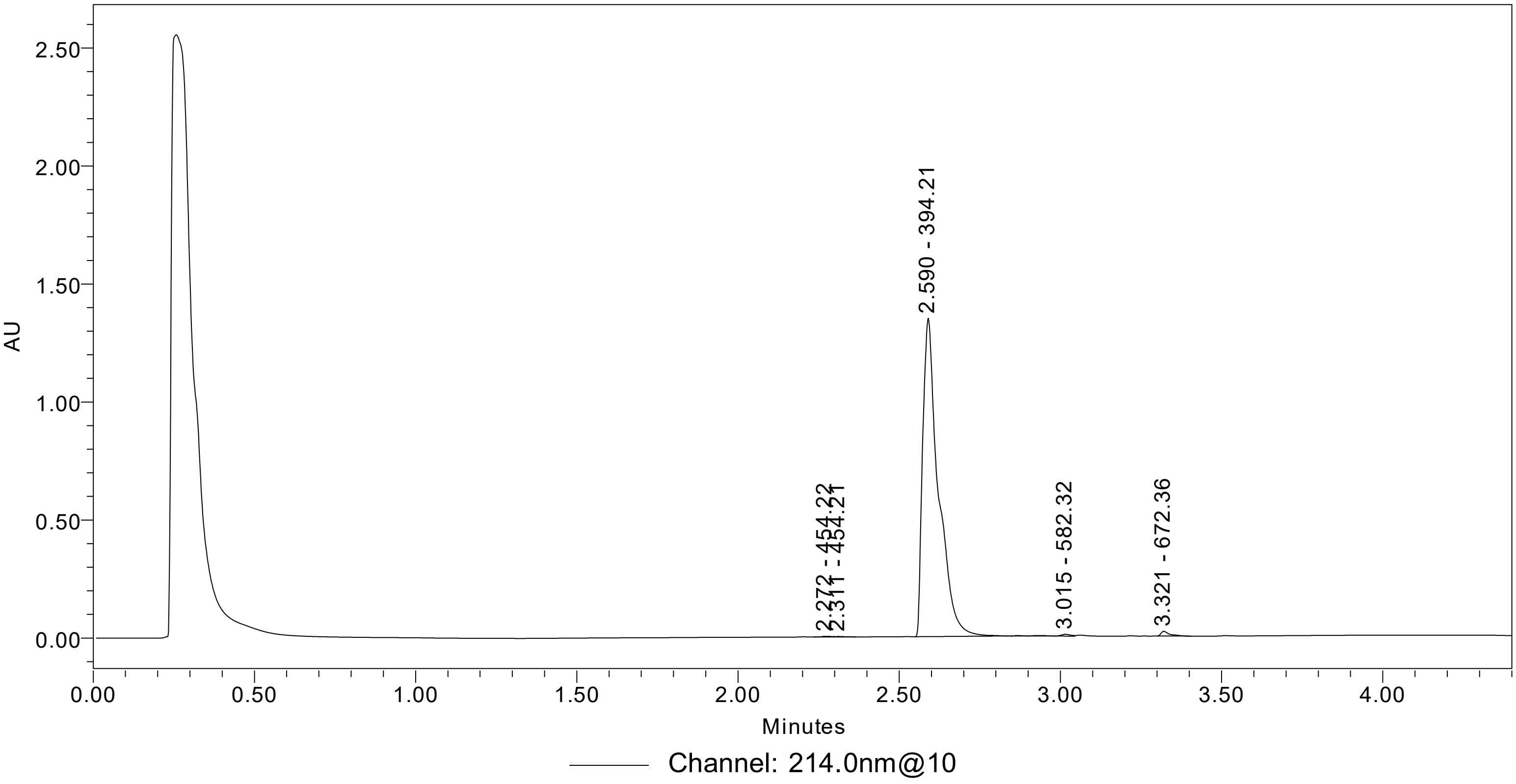

	RT	Area	% Area	Height	Base Peak (m/z)
1	2.272	5205	0.11	2689	454.22
2	2.311	3919	0.08	1855	454.21
3	2.590	4580829	98.49	1348020	394.21
4	3.015	24616	0.53	7521	582.32
5	3.321	36362	0.78	19824	672.36

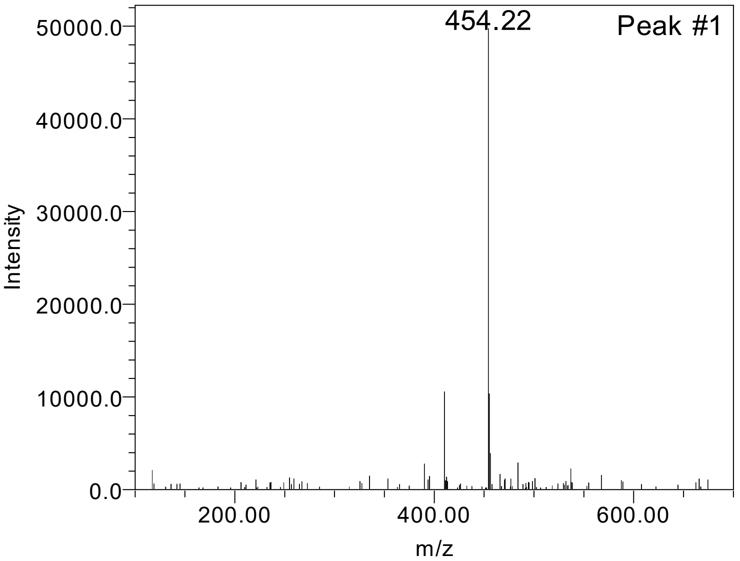

RT: 2.272

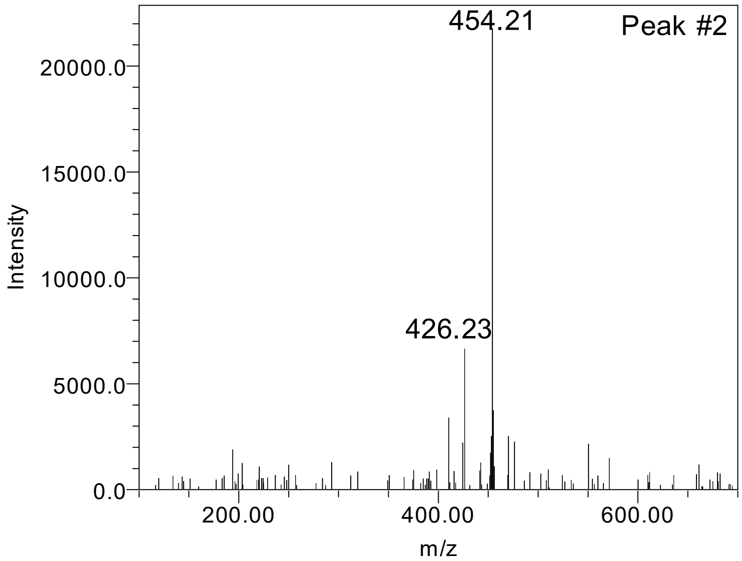

RT: 2.311

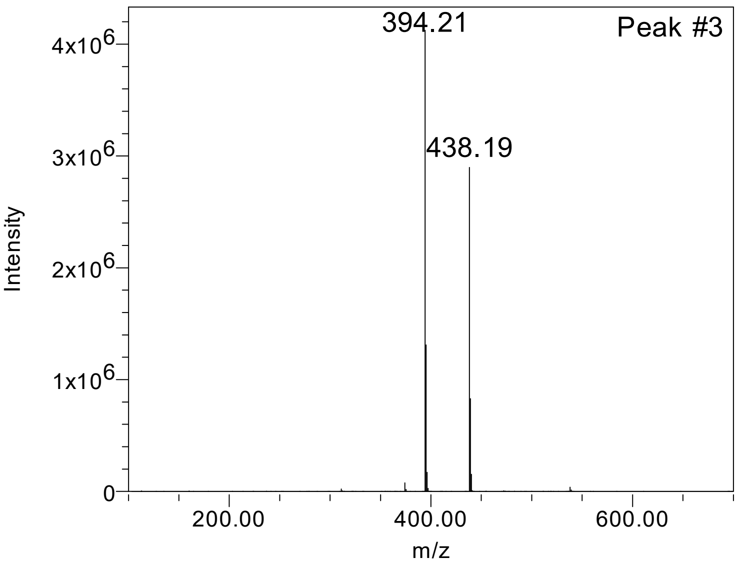

RT: 2.590

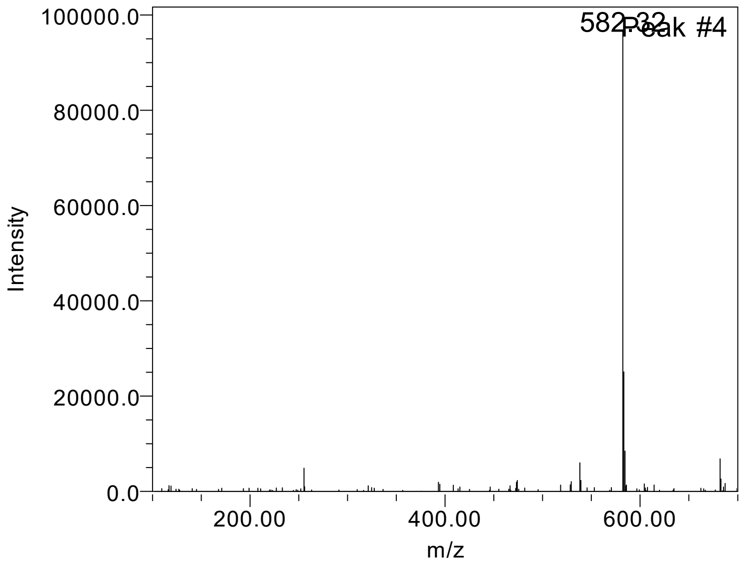

RT: 3.015

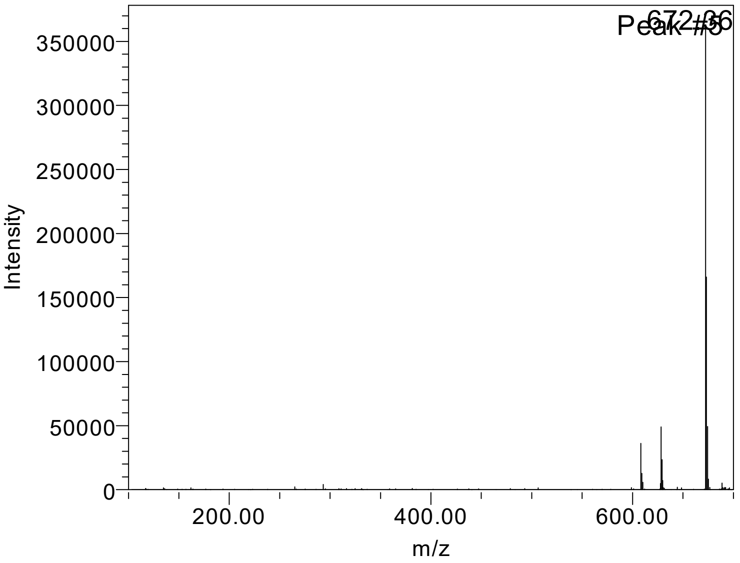

RT: 3.321

Mass Analysis Report

SAMPLE INFORMATION

Sample Name: SR210620A
Acq Method Set: Col1_MeOH_H2O_NH4HCO3
Acquired: 6/24/2021 10:38:11 PM CDT
InjVol: 7.50 uL

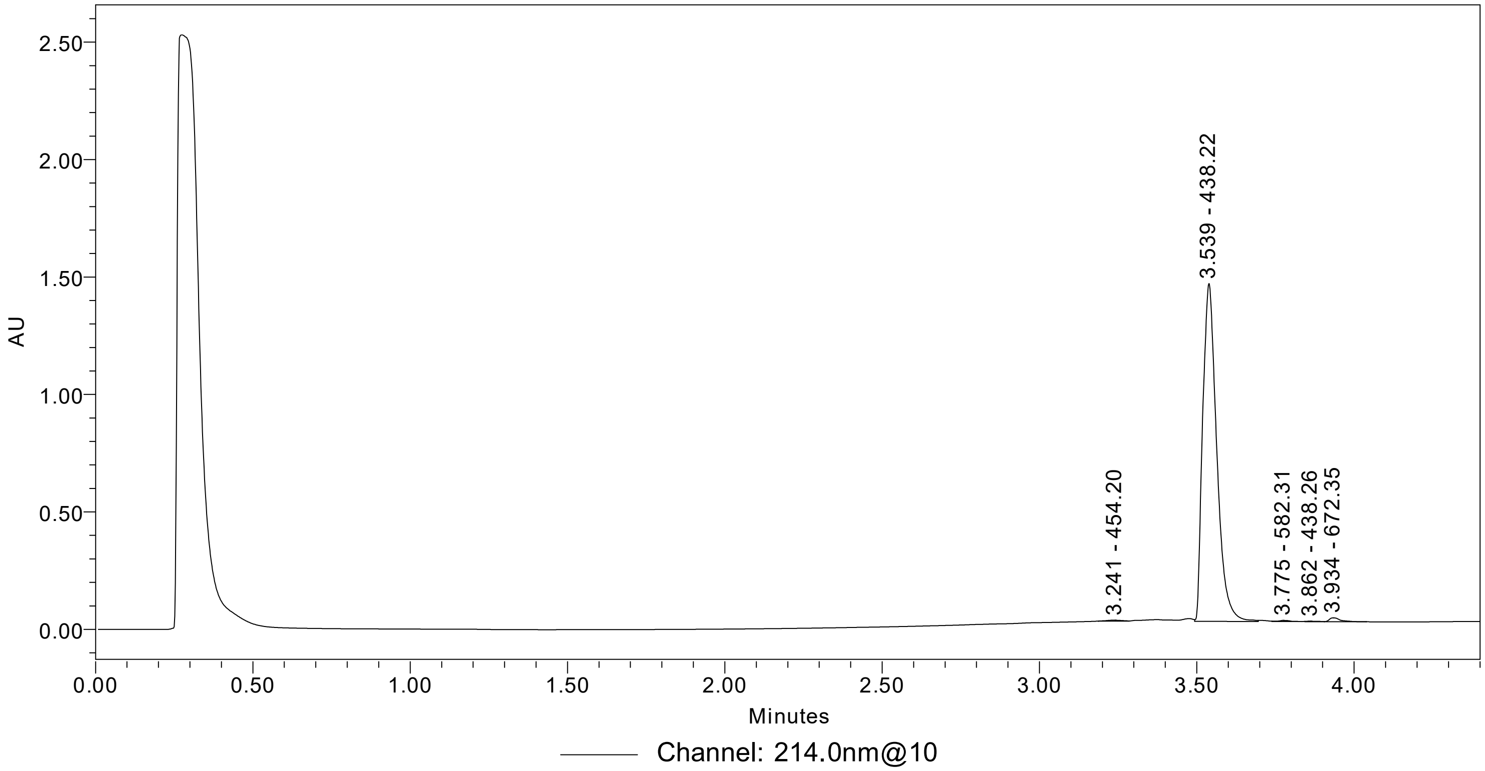

	RT	Area	% Area	Height	Base Peak (m/z)
1	3.241	14317	0.32	3846	454.20
2	3.539	4455040	98.39	1438065	438.22
3	3.775	13243	0.29	4917	582.31
4	3.862	5321	0.12	1802	438.26
5	3.934	39897	0.88	16379	672.35

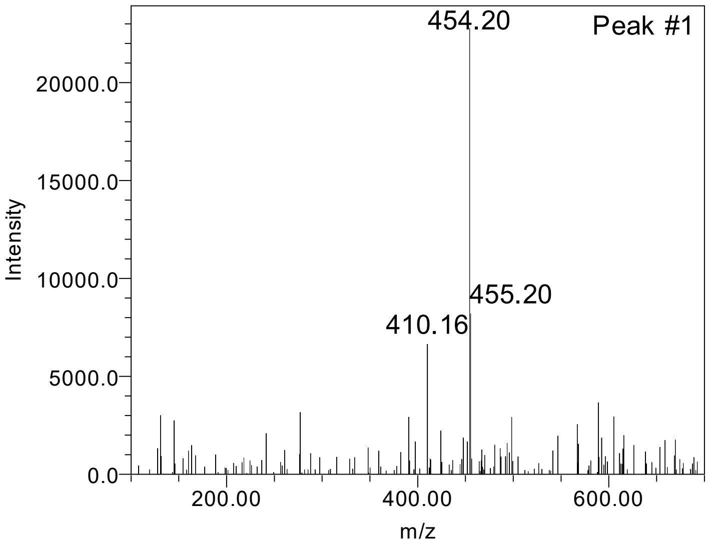

RT: 3.241

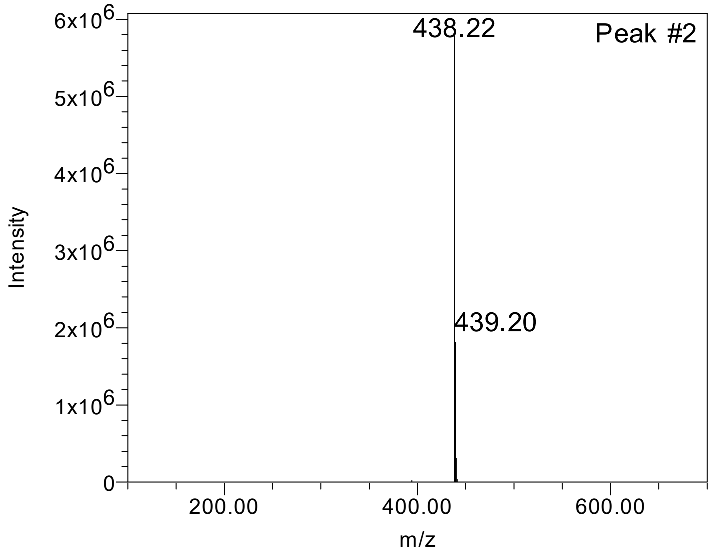

RT: 3.539

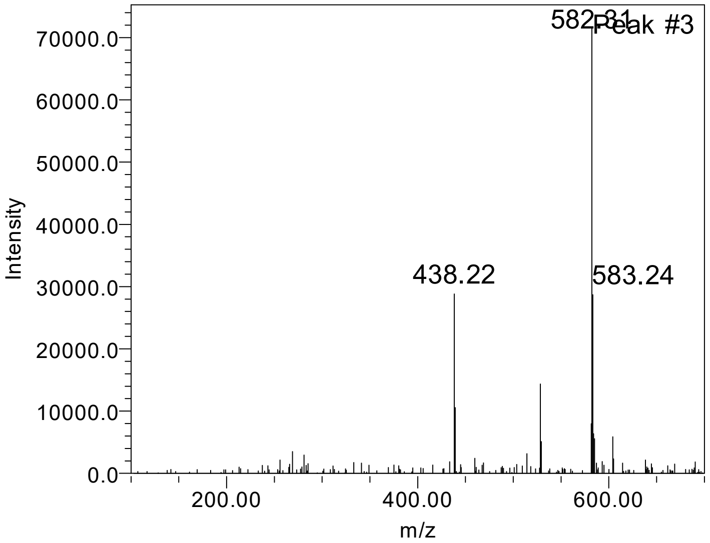

RT: 3.775

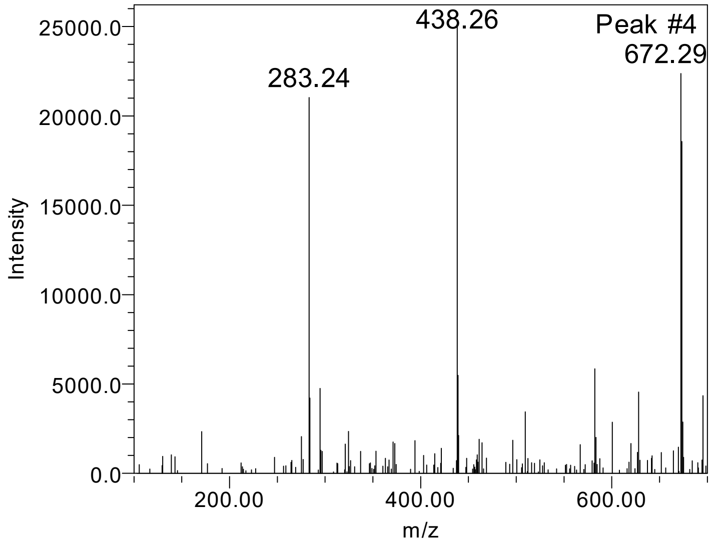

RT: 3.862

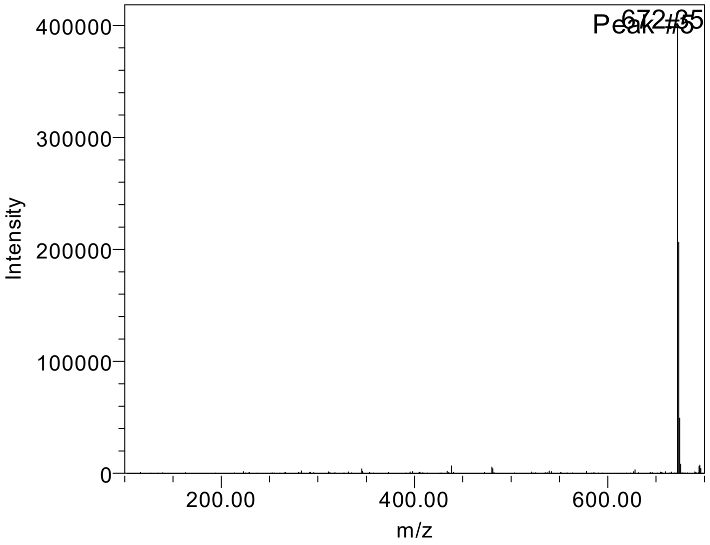

RT: 3.934

Mass Analysis Report

SAMPLE INFORMATION

Sample Name: SR210620B
Acq Method Set: Col2_MeCN_H2O_NH4HCO3

Acquired: 6/24/2021 7:28:43 PM CDT
InjVol: 7.50 uL

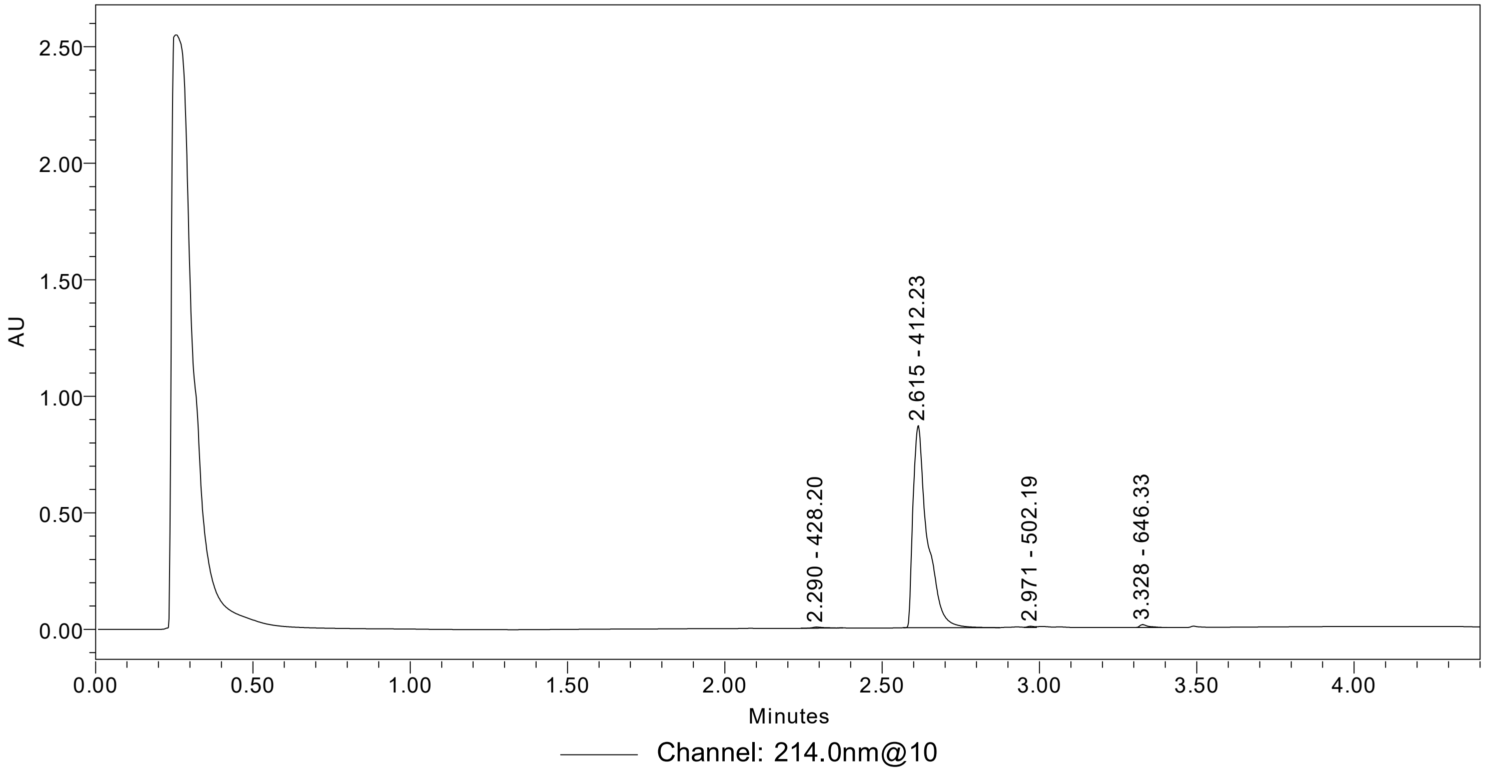

	RT	Area	% Area	Height	Base Peak (m/z)
1	2.290	11924	0.42	4507	428.20
2	2.615	2764878	98.47	867046	412.23
3	2.971	8903	0.32	5461	502.19
4	3.328	22143	0.79	12135	646.33

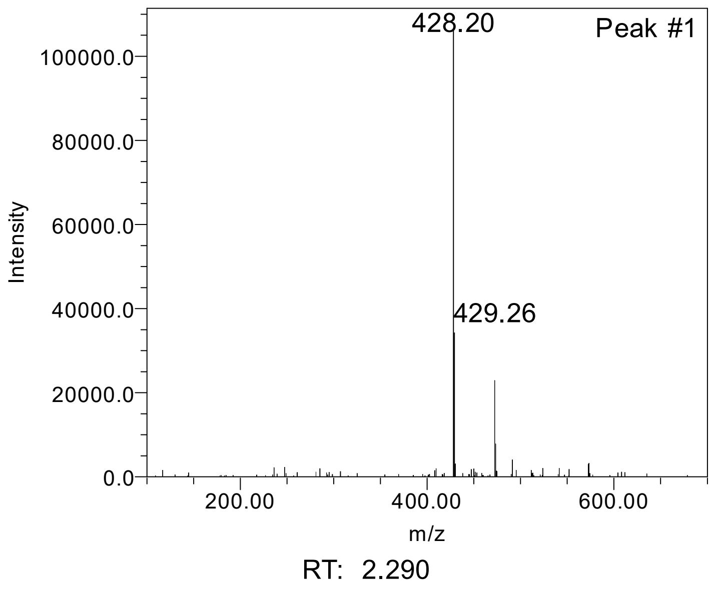
Peak #1
428.20
429.26
Intensity
m/z
RT: 2.290

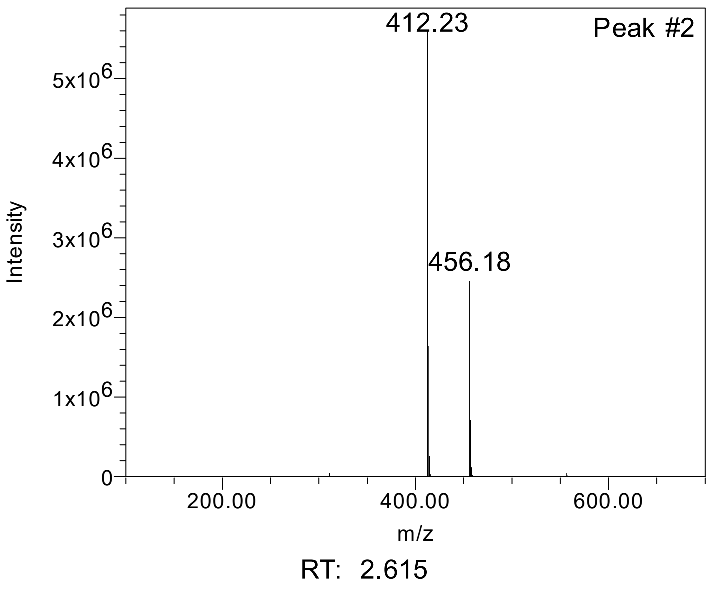
Peak #2
412.23
456.18
Intensity
m/z
RT: 2.615

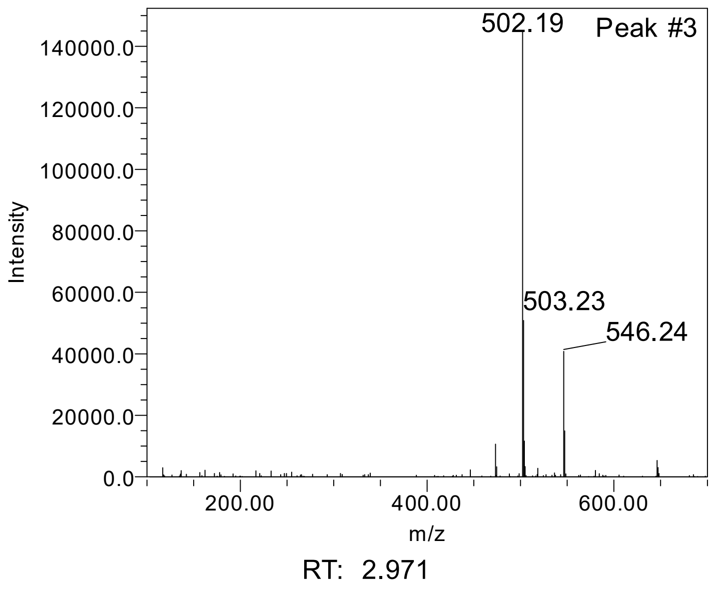
Peak #3
502.19
503.23
546.24
Intensity
m/z
RT: 2.971

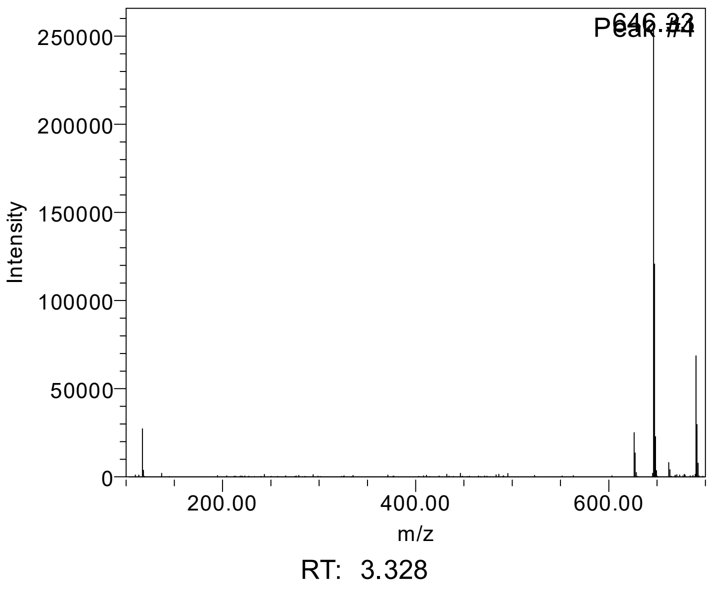
Peak #4
Intensity
m/z
RT: 3.328

Mass Analysis Report

SAMPLE INFORMATION

Sample Name: SR210620B
Acq Method Set: Col1_MeOH_H2O_NH4HCO3
Acquired: 6/24/2021 10:45:16 PM CDT
InjVol: 7.50 uL

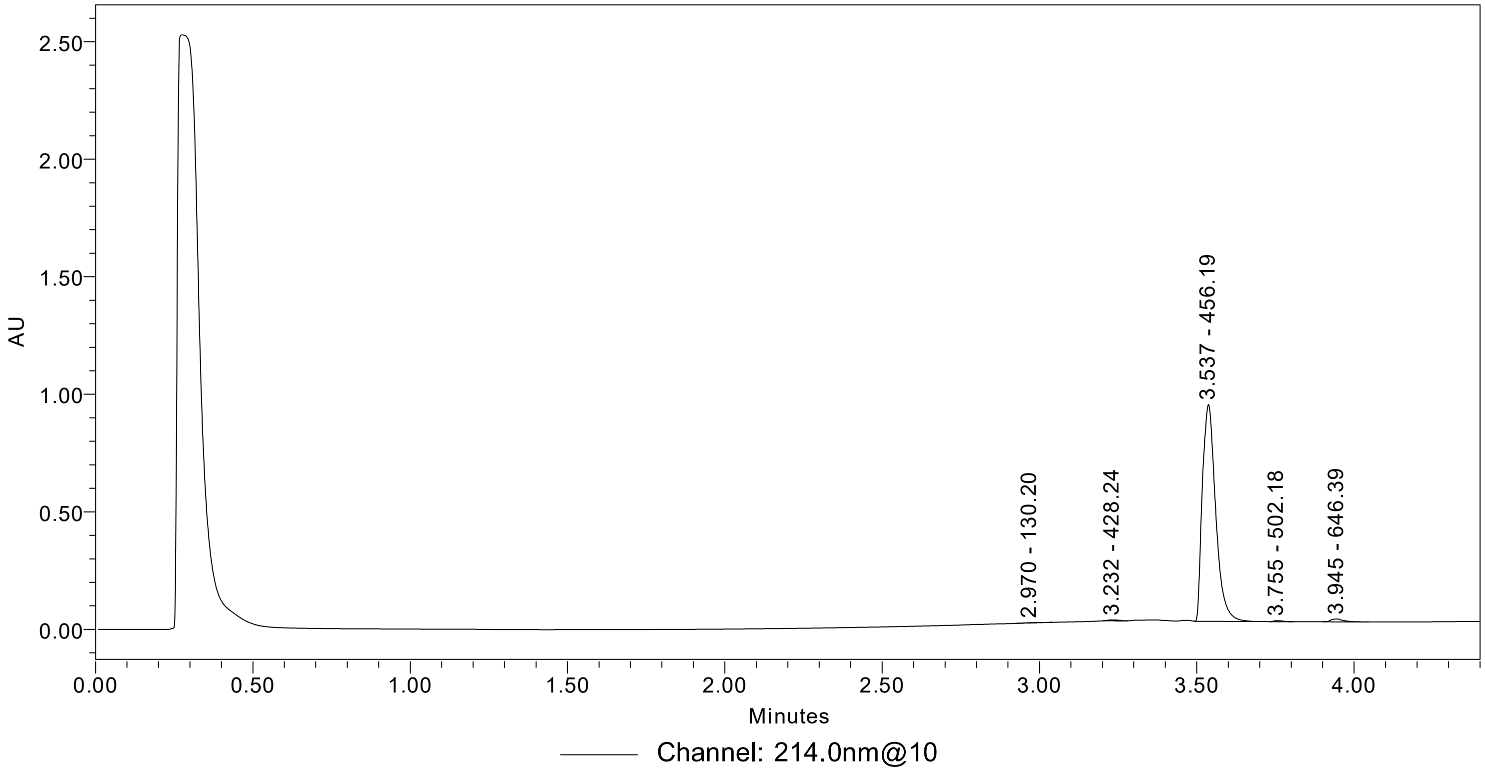

	RT	Area	% Area	Height	Base Peak (m/z)
1	2.970	4381	0.16	1289	130.20
2	3.232	10184	0.37	3699	428.24
3	3.537	2721796	97.99	922134	456.19
4	3.755	8317	0.30	3935	502.18
5	3.945	33054	1.19	12066	646.39

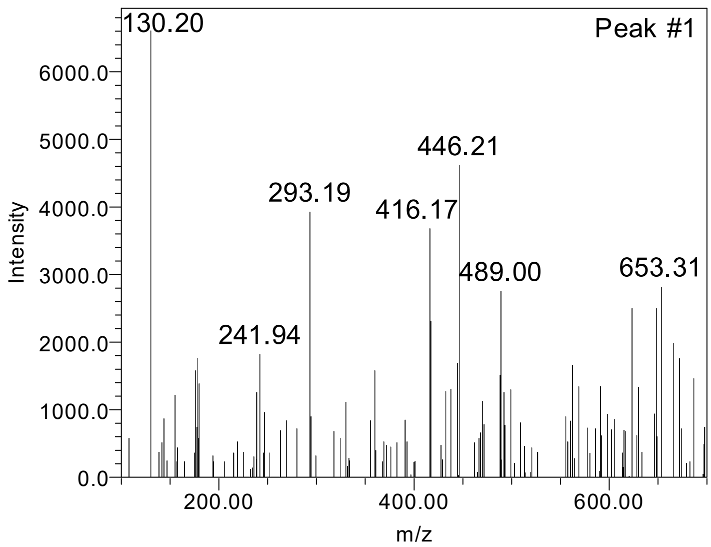

RT: 2.970

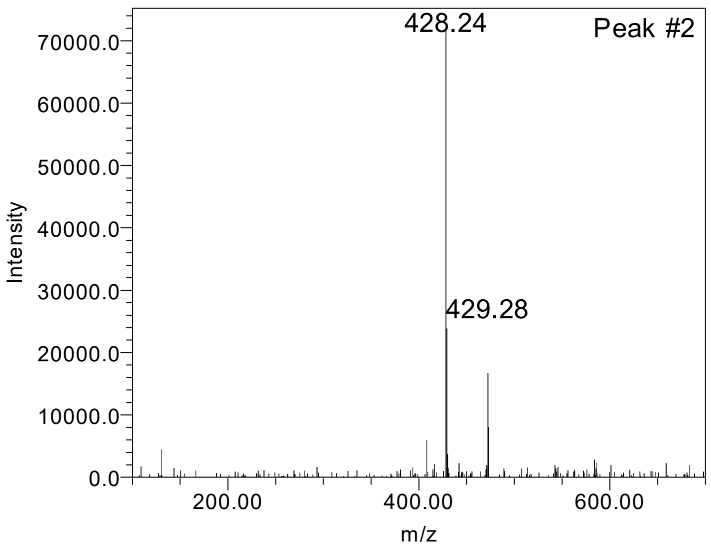

RT: 3.232

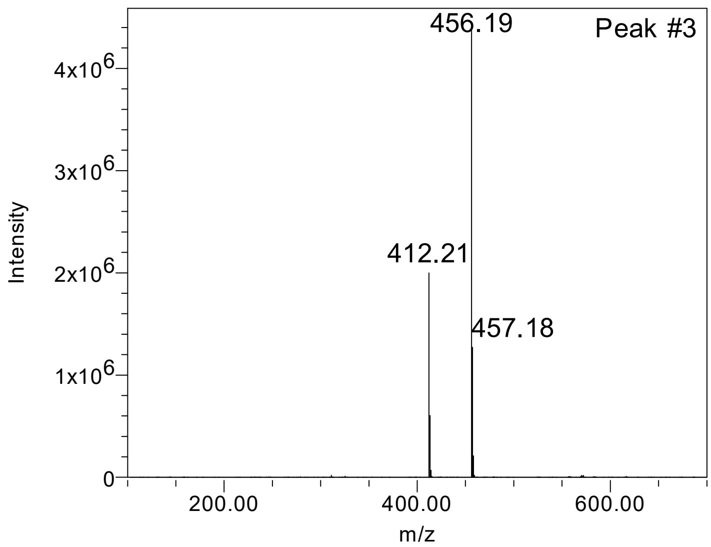

RT: 3.537

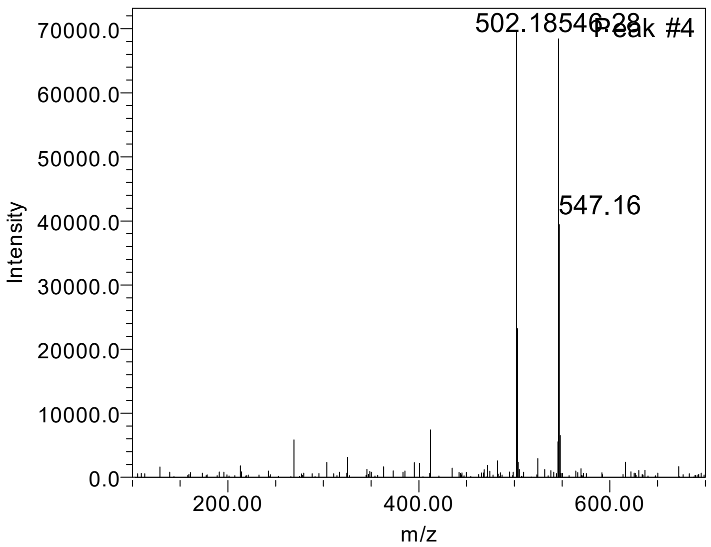

RT: 3.755

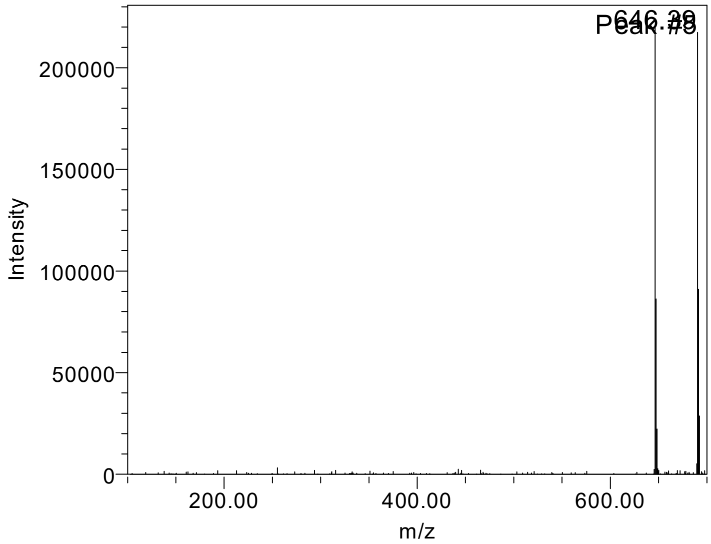

RT: 3.945

Mass Analysis Report

SAMPLE INFORMATION

Sample Name: SR211205B
Acq Method Set: Col2_MeCN_H2O_NH4HCO3
Acquired: 12/21/2021 8:20:12 PM CST
InjVol: 7.50 uL

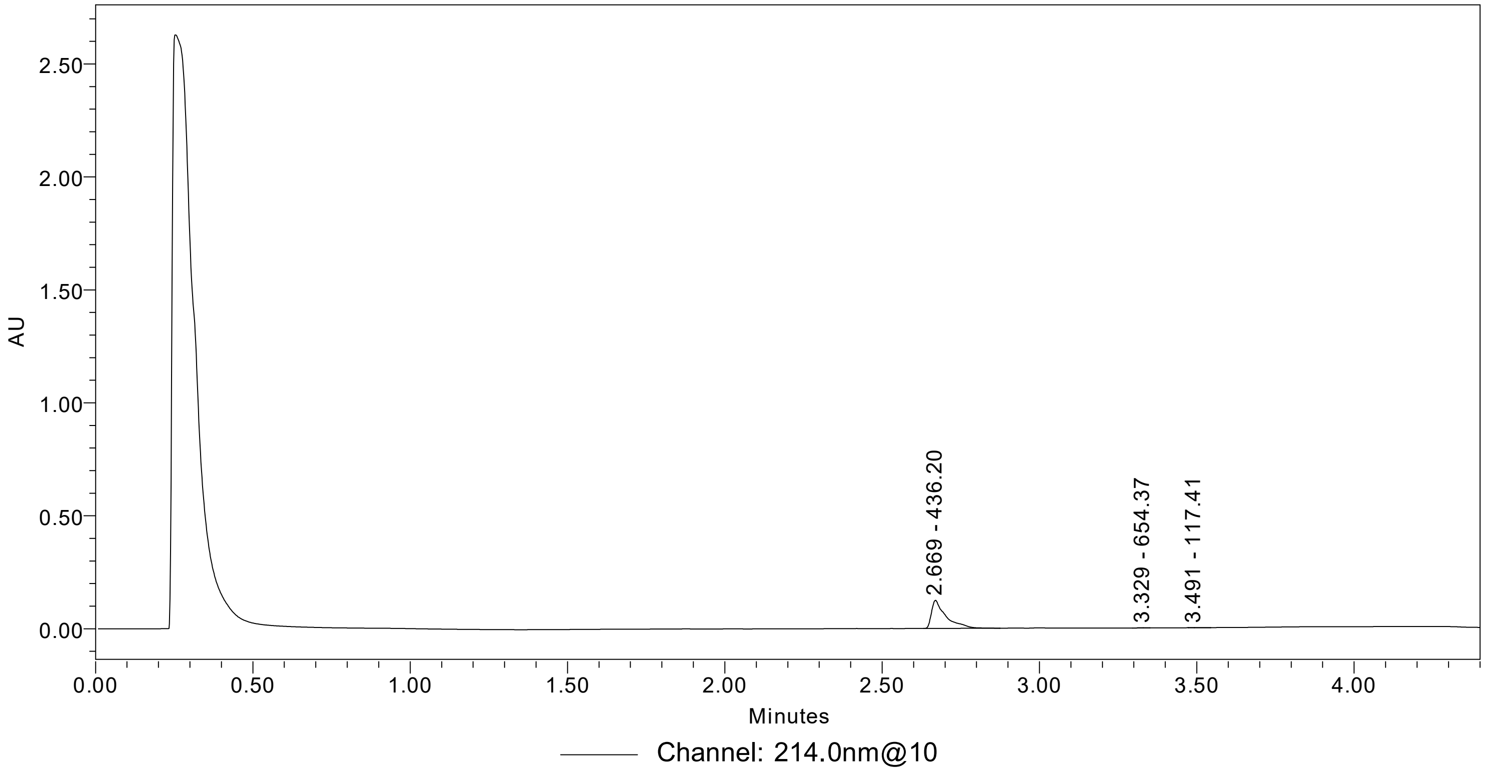

	RT	Area	% Area	Height	Base Peak (m/z)
1	2.669	398147	99.60	124359	436.20
2	3.329	483	0.12	356	654.37
3	3.491	1097	0.27	454	117.41

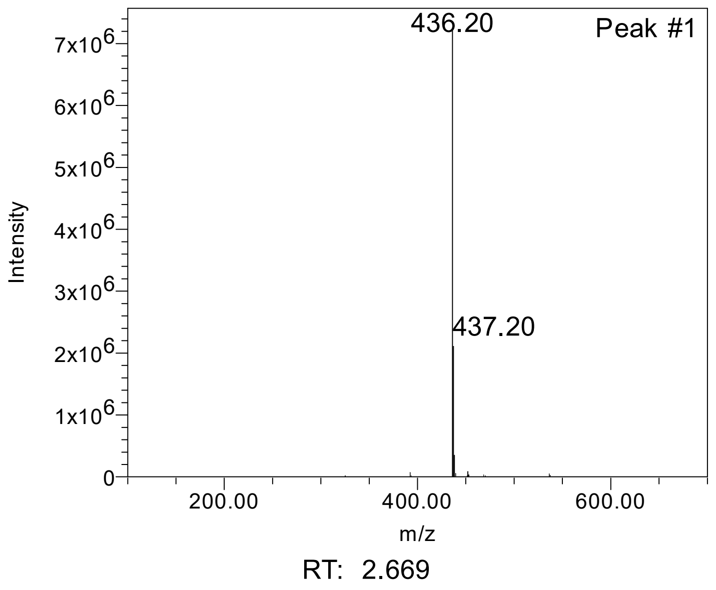
Peak #1
436.20
437.20
Intensity
m/z
RT: 2.669

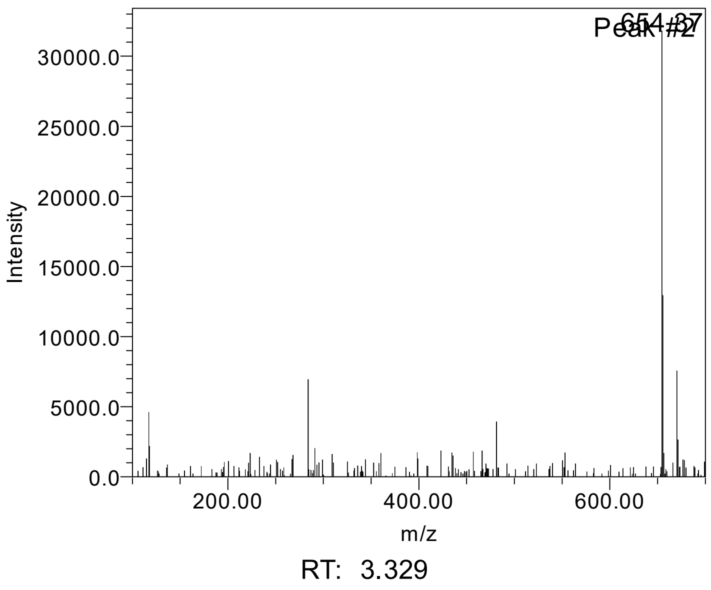
Peak #2
Intensity
m/z
RT: 3.329

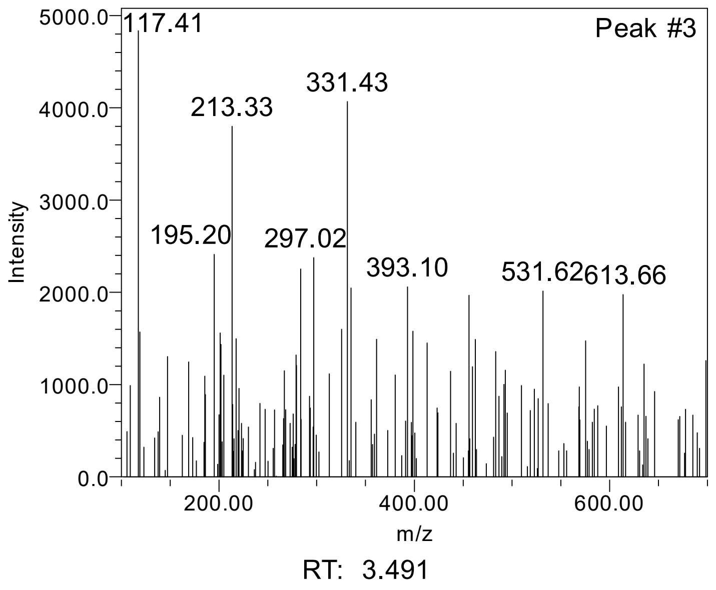
Peak #3
117.41
213.33
331.43
195.20
297.02
393.10
531.62
613.66
Intensity
m/z
RT: 3.491

Mass Analysis Report

SAMPLE INFORMATION

Sample Name: SR211205B
Acq Method Set: Col1_MeOH_H2O_NH4HCO3

Acquired: 12/22/2021 3:06:17 AM CST
InjVol: 7.50 uL

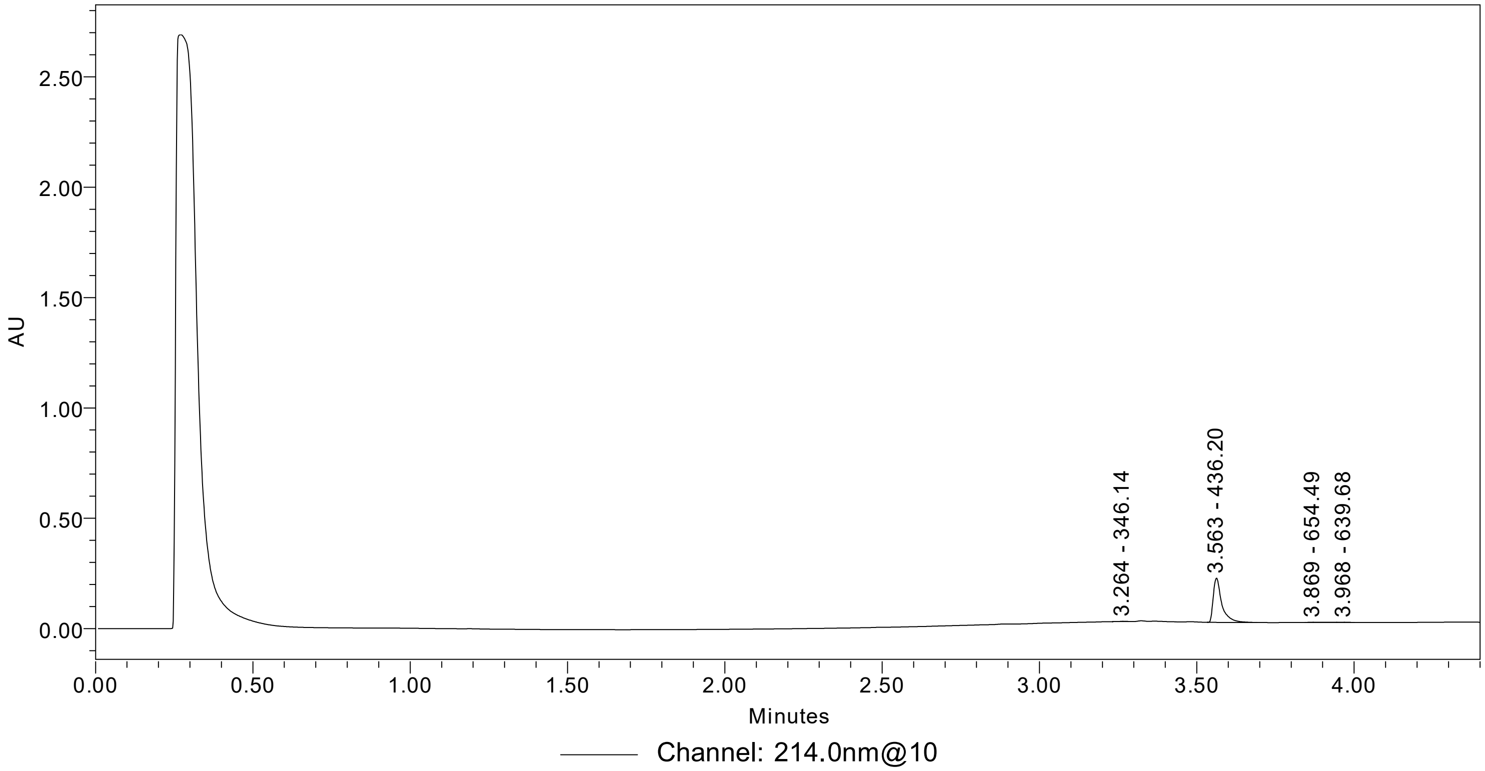

	RT	Area	% Area	Height	Base Peak (m/z)
1	3.264	1150	0.30	678	346.14
2	3.563	378636	98.59	200198	436.20
3	3.869	3099	0.81	828	654.49
4	3.968	1177	0.31	601	639.68

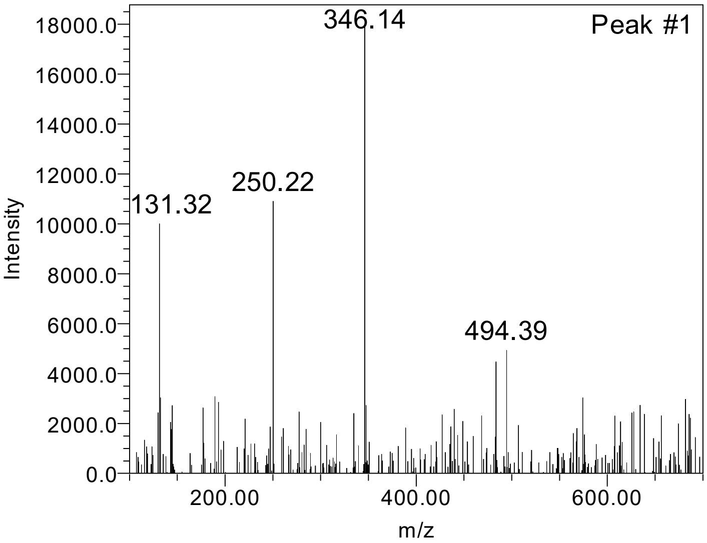

RT: 3.264

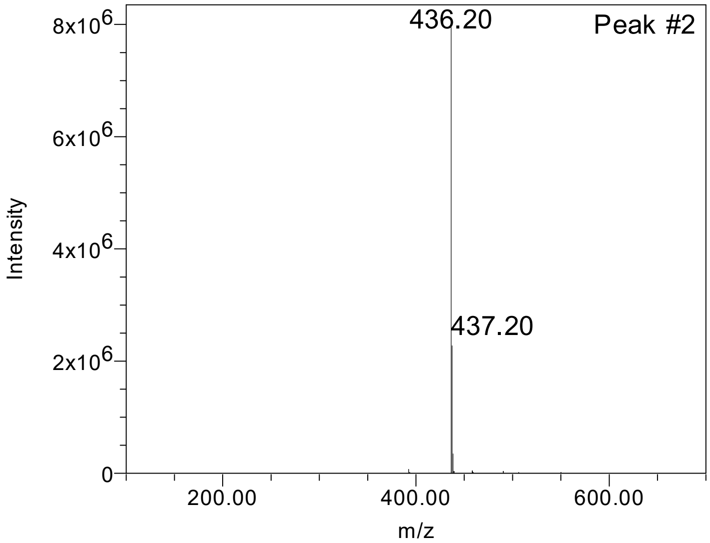

RT: 3.563

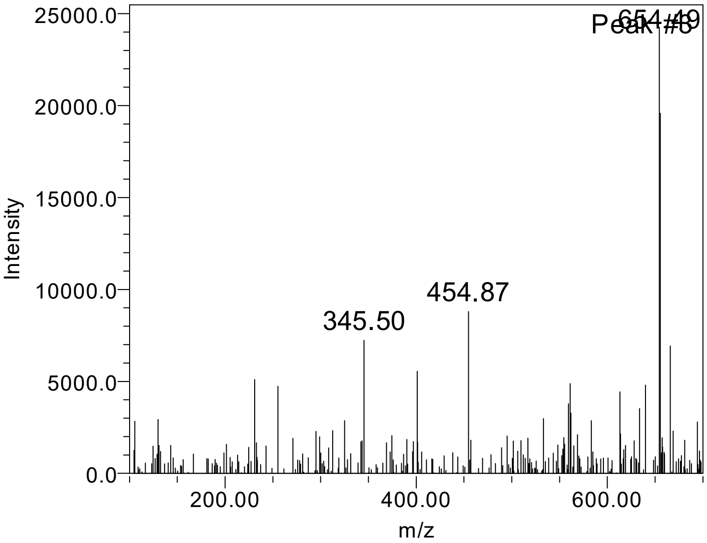

RT: 3.869

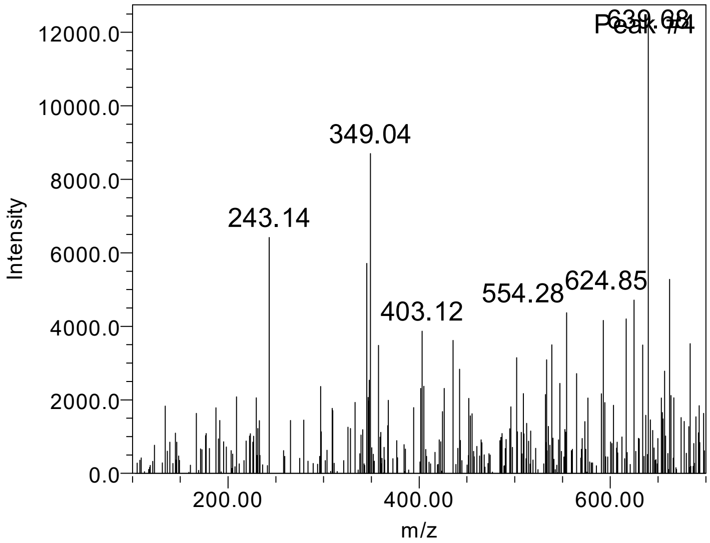

RT: 3.968

Mass Analysis Report

SAMPLE INFORMATION

Sample Name: SR210331C
Acq Method Set: Col2_MeCN_H2O_NH4HCO3
Acquired: 5/14/2021 5:59:23 PM CDT
InjVol: 7.50 uL

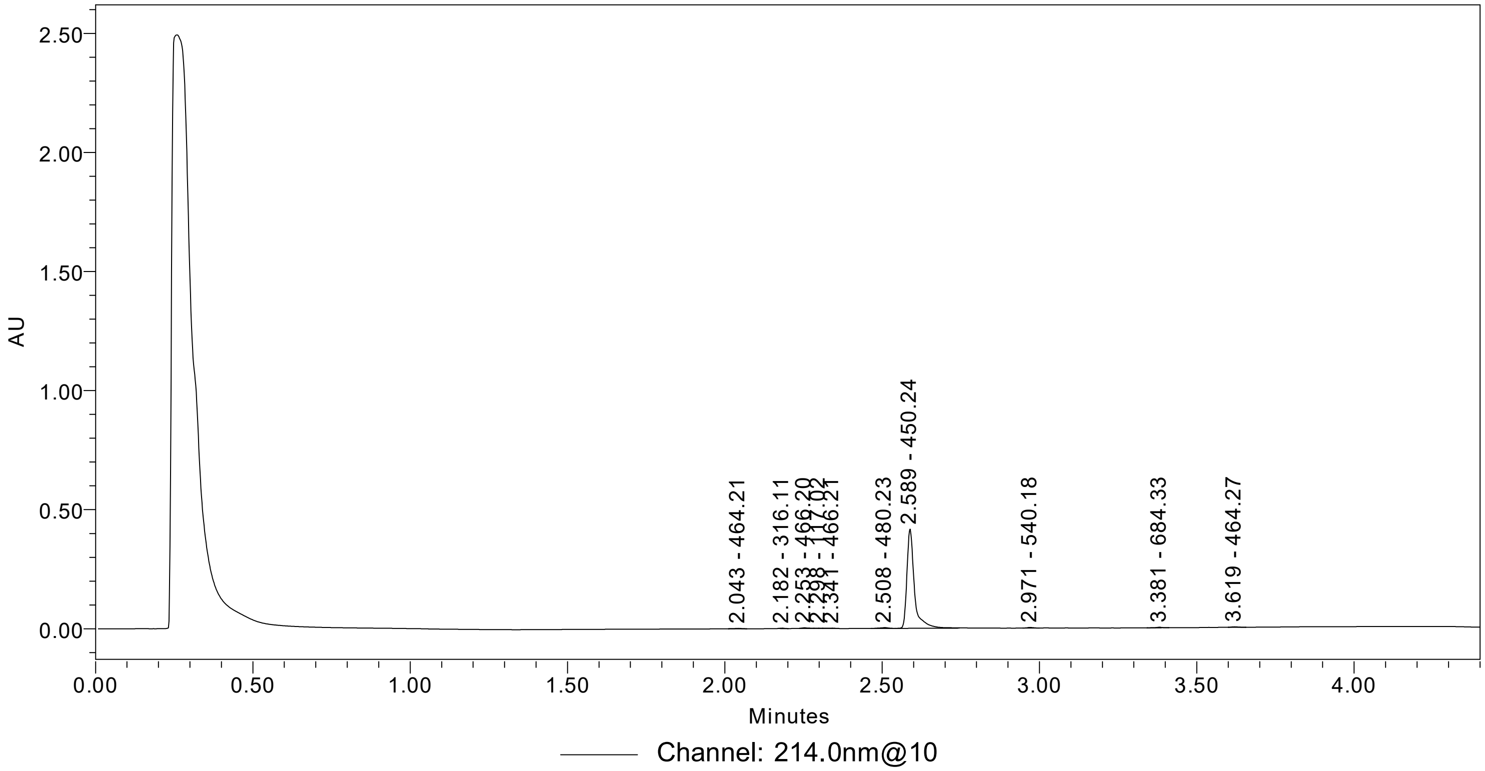

	RT	Area	% Area	Height	Base Peak (m/z)
1	2.043	2367	0.33	1595	464.21
2	2.182	1351	0.19	1371	316.11
3	2.253	3524	0.49	2268	466.20
4	2.298	1258	0.18	896	117.02
5	2.341	2483	0.35	1385	466.21
6	2.508	6481	0.90	3257	480.23
7	2.589	690002	96.35	416052	450.24
8	2.971	3142	0.44	2406	540.18
9	3.381	3049	0.43	1972	684.33
10	3.619	2455	0.34	1777	464.27

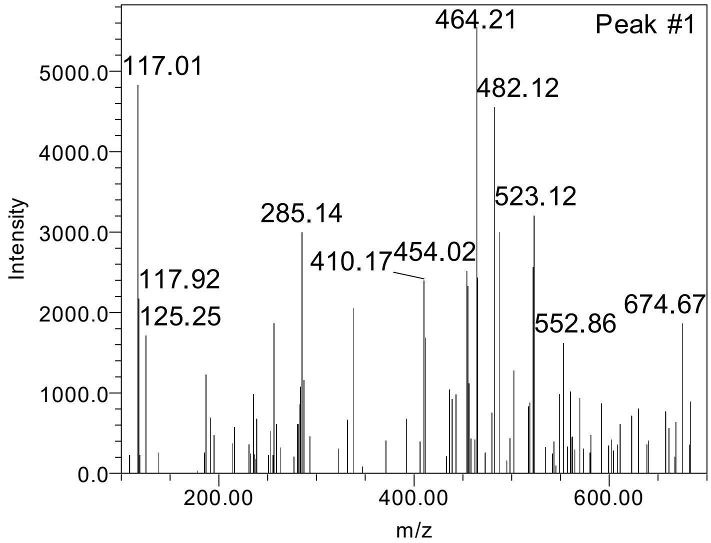

RT: 2.043

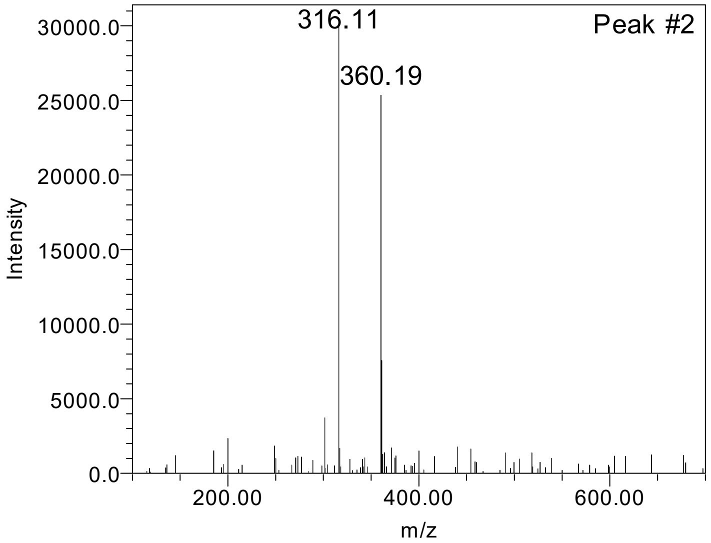

RT: 2.182

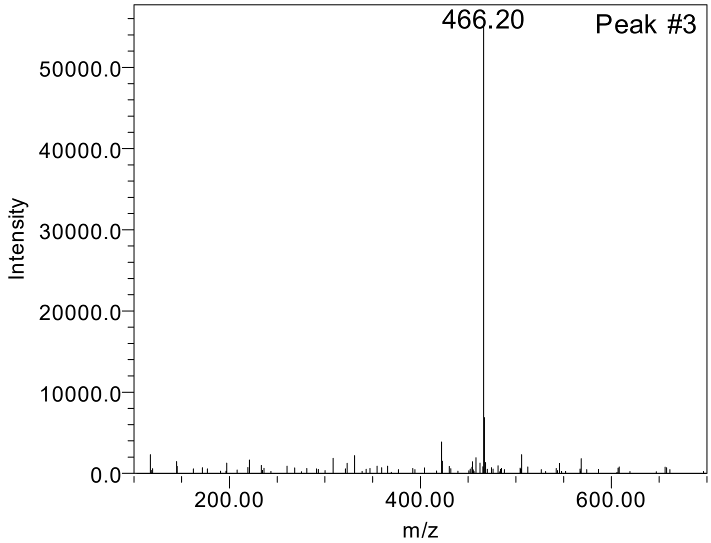

RT: 2.253

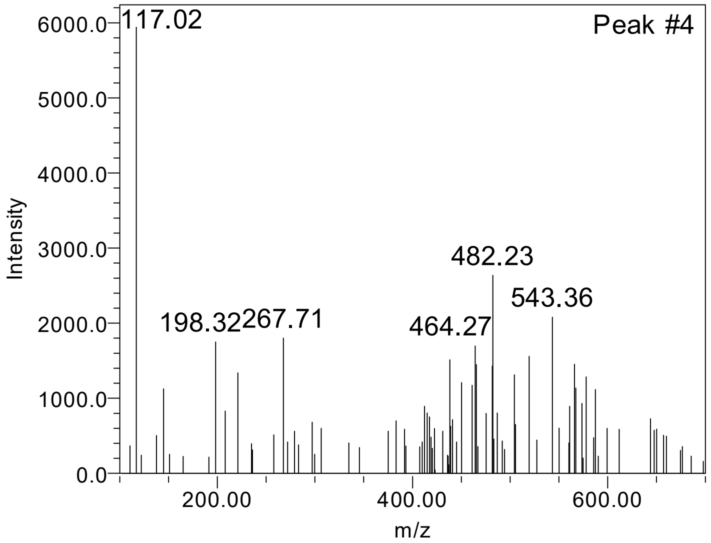

RT: 2.298

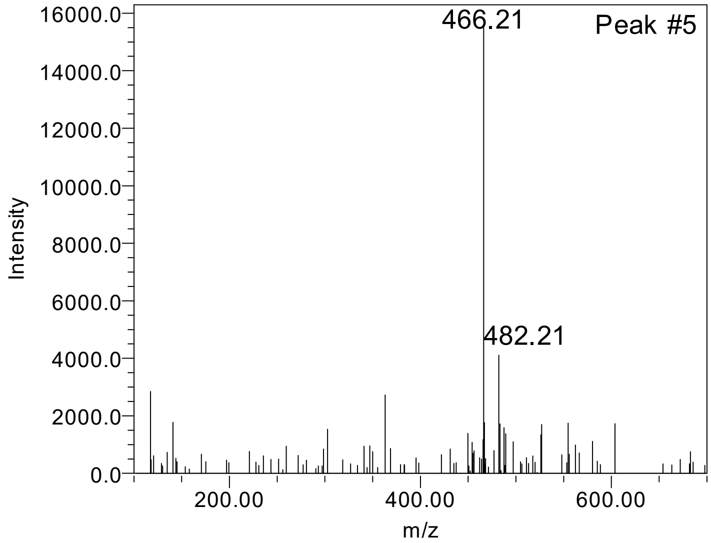

RT: 2.341

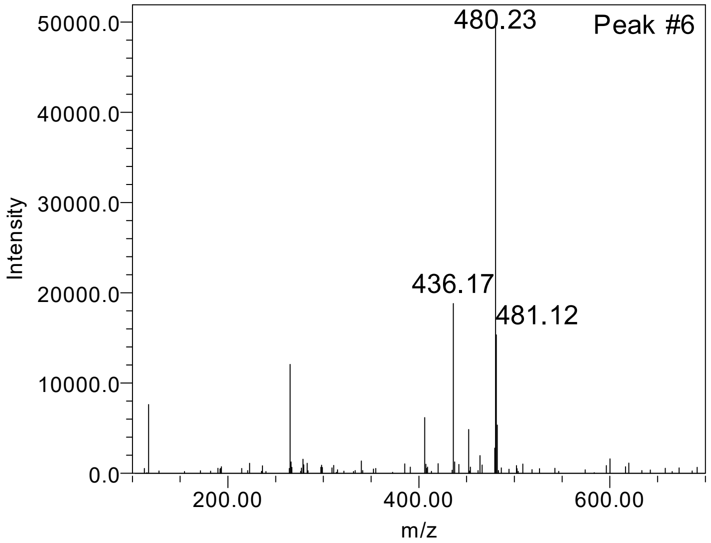

RT: 2.508

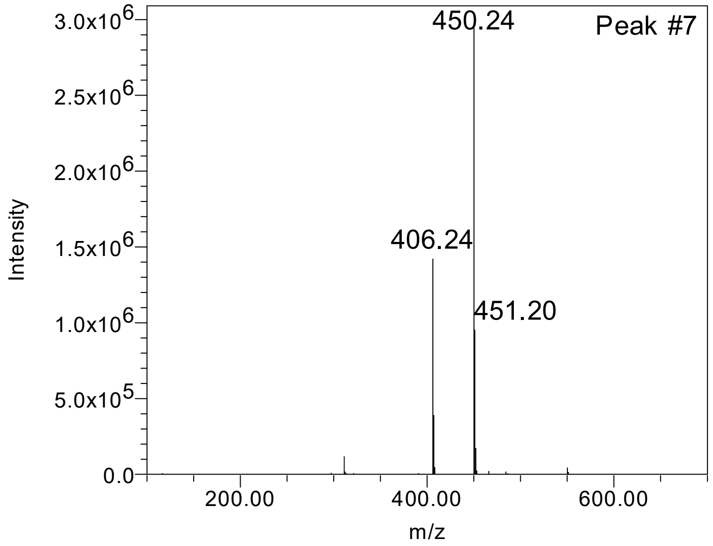

RT: 2.589

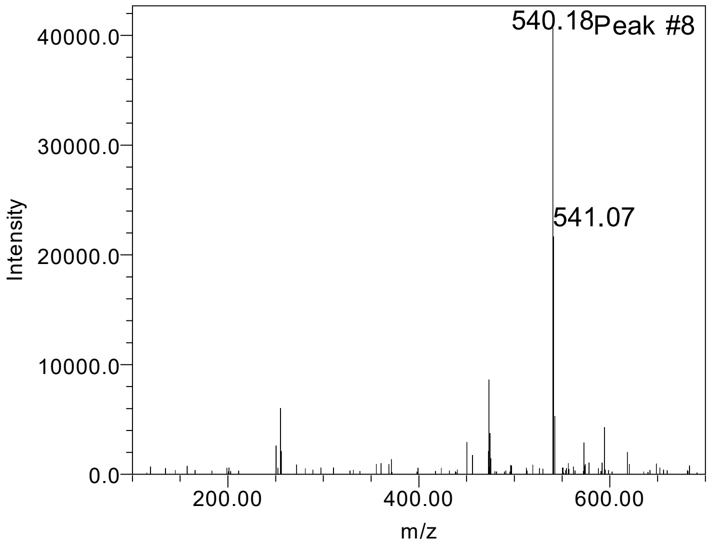

RT: 2.971

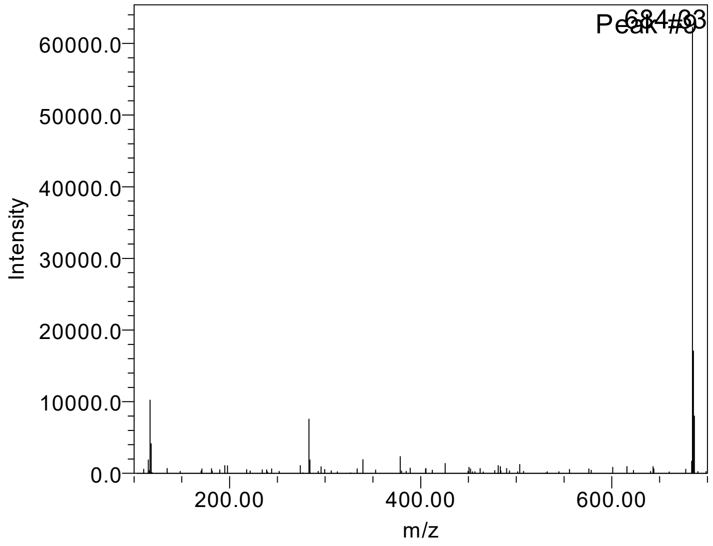

RT: 3.381

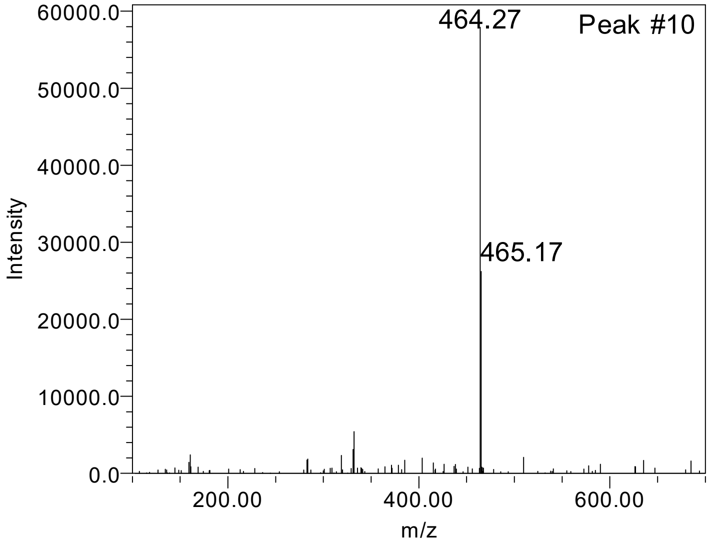

RT: 3.619

Mass Analysis Report

SAMPLE INFORMATION

Sample Name: SR210331C
Acq Method Set: Col1_MeOH_H2O_NH4HCO3
Acquired: 5/15/2021 12:39:54 AM CDT
InjVol: 7.50 uL

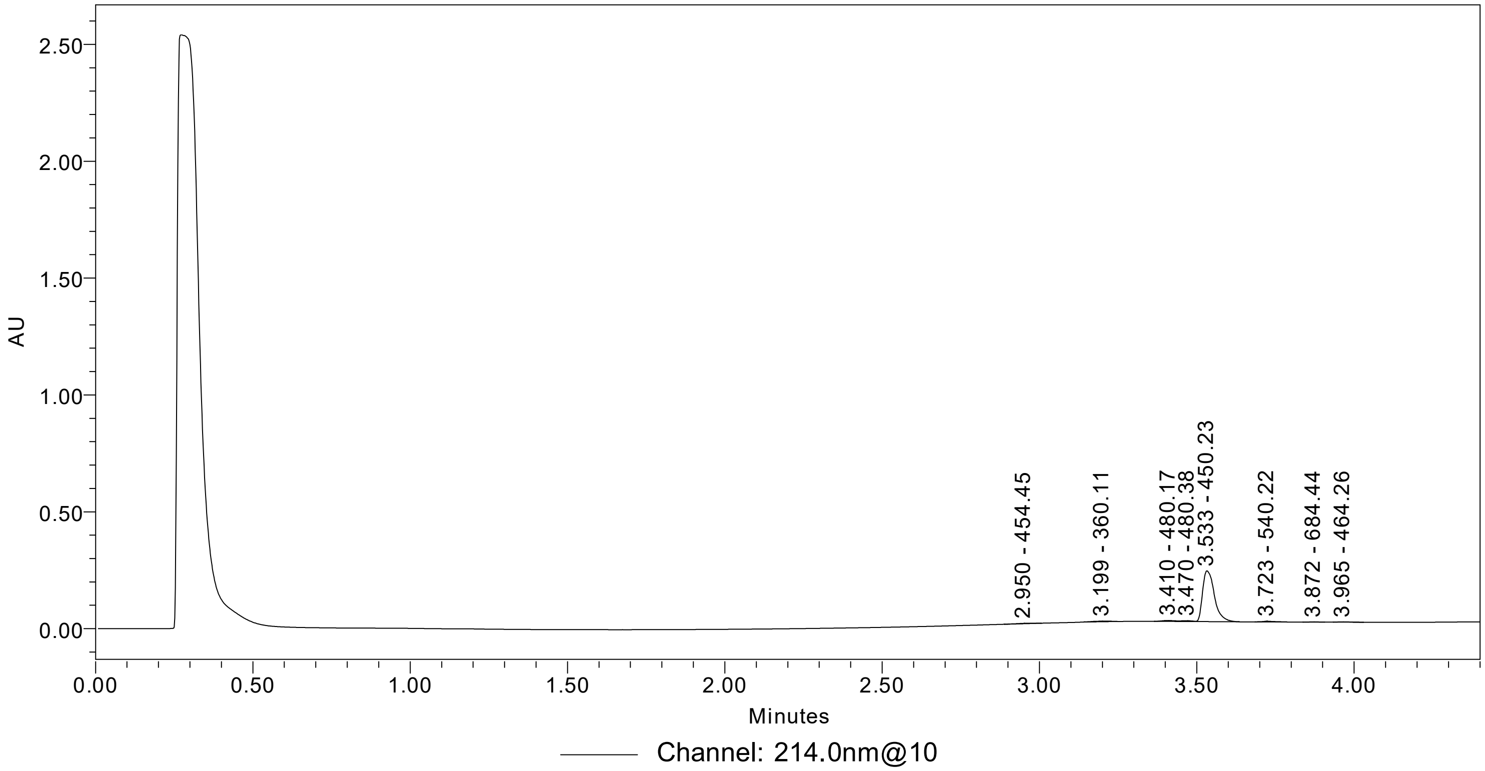

	RT	Area	% Area	Height	Base Peak (m/z)
1	2.950	5079	0.83	1537	454.45
2	3.199	9977	1.63	2587	360.11
3	3.410	10464	1.71	3668	480.17
4	3.470	8579	1.40	3031	480.38
5	3.533	564797	92.31	217074	450.23
6	3.723	8246	1.35	3181	540.22
7	3.872	1955	0.32	1141	684.44
8	3.965	2733	0.45	918	464.26

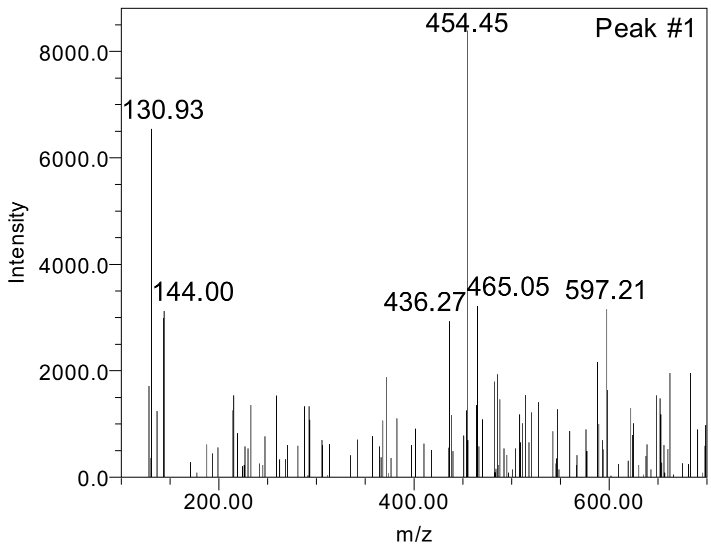

RT: 2.950

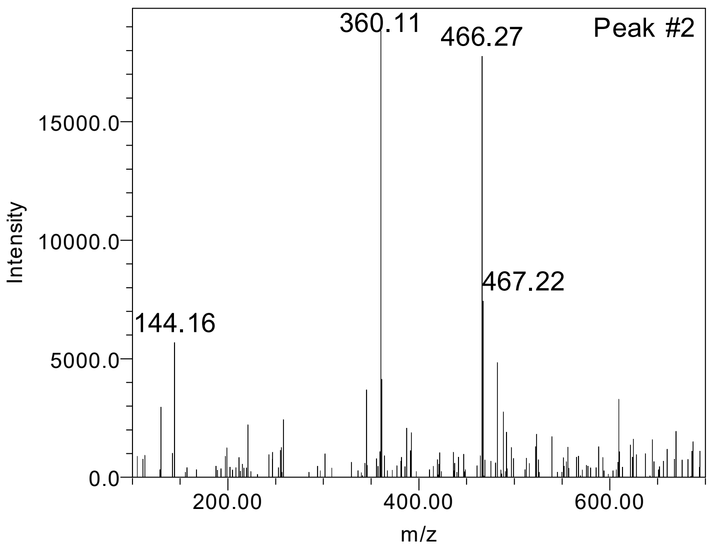

RT: 3.199

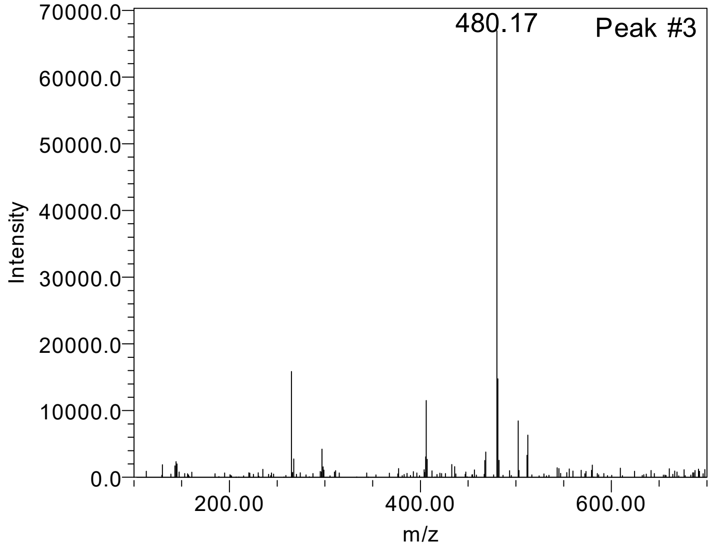

RT: 3.410

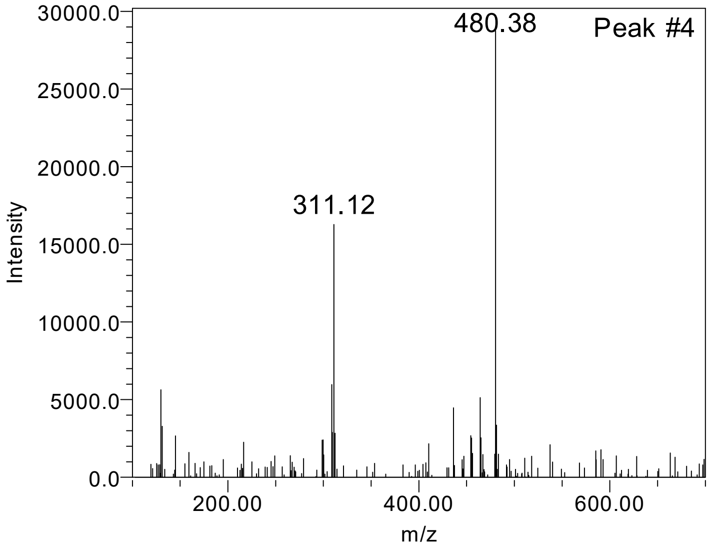

RT: 3.470

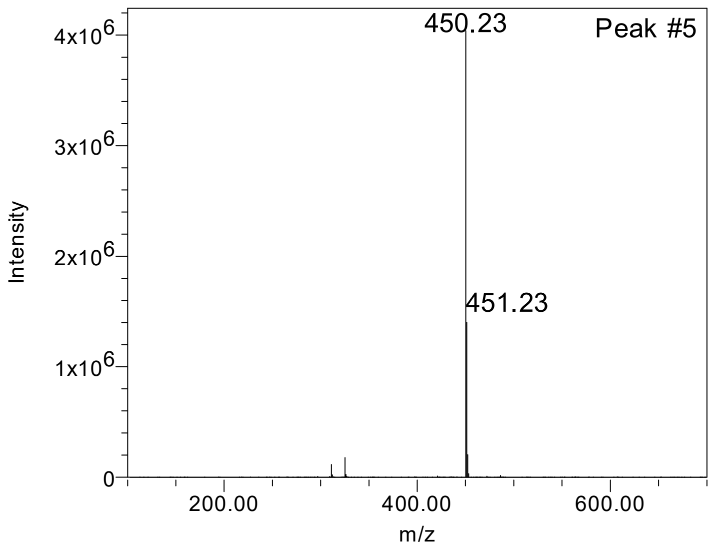

RT: 3.533

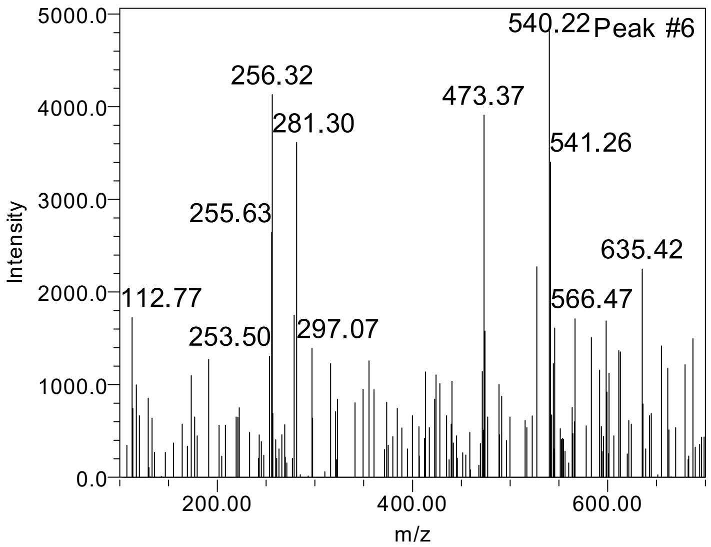

RT: 3.723

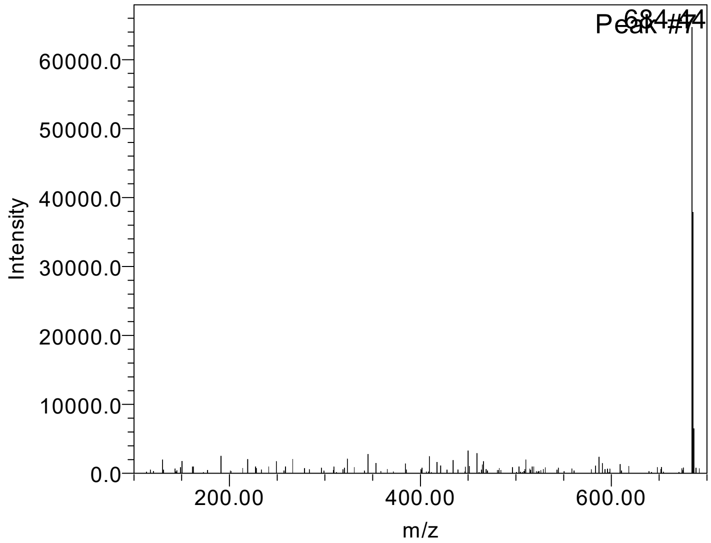

RT: 3.872

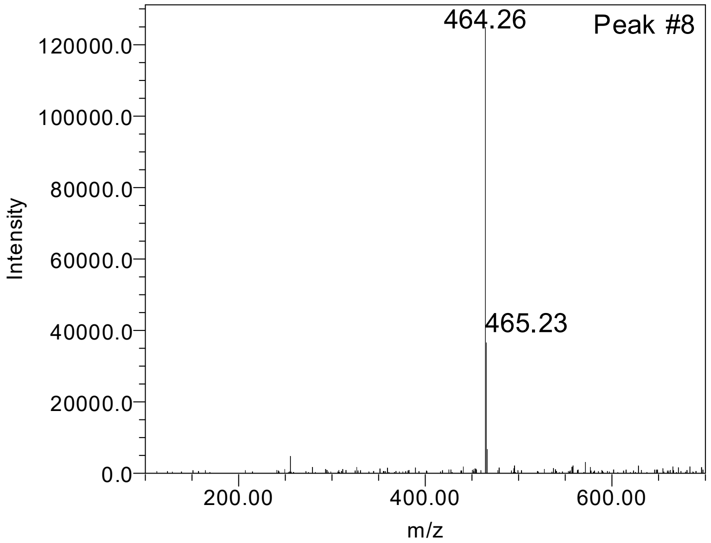

RT: 3.965

Single Injection Report

**Sample name:**	SR230329A		
**Description:**			
**Sample amount:**	0.000	**Sample type:**	Sample
**Instrument:**	LCMS	**Location:**	P1-A1
		**Injection:**	1 of 1
**Acq. method:**	Regular method.amx	**Injection volume:**	5.000 µL
**Analysis method:**	*MS method-purity.pmx	**Acq. operator:**	SYSTEM

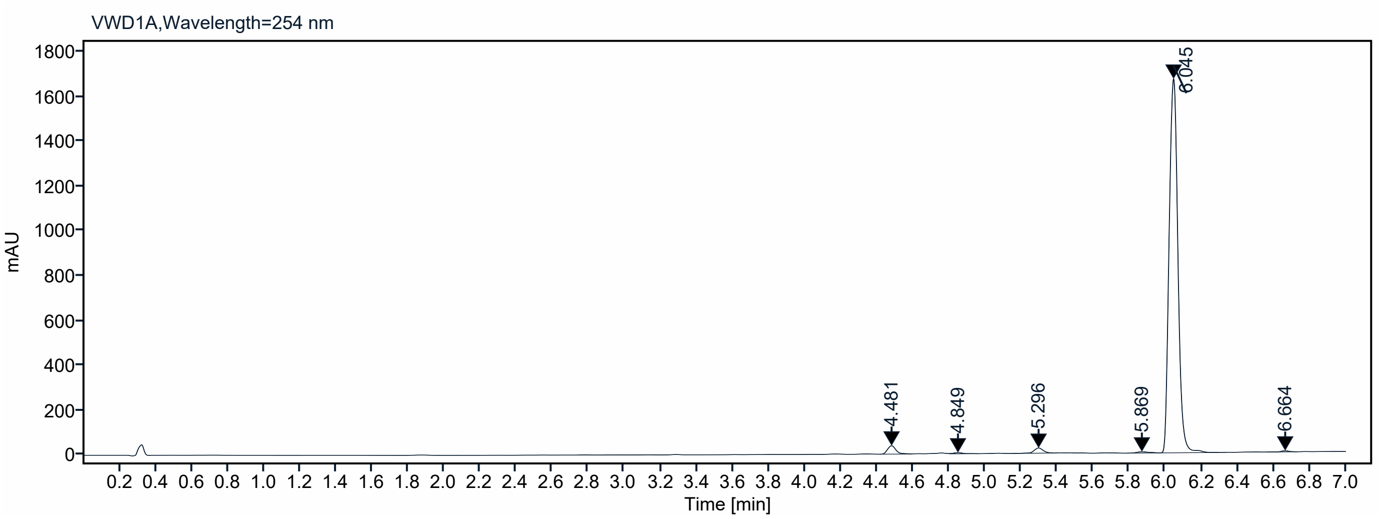

Signal: MS1 +TIC SCAN ESI Frag=110V Gain=1.0

RT [min]	Width [min]	Area	Height	Area%
6.106	0.1458	467030.3815	89267.1687	100.0000
	**Sum**	**467030.3815**		

Signal: VWD1A,Wavelength=254 nm

RT [min]	Width [min]	Area	Height	Area%
4.481	0.1723	121.5276	37.4457	2.0568
4.849	0.1599	18.9041	5.0655	0.3199
5.296	0.2342	81.1654	22.9654	1.3737
5.869	0.1780	27.5086	5.8882	0.4656
6.045	0.3332	5642.9748	1667.4936	95.5026
6.664	0.1764	16.6310	5.2337	0.2815
	**Sum**	**5908.7115**		

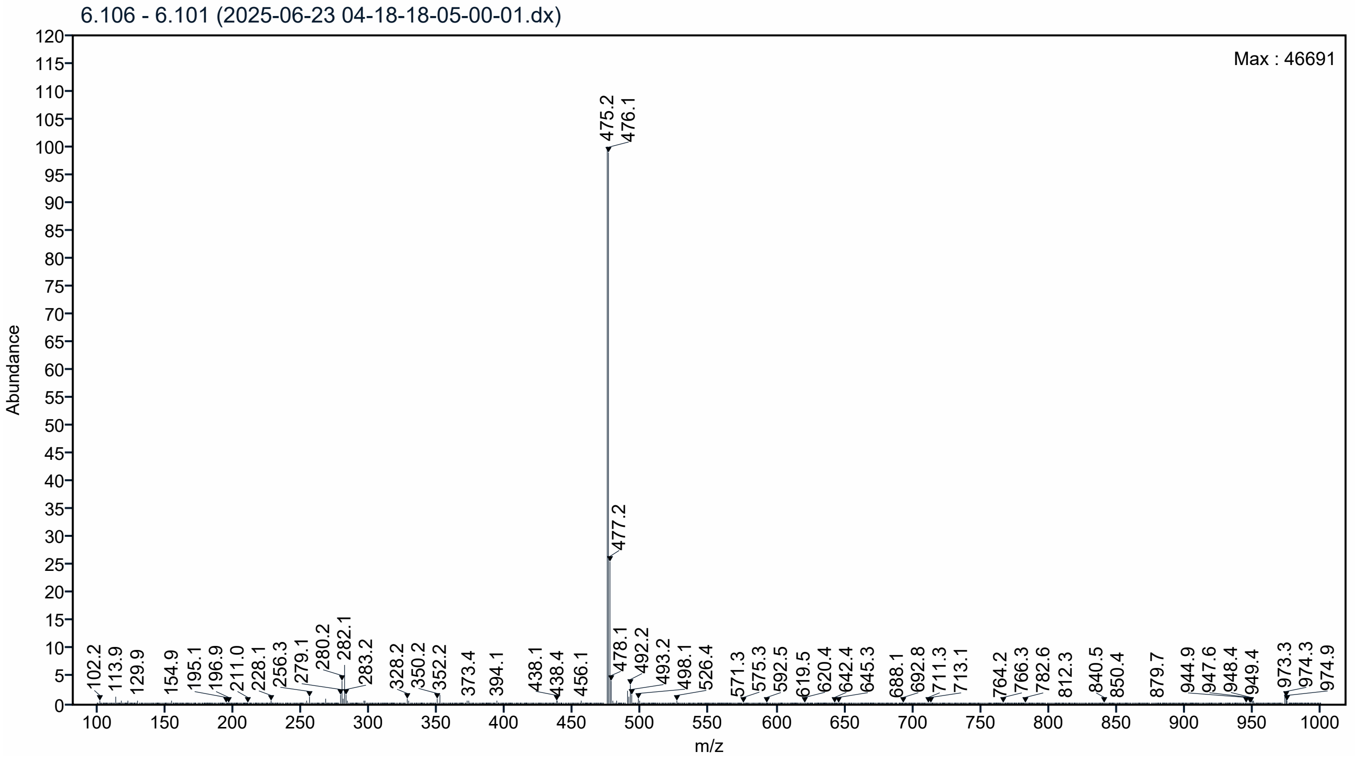
6.106 - 6.101 (2025-06-23 04-18-18-05-00-01.dx)
Max : 46691
Abundance
m/z
475.2
476.1
477.2
478.1
492.2
493.2
498.1
526.4
102.2
113.9
129.9
154.9
195.1
196.9
211.0
228.1
256.3
279.1
280.2
282.1
283.2
328.2
350.2
352.2
373.4
394.1
438.1
438.4
456.1
571.3
575.3
592.5
619.5
620.4
642.4
645.3
688.1
692.8
711.3
713.1
764.2
766.3
782.6
812.3
840.5
850.4
879.7
944.9
947.6
948.4
949.4
973.3
974.3
974.9

Mass Analysis Report

SAMPLE INFORMATION

Sample Name: SR211003B
Acq Method Set: Col2_MeCN_H2O_NH4HCO3
Acquired: 10/14/2021 6:18:49 PM CDT
InjVol: 7.50 uL

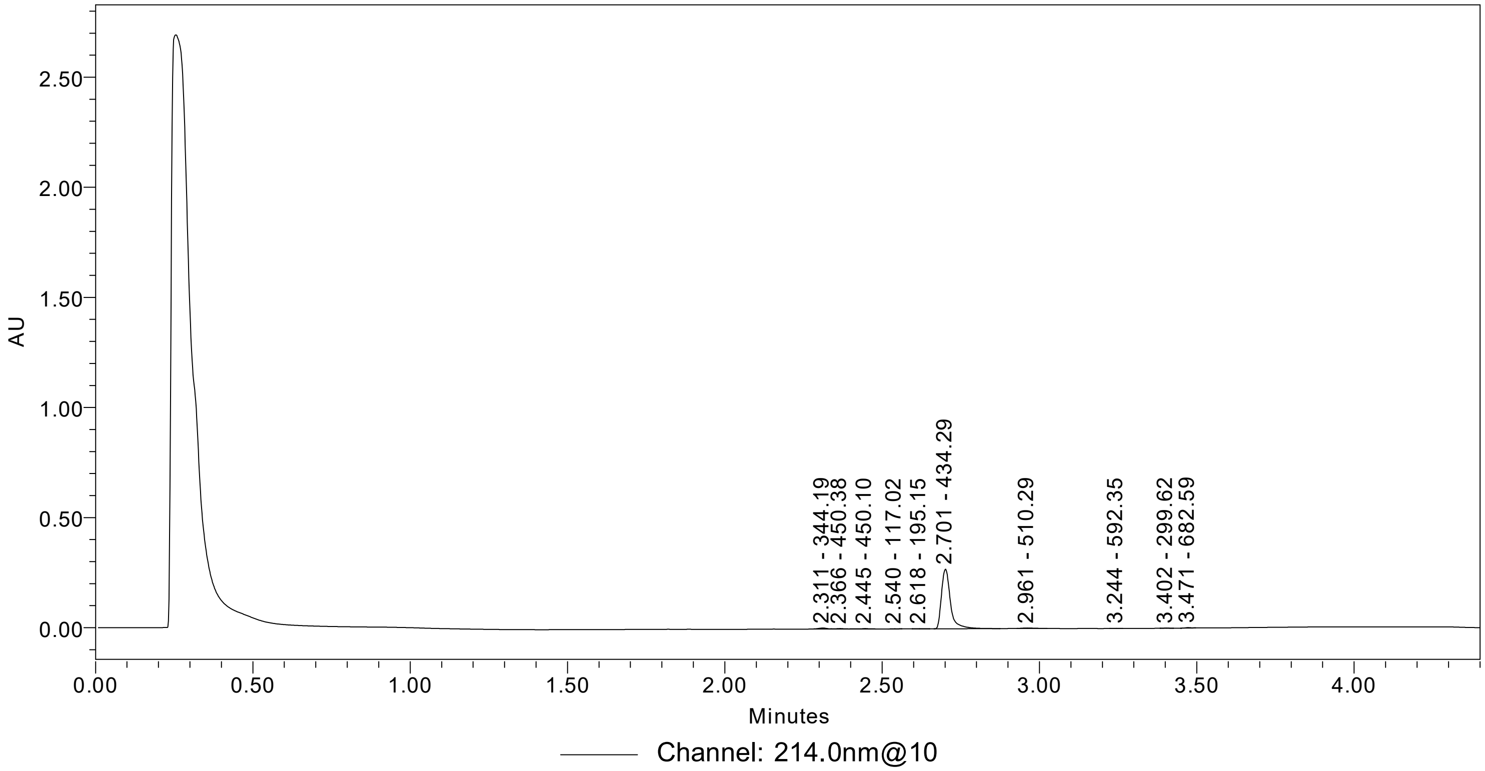

	RT	Area	% Area	Height	Base Peak (m/z)
1	2.311	9054	1.60	4613	344.19
2	2.366	3678	0.65	1621	450.38
3	2.445	3602	0.64	1123	450.10
4	2.540	828	0.15	578	117.02
5	2.618	750	0.13	371	195.15
6	2.701	537481	95.12	270830	434.29
7	2.961	6016	1.06	2037	510.29
8	3.244	392	0.07	306	592.35
9	3.402	909	0.16	726	299.62
10	3.471	2328	0.41	1934	682.59

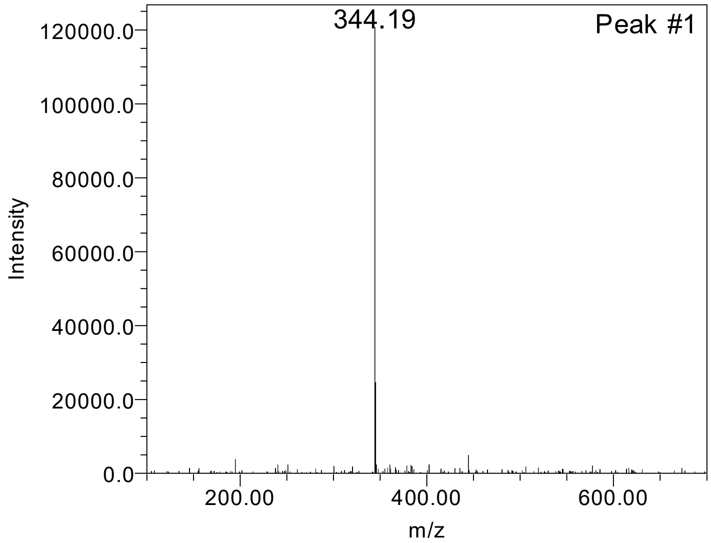

RT: 2.311

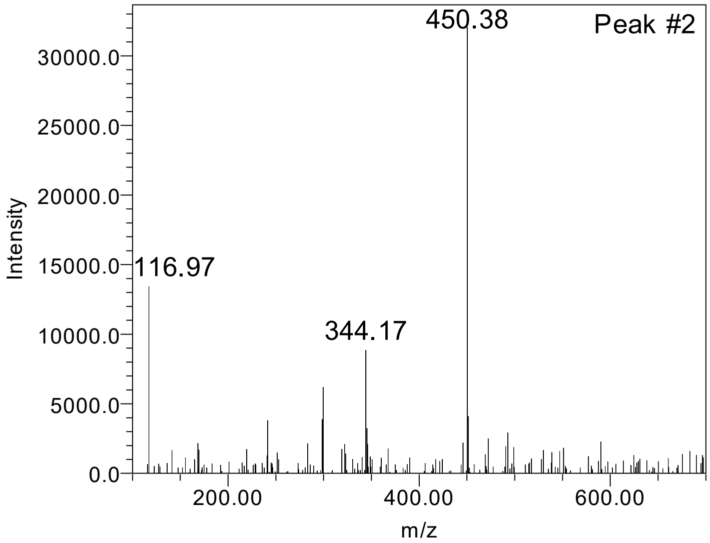

RT: 2.366

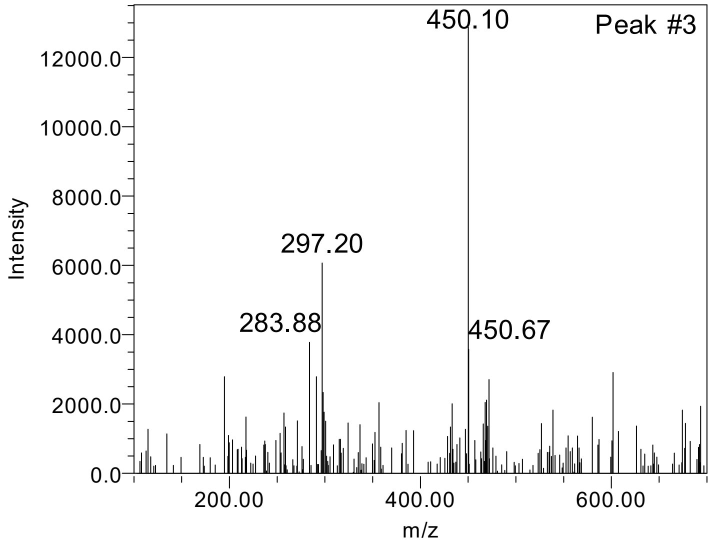

RT: 2.445

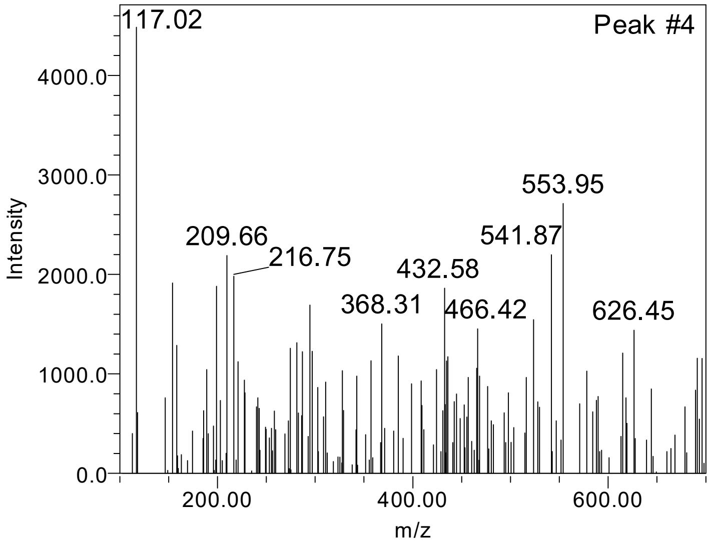

RT: 2.540

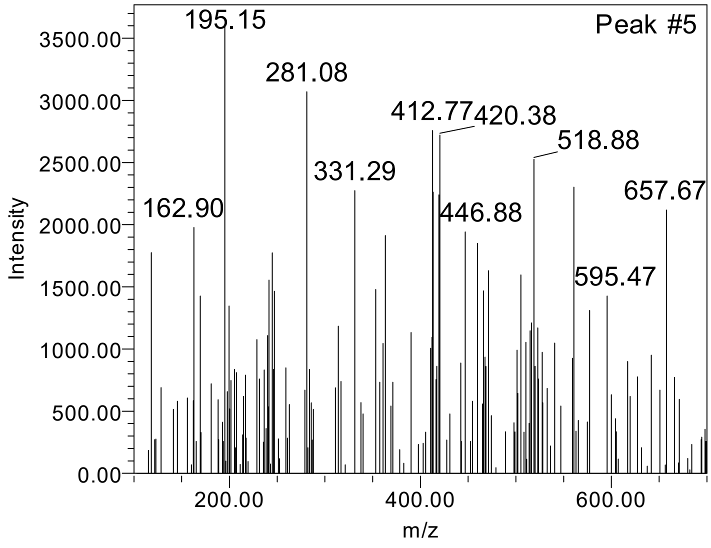

RT: 2.618

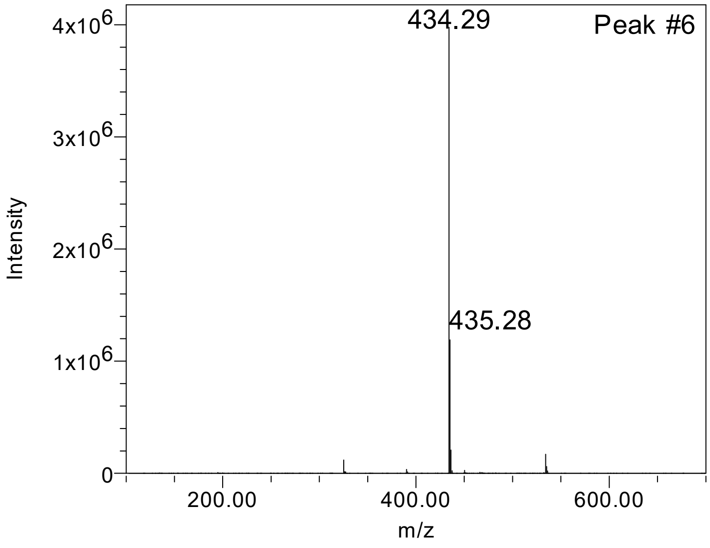

RT: 2.701

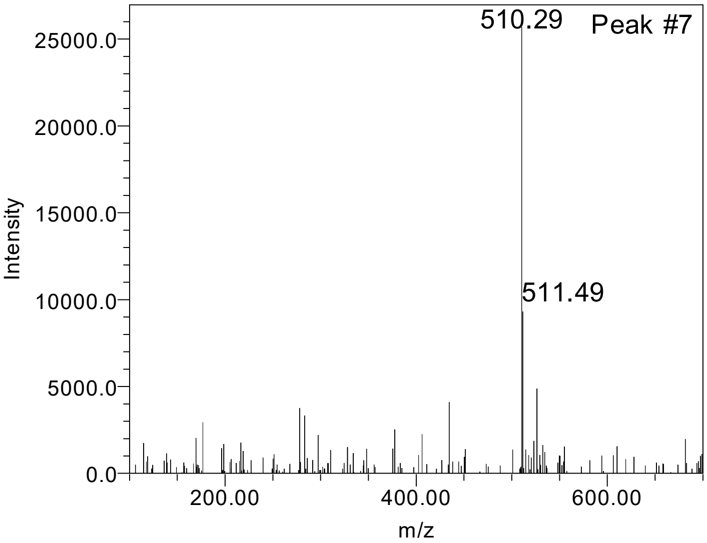

RT: 2.961

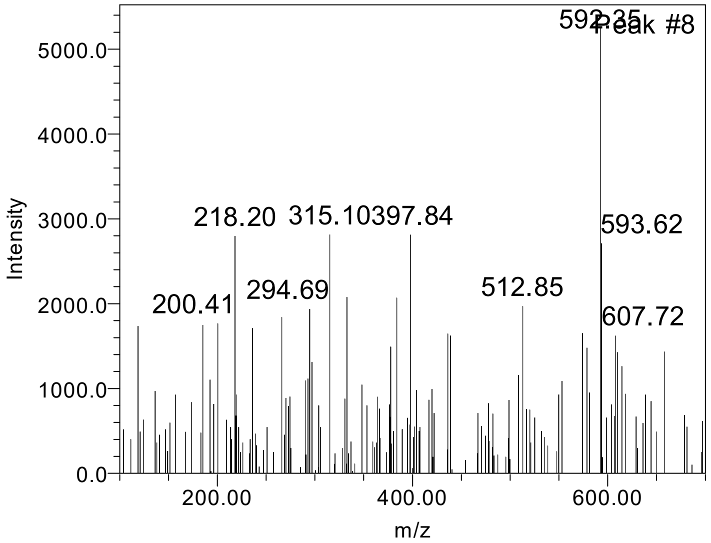

RT: 3.244

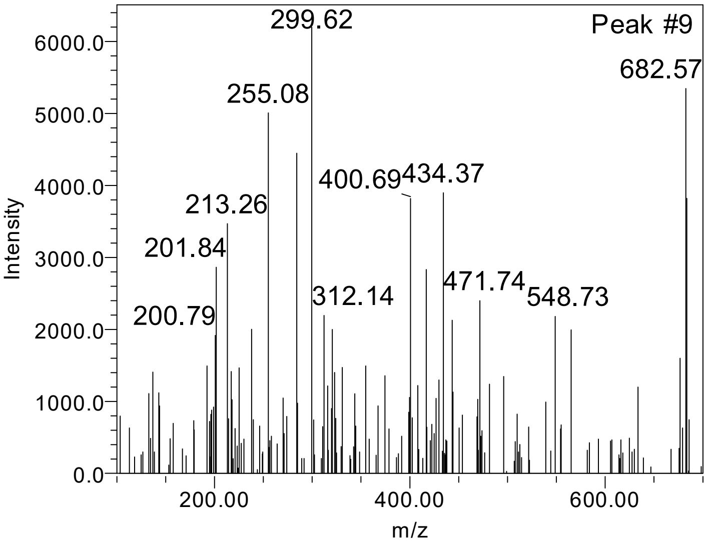

RT: 3.402

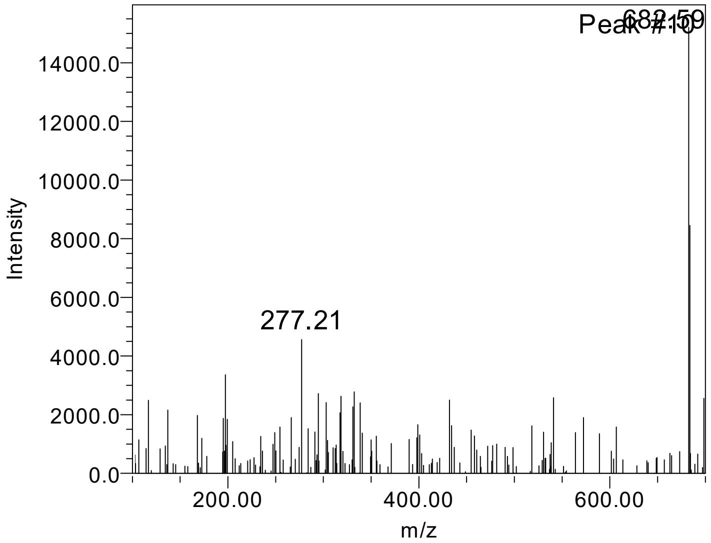

RT: 3.471

Mass Analysis Report

SAMPLE INFORMATION

Sample Name: SR211003B
Acq Method Set: Col1_MeOH_H2O_NH4HCO3
Acquired: 10/14/2021 10:31:25 PM CDT
InjVol: 7.50 uL

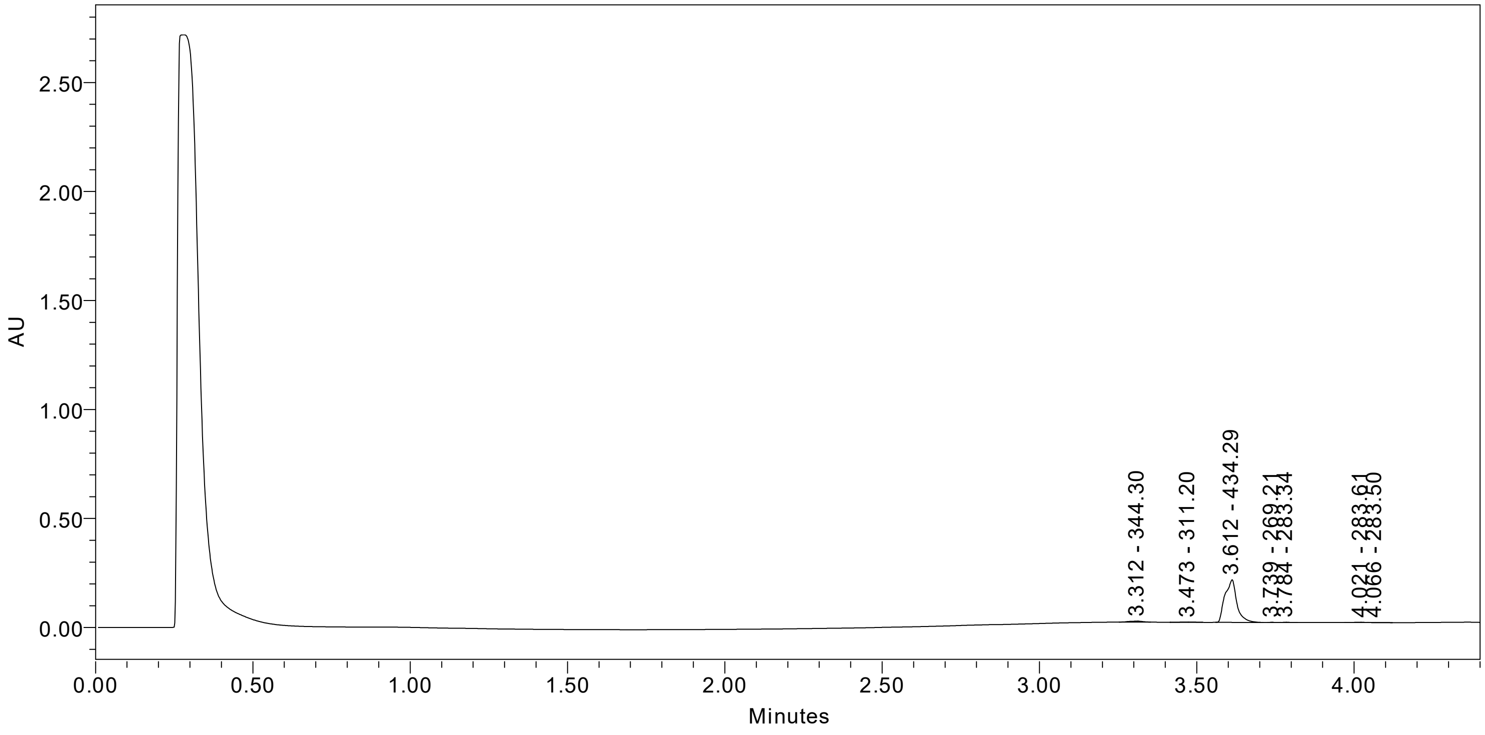

	RT	Area	% Area	Height	Base Peak (m/z)
1	3.312	12625	2.36	4002	344.30
2	3.473	3715	0.69	1149	311.20
3	3.612	511220	95.42	195196	434.29
4	3.739	1079	0.20	587	269.21
5	3.784	1716	0.32	946	283.34
6	4.021	2939	0.55	1201	283.61
7	4.066	2490	0.46	786	283.50

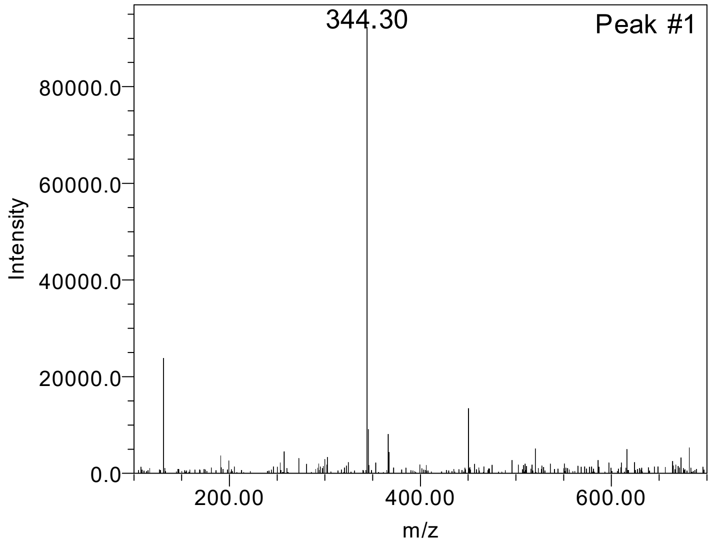

RT: 3.312

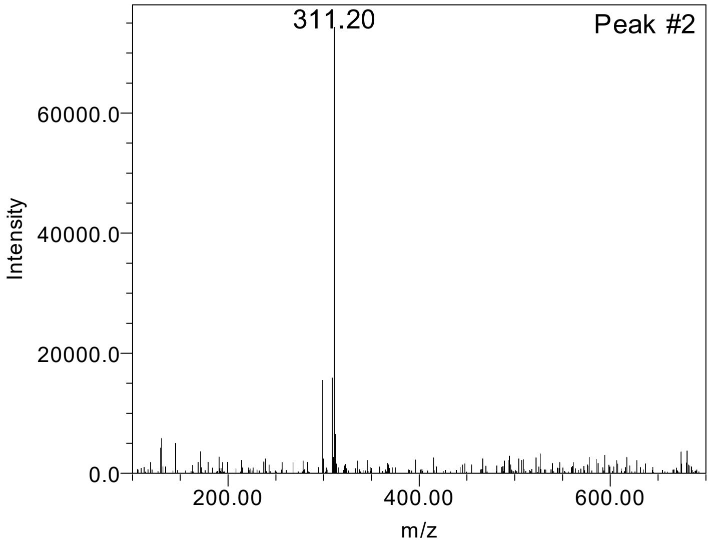

RT: 3.473

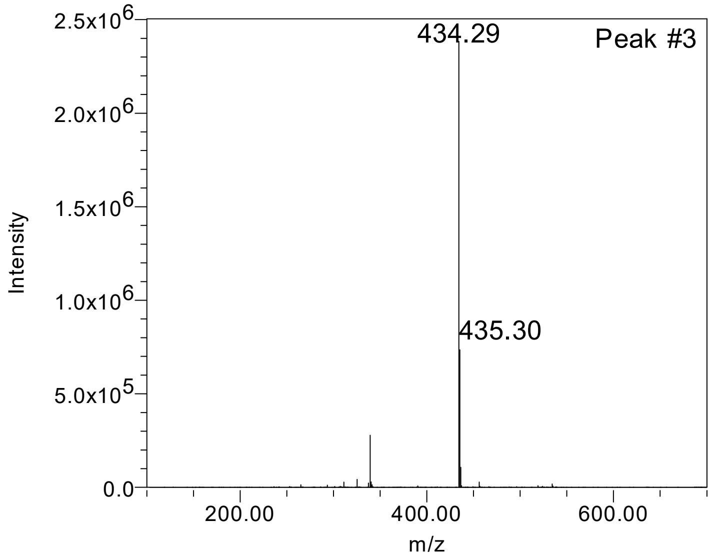

RT: 3.612

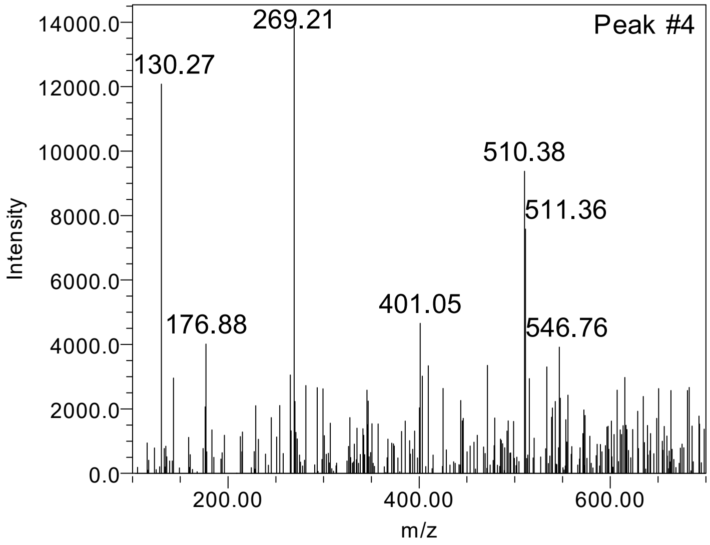

RT: 3.739

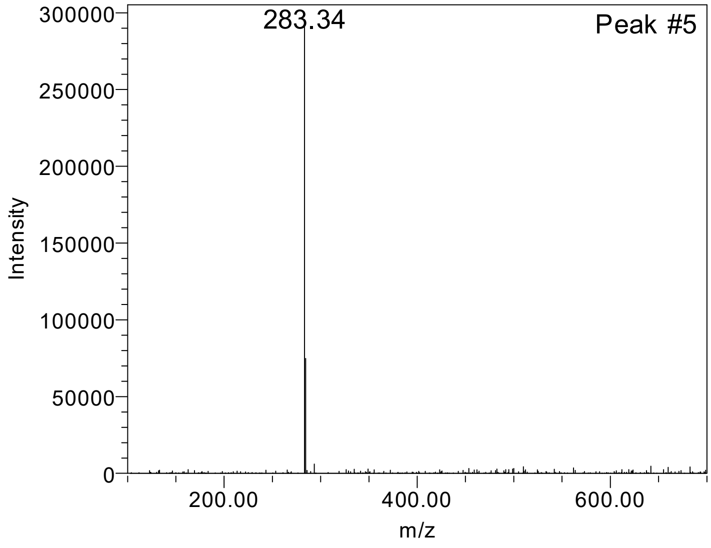

RT: 3.784

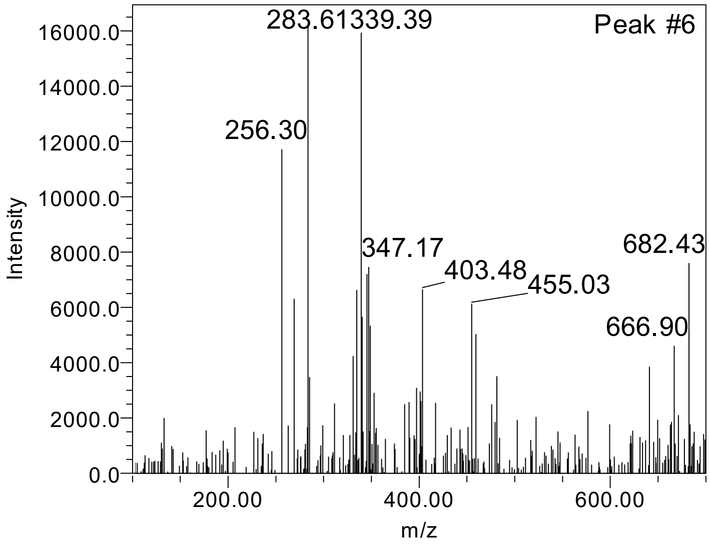

RT: 4.021

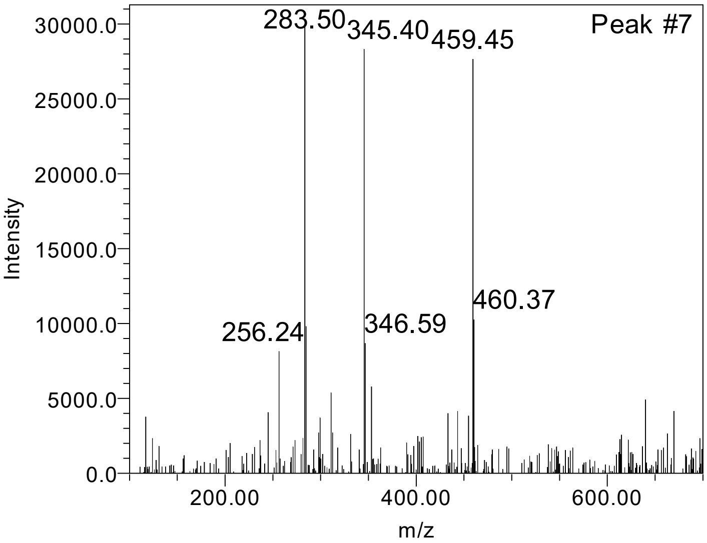

Peak #7
283.50
345.40
459.45
460.37
256.24
346.59
Intensity
30000.0
25000.0
20000.0
15000.0
10000.0
5000.0
0.0
200.00
400.00
600.00
m/z

RT: 4.066

Mass Analysis Report

SAMPLE INFORMATION

Sample Name: SR210331F
Acq Method Set: Col2_MeCN_H2O_NH4HCO3

Acquired: 5/14/2021 6:13:34 PM CDT
InjVol: 7.50 uL

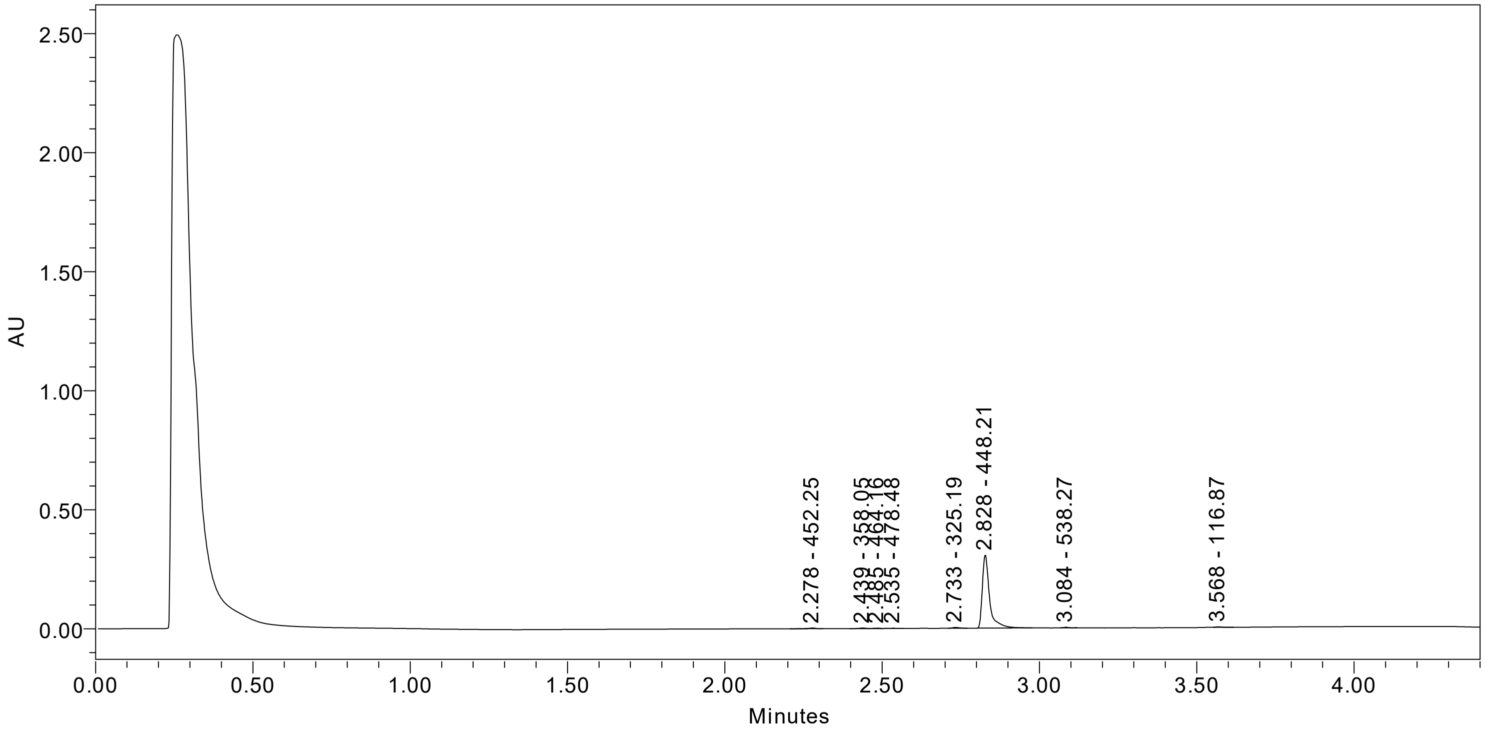

	RT	Area	% Area	Height	Base Peak (m/z)
1	2.278	5148	1.00	2707	452.25
2	2.439	3901	0.76	2309	358.05
3	2.485	2857	0.56	1622	464.16
4	2.535	1233	0.24	723	478.48
5	2.733	4865	0.95	3250	325.19
6	2.828	489664	95.50	305693	448.21
7	3.084	2466	0.48	2152	538.27
8	3.568	2583	0.50	2116	116.87

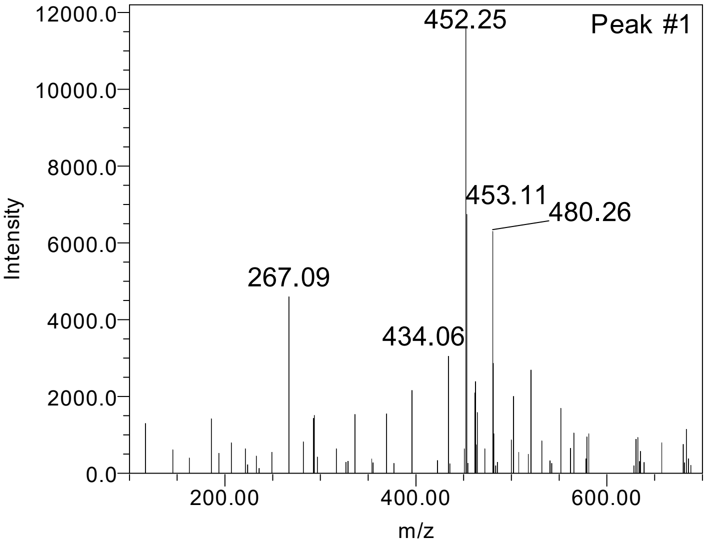

RT: 2.278

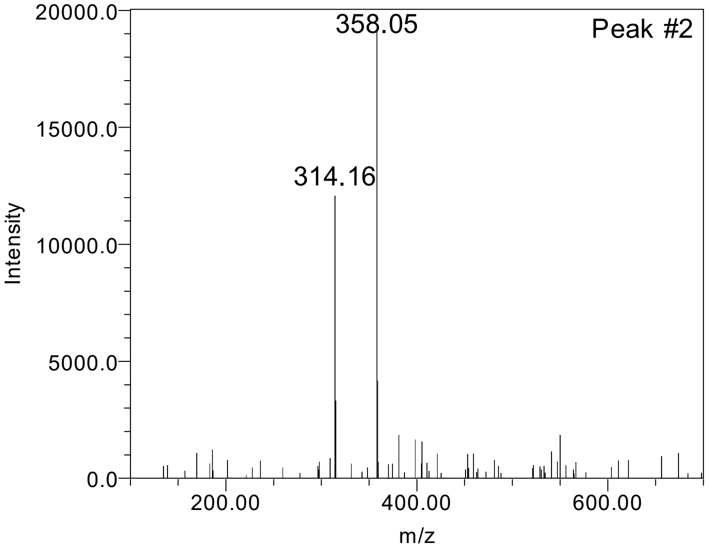

RT: 2.439

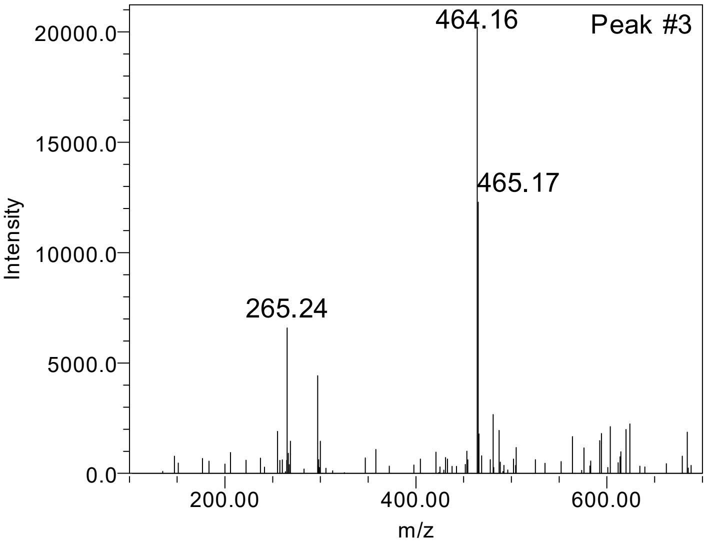

RT: 2.485

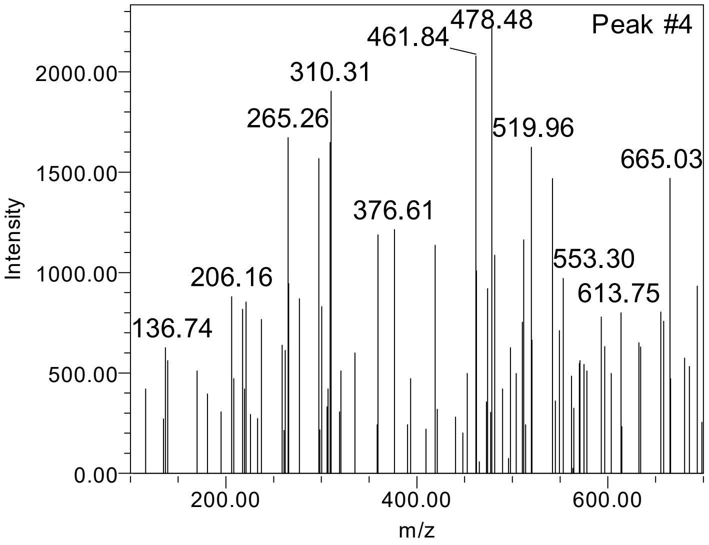

RT: 2.535

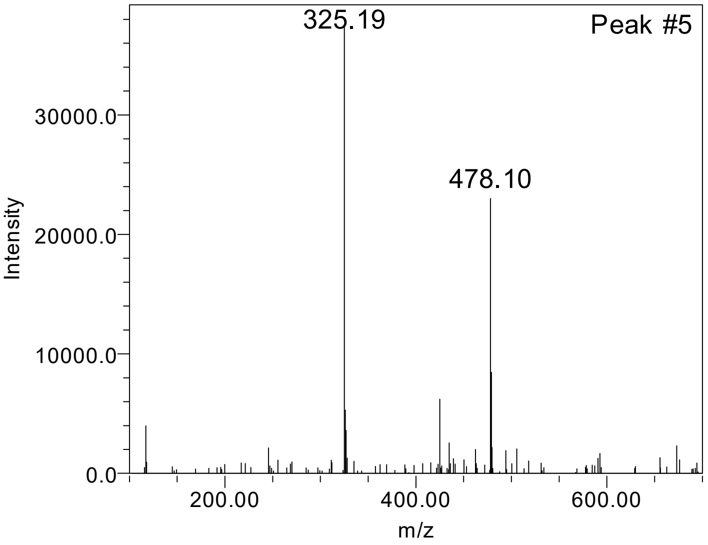

RT: 2.733

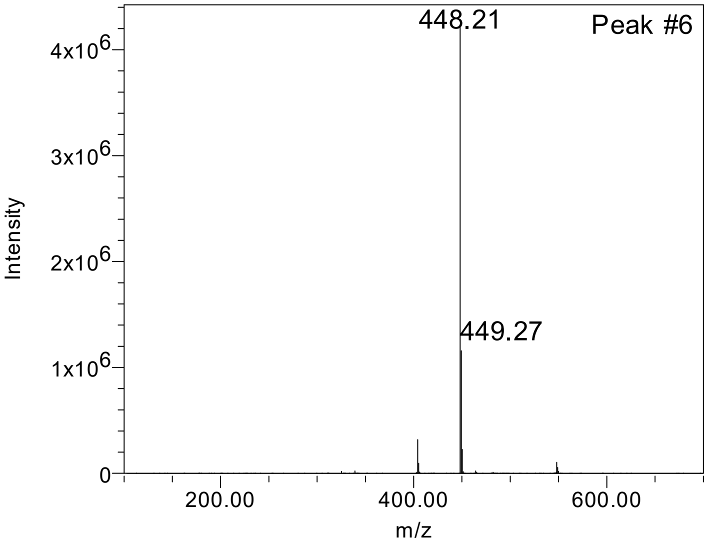

RT: 2.828

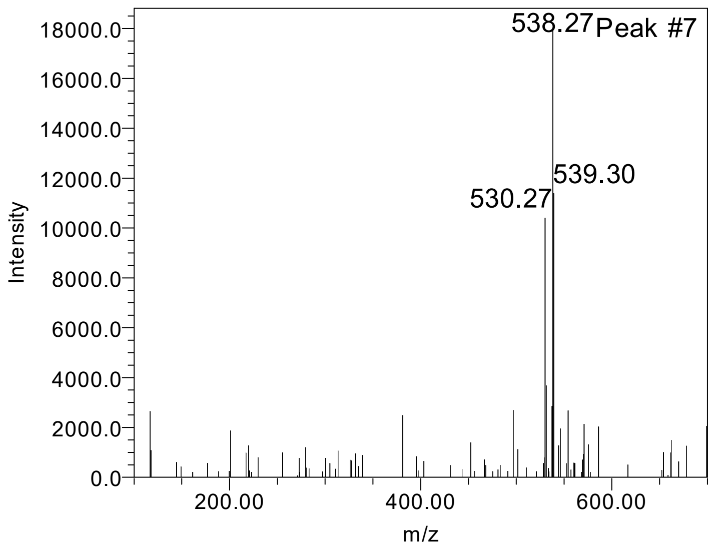

RT: 3.084

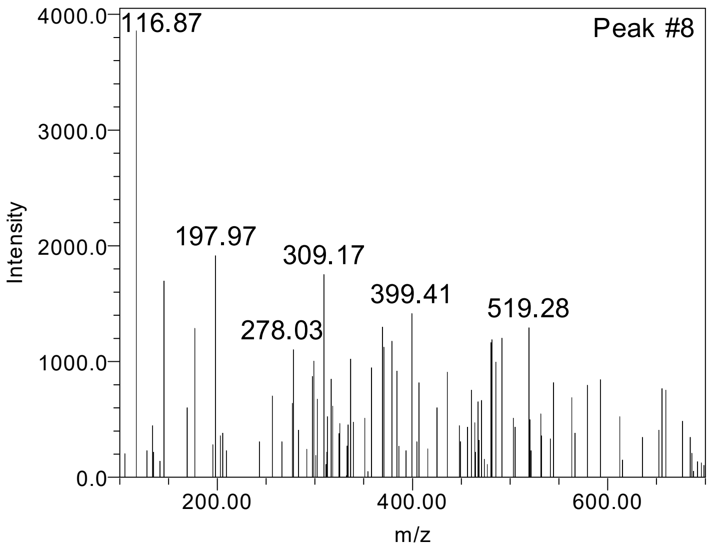

RT: 3.568

Mass Analysis Report

SAMPLE INFORMATION

Sample Name: SR210331F
Acq Method Set: Col1_MeOH_H2O_NH4HCO3
Acquired: 5/15/2021 12:54:07 AM CDT
InjVol: 7.50 uL

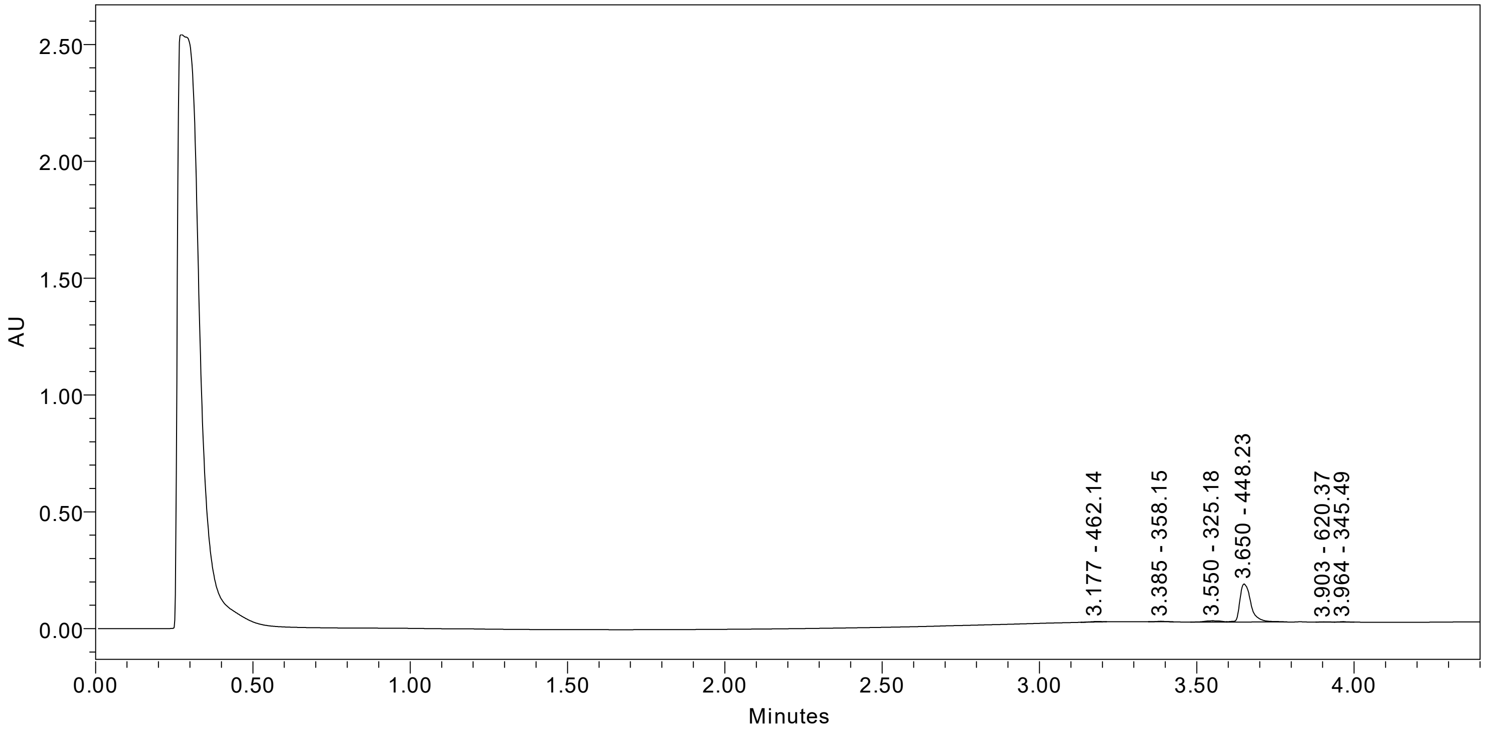

	RT	Area	% Area	Height	Base Peak (m/z)
1	3.177	4192	0.98	1736	462.14
2	3.385	4315	1.01	1826	358.15
3	3.550	19246	4.49	5287	325.18
4	3.650	396163	92.50	162297	448.23
5	3.903	1665	0.39	801	620.37
6	3.964	2725	0.64	1418	345.49

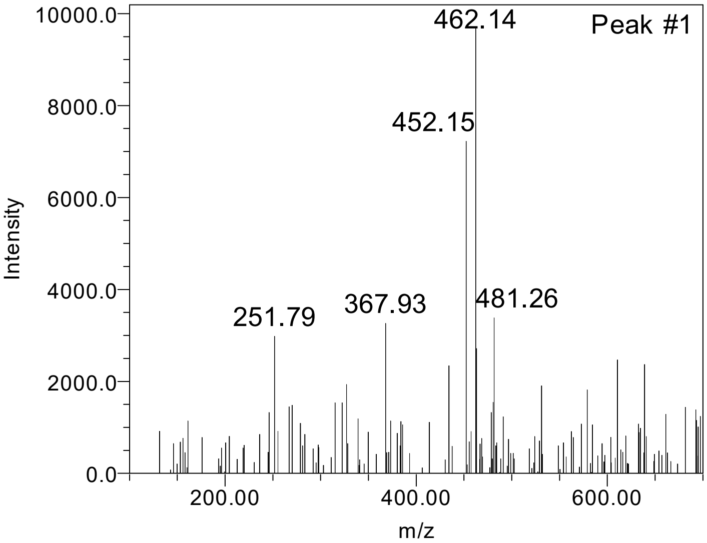

RT: 3.177

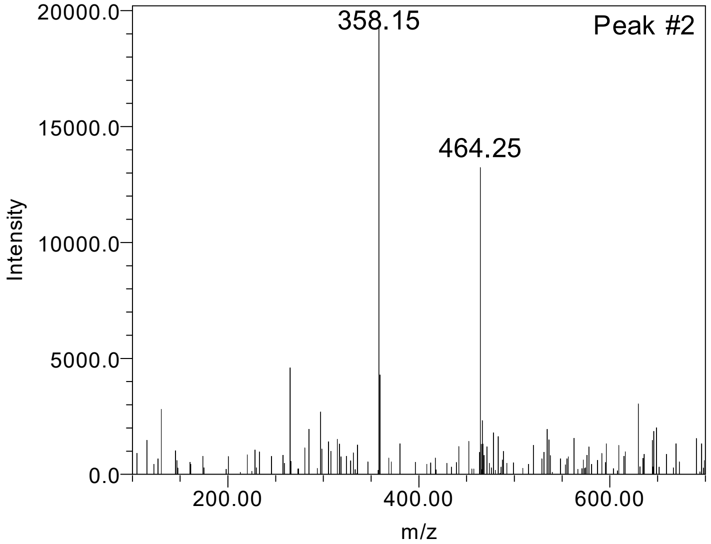

RT: 3.385

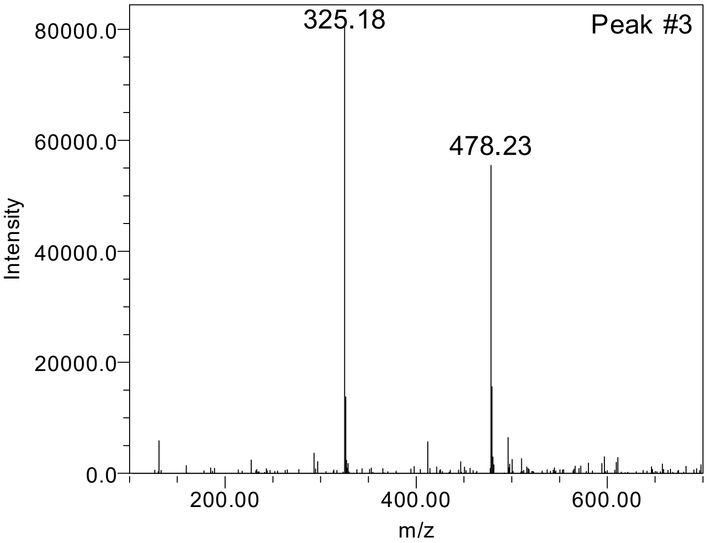

RT: 3.550

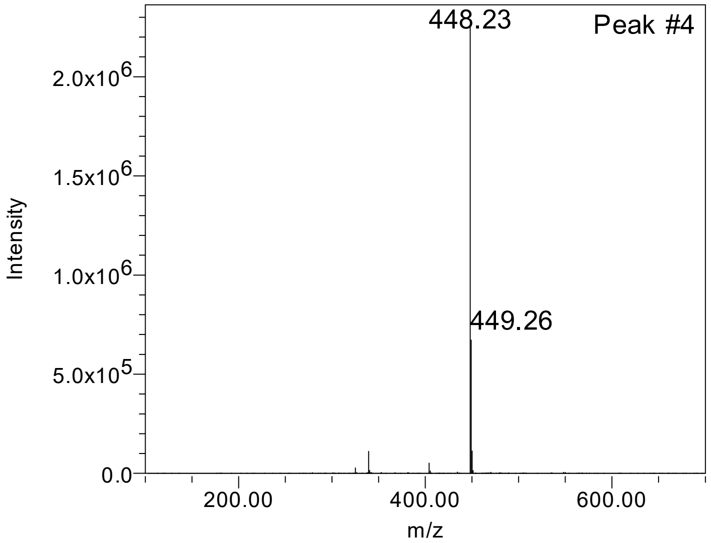

RT: 3.650

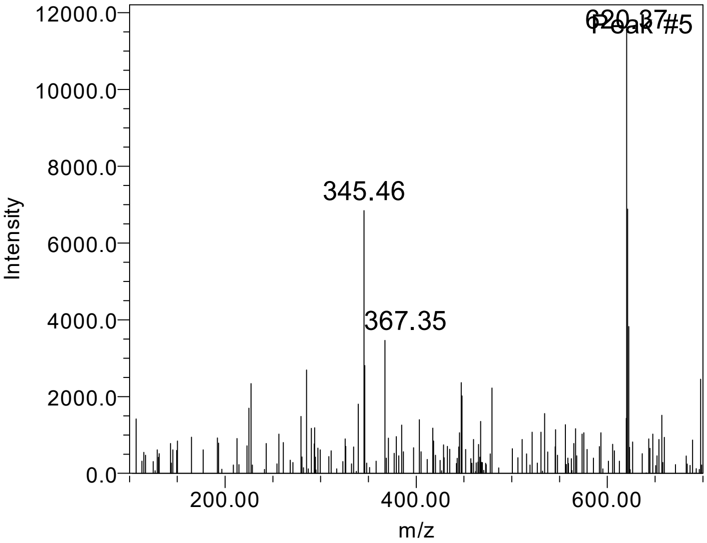

RT: 3.903

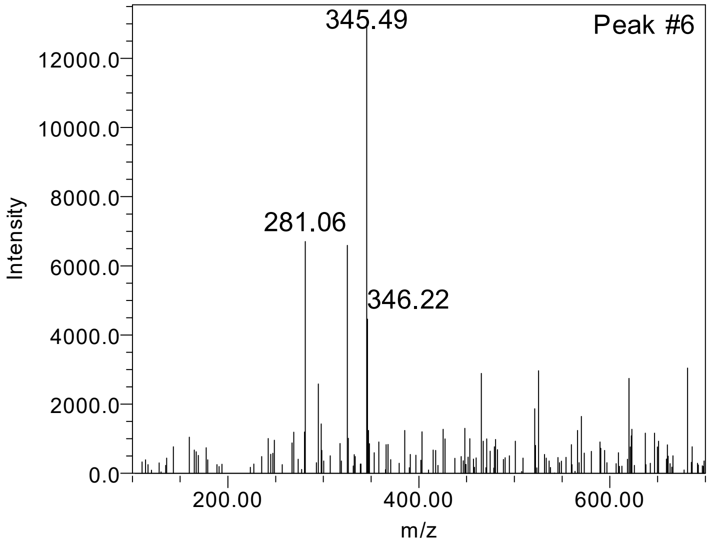

RT: 3.964

Mass Analysis Report

SAMPLE INFORMATION

Sample Name: SR210505A
Acq Method Set: Col2_MeCN_H2O_NH4HCO3
Acquired: 5/14/2021 6:48:58 PM CDT
InjVol: 7.50 uL

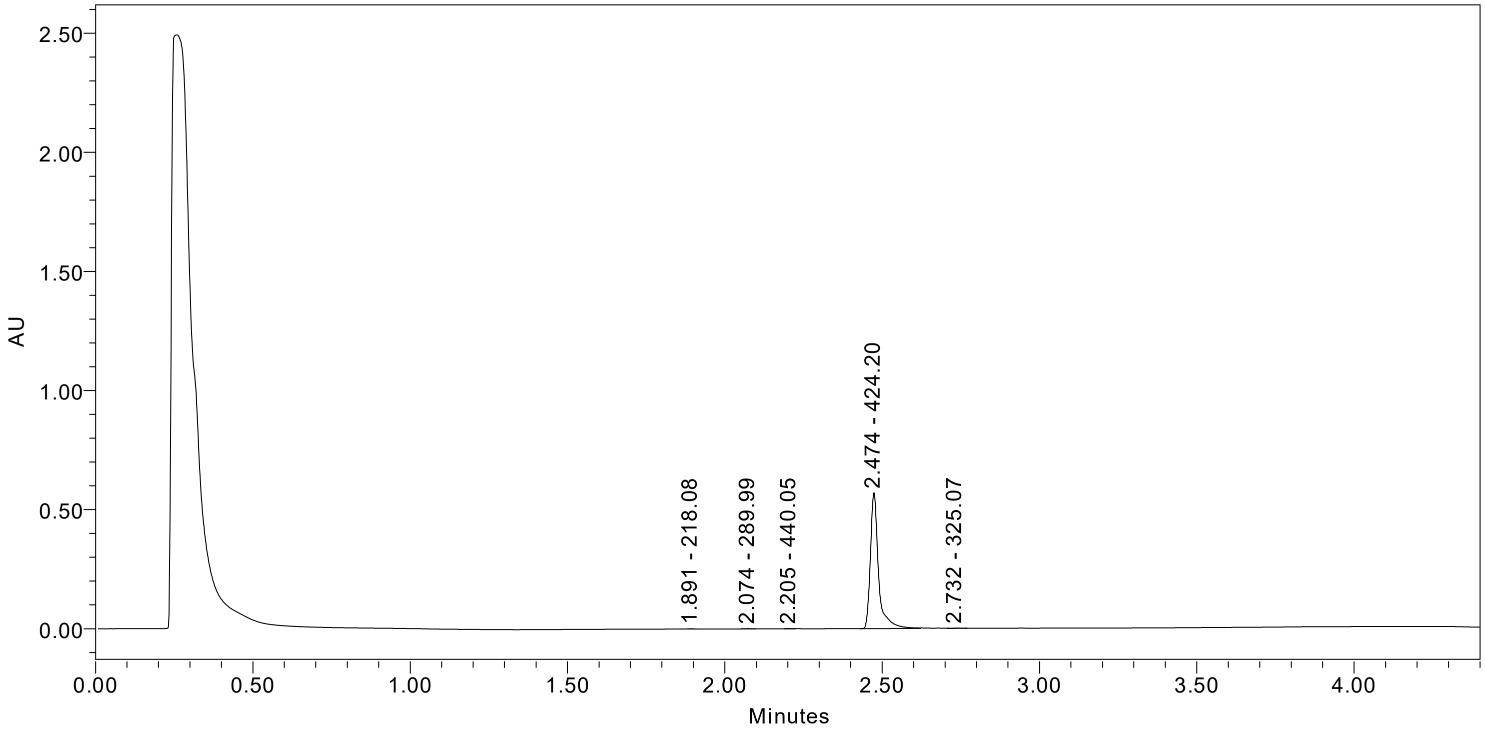

	RT	Area	% Area	Height	Base Peak (m/z)
1	1.891	1852	0.18	1211	218.08
2	2.074	820	0.08	726	289.99
3	2.205	725	0.07	573	440.05
4	2.474	1029592	99.52	570090	424.20
5	2.732	1613	0.16	724	325.07

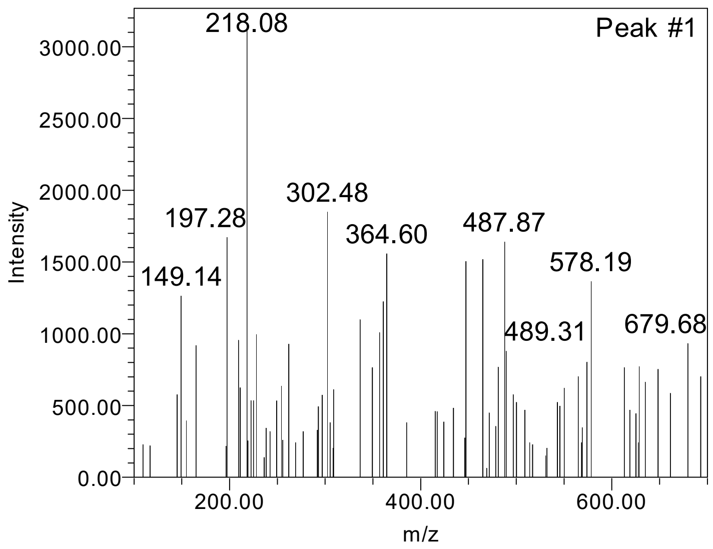

RT: 1.891

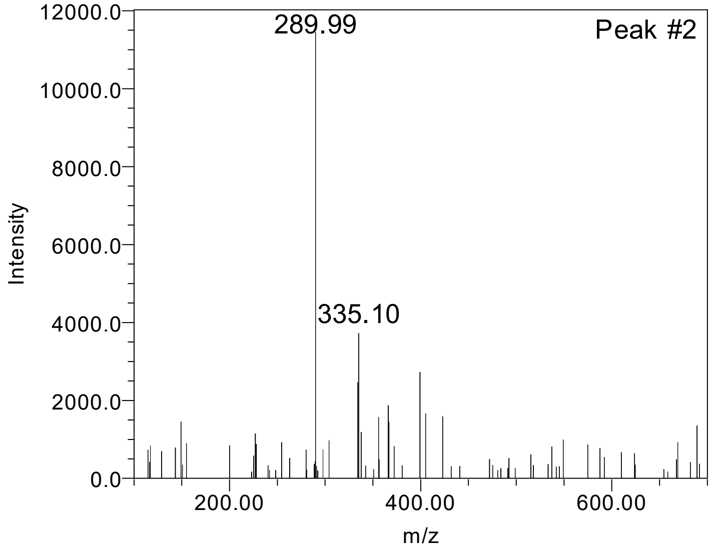

RT: 2.074

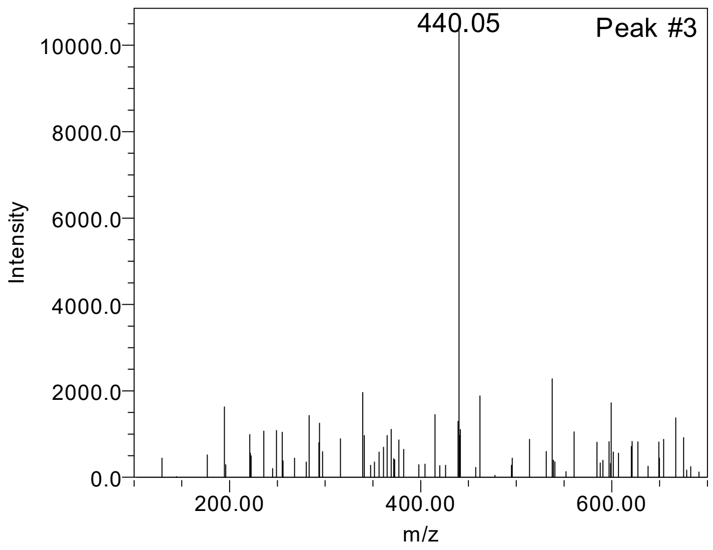

RT: 2.205

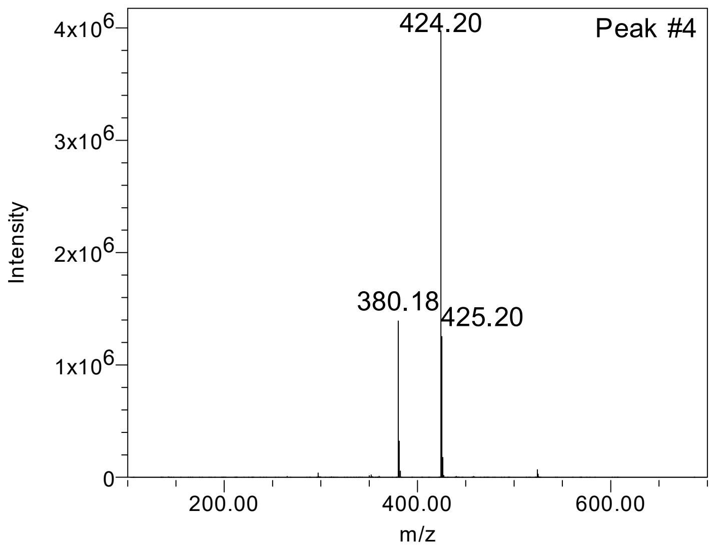

RT: 2.474

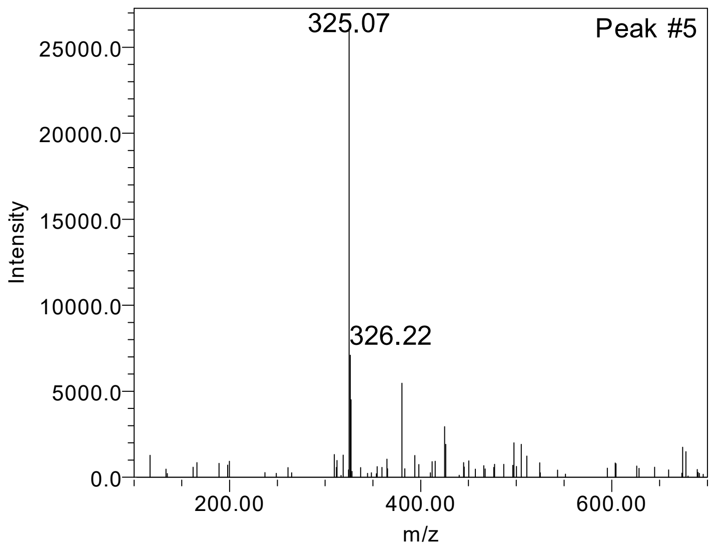

RT: 2.732

Mass Analysis Report

SAMPLE INFORMATION

Sample Name: SR210505A
Acq Method Set: Col1_MeOH_H2O_NH4HCO3
Acquired: 5/15/2021 1:29:32 AM CDT
InjVol: 7.50 uL

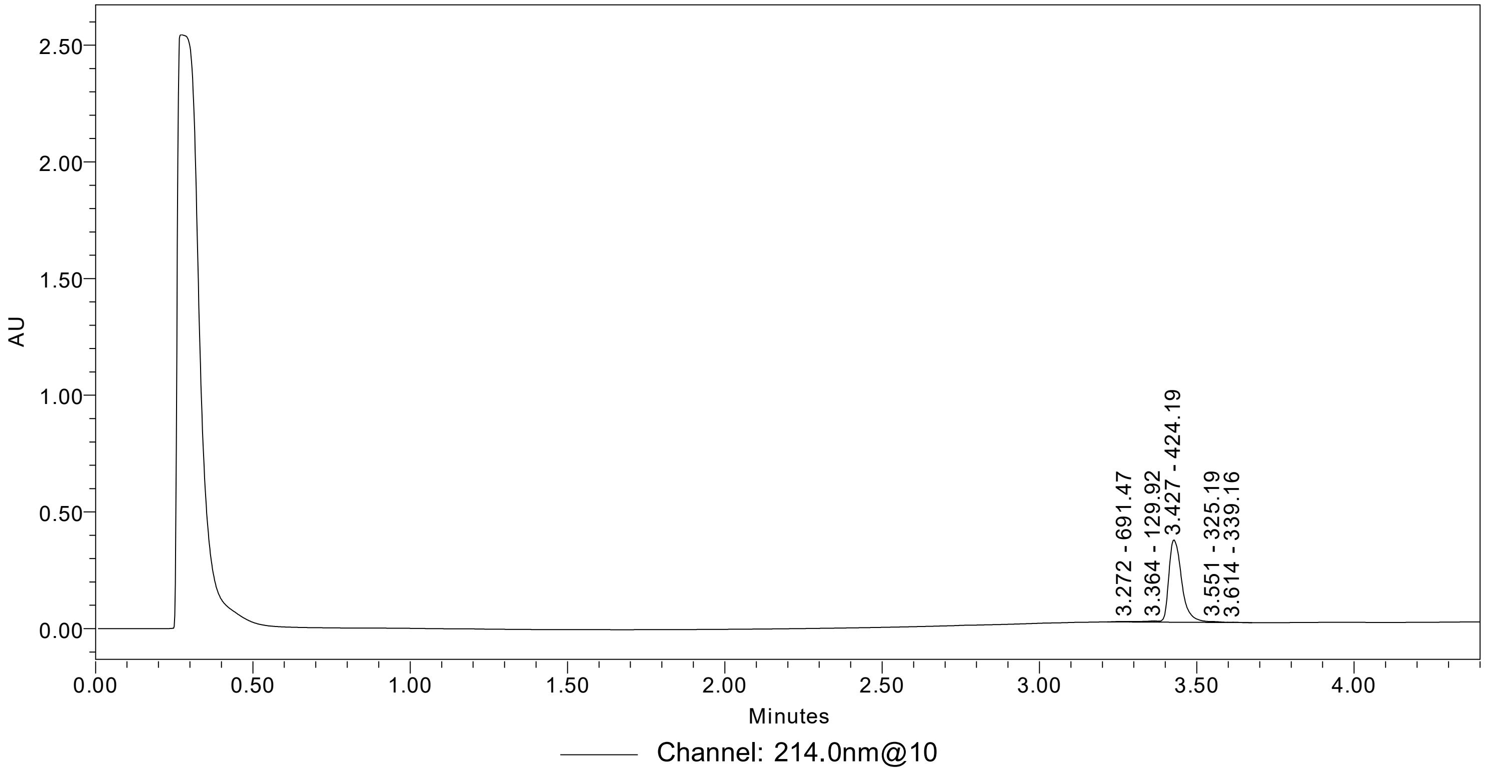

	RT	Area	% Area	Height	Base Peak (m/z)
1	3.272	5834	0.56	2090	691.47
2	3.364	18354	1.76	4958	129.92
3	3.427	1007021	96.76	352187	424.19
4	3.551	6682	0.64	3517	325.19
5	3.614	2849	0.27	1179	339.16

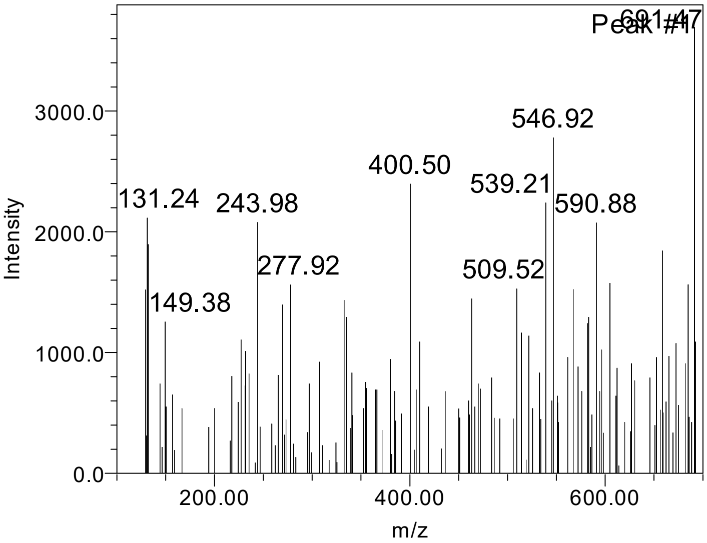

RT: 3.272

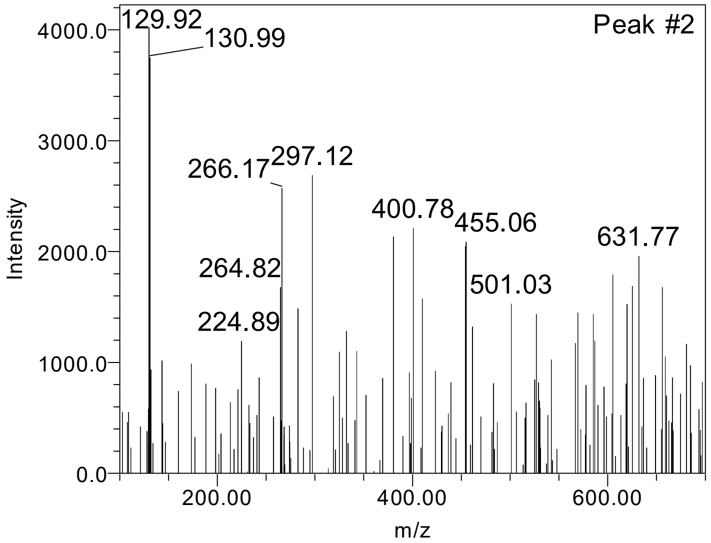

RT: 3.364

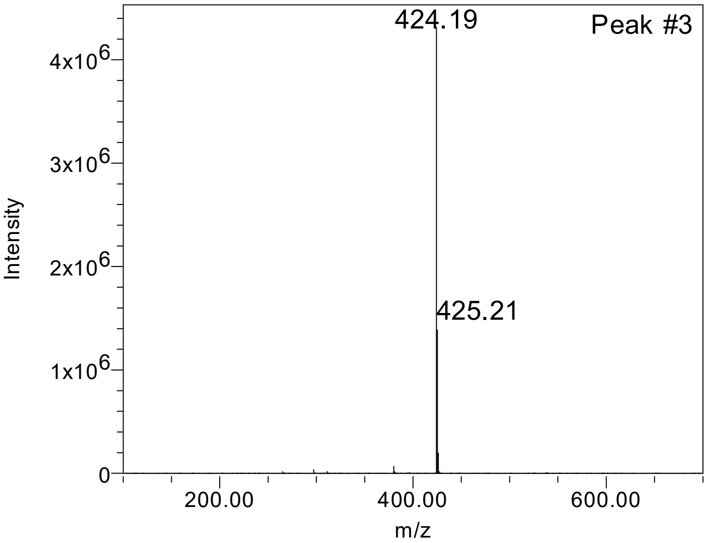

RT: 3.427

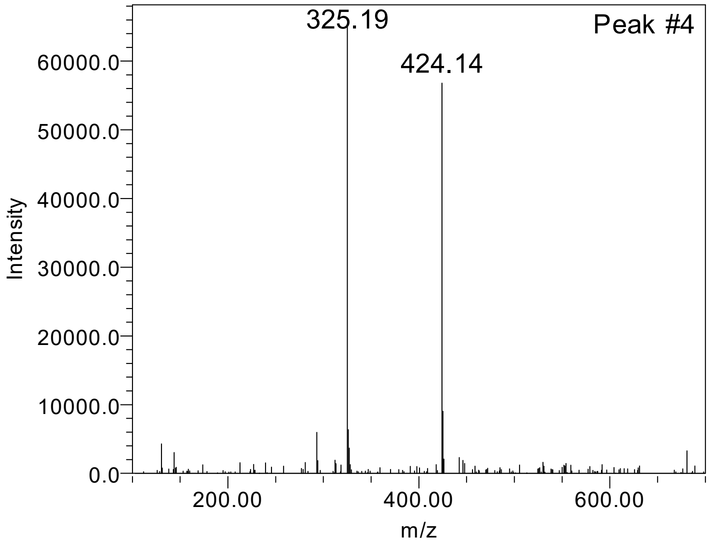

RT: 3.551

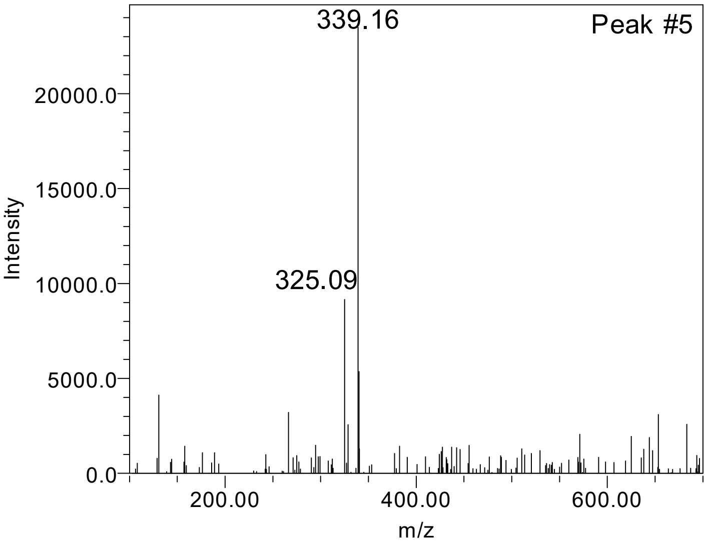

RT: 3.614

Mass Analysis Report

SAMPLE INFORMATION

Sample Name: SR210813C
Acq Method Set: Col2_MeCN_H2O_NH4HCO3
Acquired: 10/14/2021 6:04:40 PM CDT
InjVol: 7.50 uL

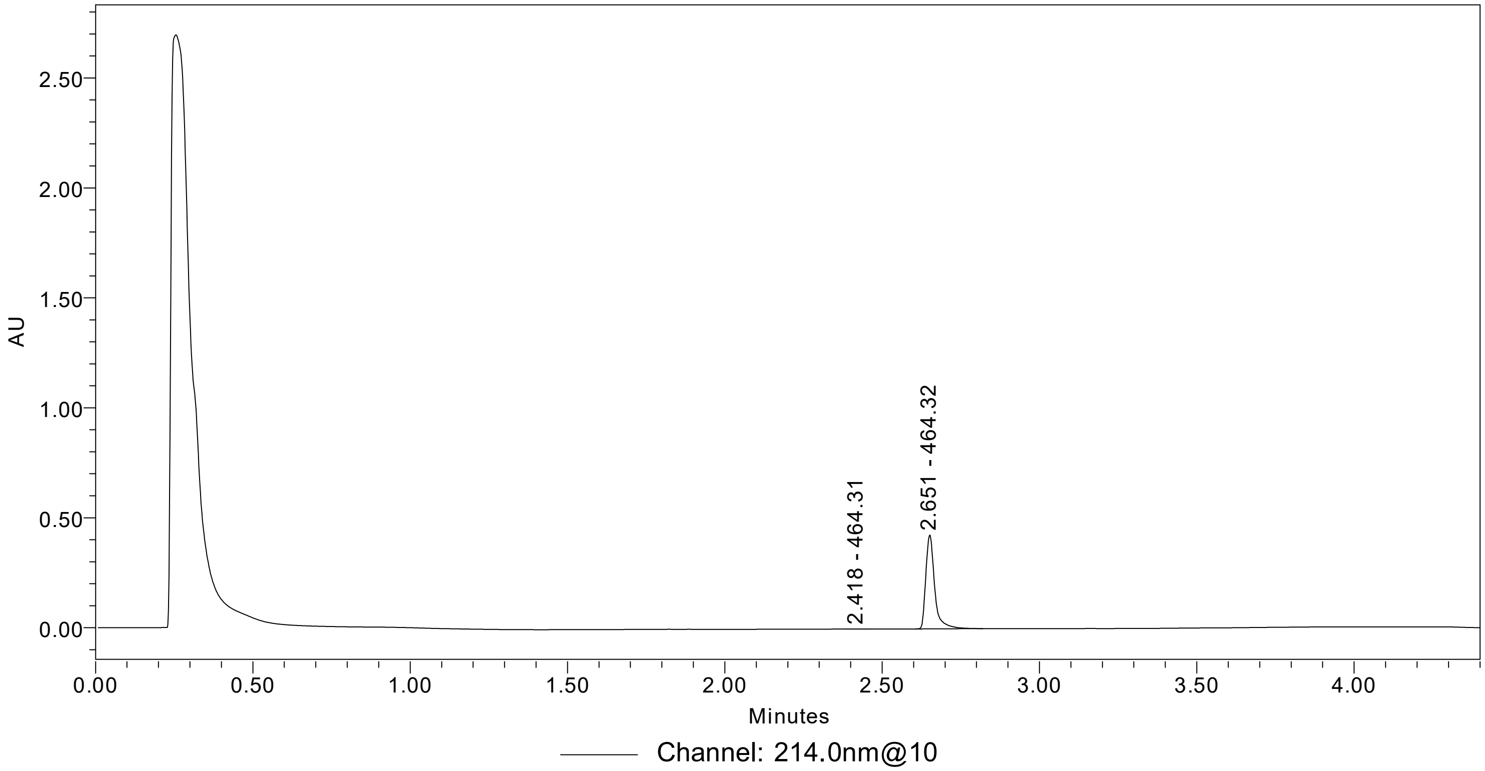

	RT	Area	% Area	Height	Base Peak (m/z)
1	2.418	323	0.04	150	464.31
2	2.651	837378	99.96	426267	464.32

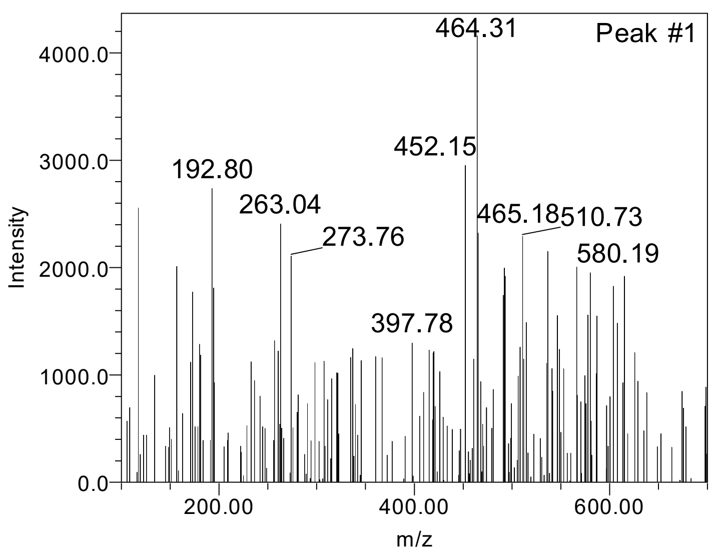

RT: 2.418

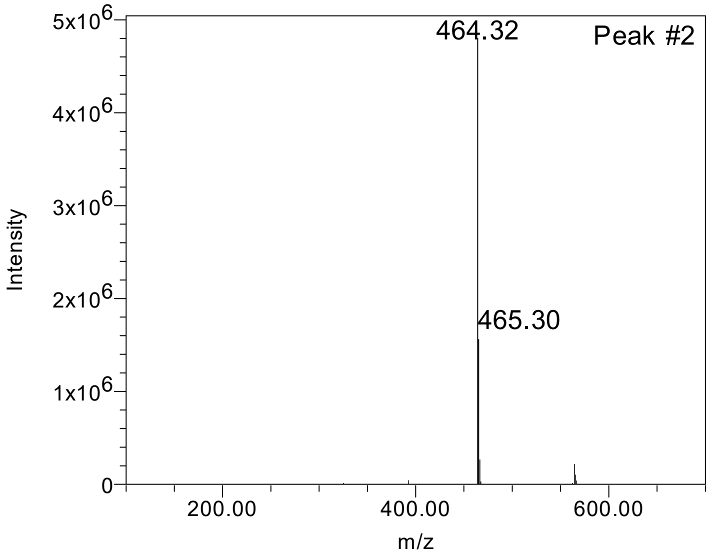

RT: 2.651

Mass Analysis Report

SAMPLE INFORMATION

Sample Name: SR210813C
Acq Method Set: Col1_MeOH_H2O_NH4HCO3
Acquired: 10/14/2021 10:17:13 PM CDT
InjVol: 7.50 uL

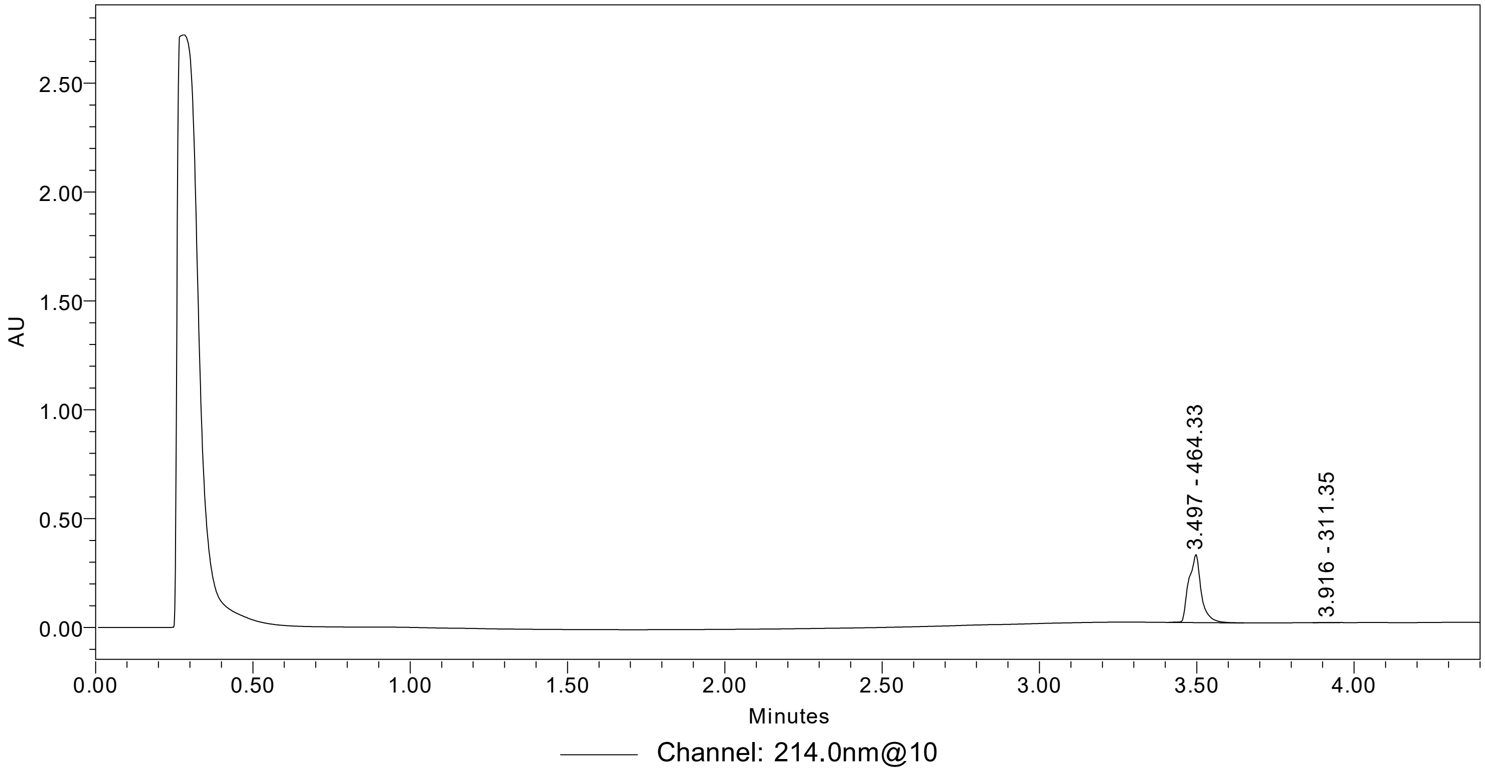

	RT	Area	% Area	Height	Base Peak (m/z)
1	3.497	856575	99.78	311547	464.33
2	3.916	1875	0.22	700	311.35

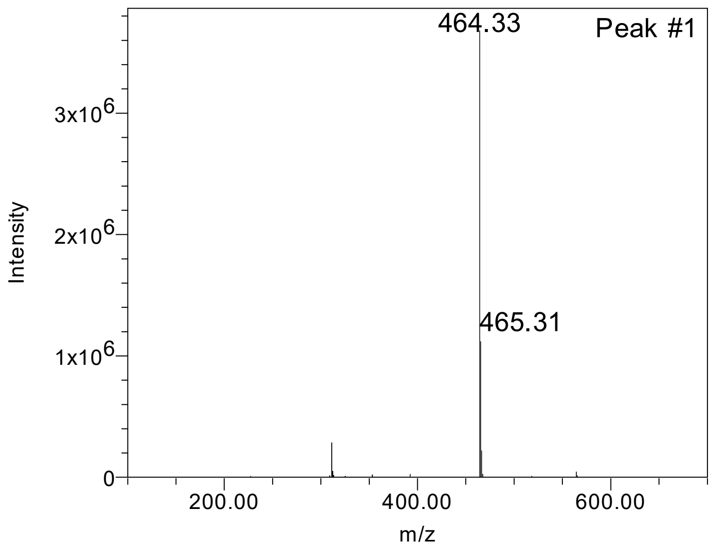

RT: 3.497

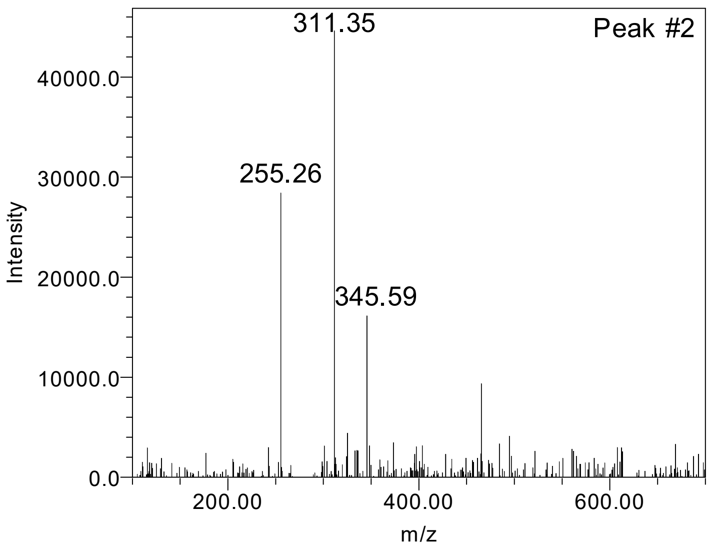

RT: 3.916

Single Injection Report

**Sample name:**	SR230210A		
**Description:**			
**Sample amount:**	0.000	**Sample type:**	Sample
**Instrument:**	LCMS	**Location:**	P1-A2
		**Injection:**	1 of 1
**Acq. method:**	Regular method.amx	**Injection volume:**	5.000 µL
**Analysis method:**	MS method-purity.pmx	**Acq. operator:**	SYSTEM

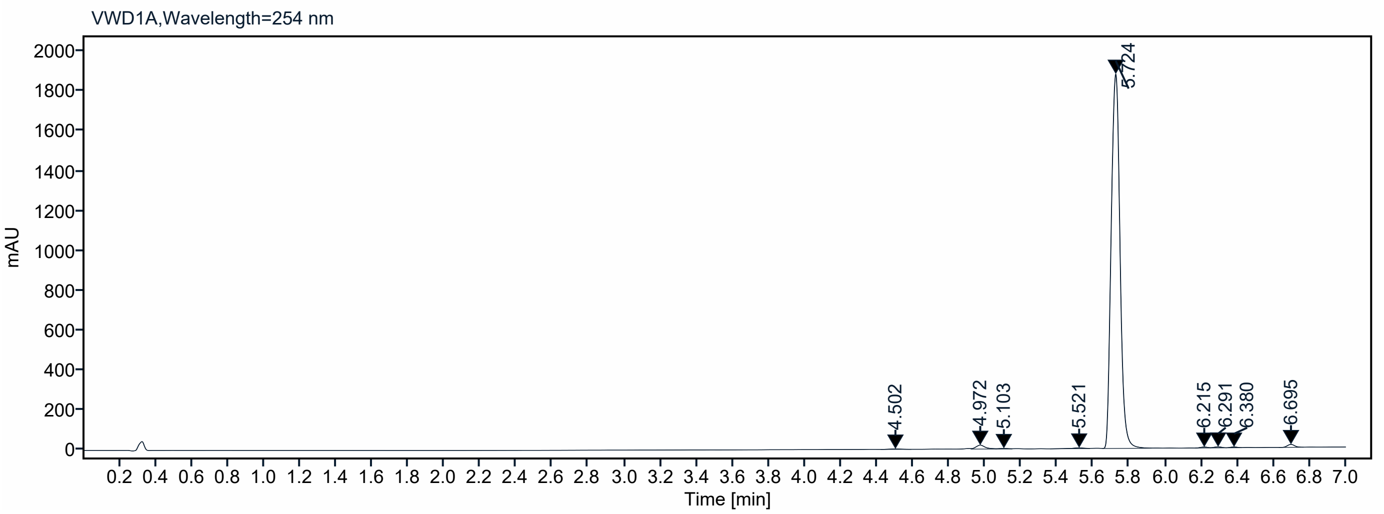

Signal: MS1 +TIC SCAN ESI Frag=110V Gain=1.0

RT [min]	Width [min]	Area	Height	Area%
5.789	0.4761	942264.8178	113387.6446	100.0000
	**Sum**	**942264.8178**		

Signal: VWD1A,Wavelength=254 nm

RT [min]	Width [min]	Area	Height	Area%
4.502	0.1680	10.4476	2.0960	0.1587
4.972	0.1396	70.8998	19.6506	1.0770
5.103	0.0892	11.2670	3.0487	0.1712
5.521	0.2198	20.2703	3.9737	0.3079
5.724	0.2705	6387.5021	1876.4824	97.0325
6.215	0.0863	13.7792	4.8123	0.2093
6.291	0.0864	15.7751	5.1621	0.2396
6.380	0.0872	11.3065	3.9536	0.1718
6.695	0.1380	41.6019	14.8066	0.6320
	**Sum**	**6582.8495**		

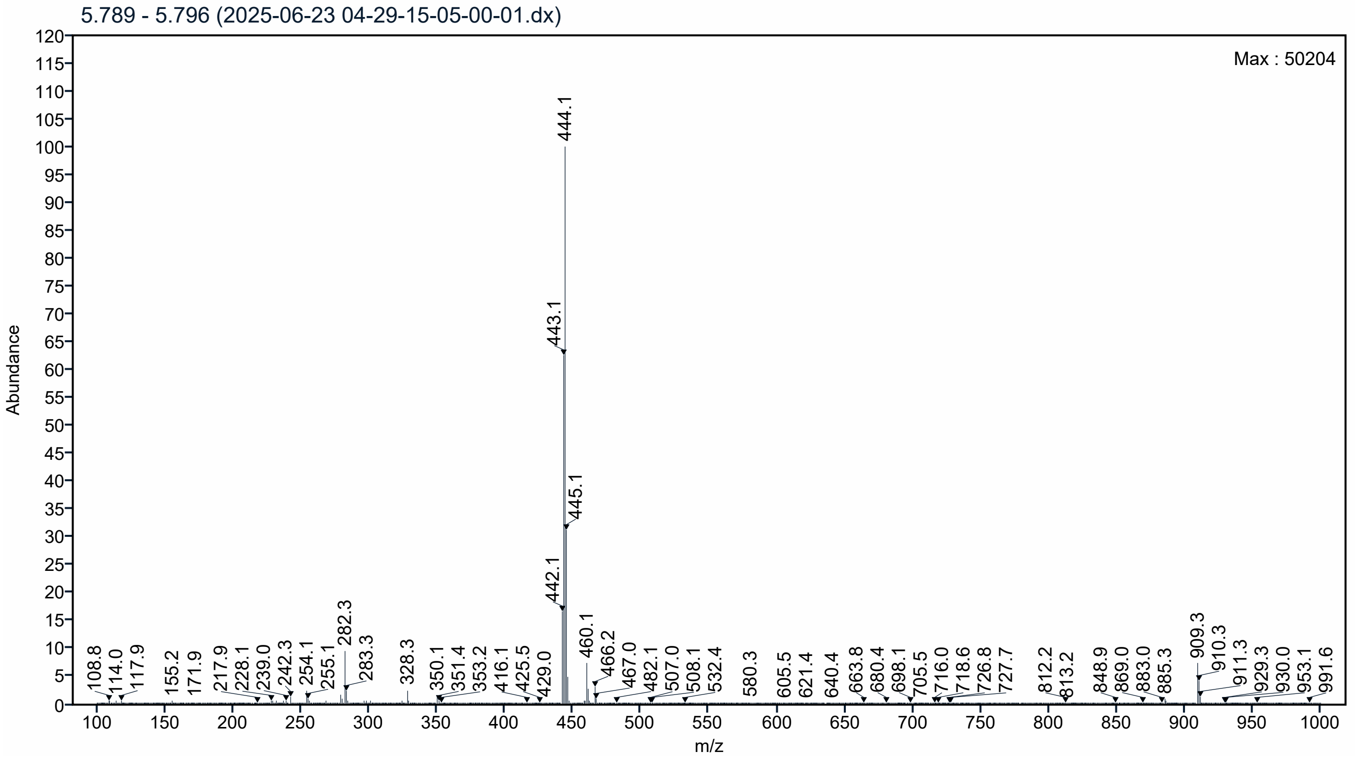
5.789 - 5.796 (2025-06-23 04-29-15-05-00-01.dx)
Max : 50204
Abundance
m/z
444.1
443.1
445.1
442.1
282.3
283.3
328.3
460.1
466.2
909.3
910.3

Single Injection Report

**Sample name:**	SR231215C		
**Description:**			
**Sample amount:**	0.000	**Sample type:**	Sample
**Instrument:**	LCMS	**Location:**	P1-A3
		**Injection:**	1 of 1
**Acq. method:**	Regular method.amx	**Injection volume:**	5.000 µL
**Analysis method:**	MS method-purity.pmx	**Acq. operator:**	SYSTEM

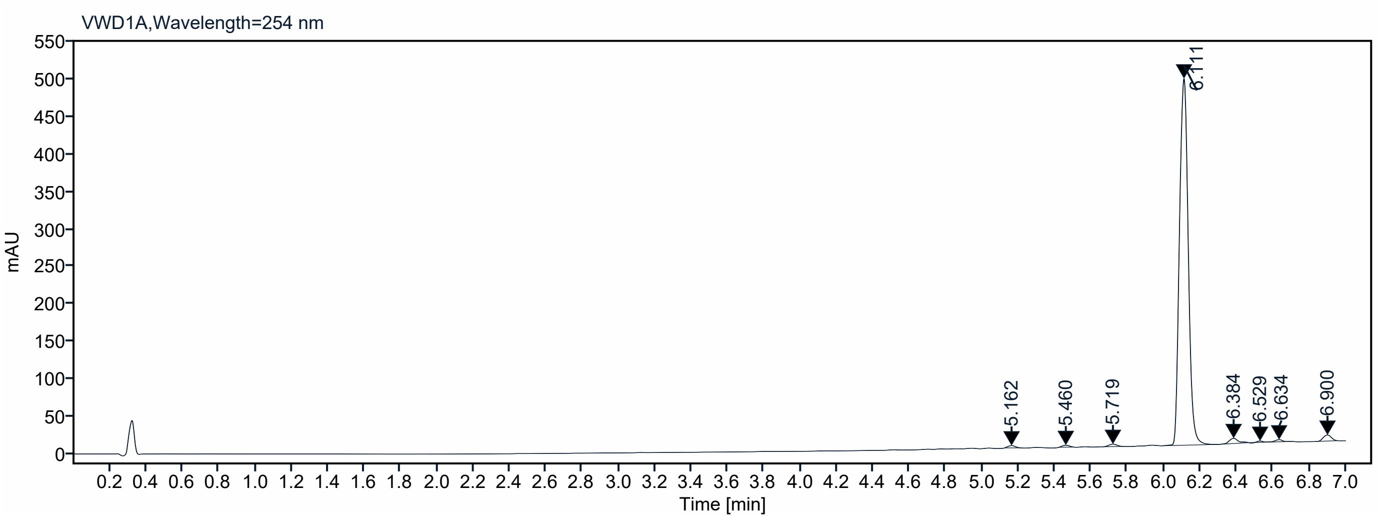

Signal: MS1 +TIC SCAN ESI Frag=110V Gain=1.0

RT [min]	Width [min]	Area	Height	Area%
6.181	0.2422	1303049.35 51	215802.8611	100.0000
	**Sum**	**1303049.35 51**		

Signal: VWD1A,Wavelength=254 nm

RT [min]	Width [min]	Area	Height	Area%
5.162	0.0877	8.4505	3.0427	0.4870
5.460	0.0797	7.0667	2.5896	0.4073
5.719	0.1116	10.1780	3.2766	0.5866
6.111	0.3079	1649.6819	490.1608	95.0763
6.384	0.1681	23.9664	6.9278	1.3813
6.529	0.0967	2.8173	1.7093	0.1624
6.634	0.1345	7.9776	2.8571	0.4598
6.900	0.1116	24.9755	8.0766	1.4394
	**Sum**	**1735.1138**		

Single Injection Report

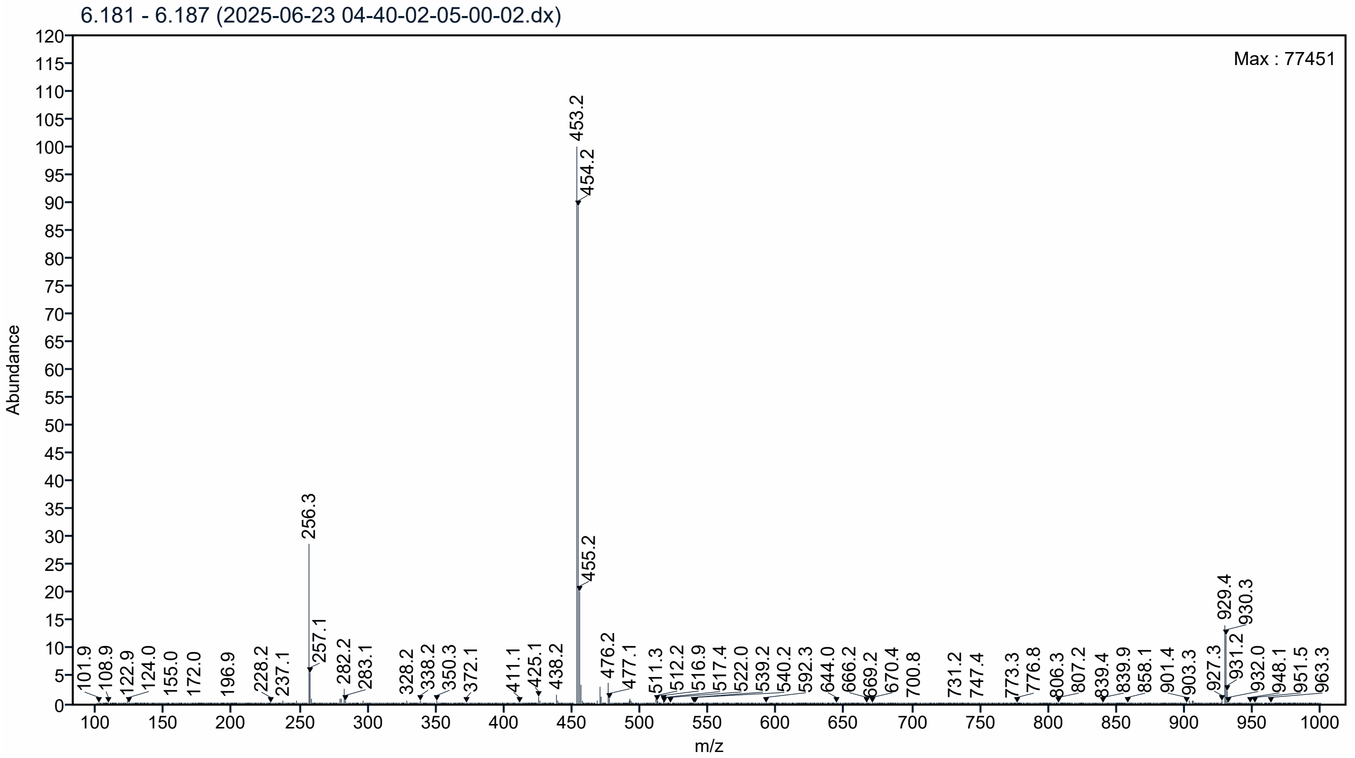

Single Injection Report

**Sample name:**	SR231215A		
**Description:**			
**Sample amount:**	0.000	**Sample type:**	Sample
**Instrument:**	LCMS	**Location:**	P1-A4
		**Injection:**	1 of 1
**Acq. method:**	Regular method.amx	**Injection volume:**	5.000 µL
**Analysis method:**	MS method-purity.pmx	**Acq. operator:**	SYSTEM

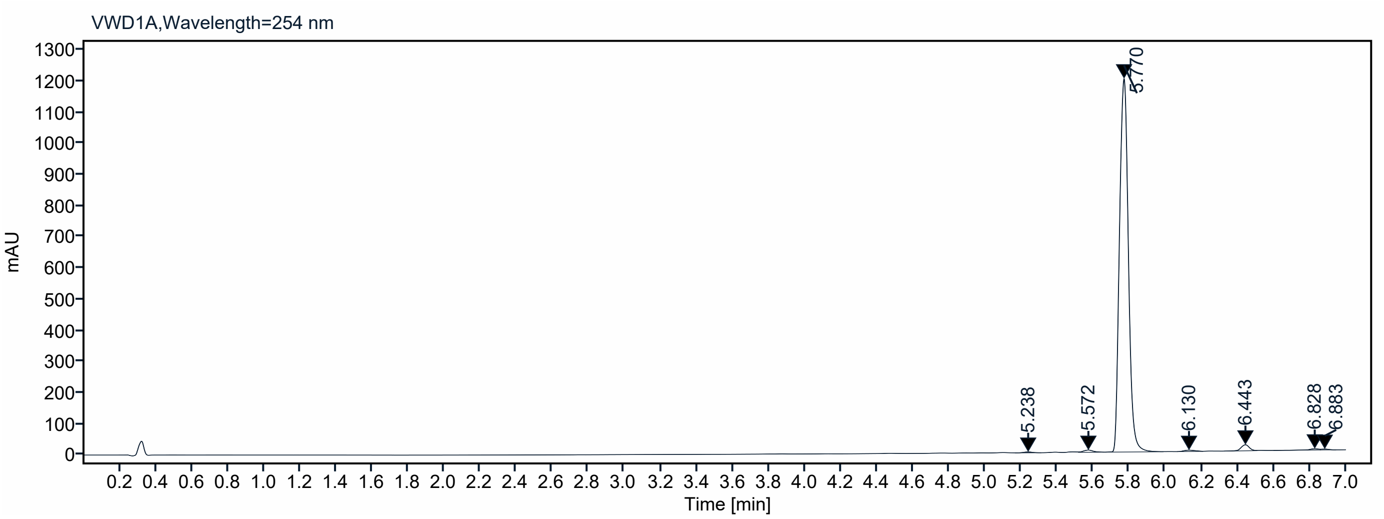

Signal: MS1 +TIC SCAN ESI Frag=110V Gain=1.0

RT [min]	Width [min]	Area	Height	Area%
5.818	0.4213	957576.8680	111815.2830	100.0000
	**Sum**	**957576.8680**		

Signal: VWD1A,Wavelength=254 nm

RT [min]	Width [min]	Area	Height	Area%
5.238	0.1448	9.3164	2.6827	0.2192
5.572	0.1467	26.5232	6.9981	0.6241
5.770	0.3230	4110.8963	1199.8255	96.7254
6.130	0.1441	18.8290	4.3104	0.4430
6.443	0.1832	61.6224	19.3711	1.4499
6.828	0.2601	14.8533	4.0909	0.3495
6.883	0.0878	8.0302	2.7463	0.1889
	**Sum**	**4250.0708**		

5.818 - 5.811 (2025-06-23 04-50-49-05-00-03.dx)

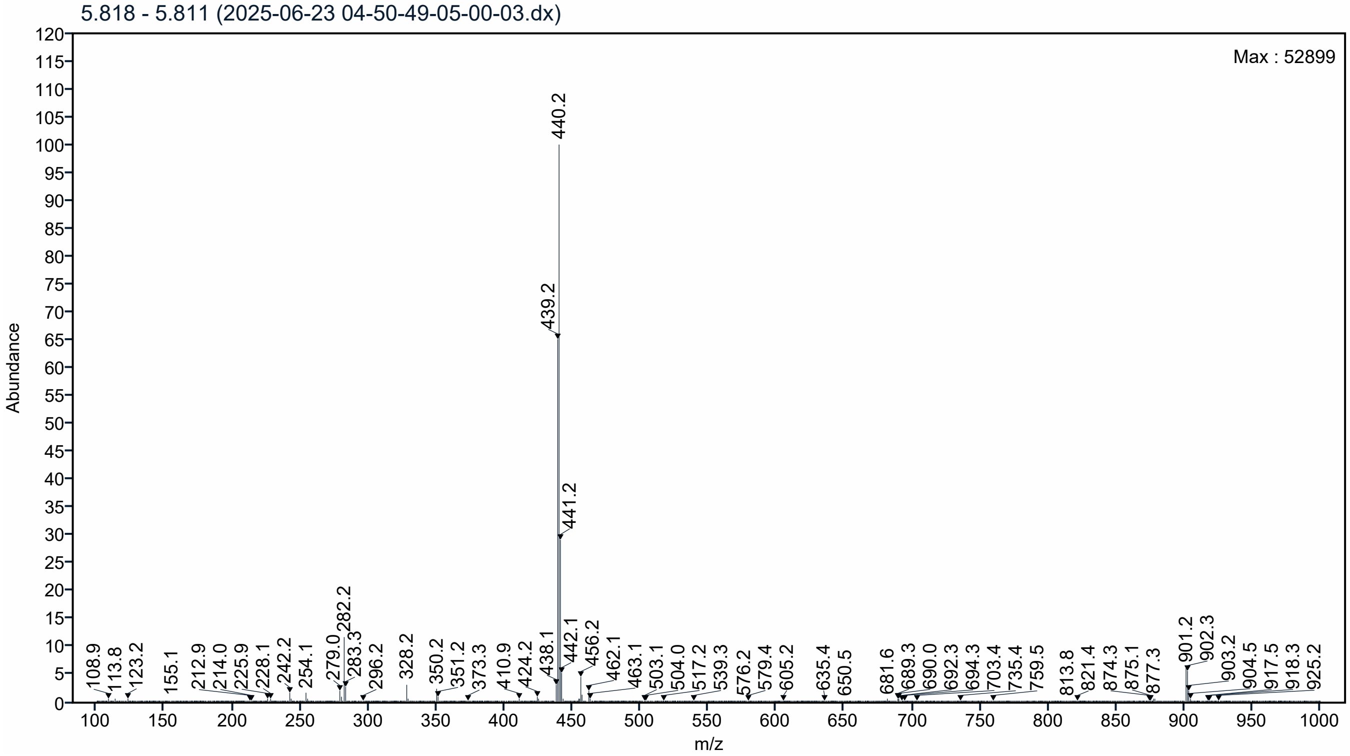

Single Injection Report

**Sample name:**	SR231215B		
**Description:**			
**Sample amount:**	0.000	**Sample type:**	Sample
**Instrument:**	LCMS	**Location:**	P1-A5
		**Injection:**	1 of 1
**Acq. method:**	Regular method.amx	**Injection volume:**	5.000 µL
**Analysis method:**	MS method-purity.pmx	**Acq. operator:**	SYSTEM

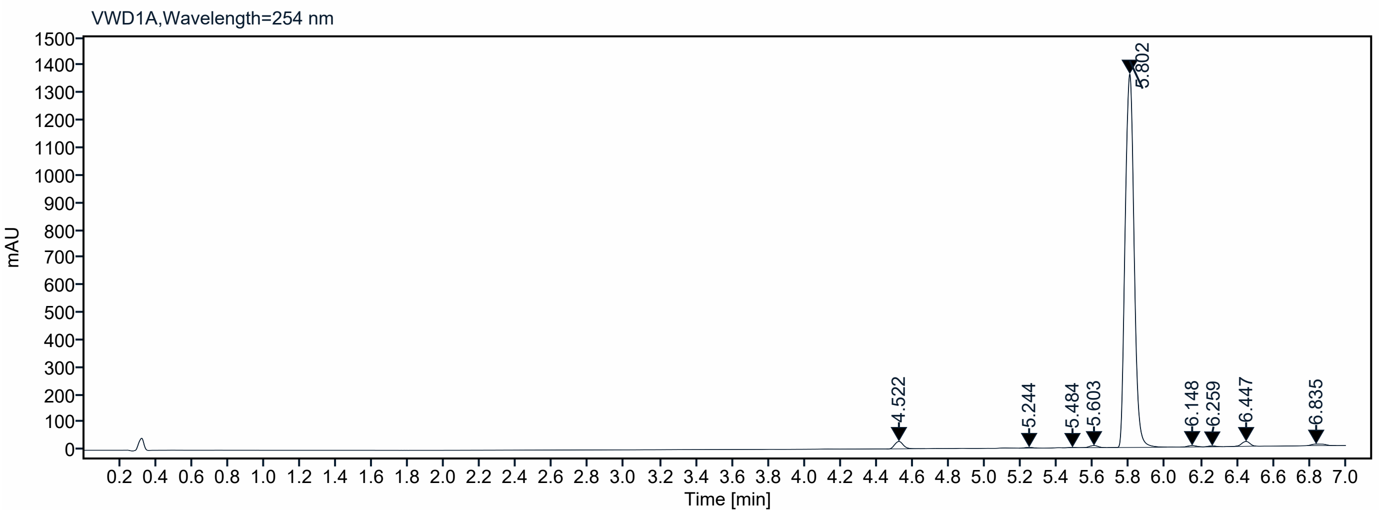

Signal: MS1 +TIC SCAN ESI Frag=110V Gain=1.0

RT [min]	Width [min]	Area	Height	Area%
5.860	0.6818	1331747.4245	160748.8390	100.0000
	**Sum**	**1331747.4245**		

Signal: VWD1A,Wavelength=254 nm

RT [min]	Width [min]	Area	Height	Area%
4.522	0.2138	89.1542	27.1132	1.8083
5.244	0.0989	8.4071	2.7348	0.1705
5.484	0.0956	2.8287	1.1073	0.0574
5.603	0.1498	25.9361	7.6513	0.5261
5.802	0.3300	4689.2603	1360.4227	95.1118
6.148	0.1256	18.3879	5.3667	0.3730
6.259	0.1106	10.7943	3.3600	0.2189
6.447	0.1776	56.7356	17.5894	1.1508
6.835	0.1603	28.7571	5.4181	0.5833
	**Sum**	**4930.2612**		

Single Injection Report

5.86 - 5.863 (2025-06-23 05-01-34-05-00-04.dx)

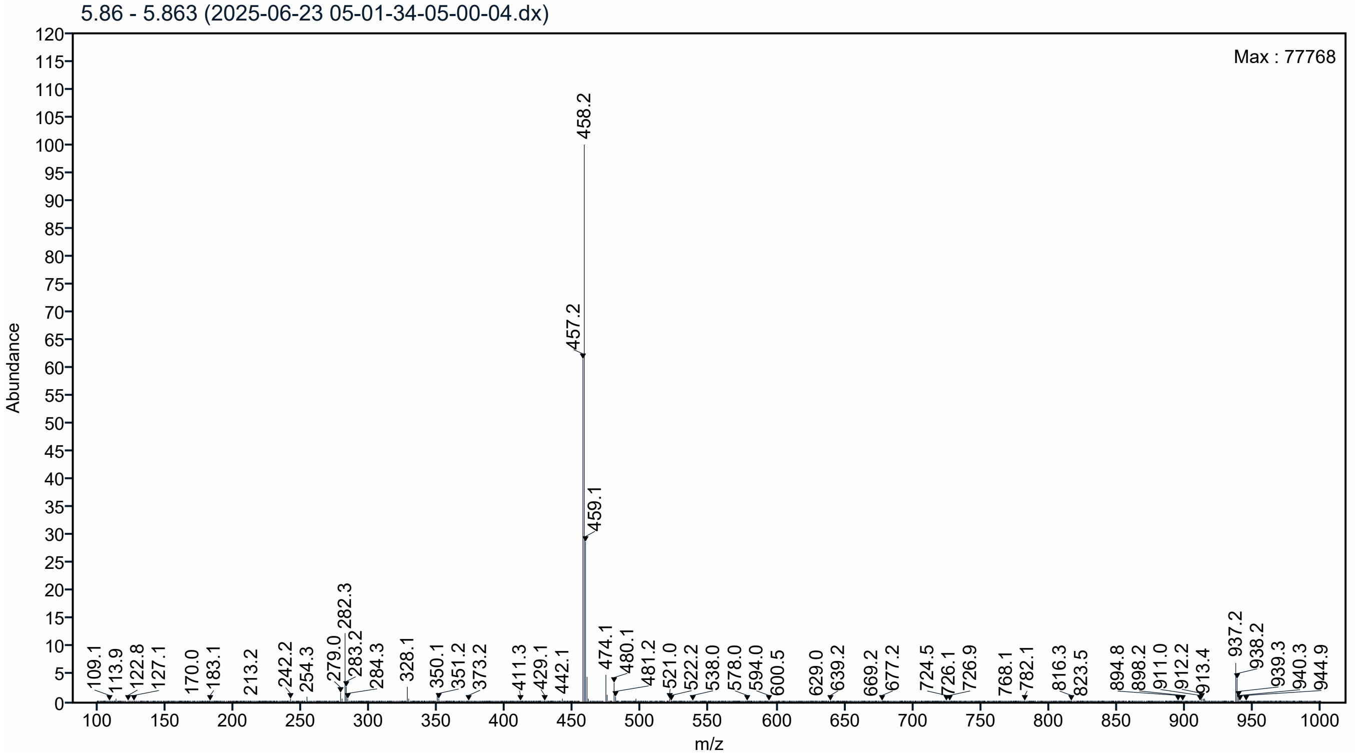

Single Injection Report

**Sample name:**	SR230329B		
**Description:**			
**Sample amount:**	0.000	**Sample type:**	Sample
**Instrument:**	LCMS	**Location:**	P1-A6
		**Injection:**	1 of 1
**Acq. method:**	Regular method.amx	**Injection volume:**	5.000 µL
**Analysis method:**	MS method-purity.pmx	**Acq. operator:**	SYSTEM

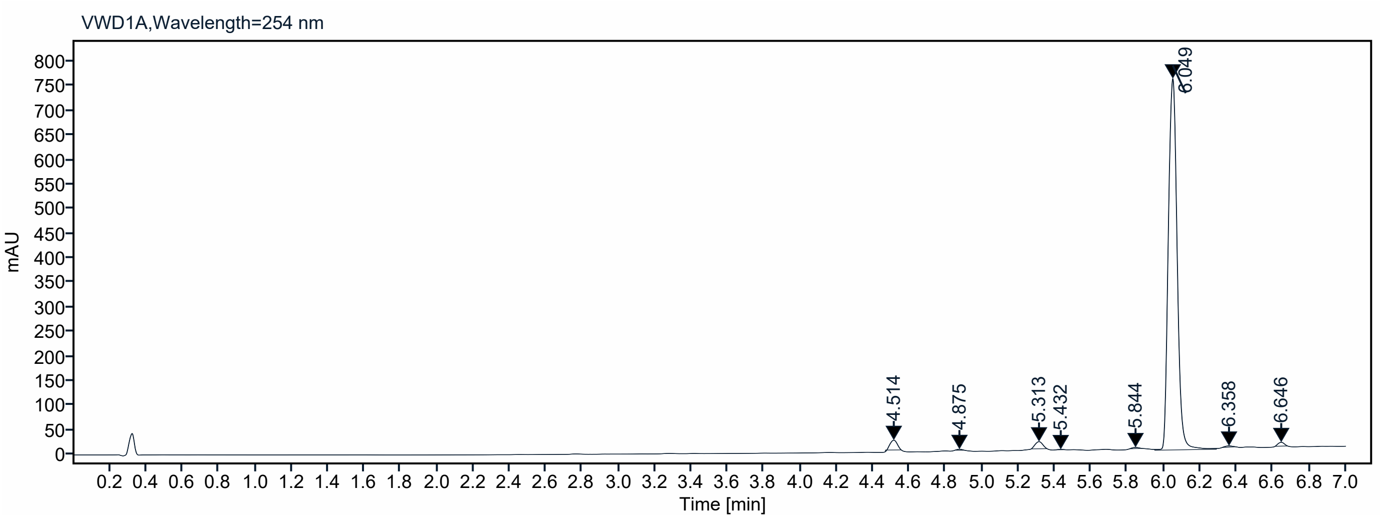

Signal: MS1 +TIC SCAN ESI Frag=110V Gain=1.0

RT [min]	Width [min]	Area	Height	Area%
6.107	0.1411	537386.9039	112381.6472	100.0000
	**Sum**	**537386.9039**		

Signal: VWD1A,Wavelength=254 nm

RT [min]	Width [min]	Area	Height	Area%
4.514	0.0869	53.1955	19.9182	1.9892
4.875	0.0514	3.7084	1.9606	0.1387
5.313	0.0869	41.5131	14.5104	1.5523
5.432	0.0514	1.4329	0.7356	0.0536
5.844	0.0672	6.0283	2.4721	0.2254
6.049	0.3398	2543.0081	755.2638	95.0923
6.358	0.0751	7.0653	2.5708	0.2642
6.646	0.0682	18.3018	7.8223	0.6844
	**Sum**	**2674.2535**		

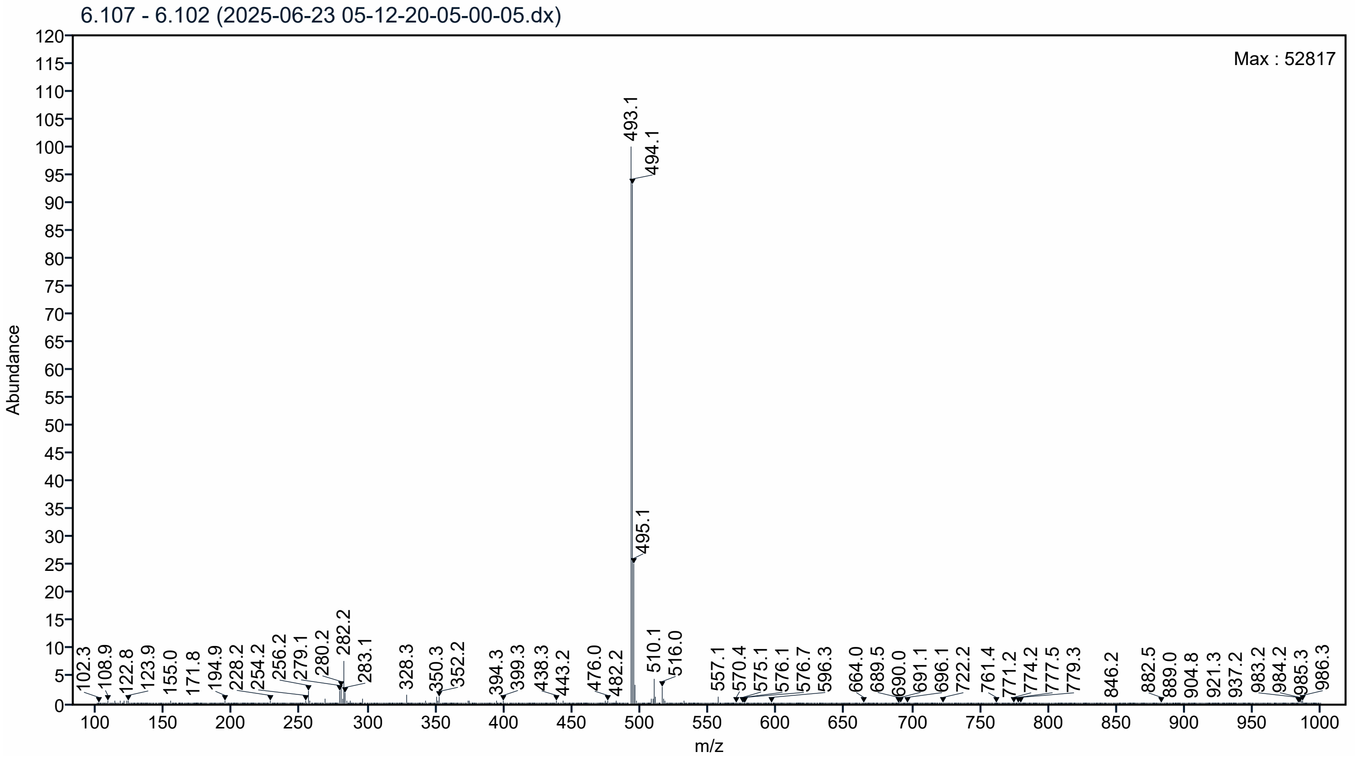
6.107 - 6.102 (2025-06-23 05-12-20-05-00-05.dx)
Max : 52817
Abundance
m/z
493.1
494.1
495.1
510.1
516.0
282.2
283.1
328.3
350.3
352.2

Mass Analysis Report

SAMPLE INFORMATION

Sample Name: SR200907
Acq Method Set: Col2_MeCN_H2O_NH4HCO3
Acquired: 1/29/2021 10:58:42 PM CST
InjVol: 7.50 uL

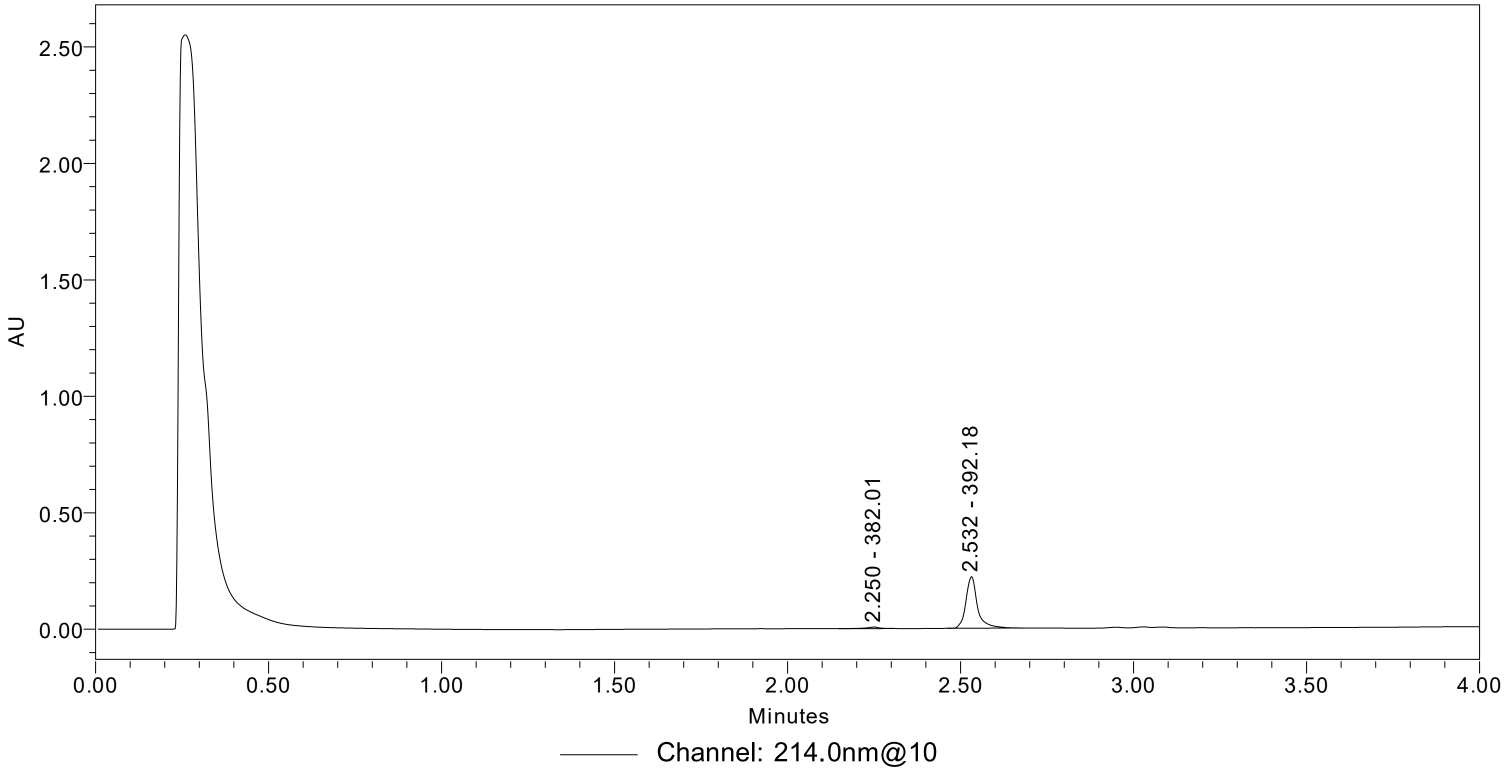

	RT	Area	% Area	Height	Base Peak (m/z)
1	2.250	16327	3.07	6314	382.01
2	2.532	515568	96.93	220650	392.18

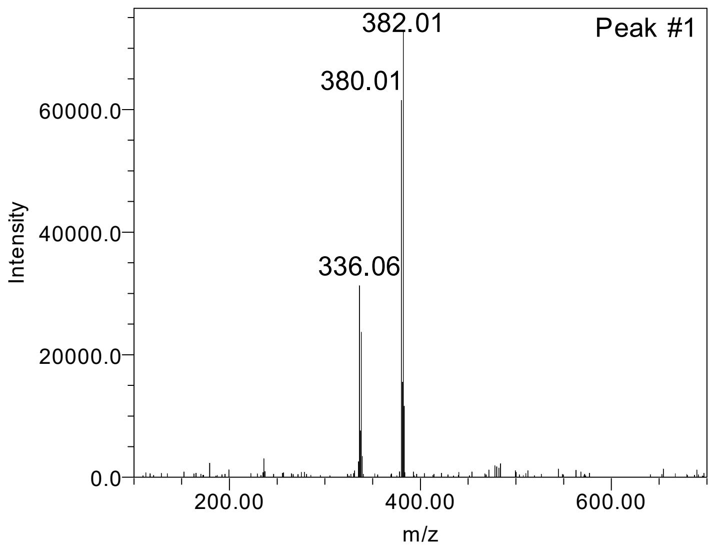

RT: 2.250

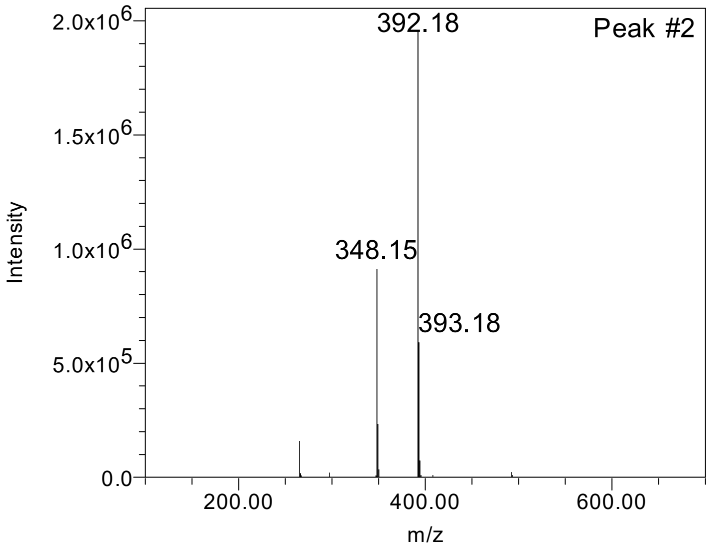

RT: 2.532

Mass Analysis Report

SAMPLE INFORMATION

Sample Name: SR200907
Acq Method Set: Col1_MeOH_H2O_NH4HCO3
Acquired: 1/30/2021 8:48:45 AM CST
InjVol: 7.50 uL

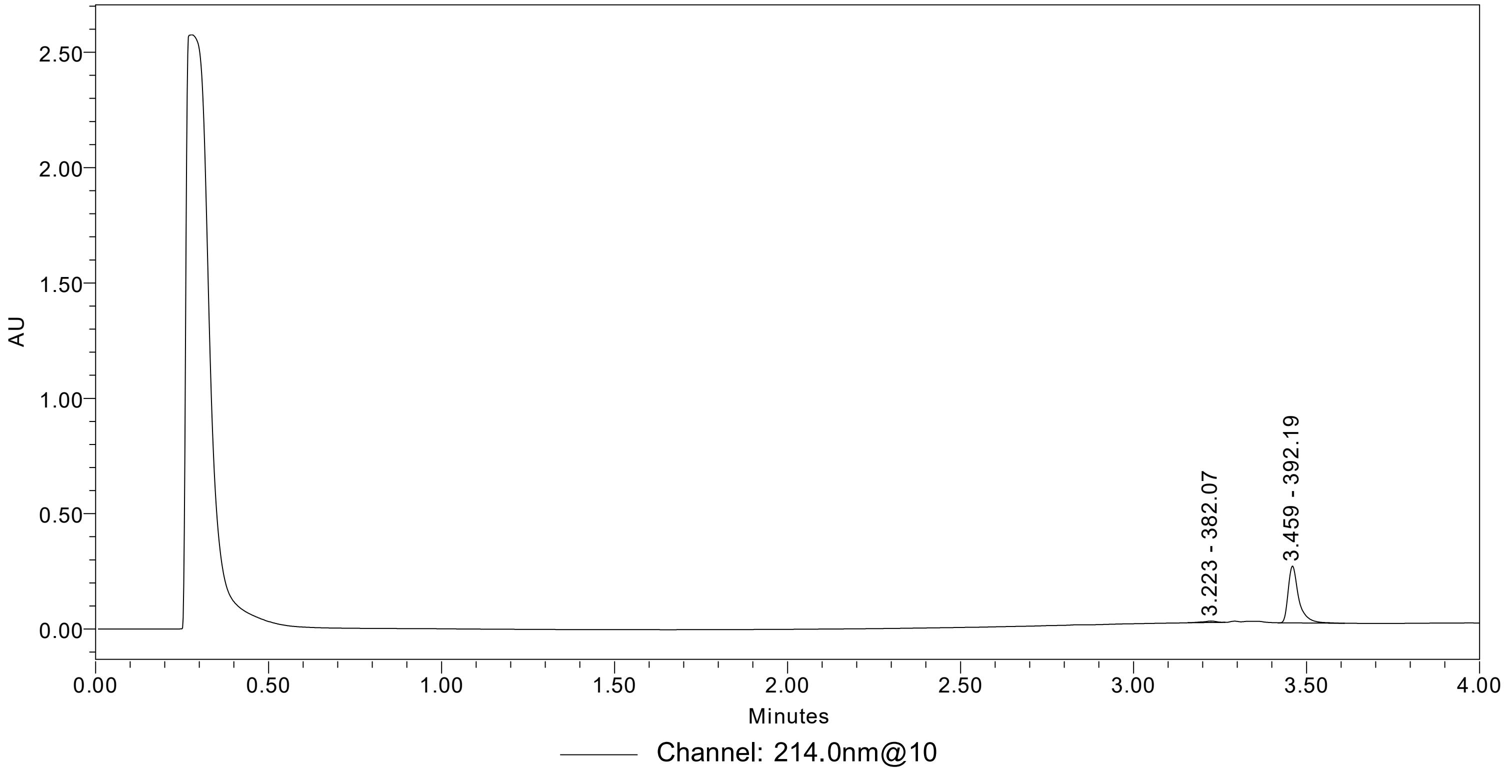

	RT	Area	% Area	Height	Base Peak (m/z)
1	3.223	17157	3.18	6302	382.07
2	3.459	522014	96.82	246060	392.19

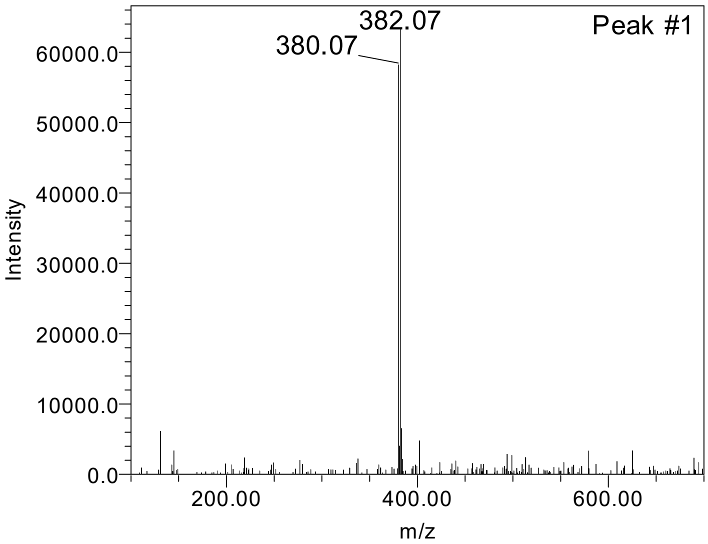

RT: 3.223

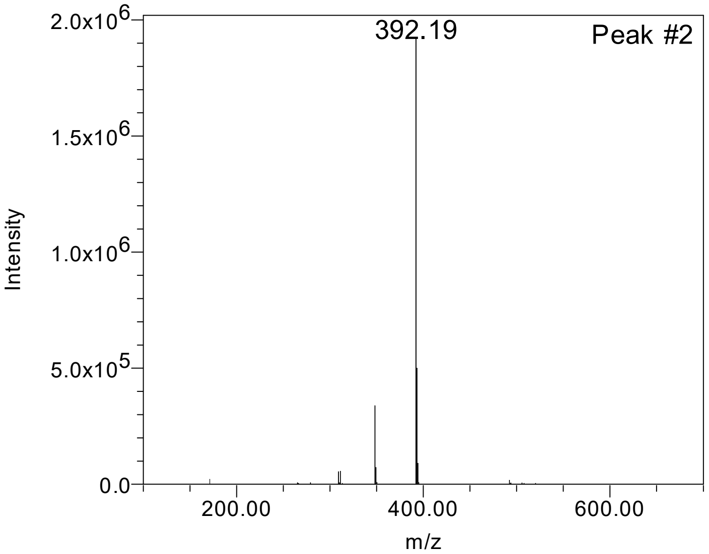

RT: 3.459

Mass Analysis Report

SAMPLE INFORMATION

Sample Name: SR201119A
Acq Method Set: Col2_MeCN_H2O_NH4HCO3
Acquired: 1/29/2021 11:05:49 PM CST
InjVol: 7.50 uL

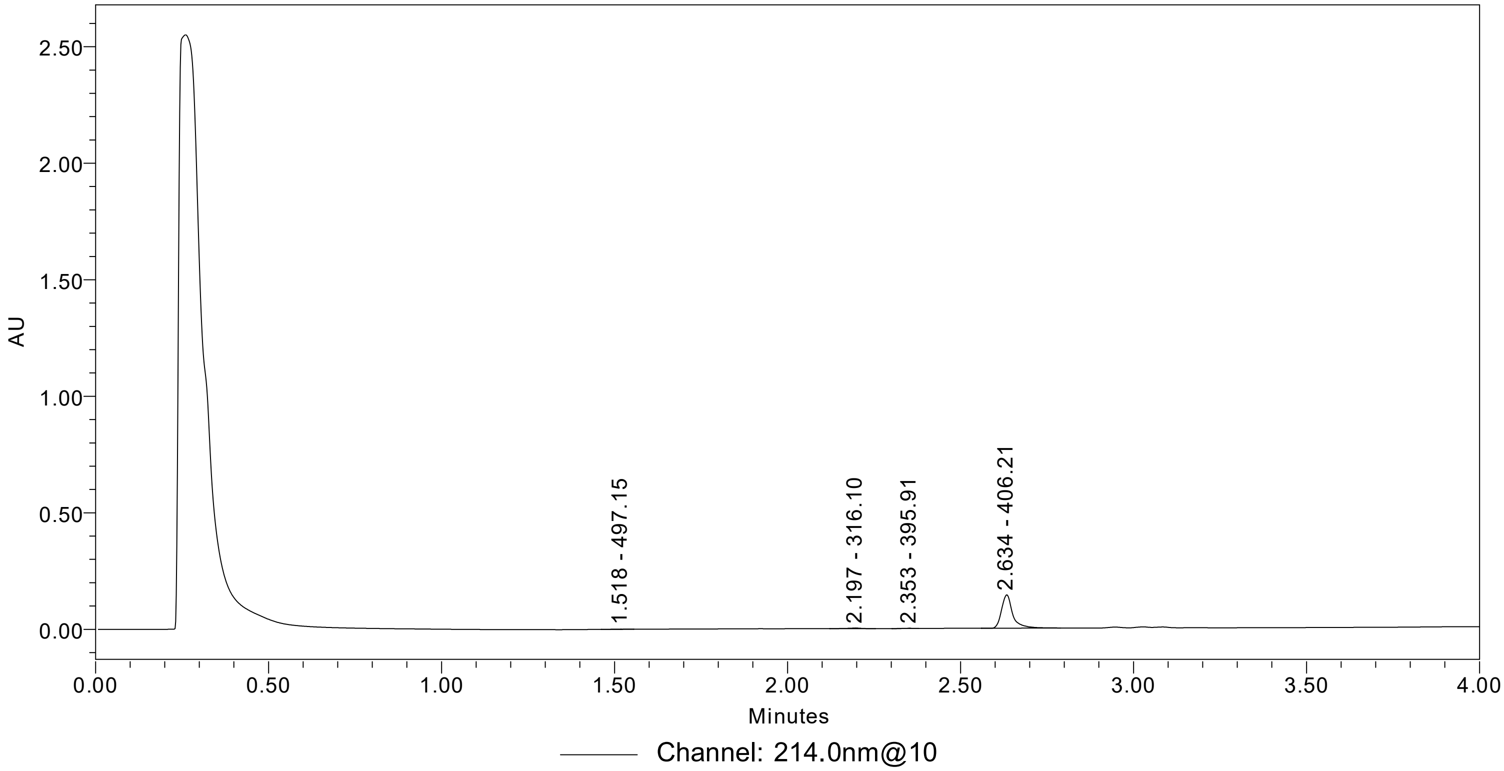

	RT	Area	% Area	Height	Base Peak (m/z)
1	1.518	2030	0.61	848	497.15
2	2.197	6310	1.89	2321	316.10
3	2.353	1623	0.49	803	395.91
4	2.634	323382	97.01	142830	406.21

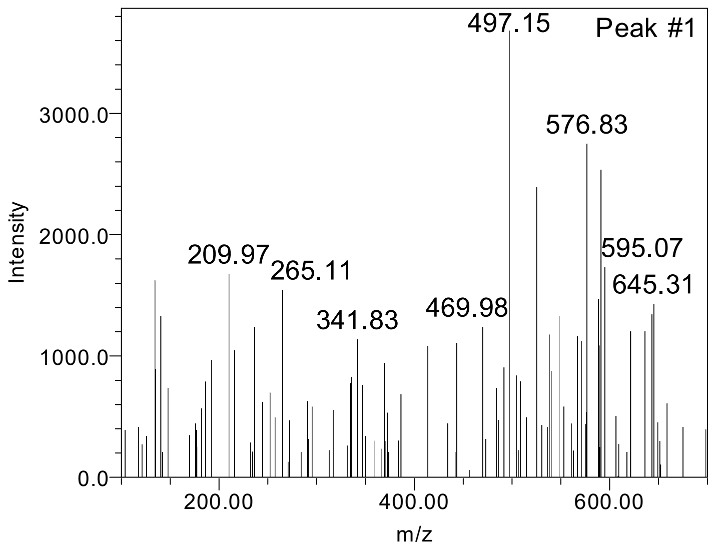

RT: 1.518

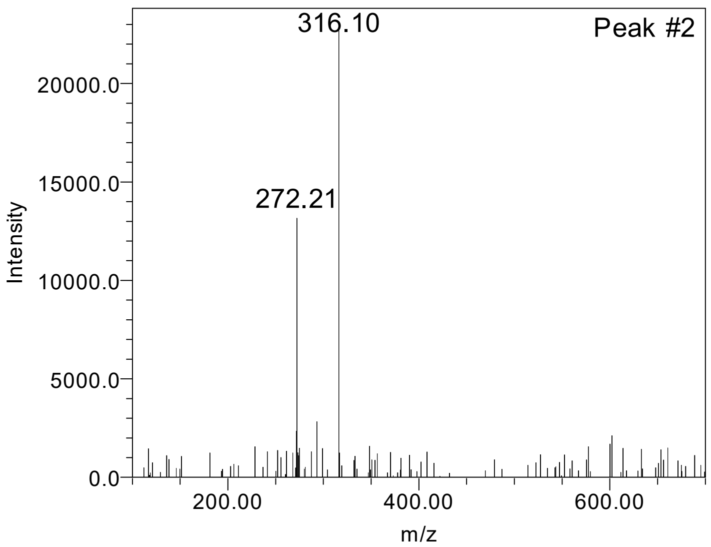

RT: 2.197

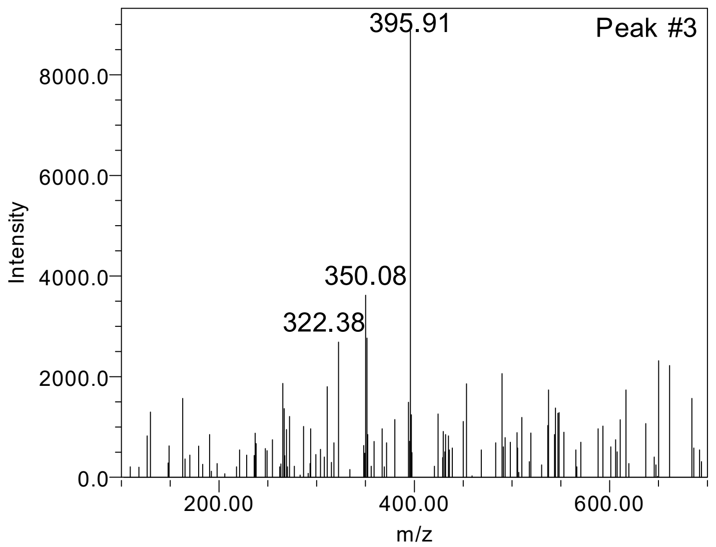

RT: 2.353

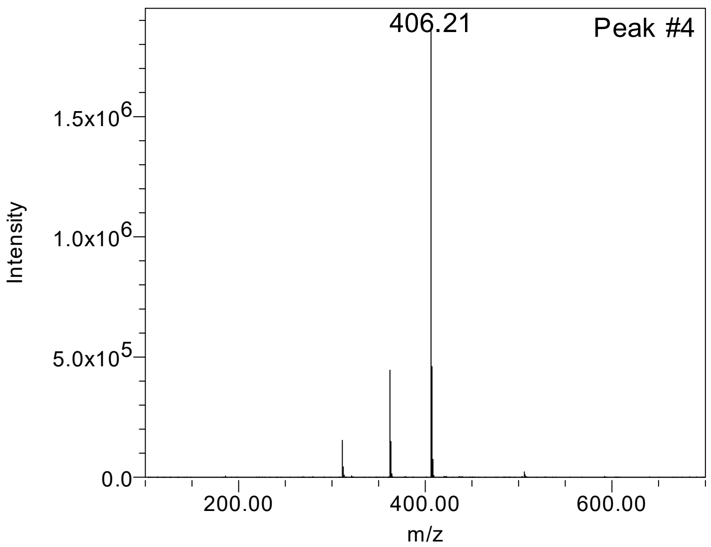

RT: 2.634

Mass Analysis Report

SAMPLE INFORMATION

Sample Name: SR201119A
Acq Method Set: Col1_MeOH_H2O_NH4HCO3
Acquired: 1/30/2021 8:55:51 AM CST
InjVol: 7.50 uL

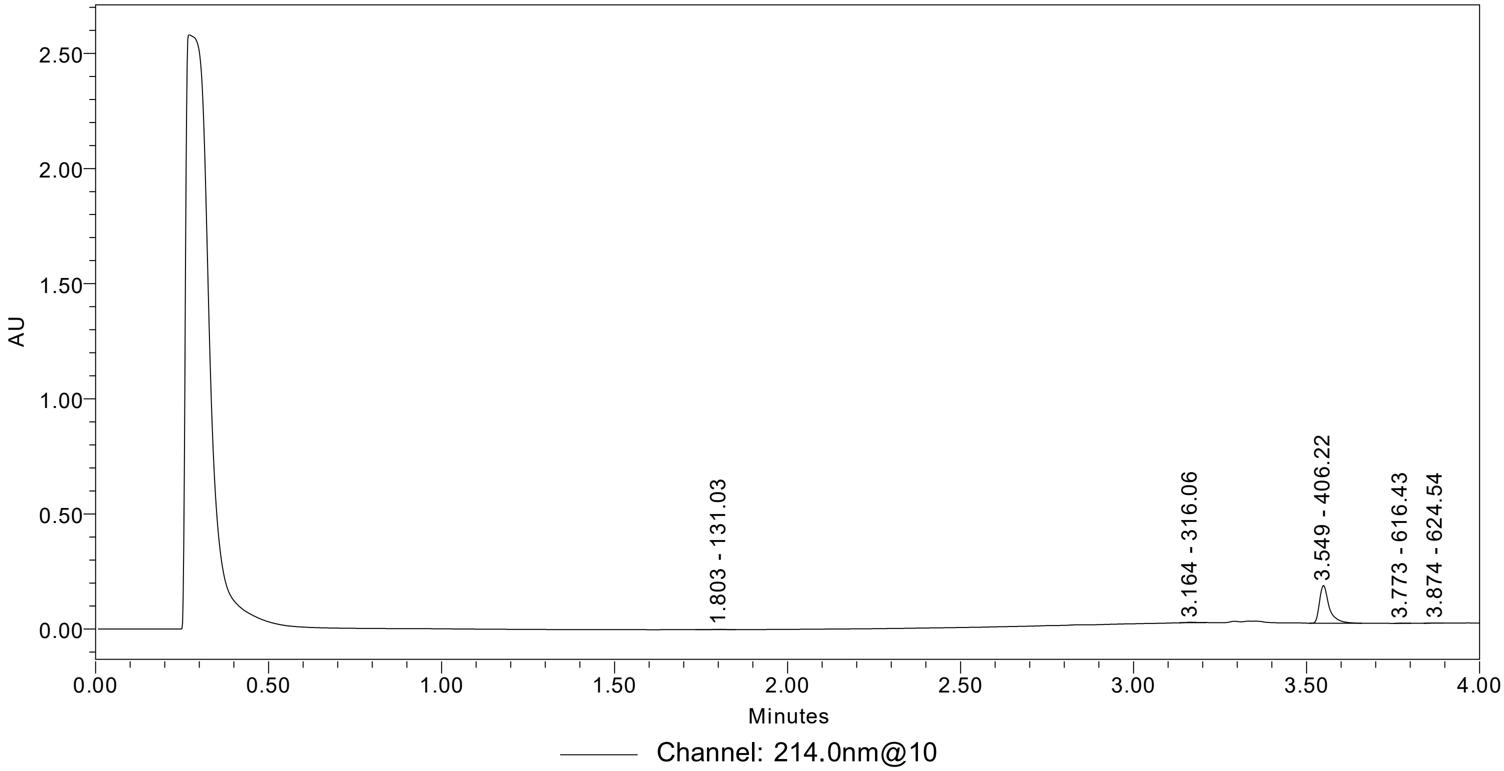

	RT	Area	% Area	Height	Base Peak (m/z)
1	1.803	2528	0.77	987	131.03
2	3.164	4599	1.40	2025	316.06
3	3.549	319208	97.41	163220	406.22
4	3.773	752	0.23	549	616.43
5	3.874	598	0.18	362	624.54

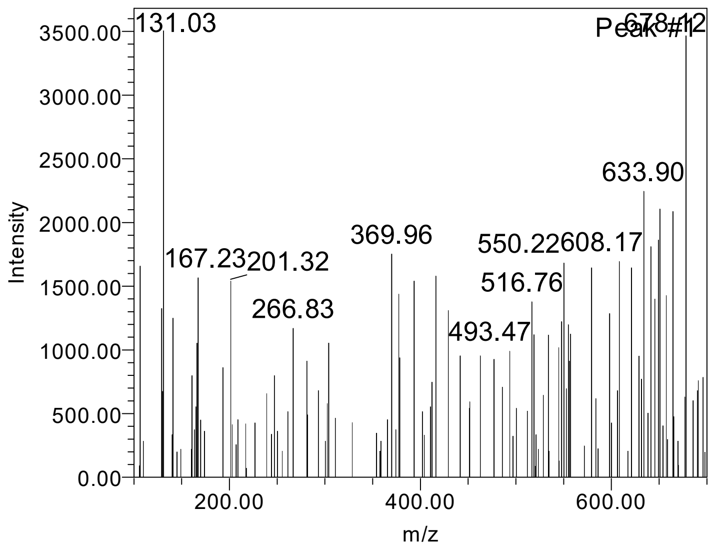

RT: 1.803

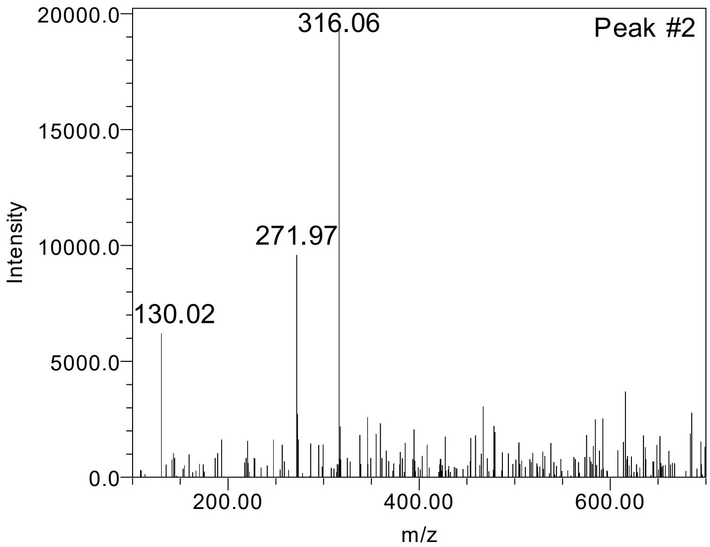

RT: 3.164

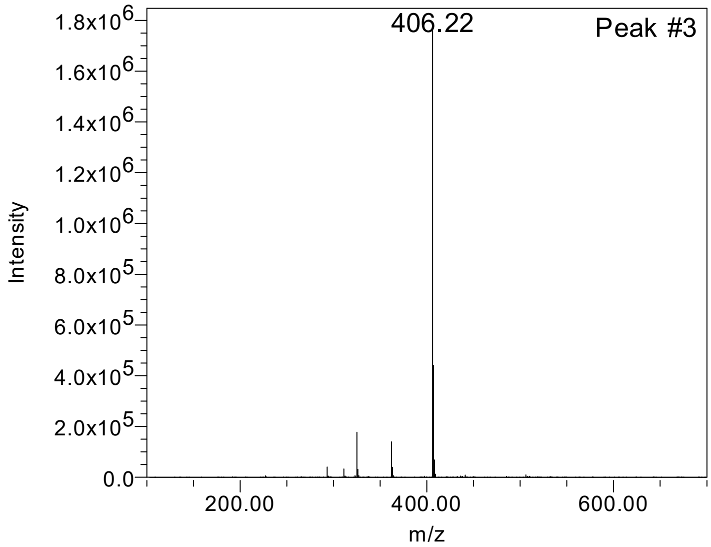

RT: 3.549

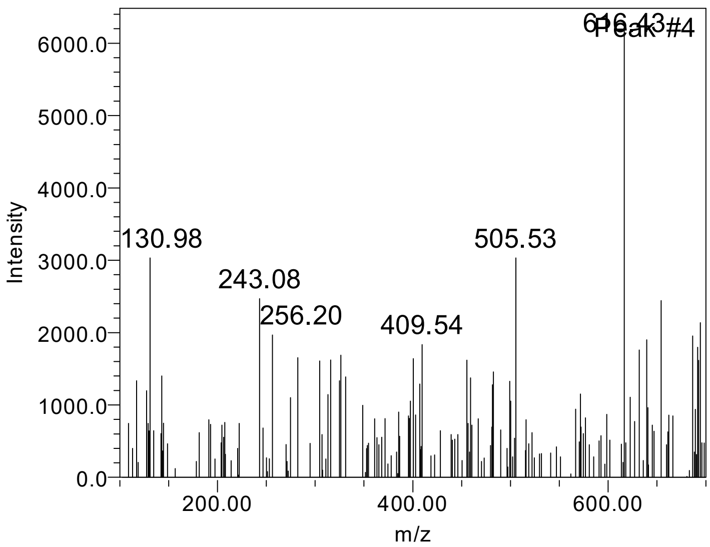

RT: 3.773

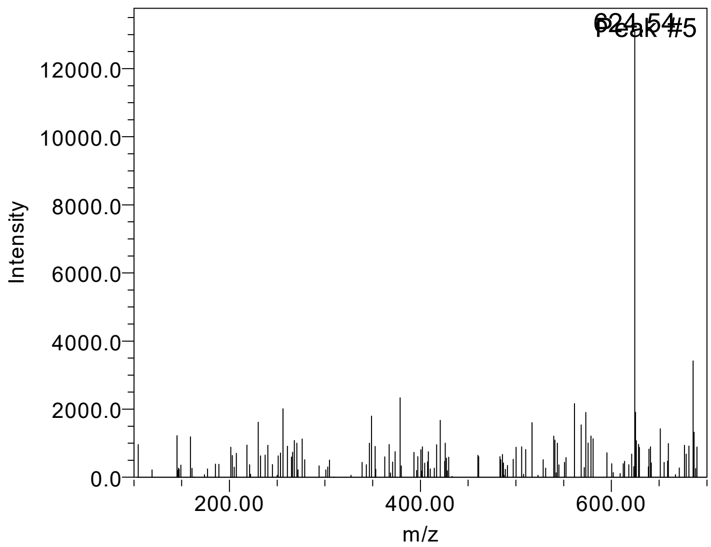

RT: 3.874

Mass Analysis Report

SAMPLE INFORMATION

Sample Name: SR210122A
Acq Method Set: Col2_MeCN_H2O_NH4HCO3
Acquired: 1/29/2021 11:34:11 PM CST
InjVol: 7.50 uL

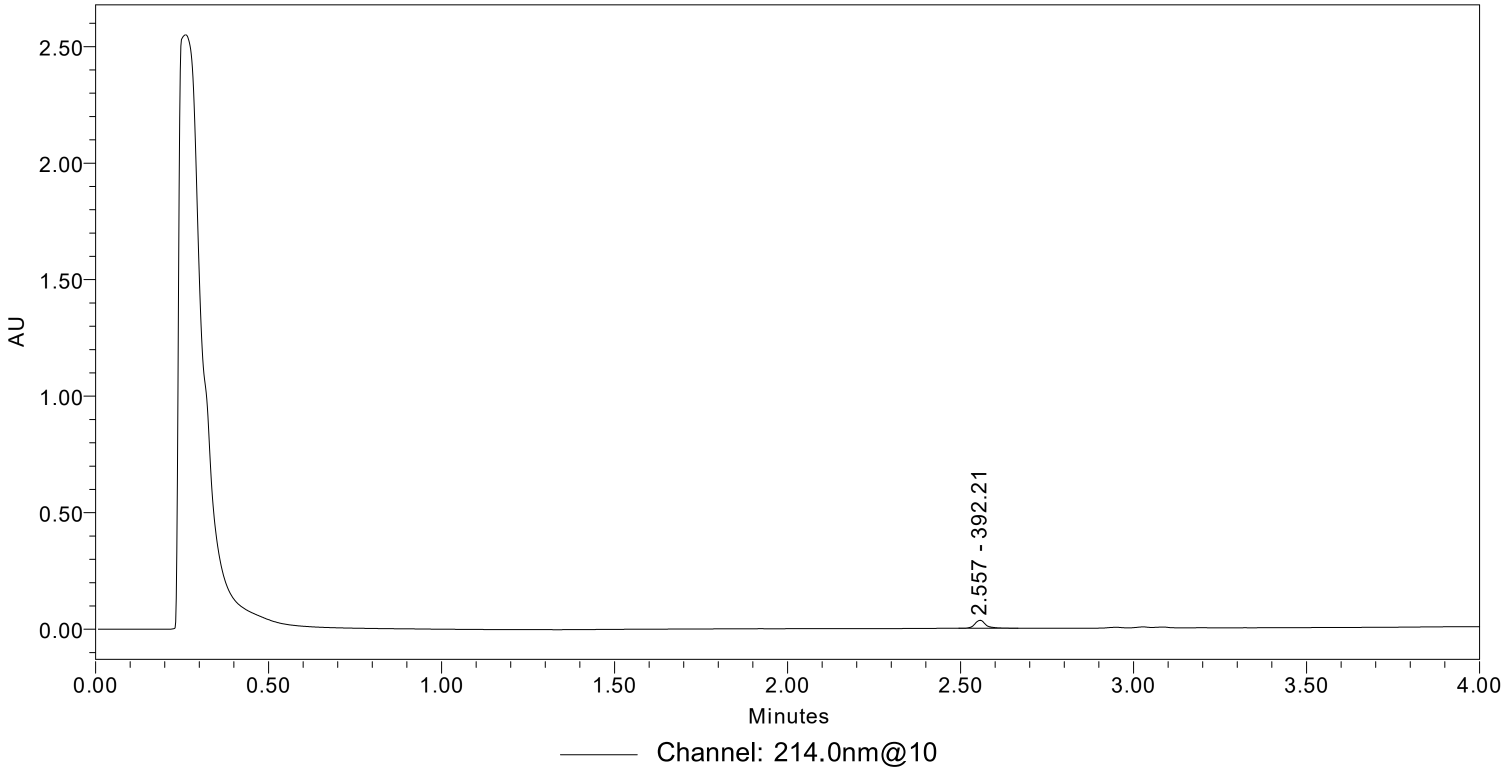

	RT	Area	% Area	Height	Base Peak (m/z)
1	2.557	75467	100.00	34016	392.21

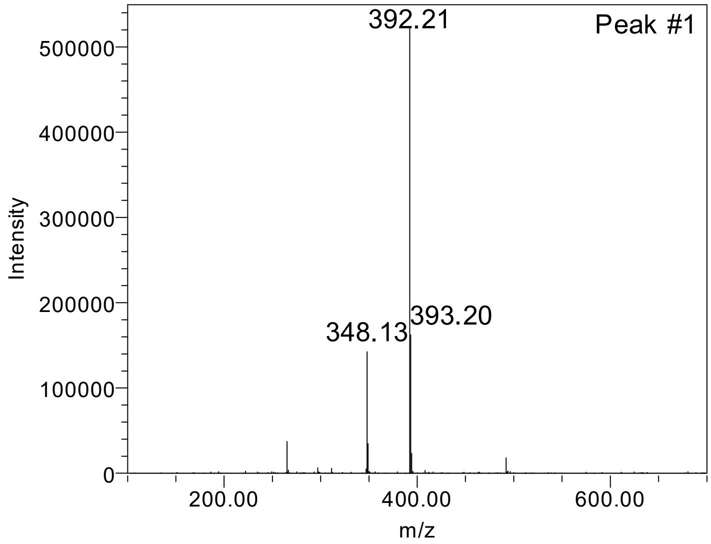

RT: 2.557

Mass Analysis Report

SAMPLE INFORMATION

Sample Name: SR210122A
Acq Method Set: Col1_MeOH_H2O_NH4HCO3
Acquired: 1/30/2021 9:24:15 AM CST
InjVol: 7.50 uL

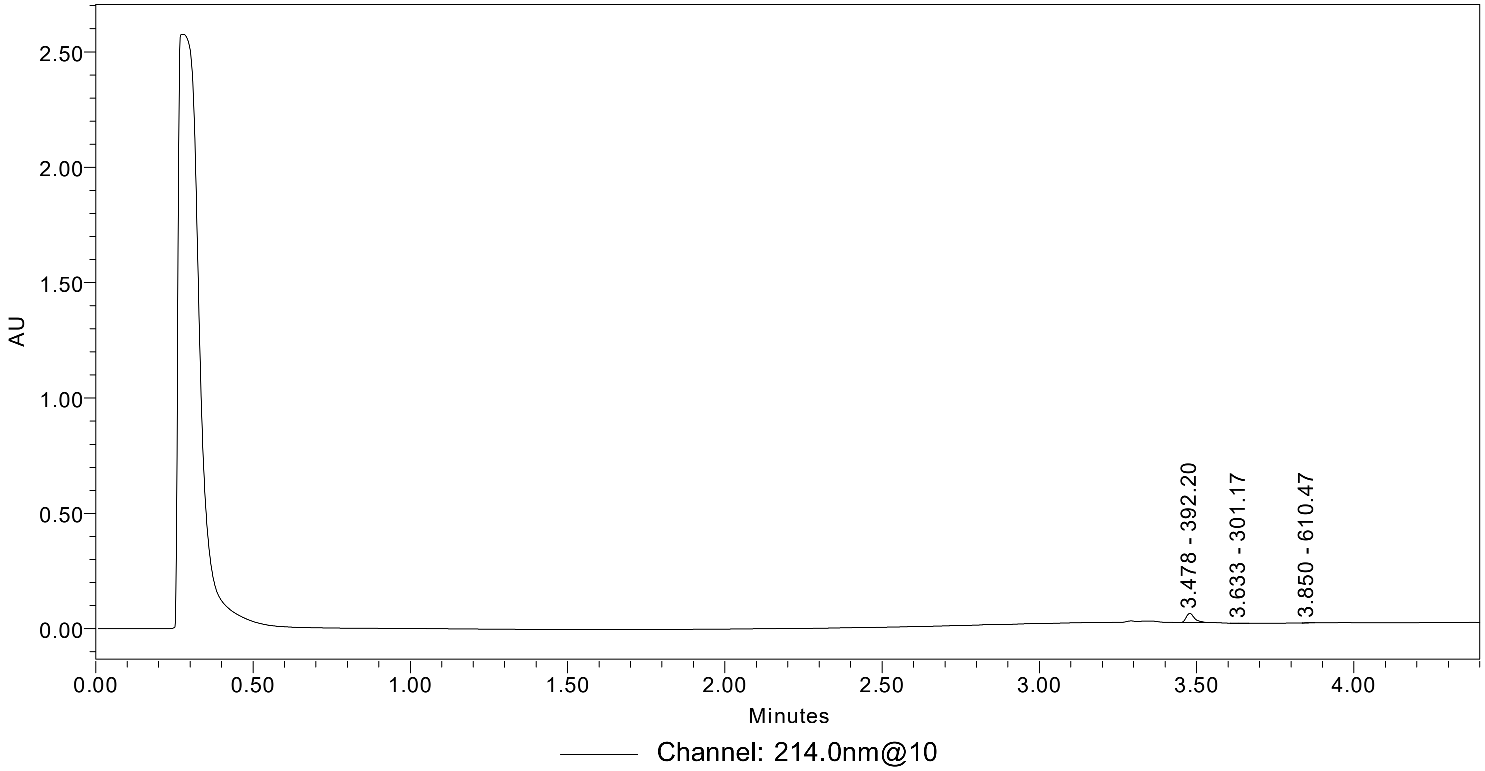

	RT	Area	% Area	Height	Base Peak (m/z)
1	3.478	75778	98.82	39881	392.20
2	3.633	526	0.69	293	301.17
3	3.850	380	0.50	326	610.47

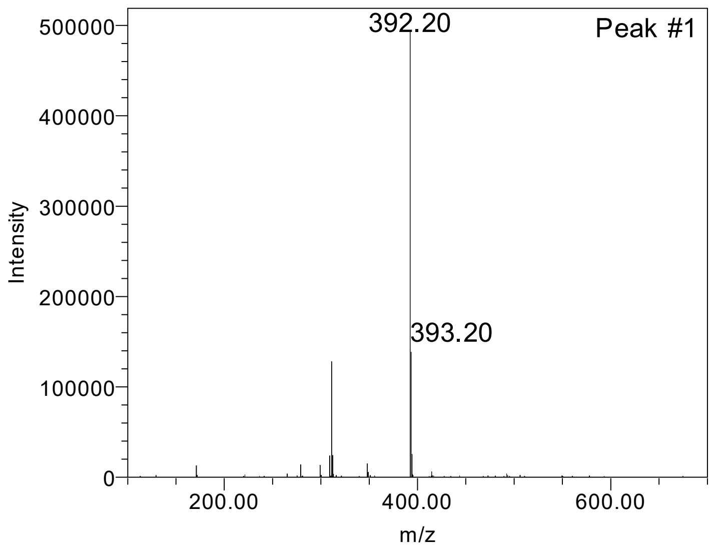

RT: 3.478

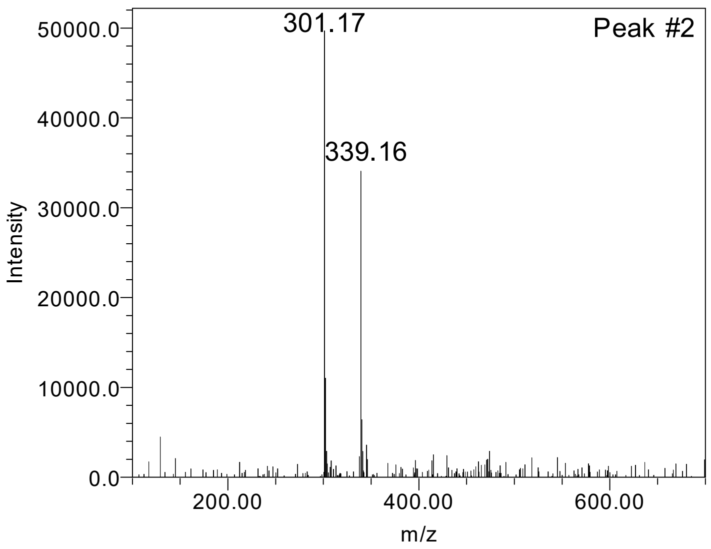

RT: 3.633

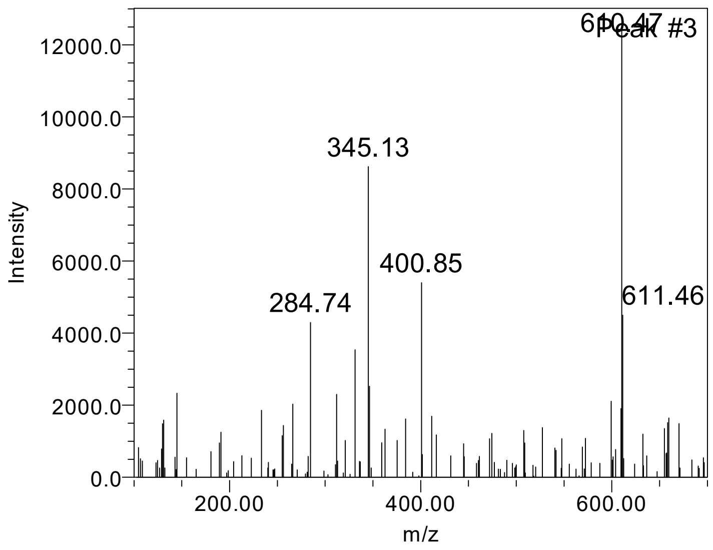

RT: 3.850

Mass Analysis Report

SAMPLE INFORMATION

Sample Name: SR201119B
Acq Method Set: Col2_MeCN_H2O_NH4HCO3
Acquired: 1/29/2021 11:12:54 PM CST
InjVol: 7.50 uL

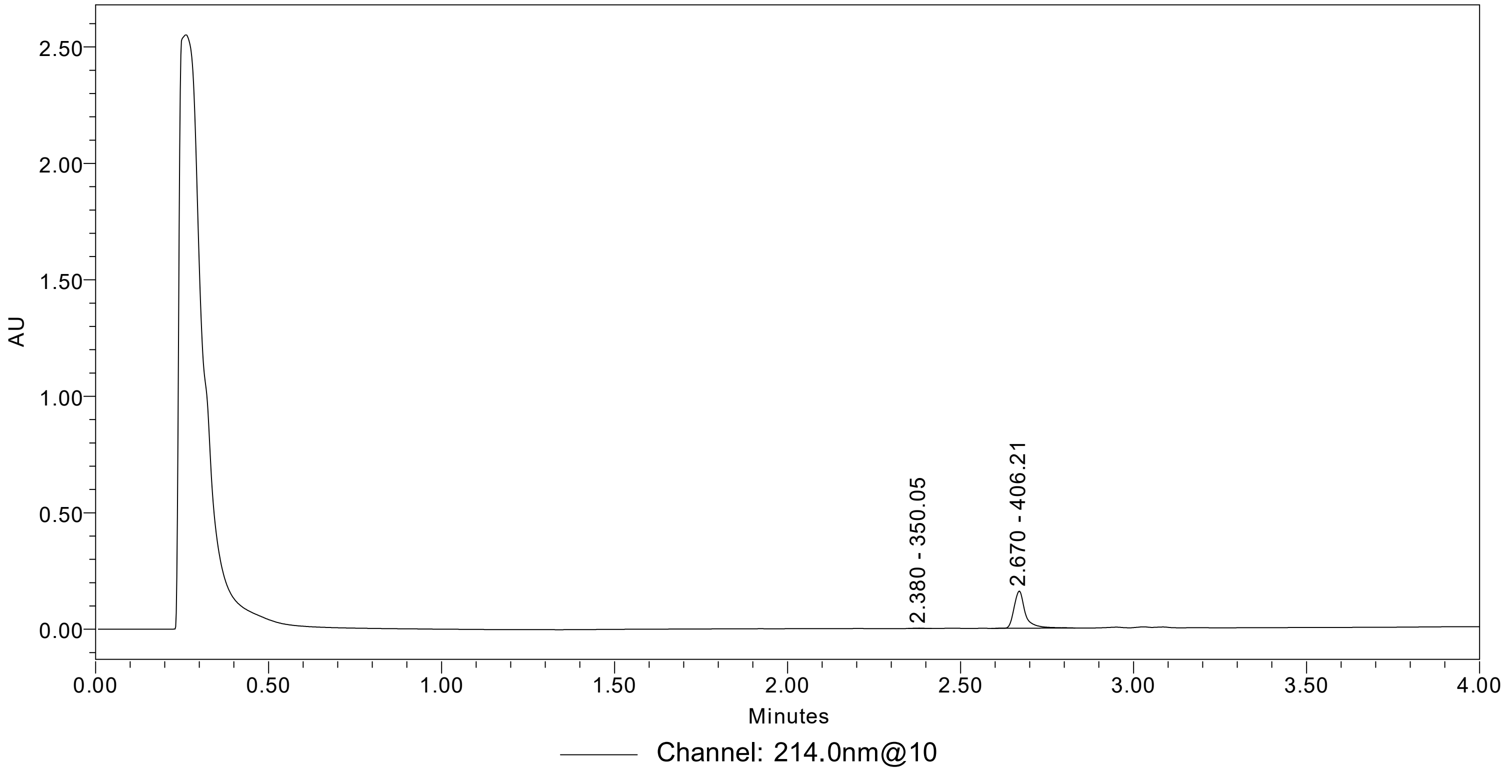

	RT	Area	% Area	Height	Base Peak (m/z)
1	2.380	2061	0.57	1204	350.05
2	2.670	359588	99.43	159229	406.21

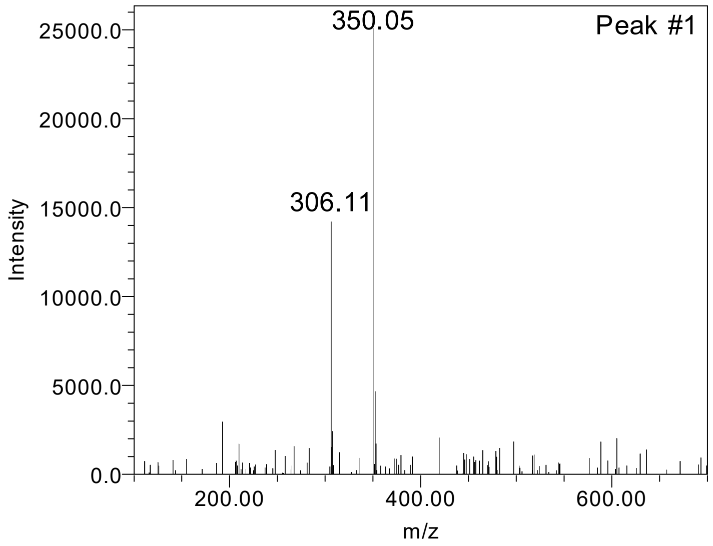

RT: 2.380

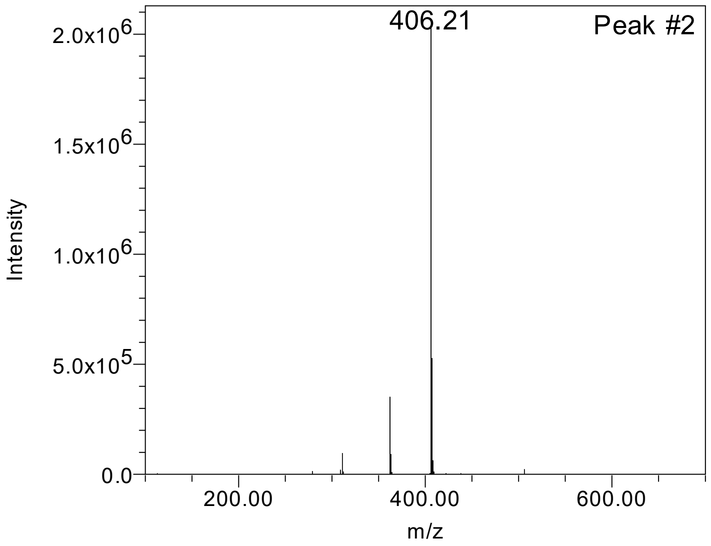

RT: 2.670

Mass Analysis Report

SAMPLE INFORMATION

Sample Name: SR201119B
Acq Method Set: Col1_MeOH_H2O_NH4HCO3
Acquired: 1/30/2021 9:02:57 AM CST
InjVol: 7.50 uL

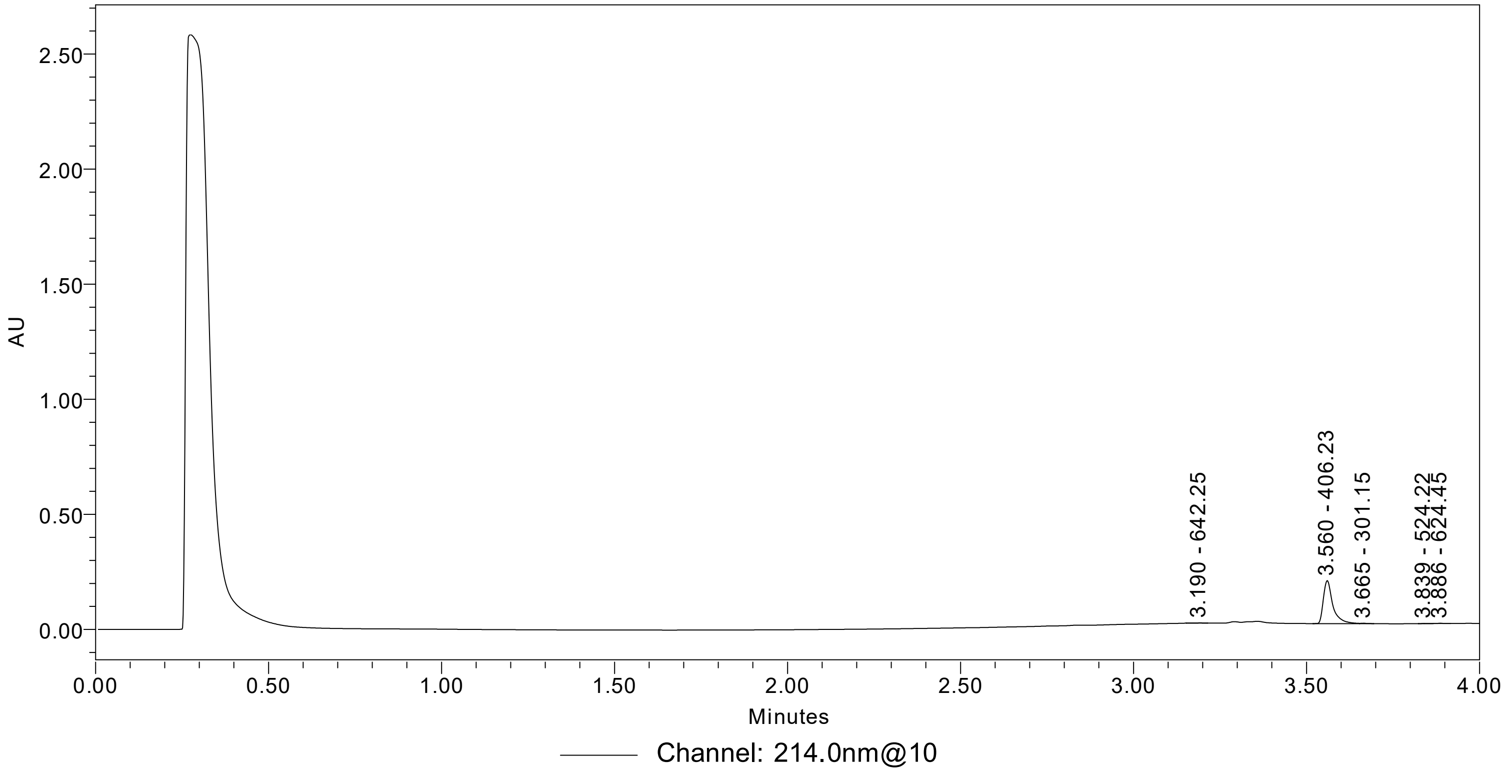

	RT	Area	% Area	Height	Base Peak (m/z)
1	3.190	993	0.28	372	642.25
2	3.560	354608	98.75	186446	406.23
3	3.665	1774	0.49	1156	301.15
4	3.839	719	0.20	500	524.22
5	3.886	993	0.28	776	624.45

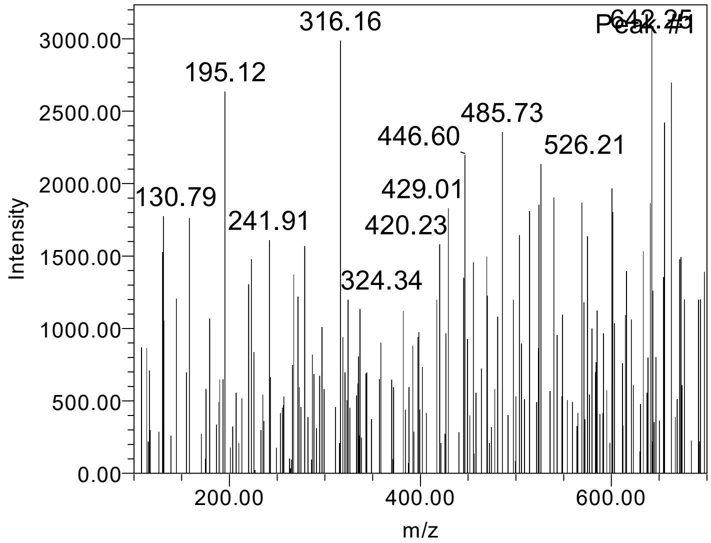

RT: 3.190

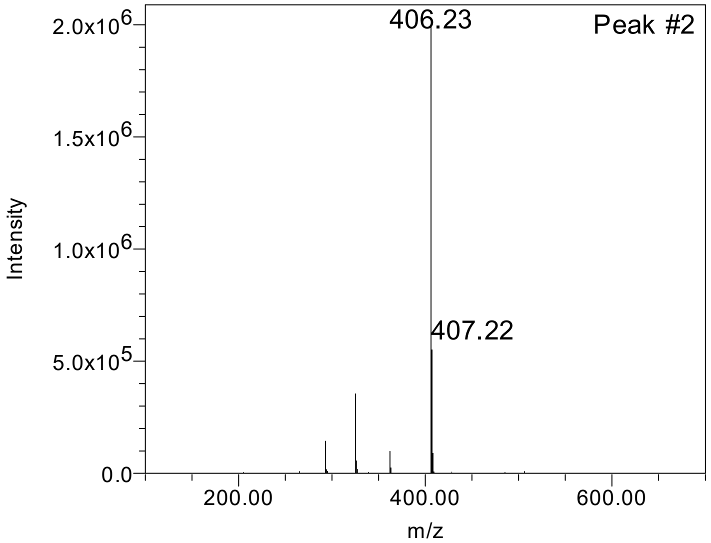

RT: 3.560

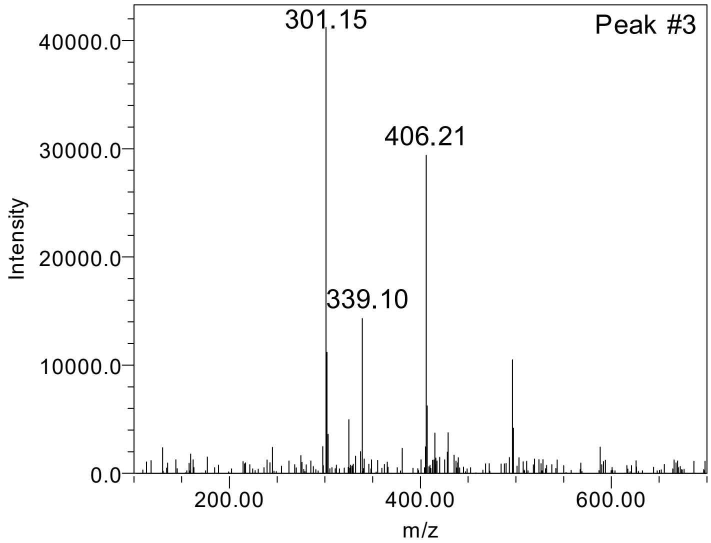

RT: 3.665

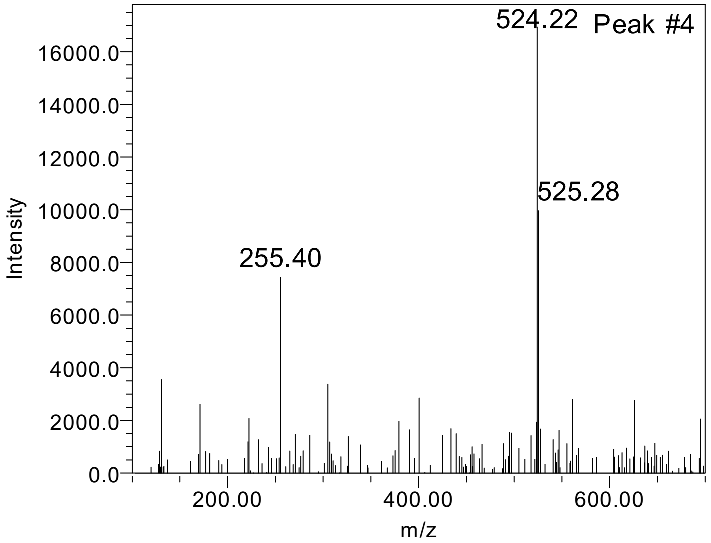

RT: 3.839

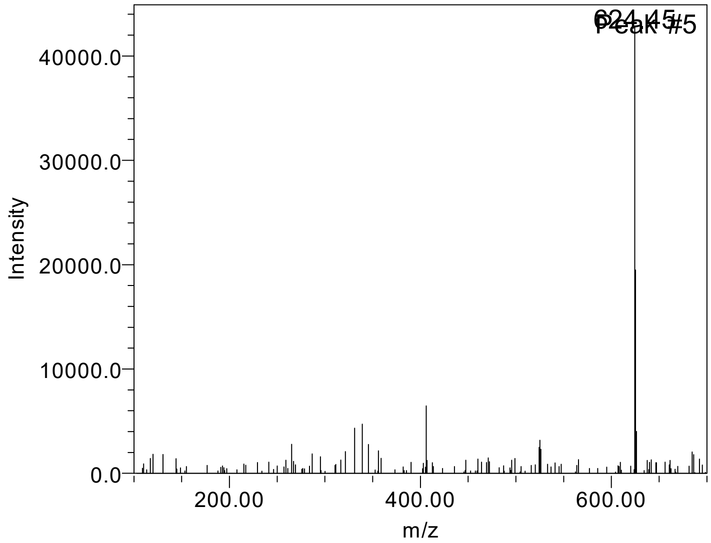

RT: 3.886

Mass Analysis Report

SAMPLE INFORMATION

Sample Name: SR211009A
Acq Method Set: Col2_MeCN_H2O_NH4HCO3
Acquired: 11/11/2021 12:45:45 PM CST
InjVol: 3.00 uL

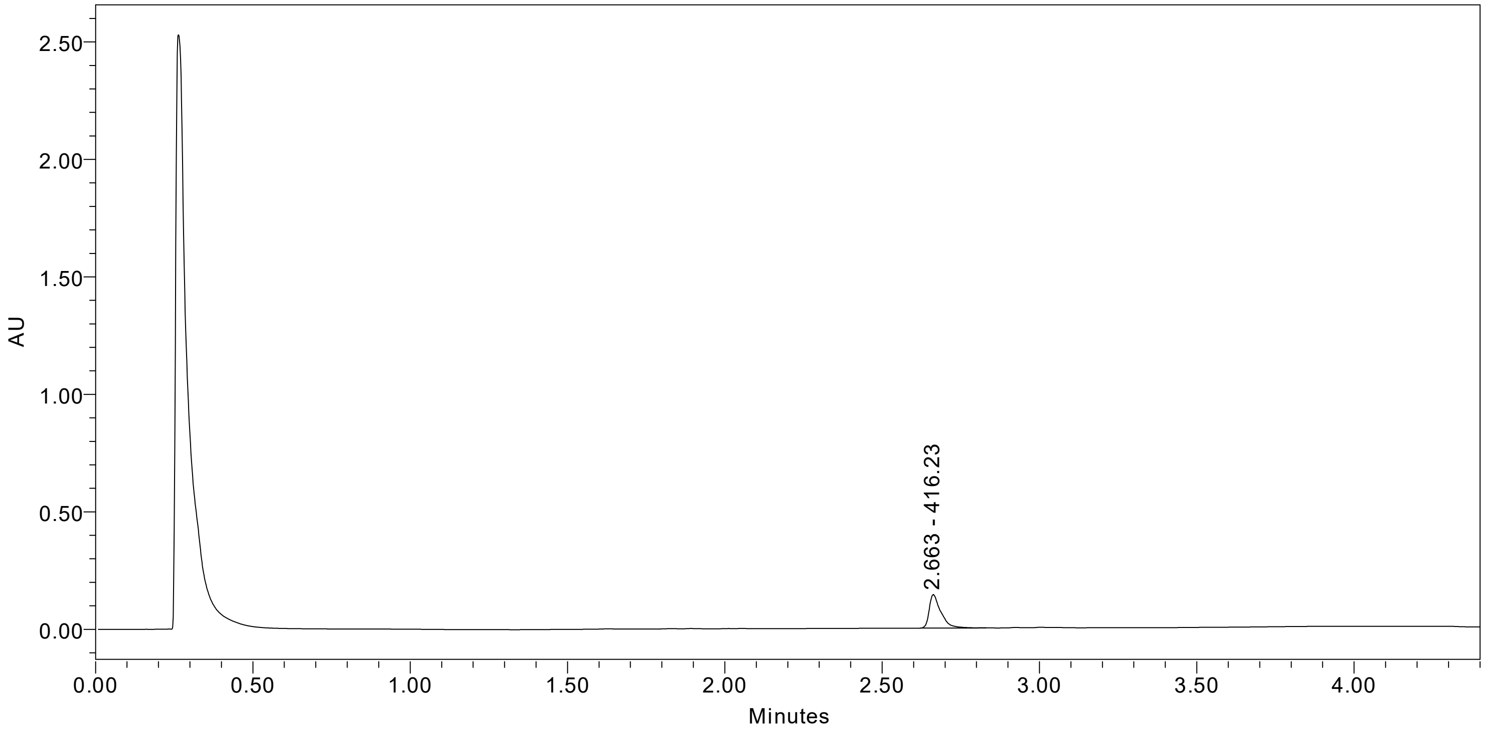

	RT	Area	% Area	Height	Base Peak (m/z)
1	2.663	367210	100.00	142535	416.23

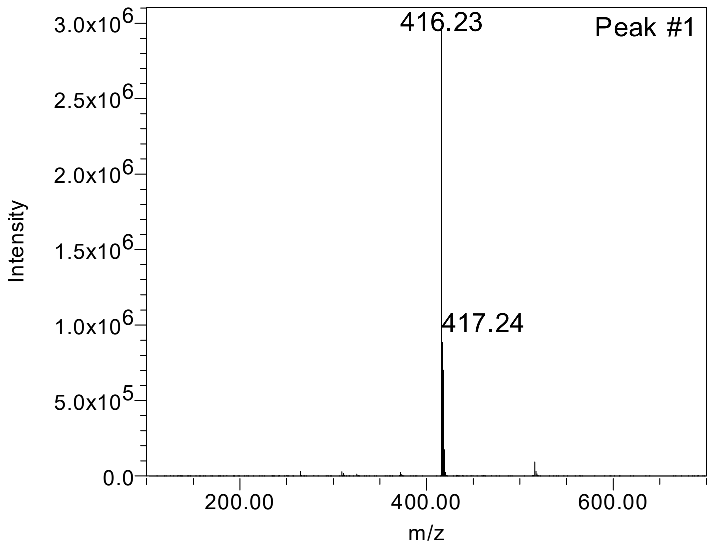

RT: 2.663

Mass Analysis Report

SAMPLE INFORMATION

Sample Name: SR211009A
Acq Method Set: Col1_MeOH_H2O_NH4HCO3
Acquired: 11/11/2021 4:36:39 PM CST
InjVol: 3.00 uL

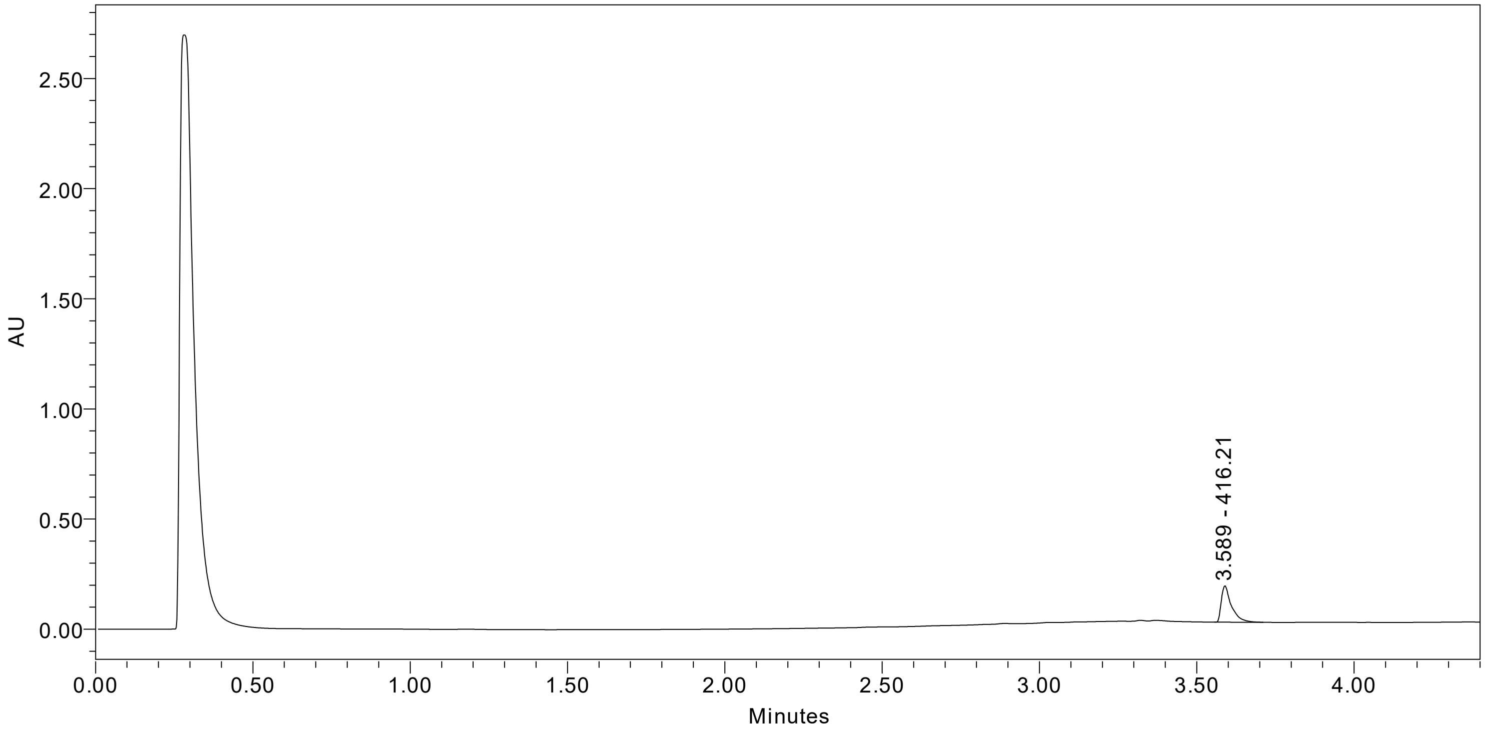

	RT	Area	% Area	Height	Base Peak (m/z)
1	3.589	367595	100.00	164451	416.21

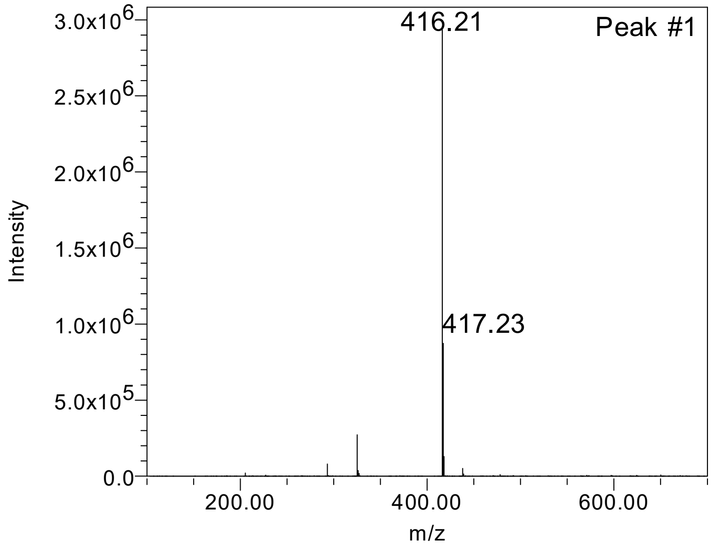

RT: 3.589

Single Injection Report

**Sample name:**	SR211217C		
**Description:**			
**Sample amount:**	0.000	**Sample type:**	Sample
**Instrument:**	LCMS	**Location:**	P1-A8
		**Injection:**	1 of 1
**Acq. method:**	Regular method.amx	**Injection volume:**	5.000 µL
**Analysis method:**	MS method-purity.pmx	**Acq. operator:**	SYSTEM

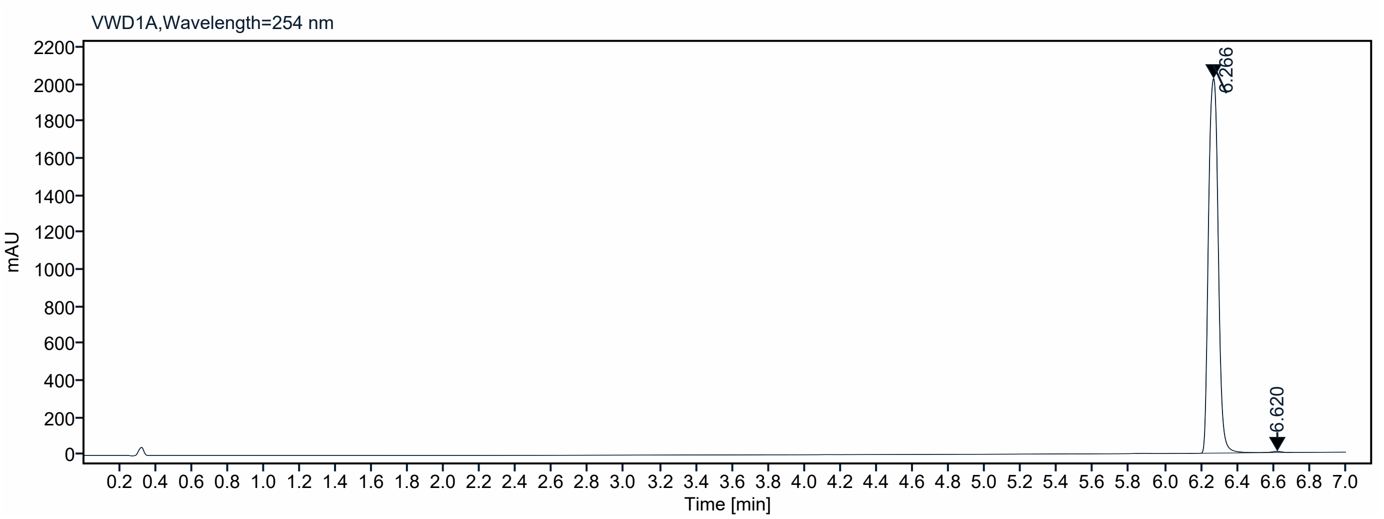

Signal: MS1 +TIC SCAN ESI Frag=110V Gain=1.0

RT [min]	Width [min]	Area	Height	Area%
6.324	0.2651	578047.2927	110357.0233	100.0000
	**Sum**	**578047.2927**		

Signal: VWD1A,Wavelength=254 nm

RT [min]	Width [min]	Area	Height	Area%
6.266	0.3919	7587.0722	2021.4264	99.6397
6.620	0.1844	27.4320	6.4199	0.3603
	**Sum**	**7614.5042**		

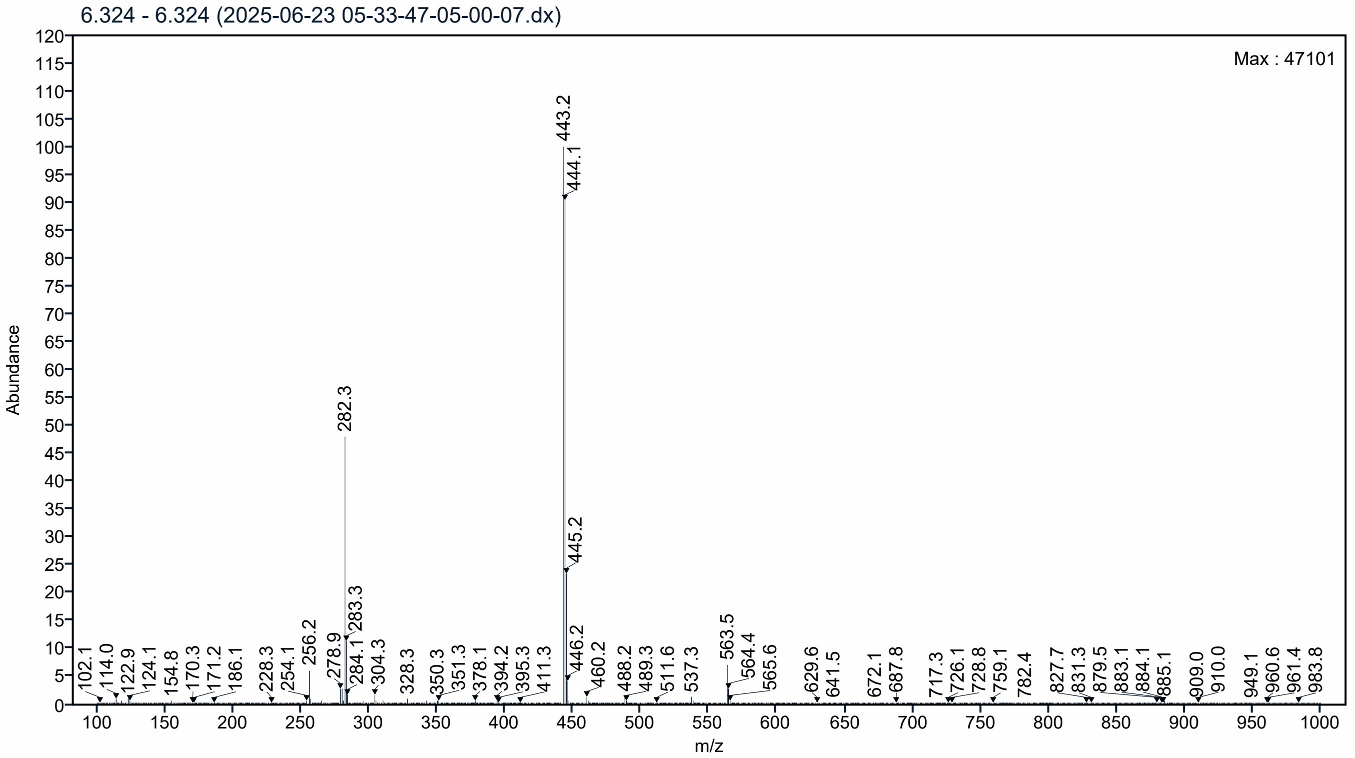
6.324 - 6.324 (2025-06-23 05-33-47-05-00-07.dx)
Max : 47101
Abundance
m/z
443.2
444.1
282.3
445.2
283.3
563.5
102.1
114.0
122.9
124.1
154.8
170.3
171.2
186.1
228.3
254.1
256.2
278.9
284.1
304.3
328.3
350.3
351.3
378.1
394.2
395.3
411.3
446.2
460.2
488.2
489.3
511.6
537.3
564.4
565.6
629.6
641.5
672.1
687.8
717.3
726.1
728.8
759.1
782.4
827.7
831.3
879.5
883.1
884.1
885.1
909.0
910.0
949.1
960.6
961.4
983.8

Mass Analysis Report

SAMPLE INFORMATION

Sample Name: SR210420A
Acq Method Set: Col2_MeCN_H2O_NH4HCO3
Acquired: 5/14/2021 6:27:47 PM CDT
InjVol: 7.50 uL

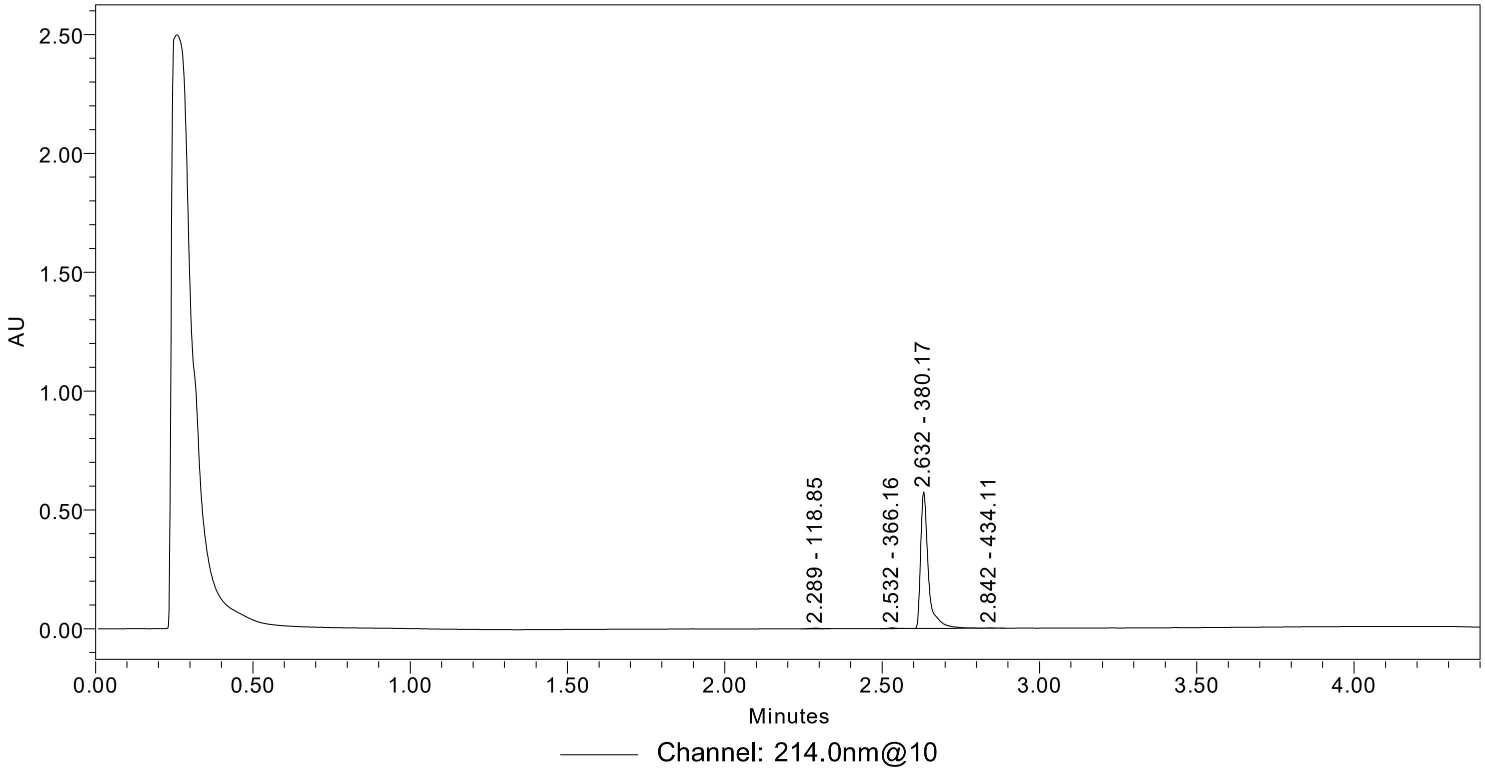

	RT	Area	% Area	Height	Base Peak (m/z)
1	2.289	4841	0.48	2338	118.85
2	2.532	4512	0.45	3006	366.16
3	2.632	990130	98.68	574194	380.17
4	2.842	3851	0.38	1595	434.11

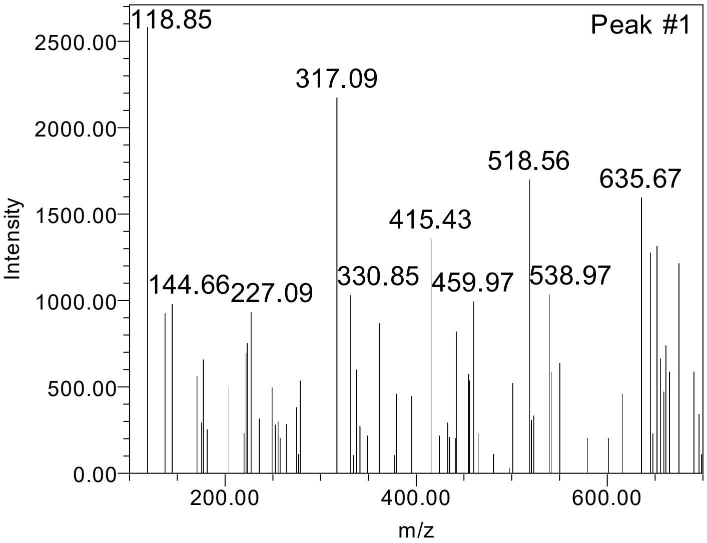

RT: 2.289

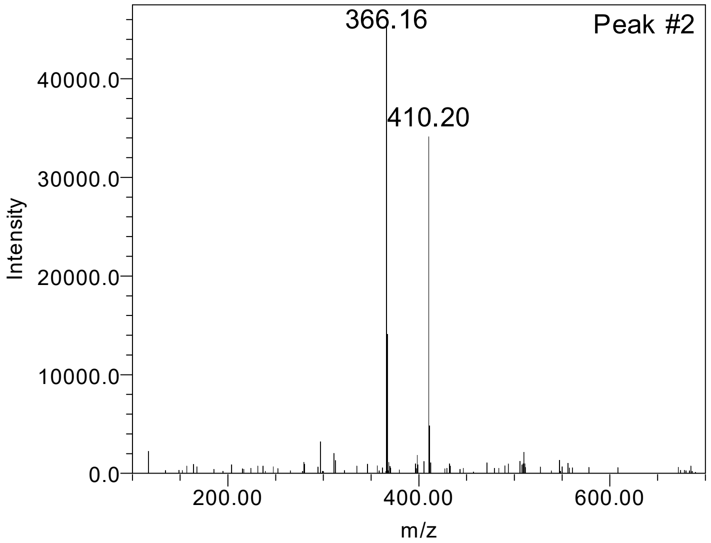

RT: 2.532

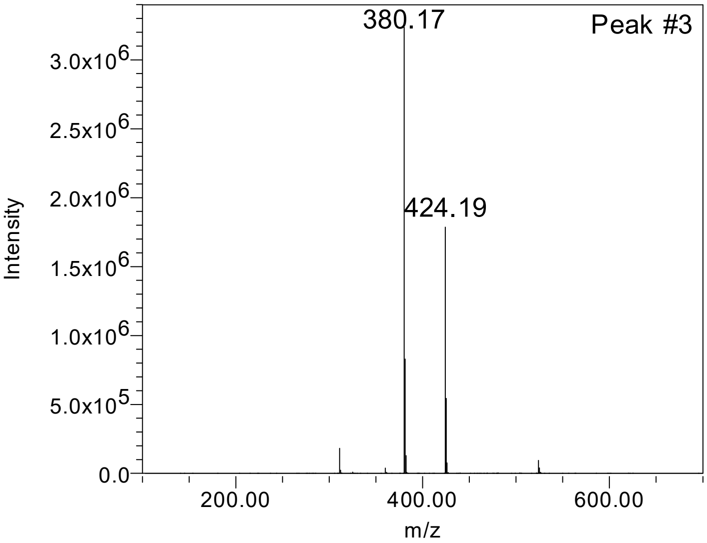

RT: 2.632

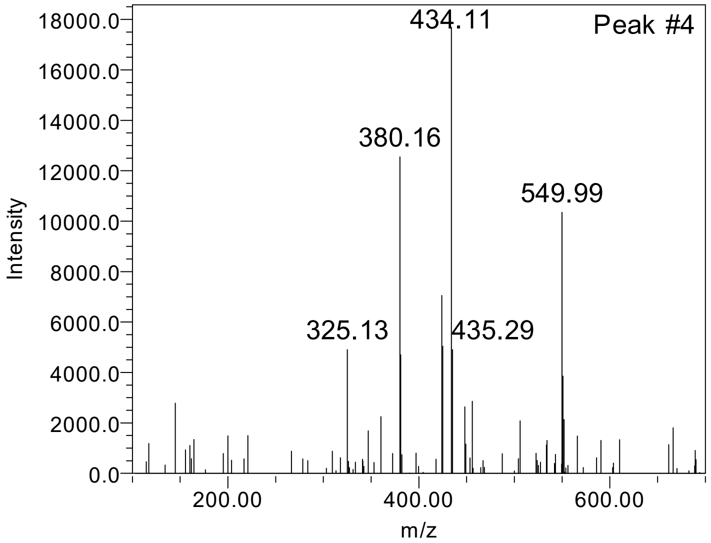

RT: 2.842

Mass Analysis Report

SAMPLE INFORMATION

Sample Name: SR210420A
Acq Method Set: Col1_MeOH_H2O_NH4HCO3
Acquired: 5/15/2021 1:08:20 AM CDT
InjVol: 7.50 uL

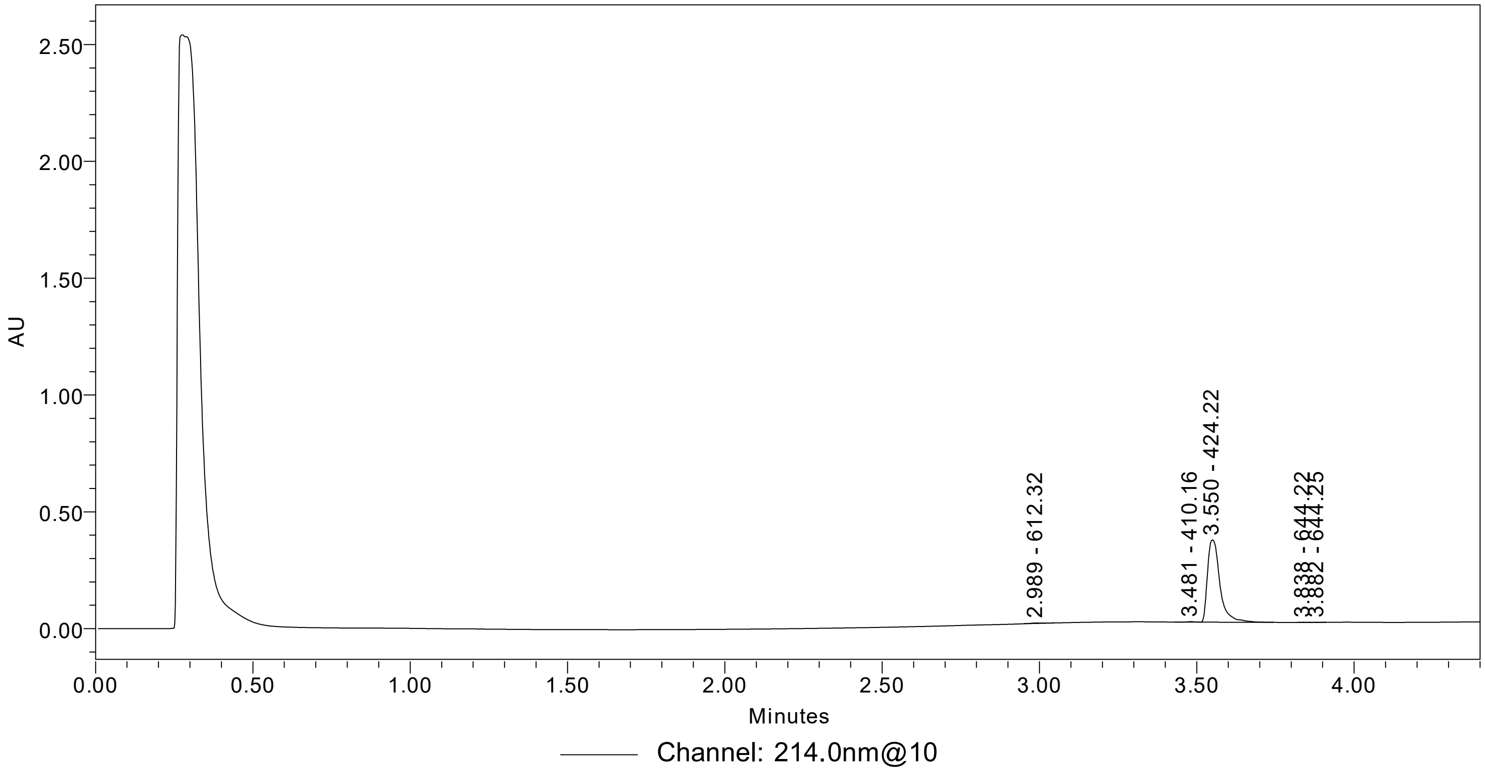

	RT	Area	% Area	Height	Base Peak (m/z)
1	2.989	4684	0.48	1648	612.32
2	3.481	3611	0.37	1765	410.16
3	3.550	966327	98.90	351574	424.22
4	3.838	889	0.09	448	644.22
5	3.882	1562	0.16	647	644.25

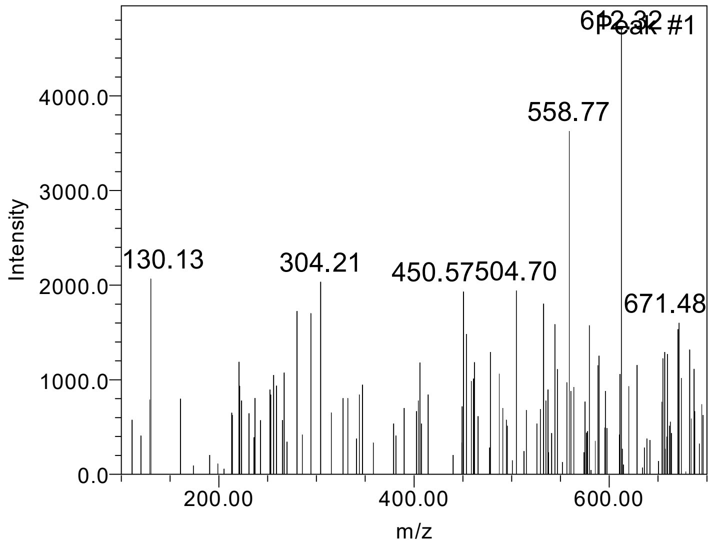

RT: 2.989

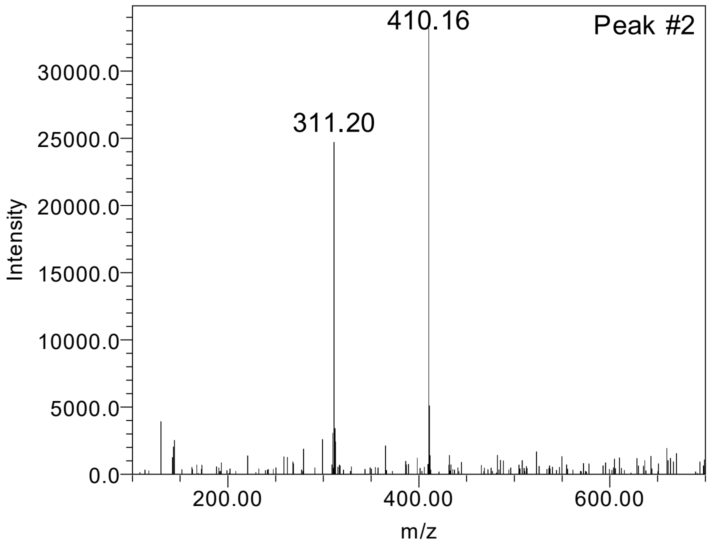

RT: 3.481

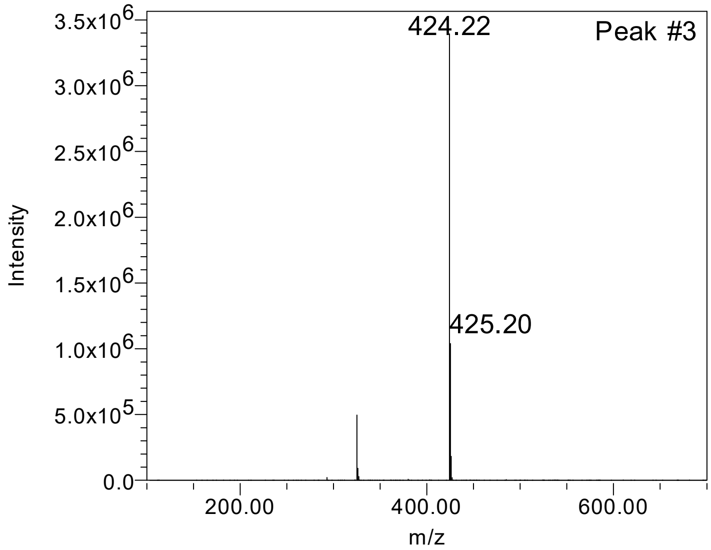

RT: 3.550

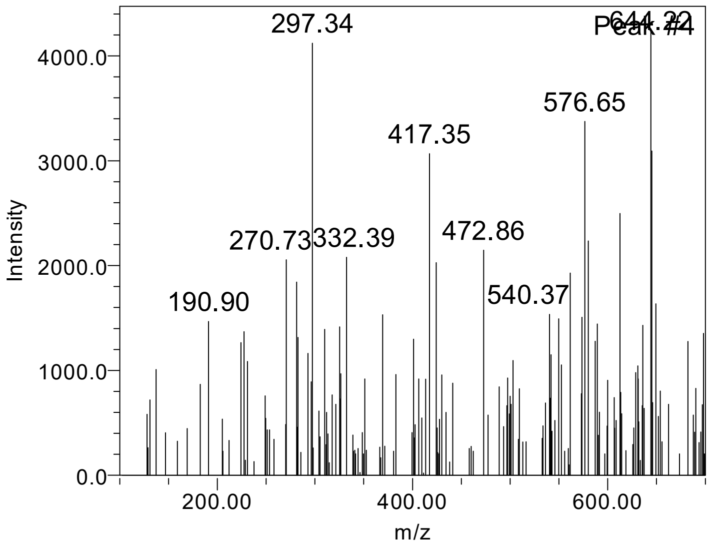

RT: 3.838

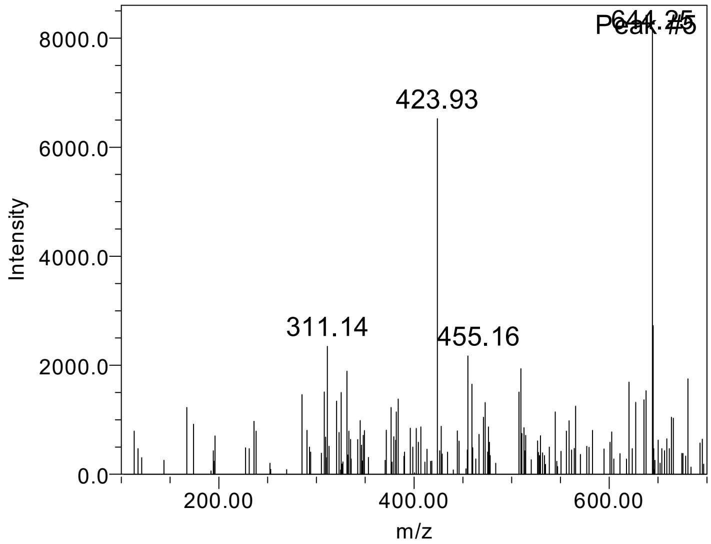

RT: 3.882

Mass Analysis Report

SAMPLE INFORMATION

Sample Name: SR210420C
Acq Method Set: Col2_MeCN_H2O_NH4HCO3
Acquired: 5/14/2021 6:34:50 PM CDT
InjVol: 7.50 uL

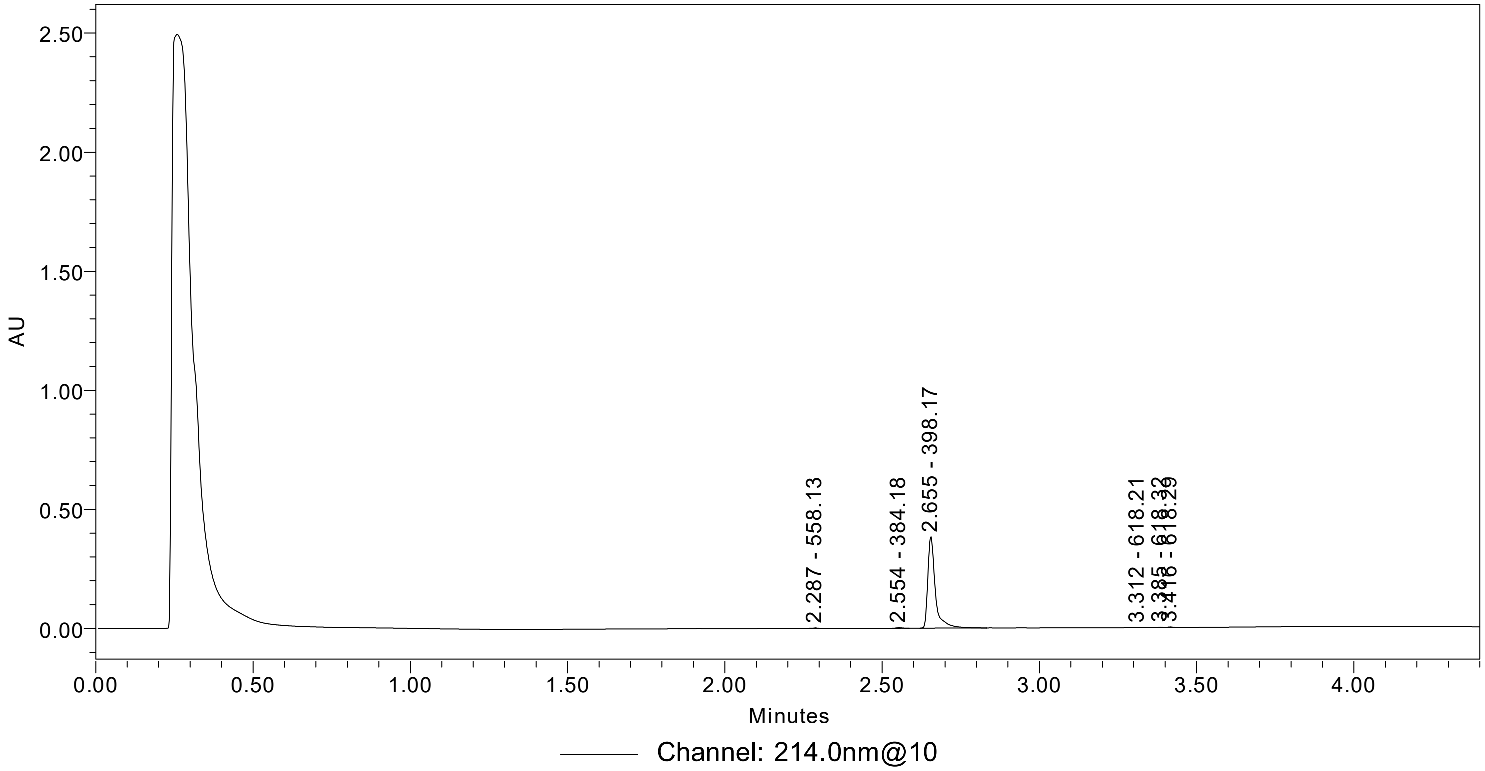

	RT	Area	% Area	Height	Base Peak (m/z)
1	2.287	4104	0.63	1989	558.13
2	2.554	3494	0.54	2449	384.18
3	2.655	634156	97.87	382862	398.17
4	3.312	1765	0.27	1202	618.21
5	3.385	1900	0.29	1546	618.32
6	3.416	2572	0.40	2221	618.29

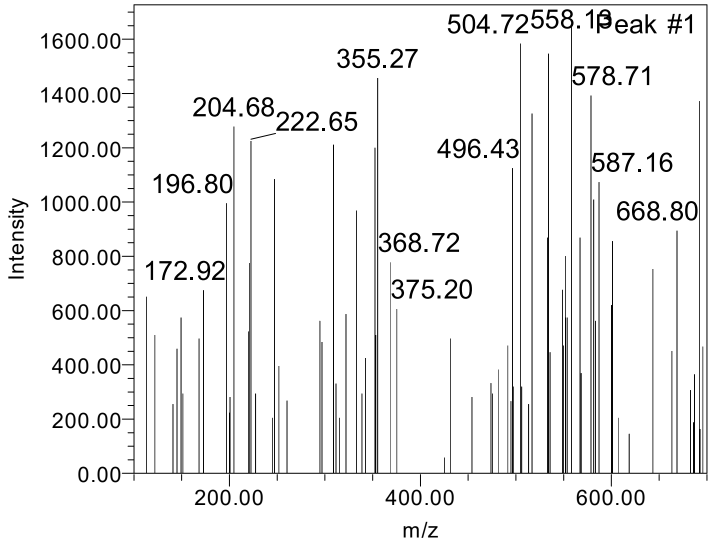

RT: 2.287

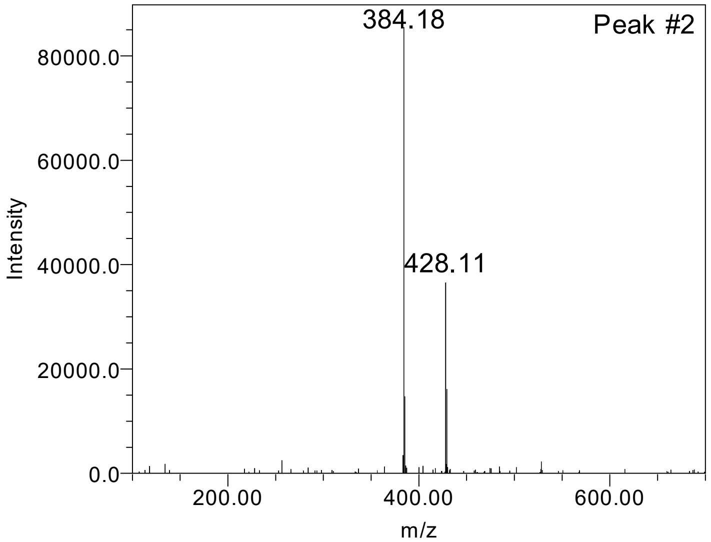

RT: 2.554

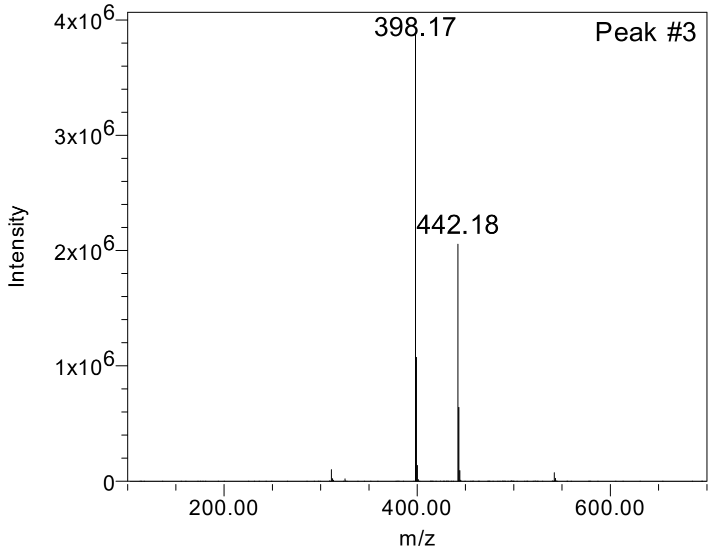

RT: 2.655

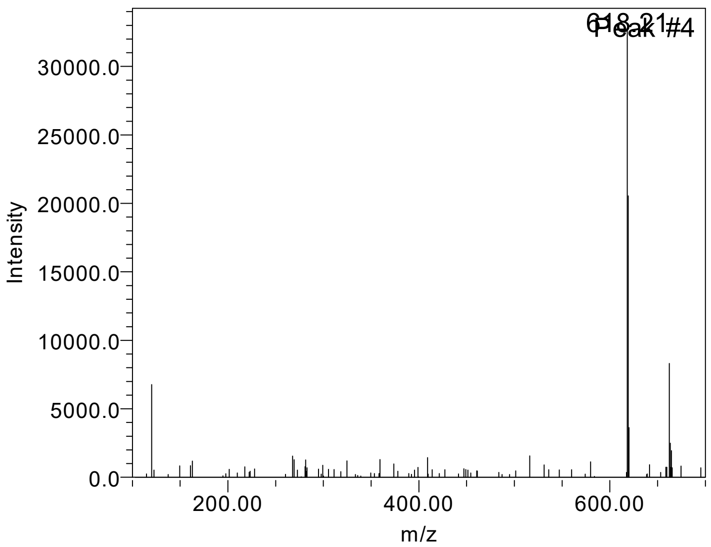

RT: 3.312

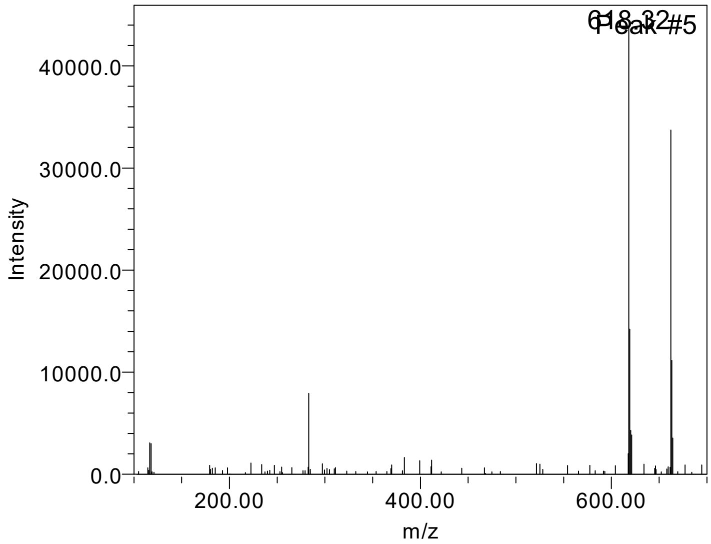

RT: 3.385

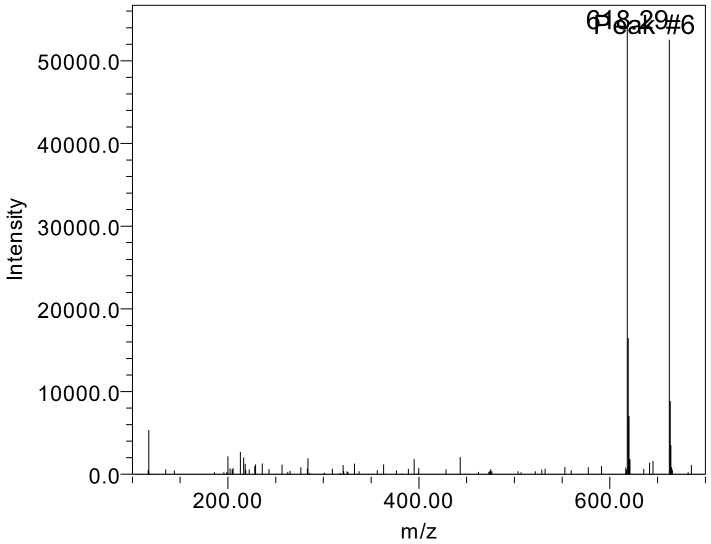

RT: 3.416

Mass Analysis Report

SAMPLE INFORMATION

Sample Name: SR210420C
Acq Method Set: Col1_MeOH_H2O_NH4HCO3
Acquired: 5/15/2021 1:15:23 AM CDT
InjVol: 7.50 uL

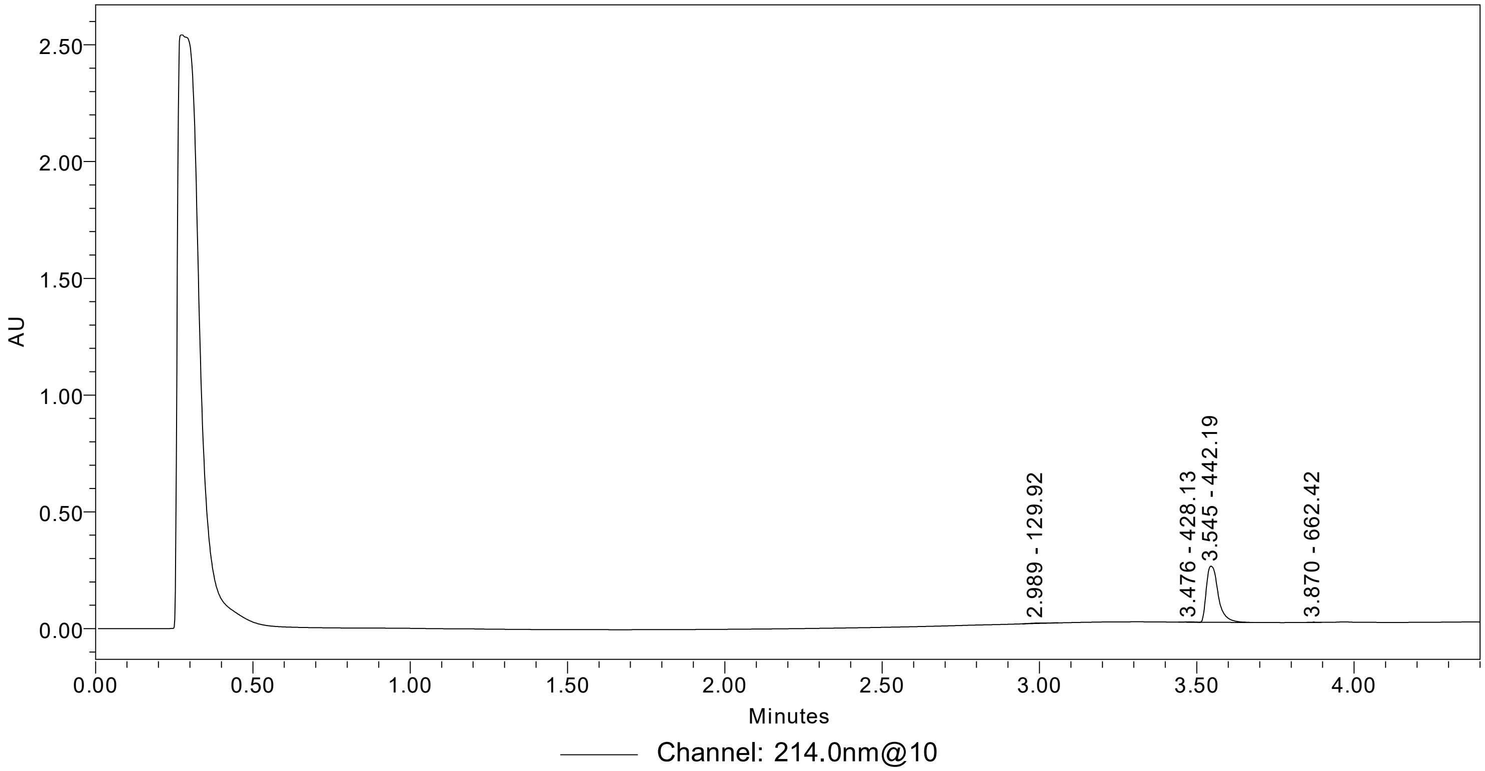

	RT	Area	% Area	Height	Base Peak (m/z)
1	2.989	4891	0.76	1522	129.92
2	3.476	3291	0.51	1627	428.13
3	3.545	632136	98.45	240487	442.19
4	3.870	1792	0.28	953	662.42

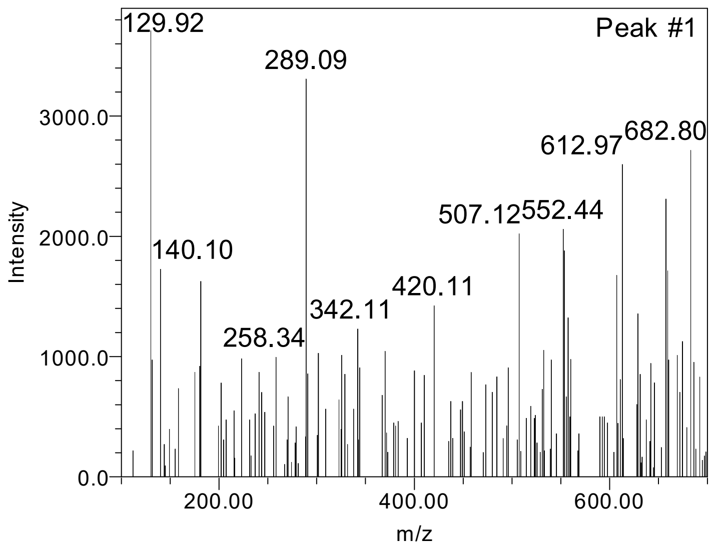

RT: 2.989

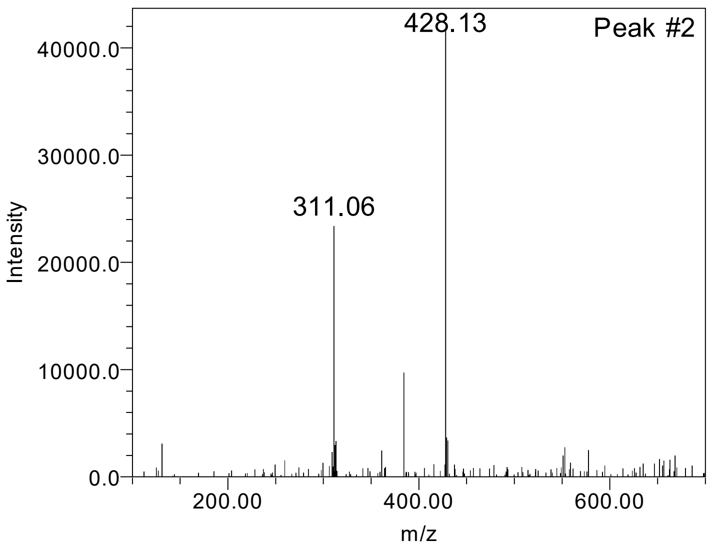

RT: 3.476

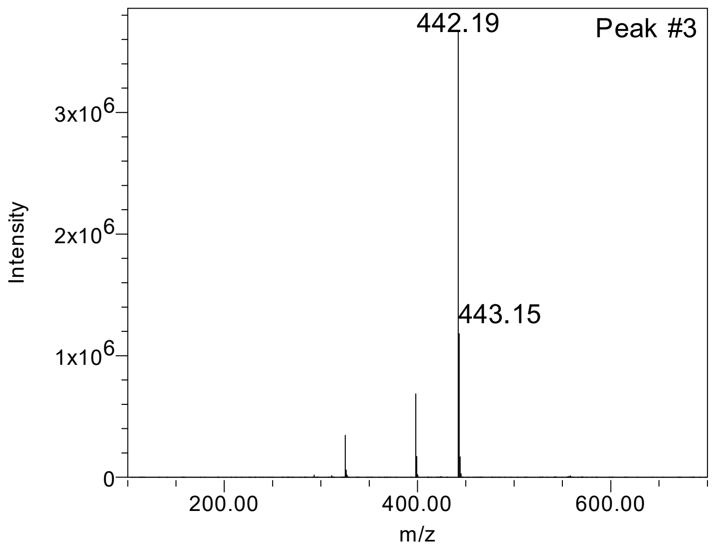

RT: 3.545

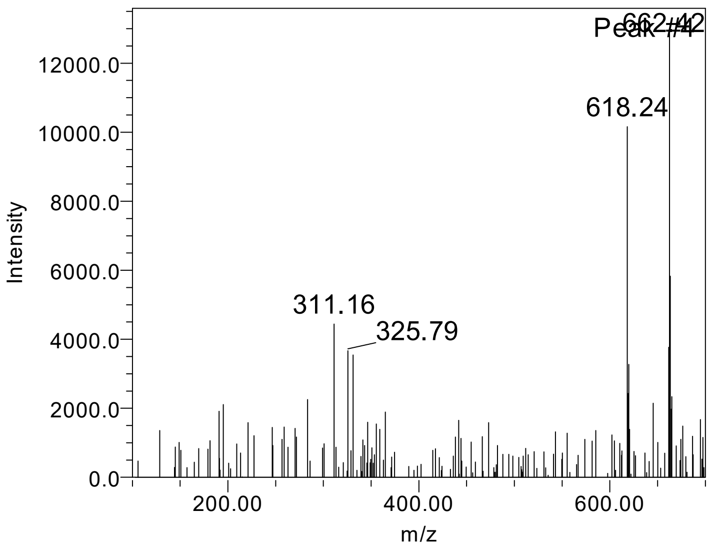

RT: 3.870

Mass Analysis Report

SAMPLE INFORMATION

Sample Name: SR211202A
Acq Method Set: Col2_MeCN_H2O_NH4HCO3
Acquired: 12/21/2021 7:58:57 PM CST
InjVol: 7.50 uL

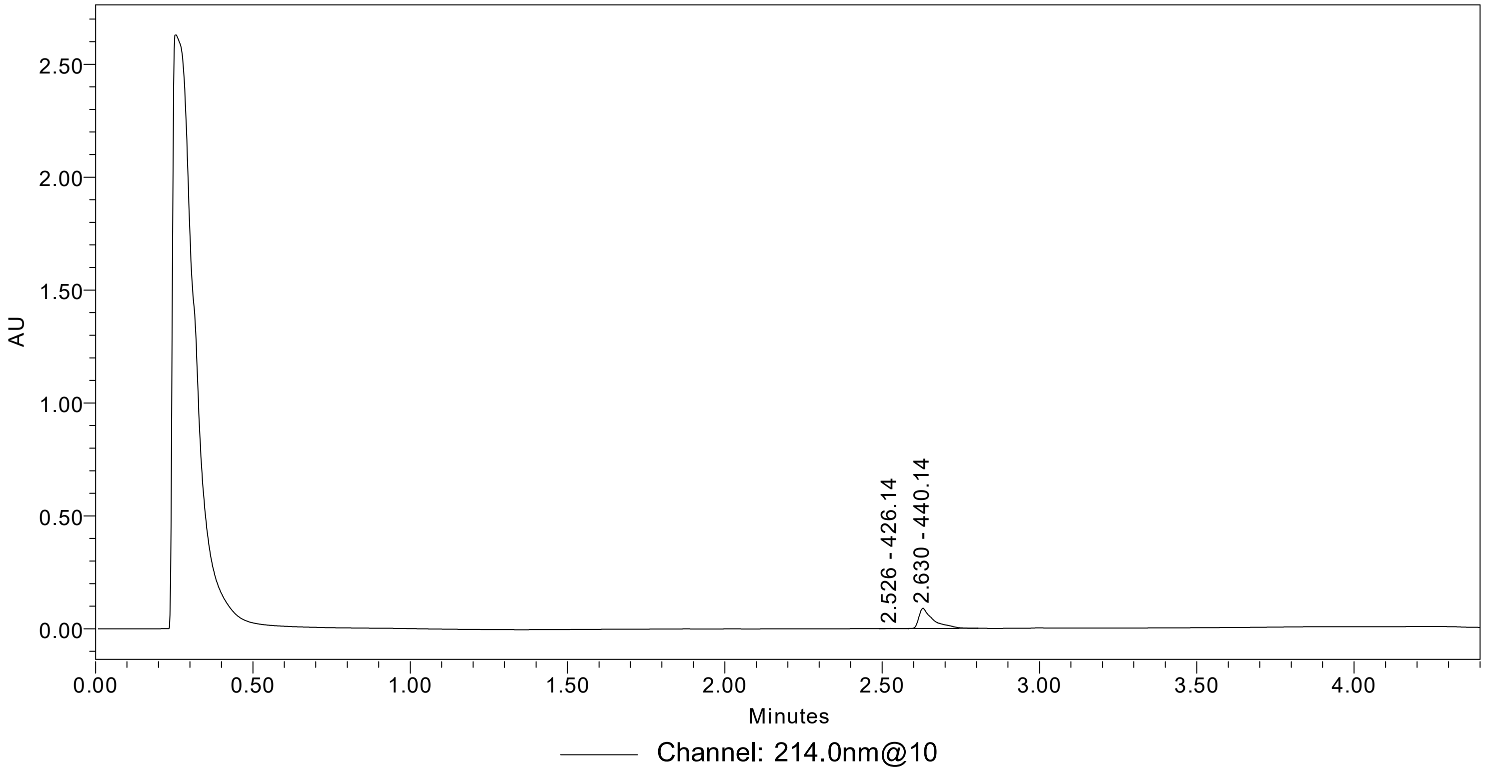

	RT	Area	% Area	Height	Base Peak (m/z)
1	2.526	2301	0.81	1013	426.14
2	2.630	283289	99.19	89189	440.14

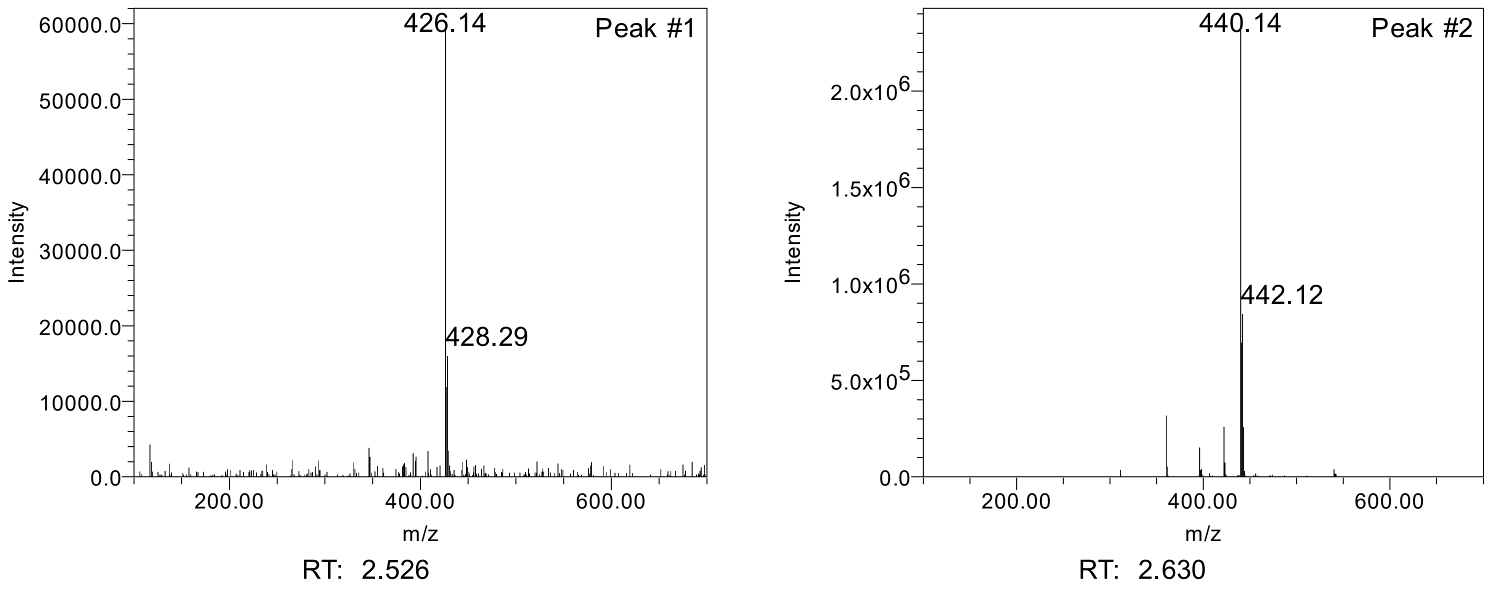
Peak #1
426.14
428.29
Intensity
60000.0
50000.0
40000.0
30000.0
20000.0
10000.0
0.0
200.00
400.00
600.00
m/z
RT: 2.526
Peak #2
440.14
442.12
Intensity
$2.0x10^6$
$1.5x10^6$
$1.0x10^6$
$5.0x10^5$
0.0
200.00
400.00
600.00
m/z
RT: 2.630

Mass Analysis Report

SAMPLE INFORMATION

Sample Name: SR211202A
Acq Method Set: Col1_MeOH_H2O_NH4HCO3
Acquired: 12/22/2021 2:45:02 AM CST
InjVol: 7.50 uL

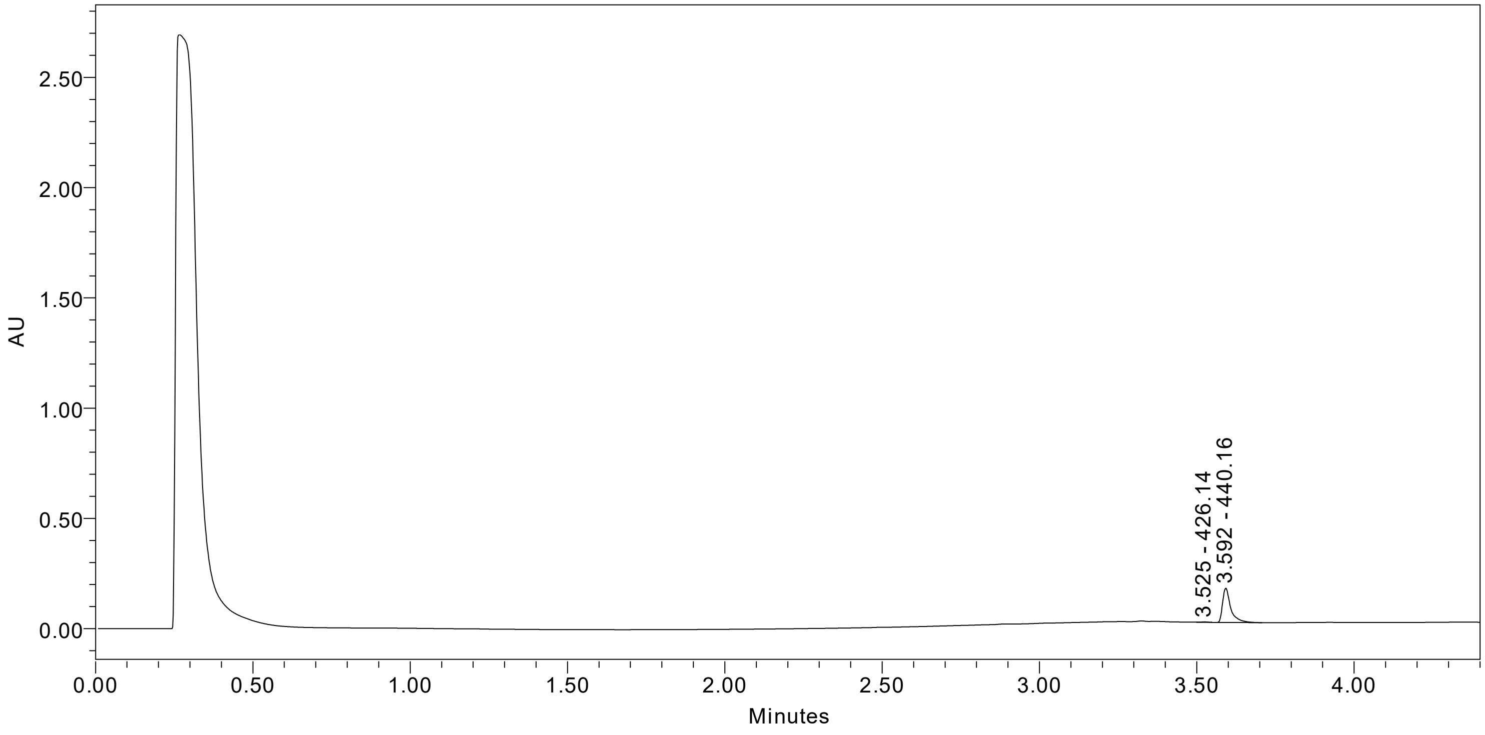

	RT	Area	% Area	Height	Base Peak (m/z)
1	3.525	2467	0.87	1682	426.14
2	3.592	280519	99.13	155032	440.16

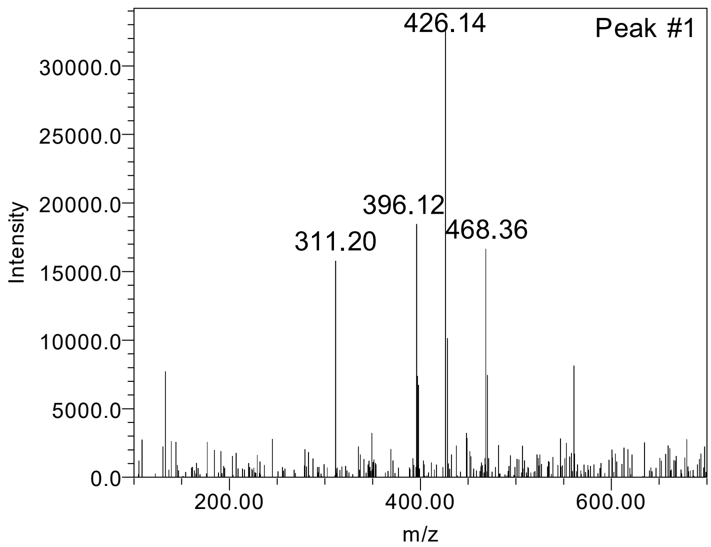

RT: 3.525

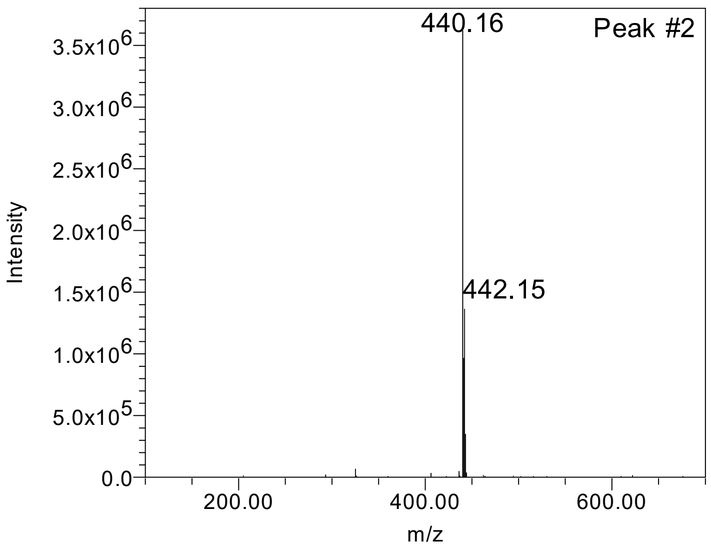

RT: 3.592

Mass Analysis Report

SAMPLE INFORMATION

Sample Name: SR211202B
Acq Method Set: Col2_MeCN_H2O_NH4HCO3
Acquired: 12/21/2021 8:06:00 PM CST
InjVol: 7.50 uL

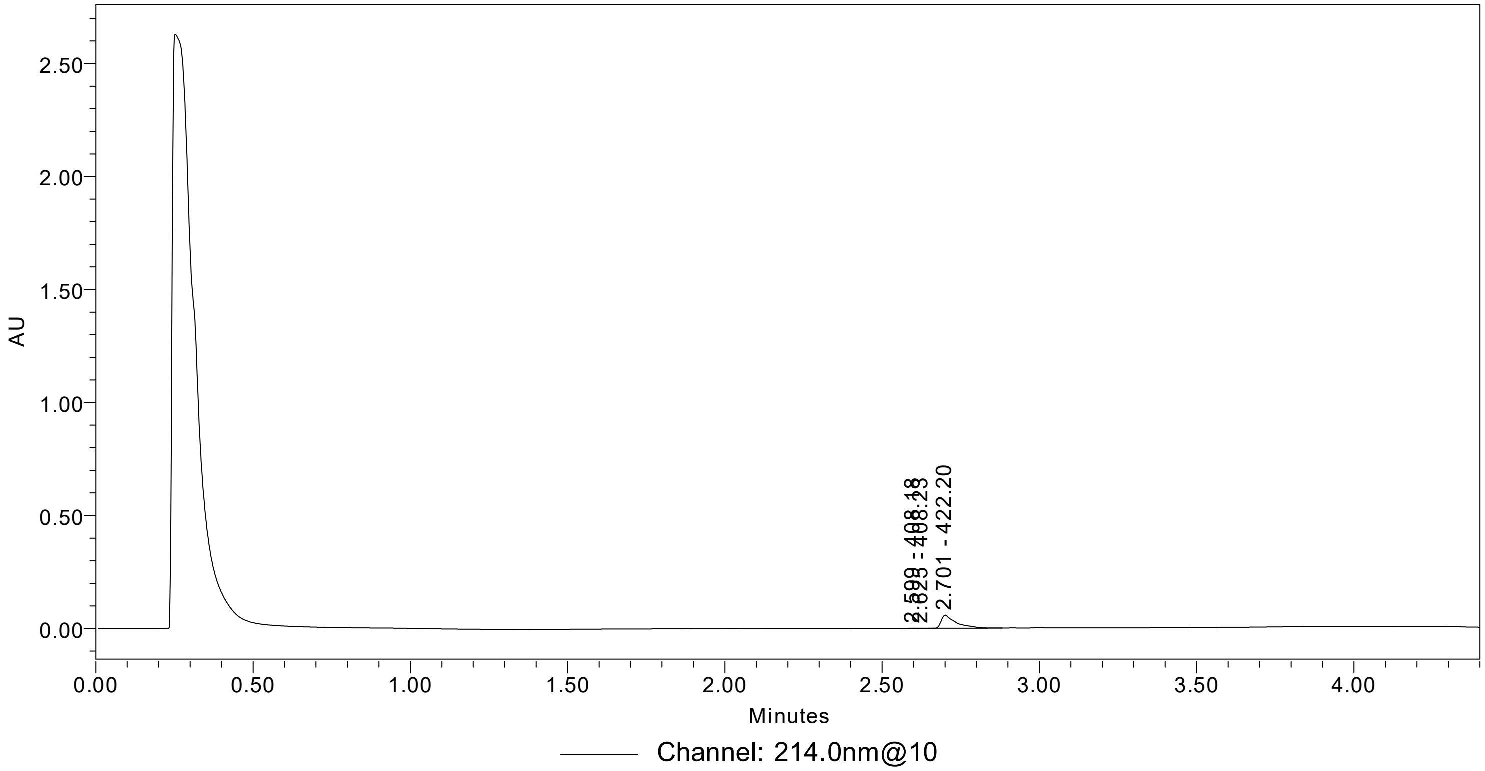

	RT	Area	% Area	Height	Base Peak (m/z)
1	2.599	1016	0.53	651	408.18
2	2.625	1407	0.73	625	408.23
3	2.701	189494	98.74	58247	422.20

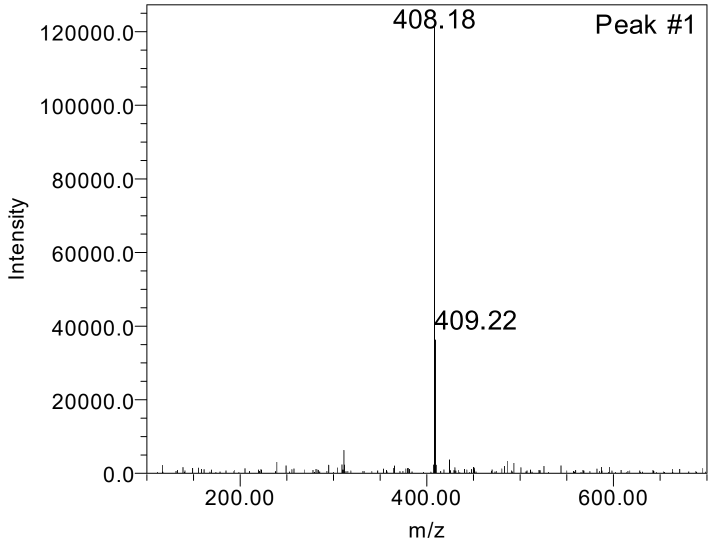

RT: 2.599

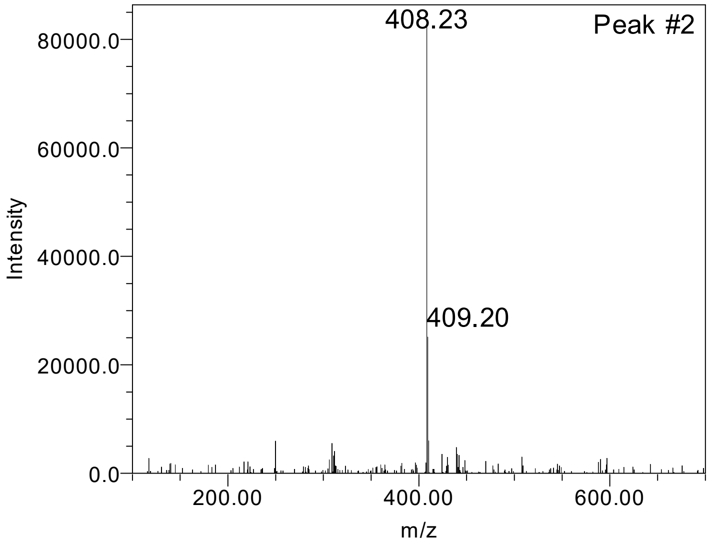

RT: 2.625

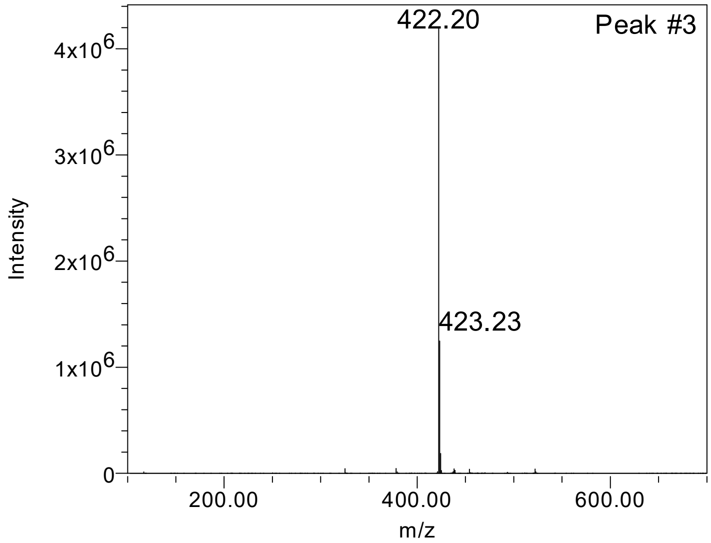

RT: 2.701

Mass Analysis Report

SAMPLE INFORMATION

Sample Name: SR211202B
Acq Method Set: Col1_MeOH_H2O_NH4HCO3
Acquired: 12/22/2021 2:52:06 AM CST
InjVol: 7.50 uL

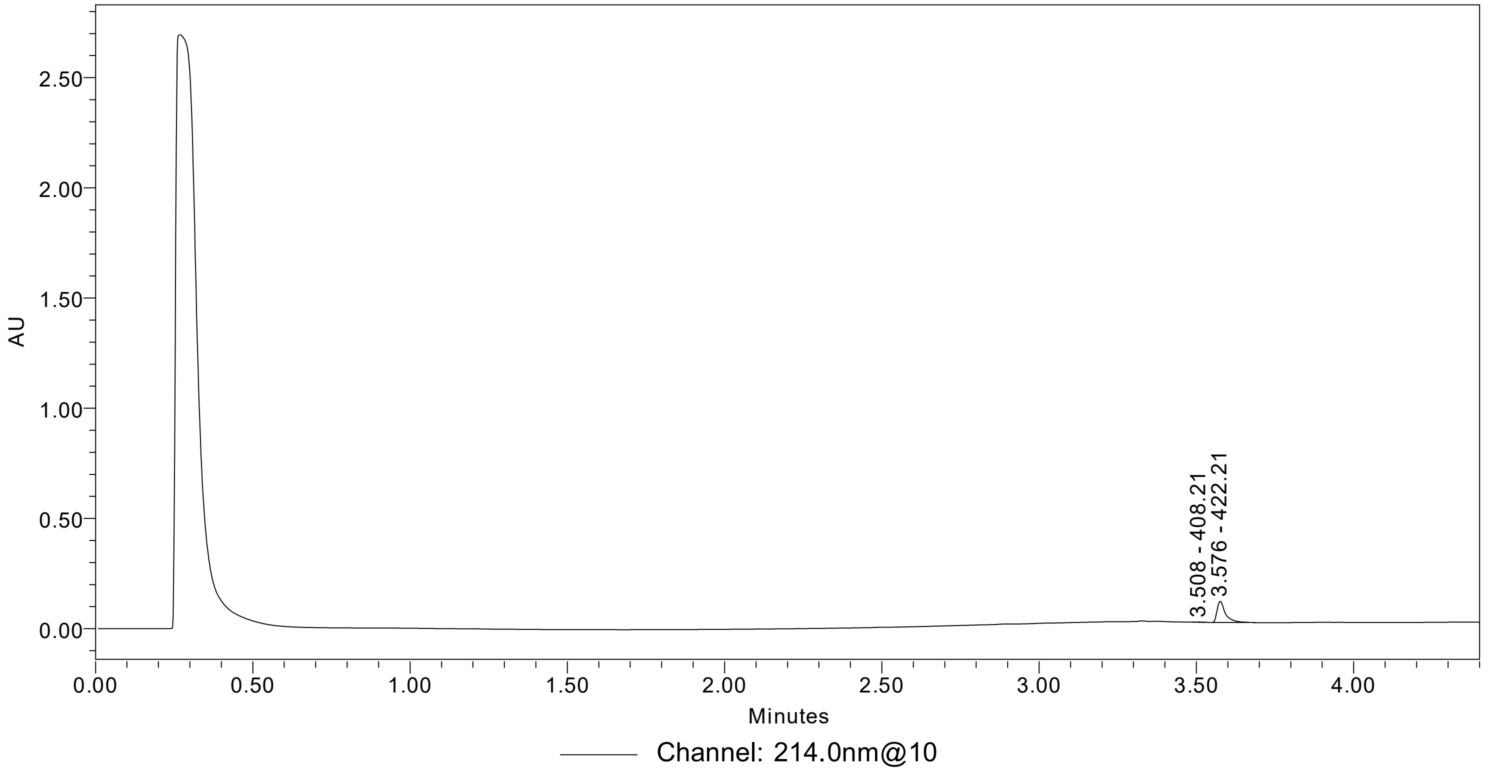

	RT	Area	% Area	Height	Base Peak (m/z)
1	3.508	1201	0.67	923	408.21
2	3.576	179160	99.33	95586	422.21

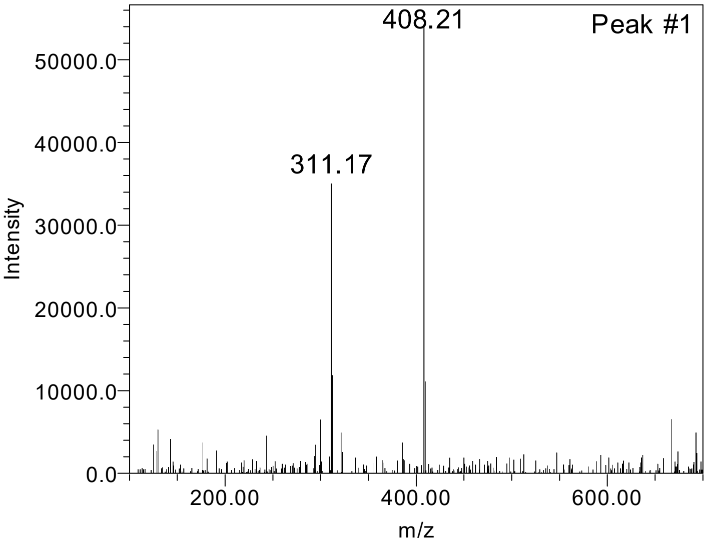

RT: 3.508

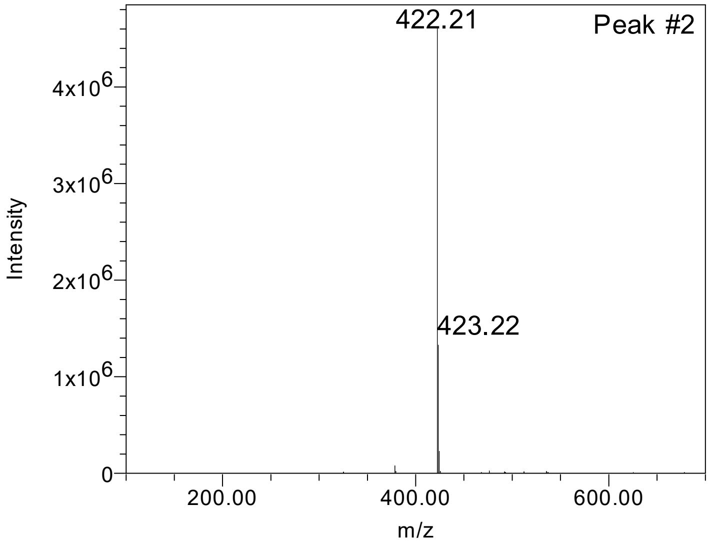

RT: 3.576

Mass Analysis Report

SAMPLE INFORMATION

Sample Name: SR210708B
Acq Method Set: Col2_MeCN_H2O_NH4HCO3
Acquired: 7/15/2021 7:39:26 PM CDT
InjVol: 7.50 uL

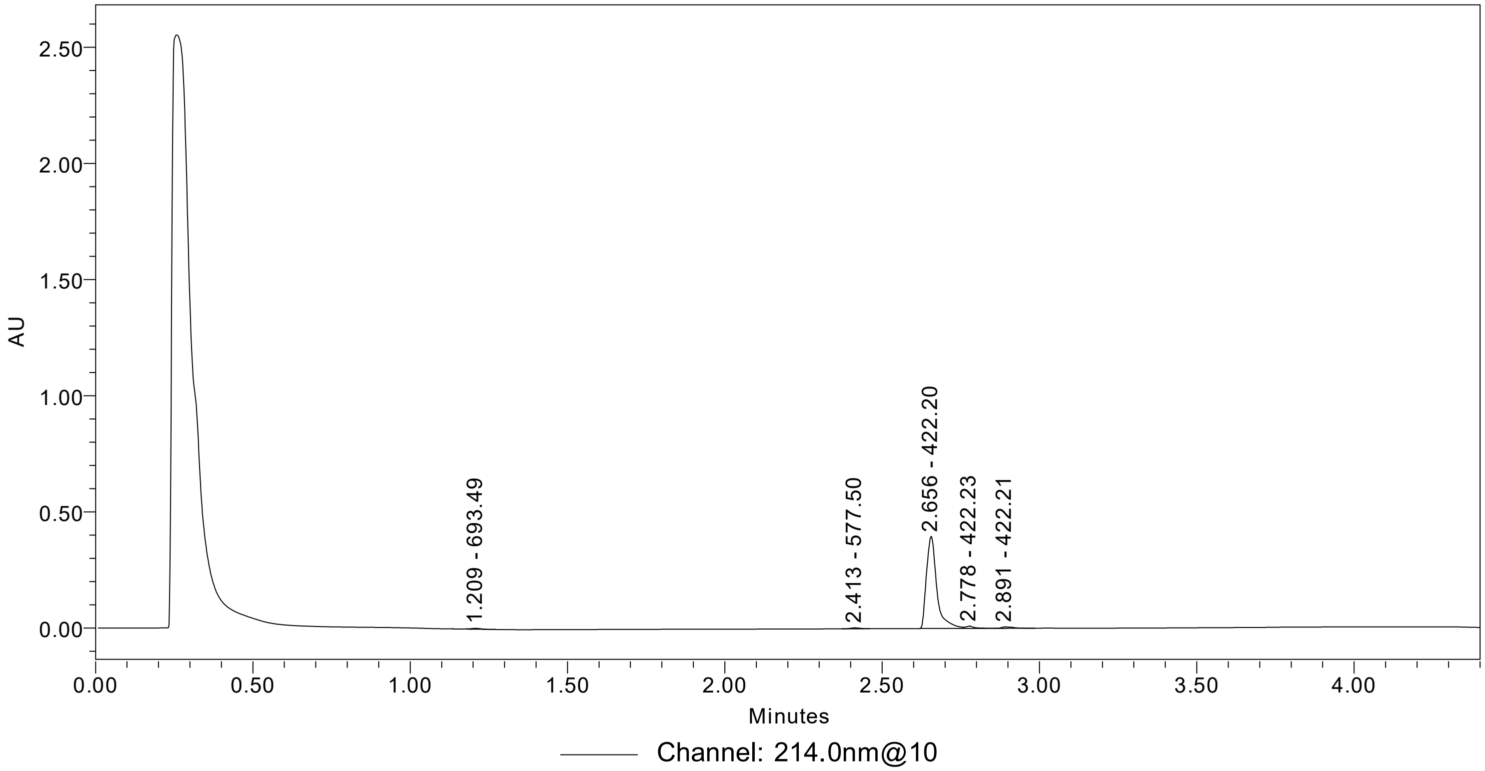

	RT	Area	% Area	Height	Base Peak (m/z)
1	1.209	8885	0.93	3773	693.49
2	2.413	8122	0.85	3878	577.50
3	2.656	906227	94.37	396305	422.20
4	2.778	20143	2.10	9704	422.23
5	2.891	16949	1.76	6220	422.21

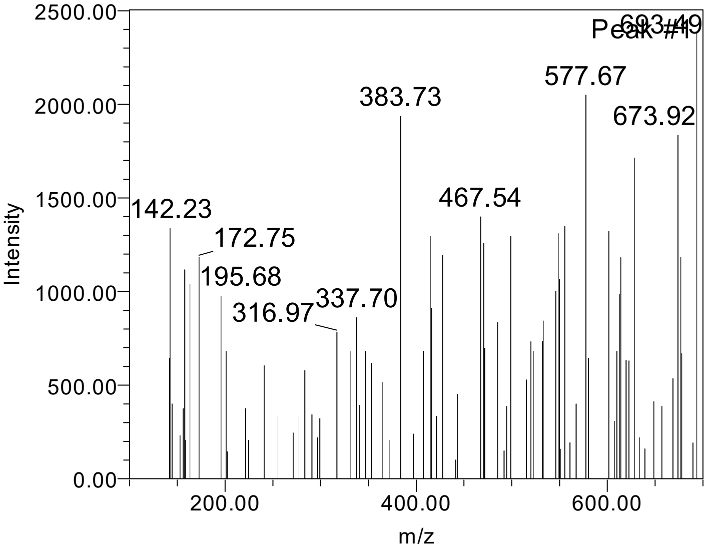

RT: 1.209

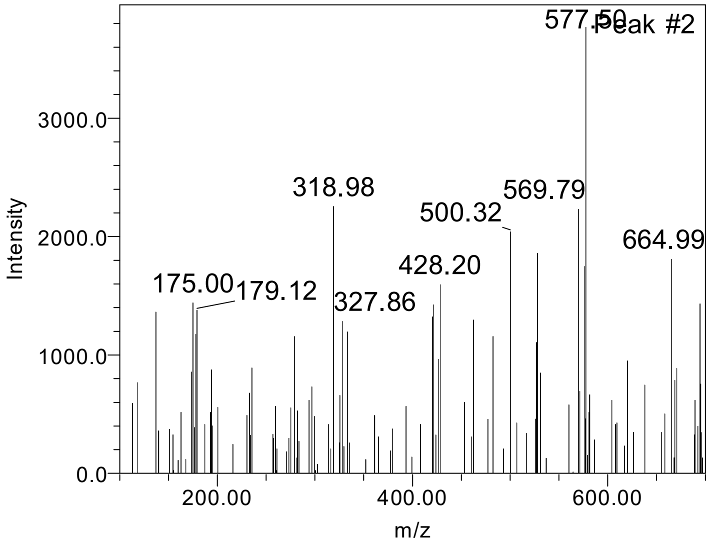

RT: 2.413

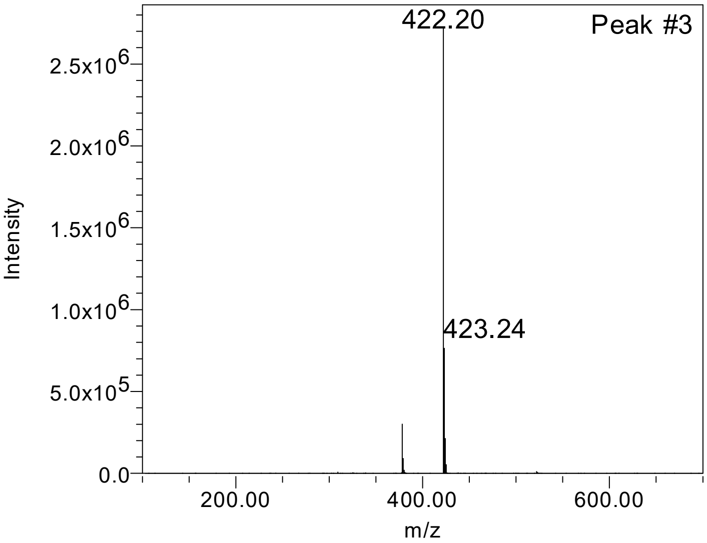

RT: 2.656

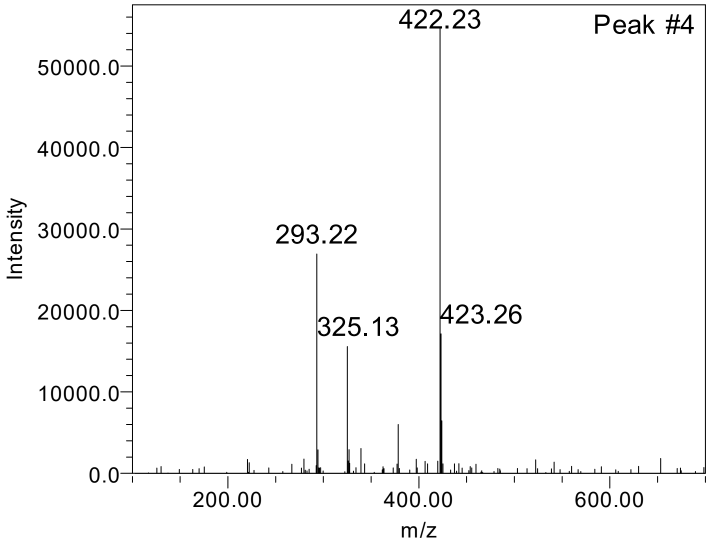

RT: 2.778

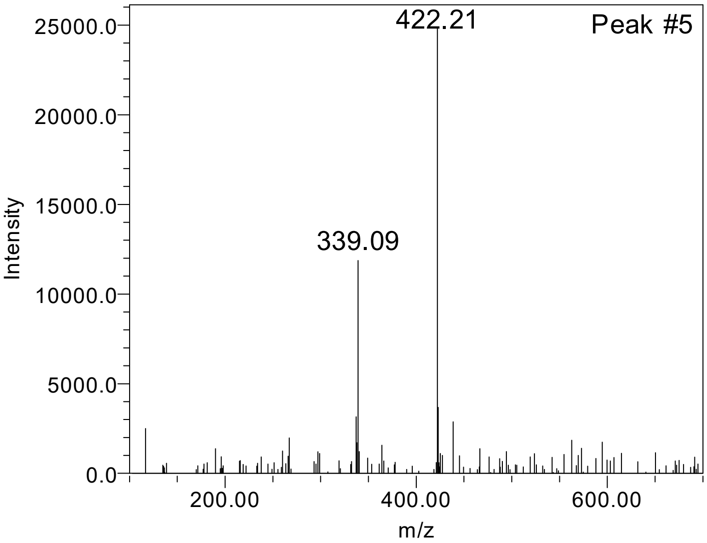

RT: 2.891

Mass Analysis Report

SAMPLE INFORMATION

Sample Name: SR210708B
Acq Method Set: Col1_MeOH_H2O_NH4HCO3
Acquired: 7/16/2021 8:33:31 PM CDT
InjVol: 7.50 uL

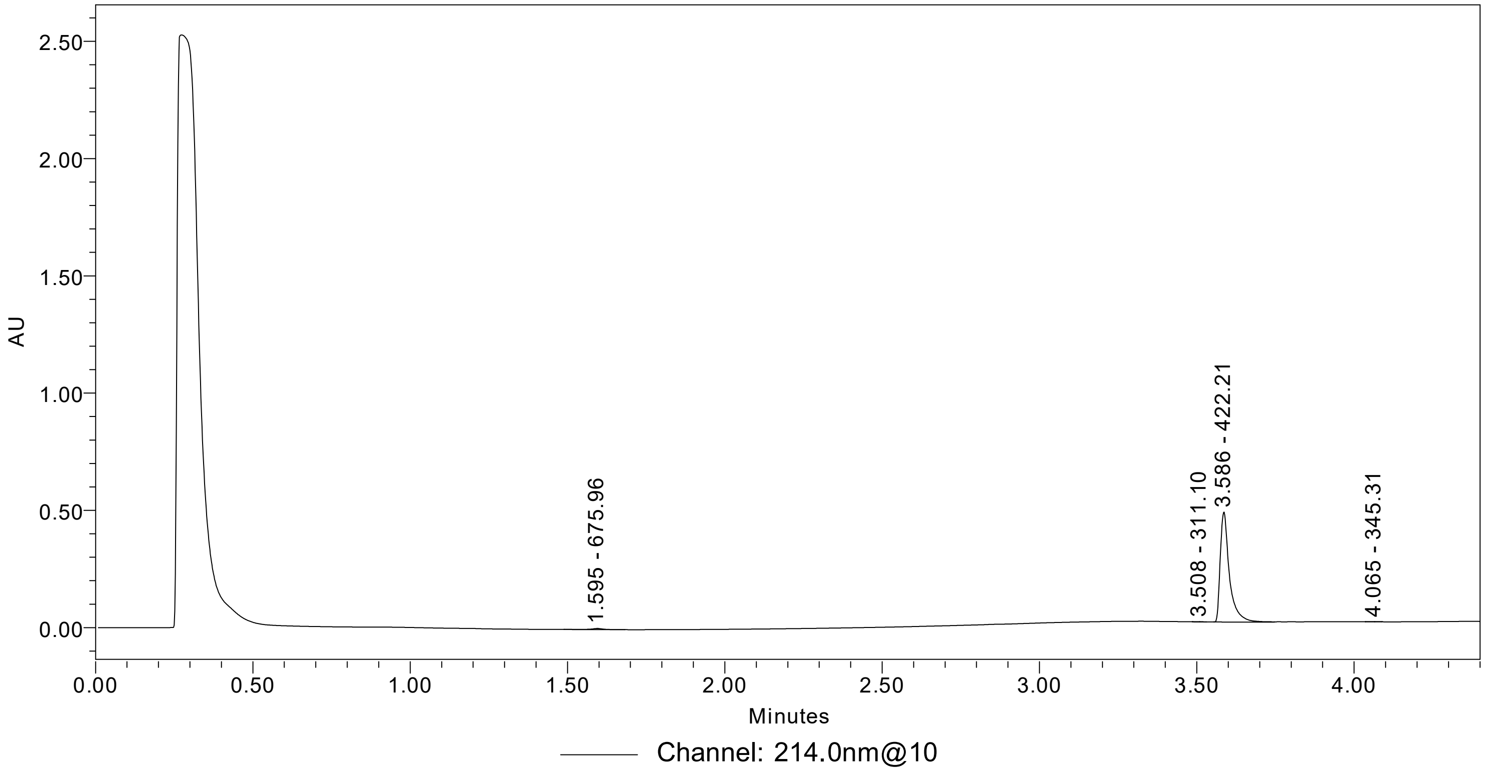

	RT	Area	% Area	Height	Base Peak (m/z)
1	1.595	12586	1.31	4640	675.96
2	3.508	1287	0.13	932	311.10
3	3.586	943678	98.51	468577	422.21
4	4.065	434	0.05	297	345.31

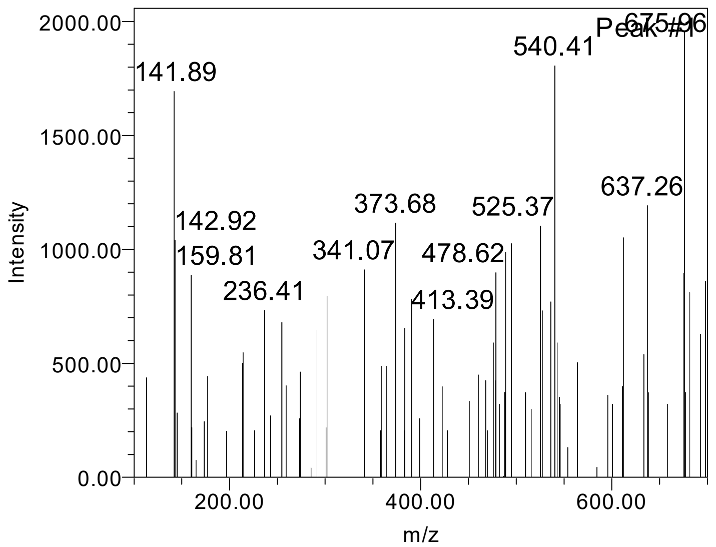

RT: 1.595

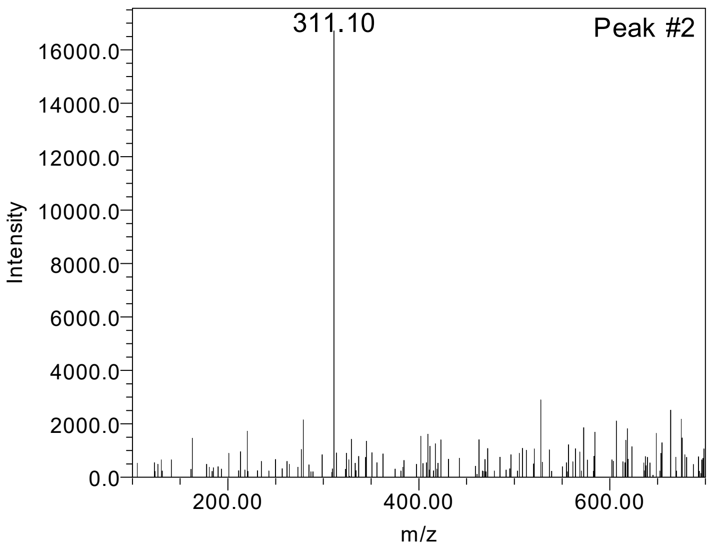

RT: 3.508

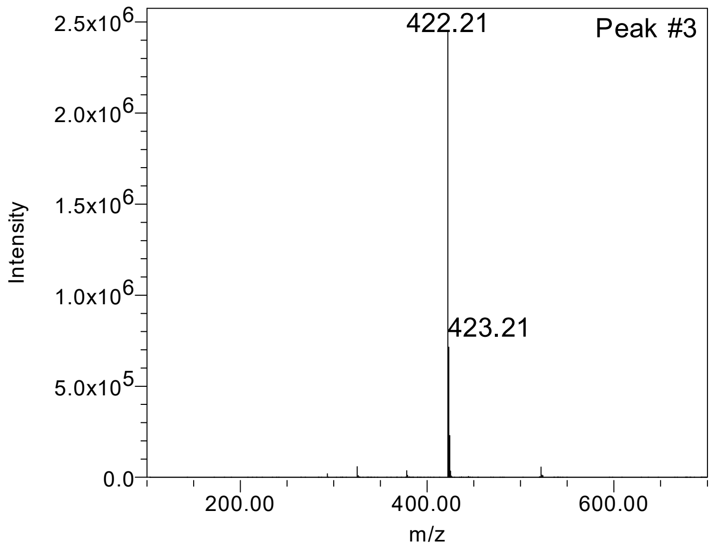

RT: 3.586

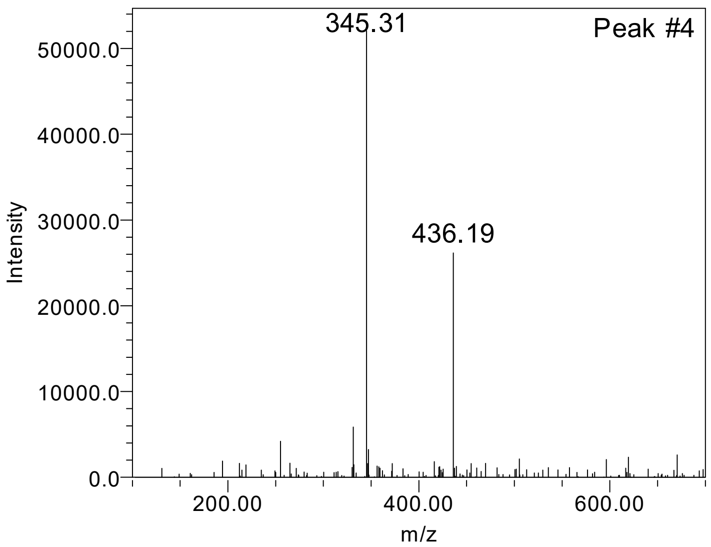

RT: 4.065

Mass Analysis Report

SAMPLE INFORMATION

Sample Name: SR210708A
Acq Method Set: Col2_MeCN_H2O_NH4HCO3
Acquired: 7/15/2021 7:32:23 PM CDT
InjVol: 7.50 uL

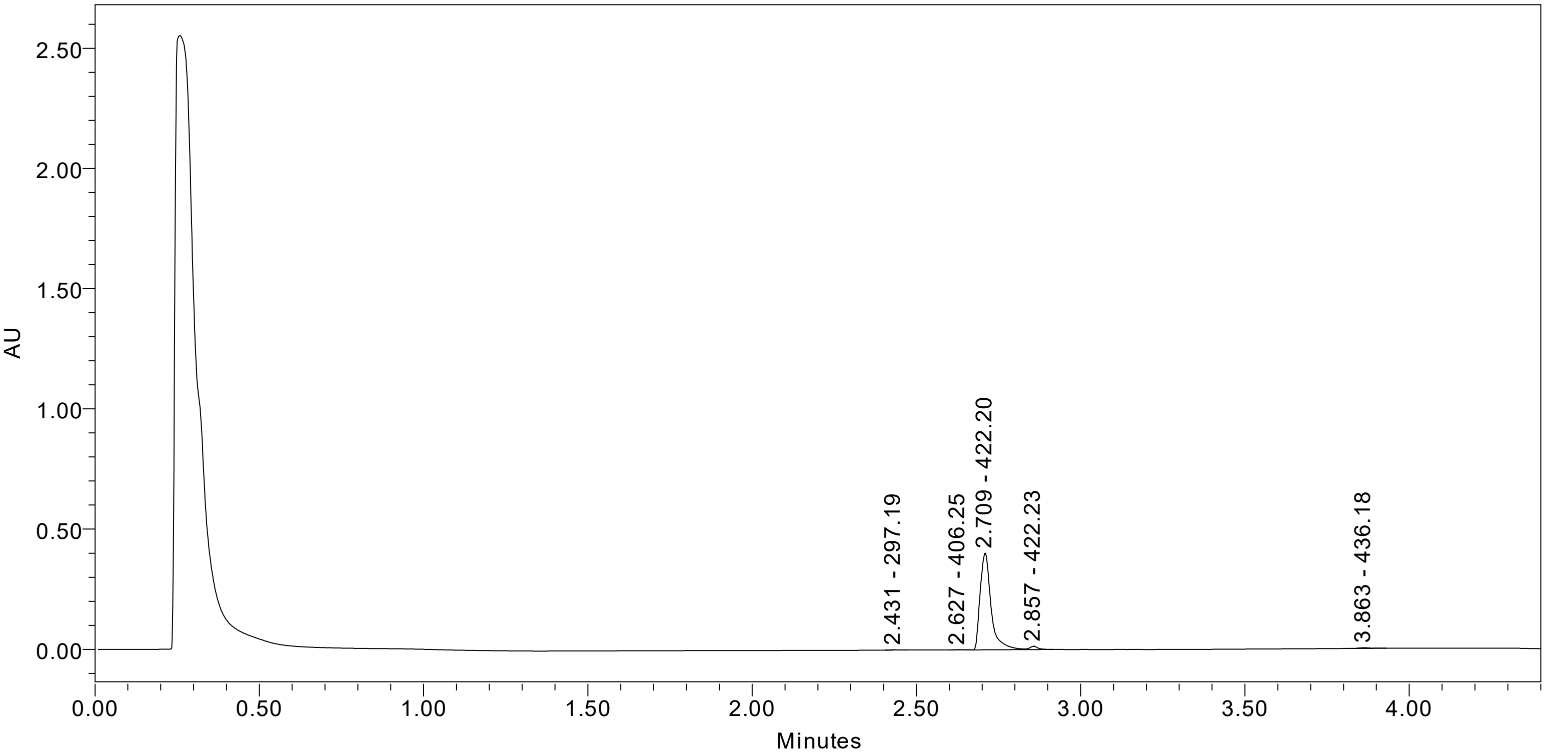

	RT	Area	% Area	Height	Base Peak (m/z)
1	2.431	1790	0.19	922	297.19
2	2.627	1688	0.17	863	406.25
3	2.709	933102	96.71	402628	422.20
4	2.857	23175	2.40	13618	422.23
5	3.863	5065	0.52	2205	436.18

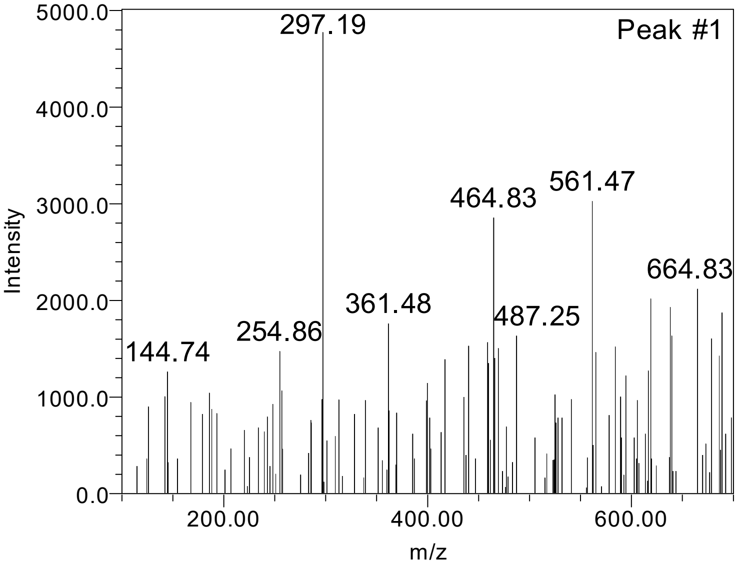

RT: 2.431

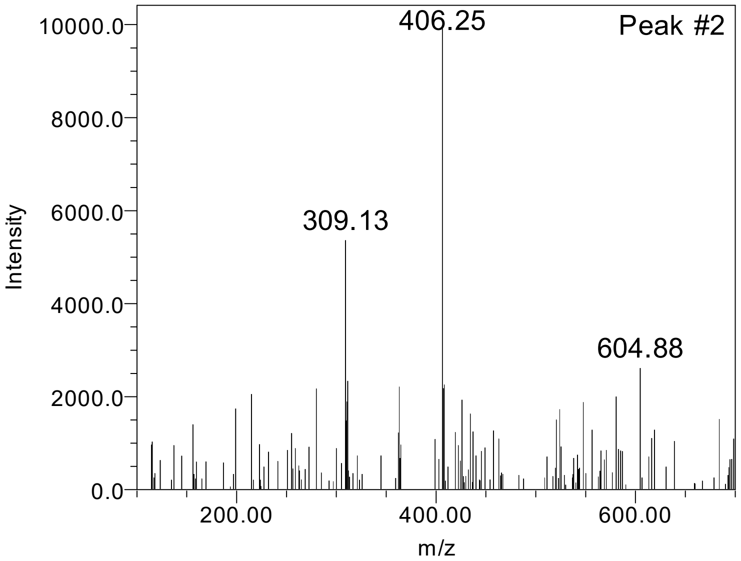

RT: 2.627

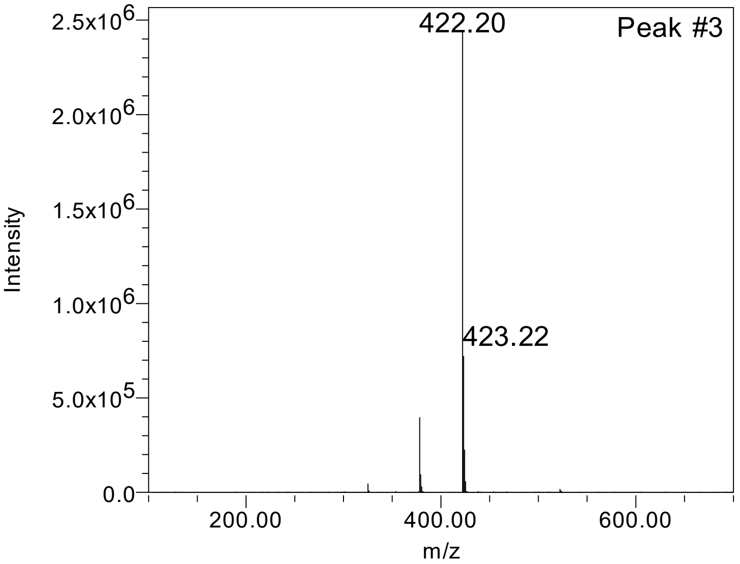

RT: 2.709

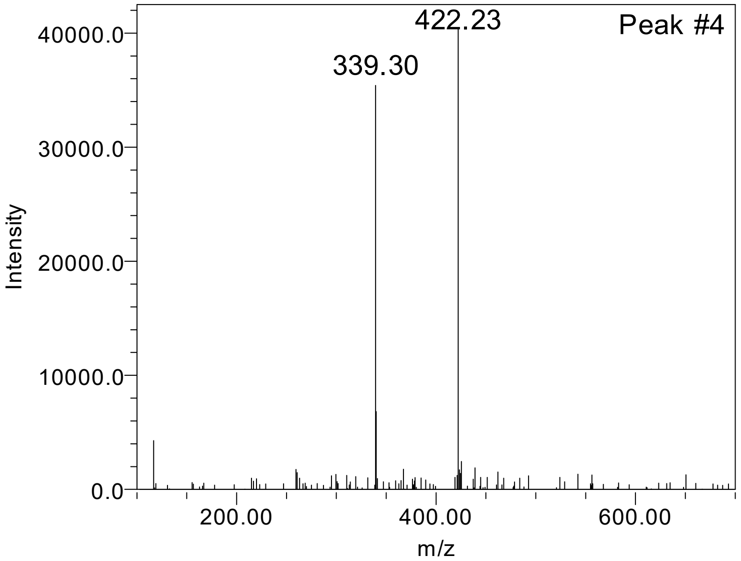

RT: 2.857

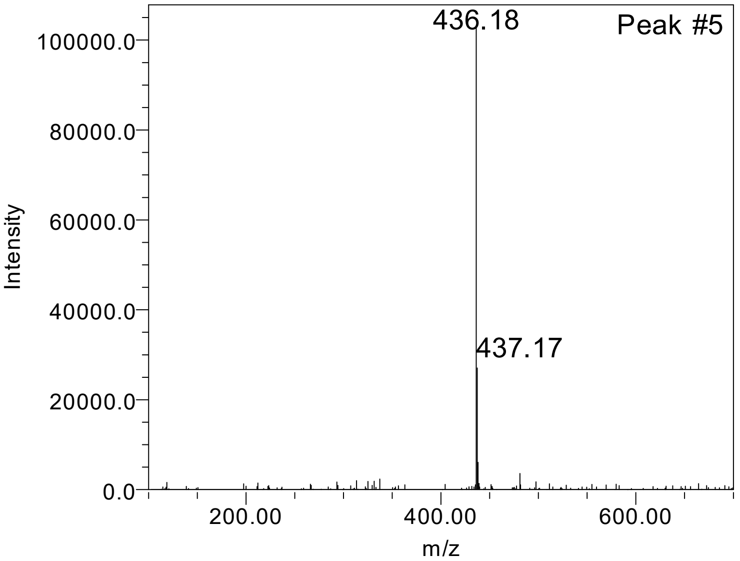

RT: 3.863

Mass Analysis Report

SAMPLE INFORMATION

Sample Name: SR210708A
Acq Method Set: Col1_MeOH_H2O_NH4HCO3
Acquired: 7/16/2021 8:26:26 PM CDT
InjVol: 7.50 uL

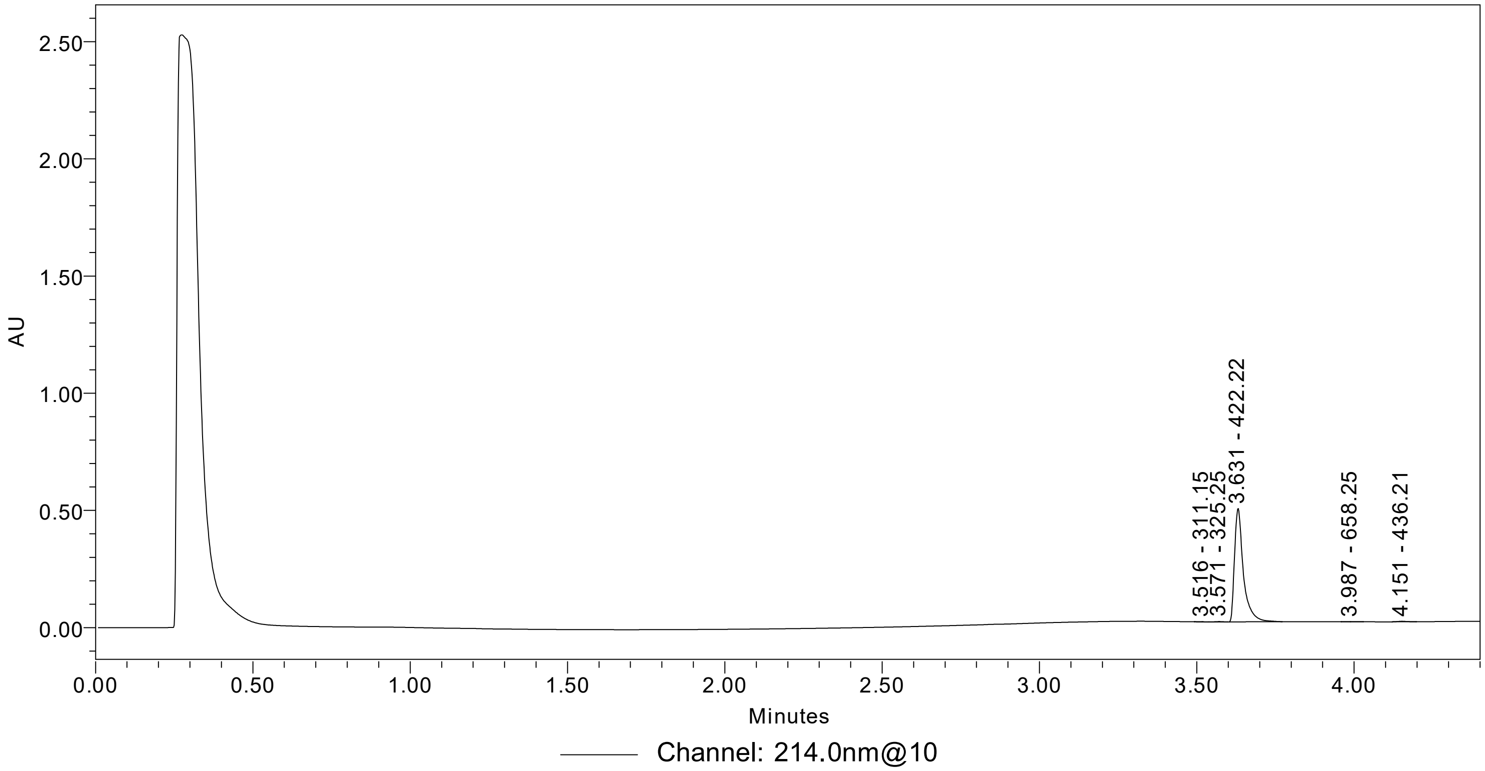

	RT	Area	% Area	Height	Base Peak (m/z)
1	3.516	473	0.05	396	311.15
2	3.571	2394	0.25	1465	325.25
3	3.631	948275	99.22	483193	422.22
4	3.987	953	0.10	518	658.25
5	4.151	3630	0.38	1847	436.21

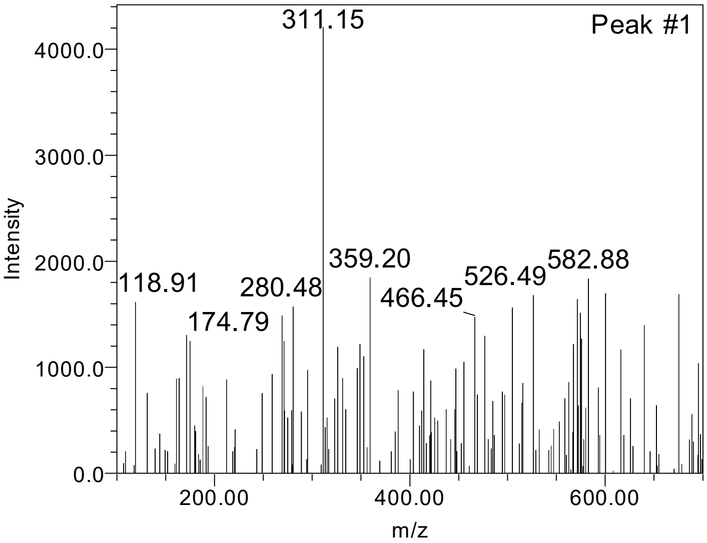

RT: 3.516

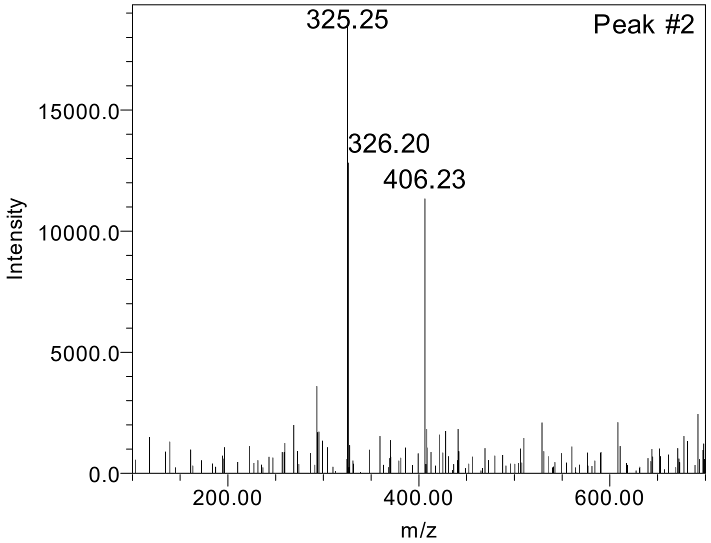

RT: 3.571

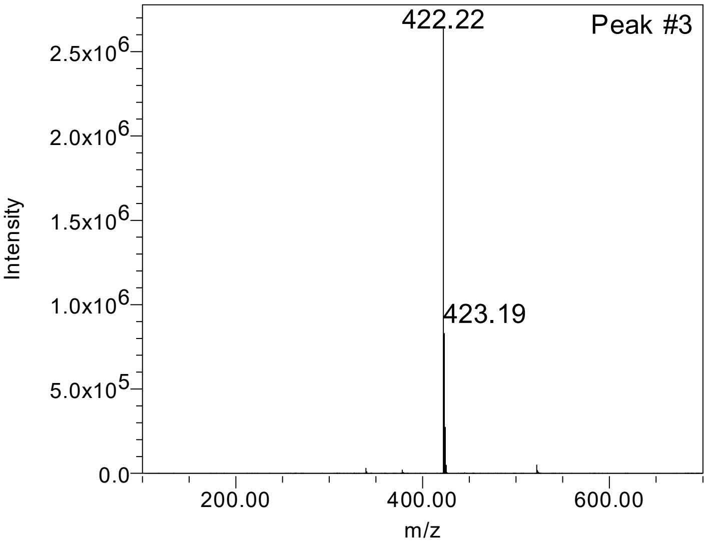

RT: 3.631

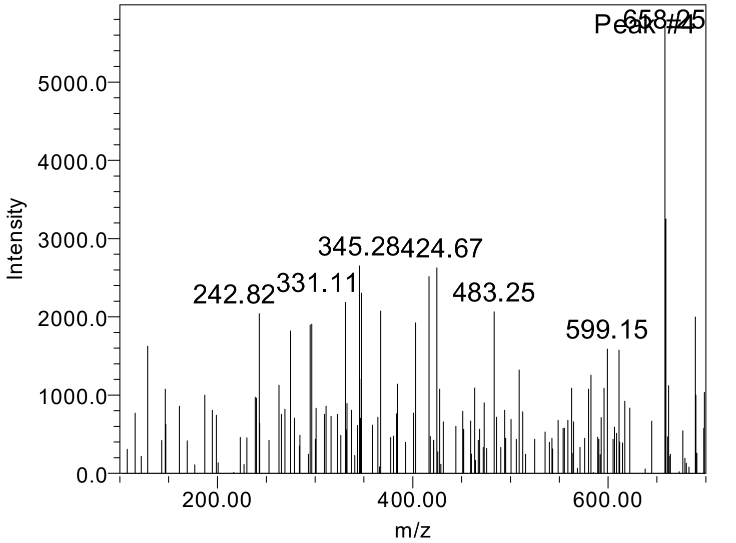

RT: 3.987

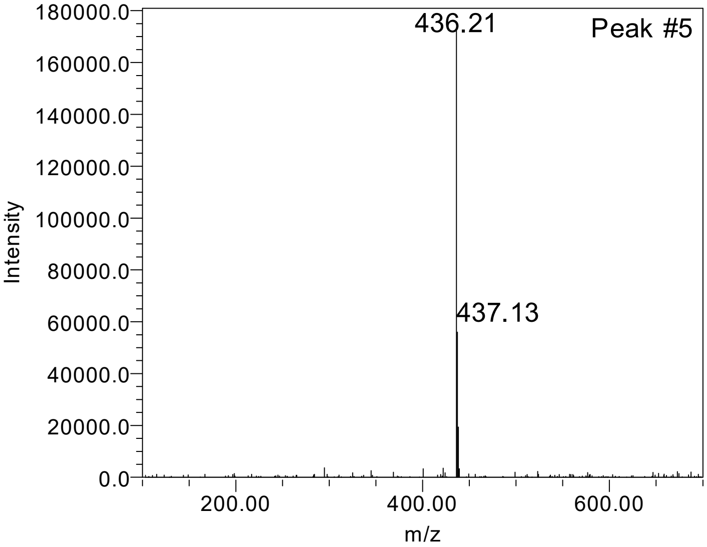

RT: 4.151

Single Injection Report

**Sample name:**	SR220826A		
**Description:**			
**Sample amount:**	0.000	**Sample type:**	Sample
**Instrument:**	LCMS	**Location:**	P1-A9
		**Injection:**	1 of 1
**Acq. method:**	Regular method.amx	**Injection volume:**	5.000 µL
**Analysis method:**	MS method-purity.pmx	**Acq. operator:**	SYSTEM

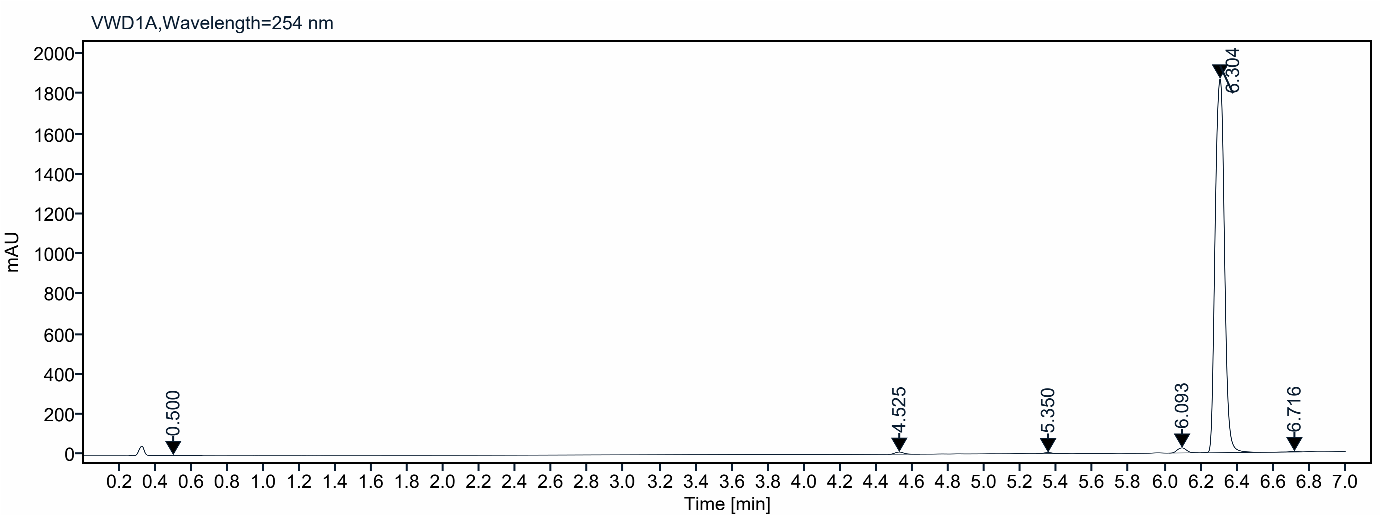

Signal: MS1 +TIC SCAN ESI Frag=110V Gain=1.0

RT [min]	Width [min]	Area	Height	Area%
6.370	0.3094	1807975.04 71	232282.2209	100.0000
	**Sum**	**1807975.04 71**		

Signal: VWD1A,Wavelength=254 nm

RT [min]	Width [min]	Area	Height	Area%
0.500	0.2961	28.6904	1.7843	0.4189
4.525	0.1788	35.0369	10.6949	0.5115
5.350	0.1384	18.5165	5.0825	0.2703
6.093	0.1647	88.9525	25.1259	1.2987
6.304	0.3273	6660.7877	1863.8316	97.2480
6.716	0.2340	17.2935	3.2605	0.2525
	**Sum**	**6849.2774**		

Single Injection Report

6.37 - 6.374 (2025-06-23 05-44-31-05-00-08.dx)

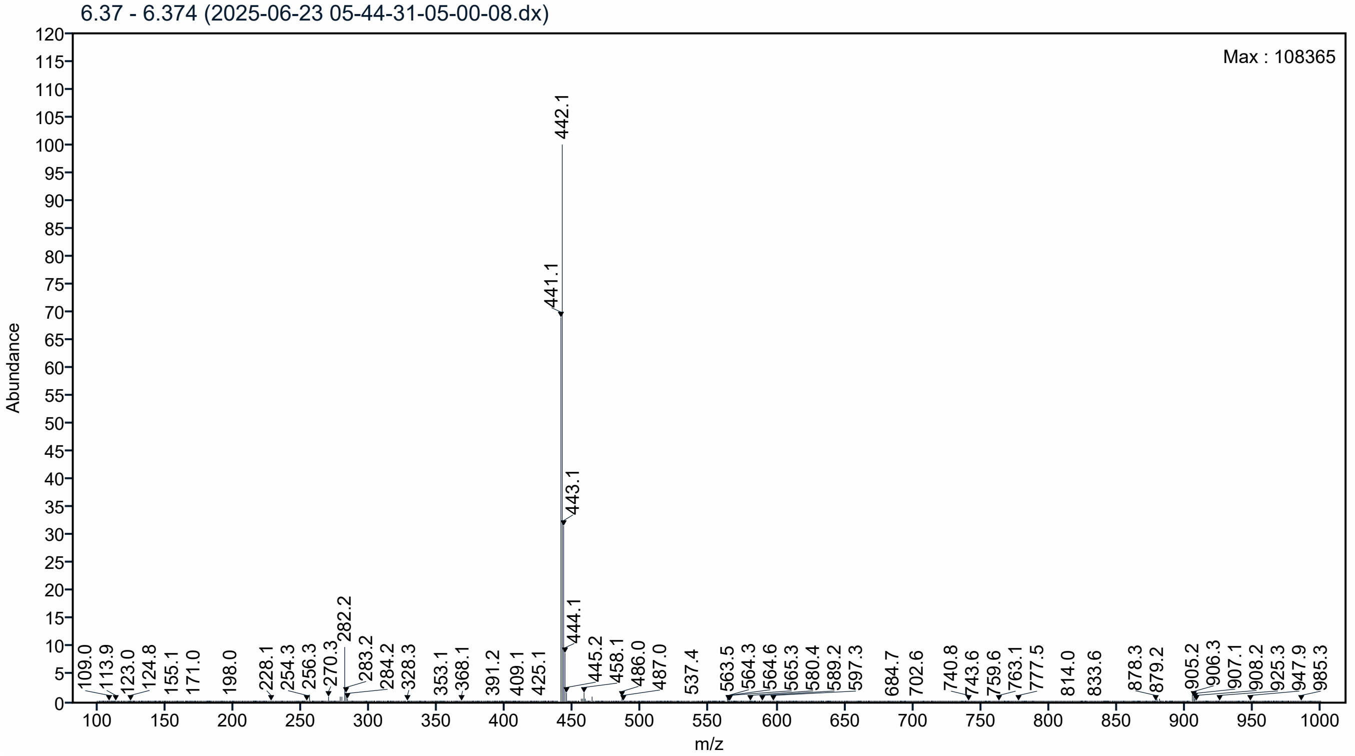

Single Injection Report

**Sample name:**	SR220826B		
**Description:**			
**Sample amount:**	0.000	**Sample type:**	Sample
**Instrument:**	LCMS	**Location:**	P1-A10
		**Injection:**	1 of 1
**Acq. method:**	Regular method.amx	**Injection volume:**	5.000 µL
**Analysis method:**	MS method-purity.pmx	**Acq. operator:**	SYSTEM

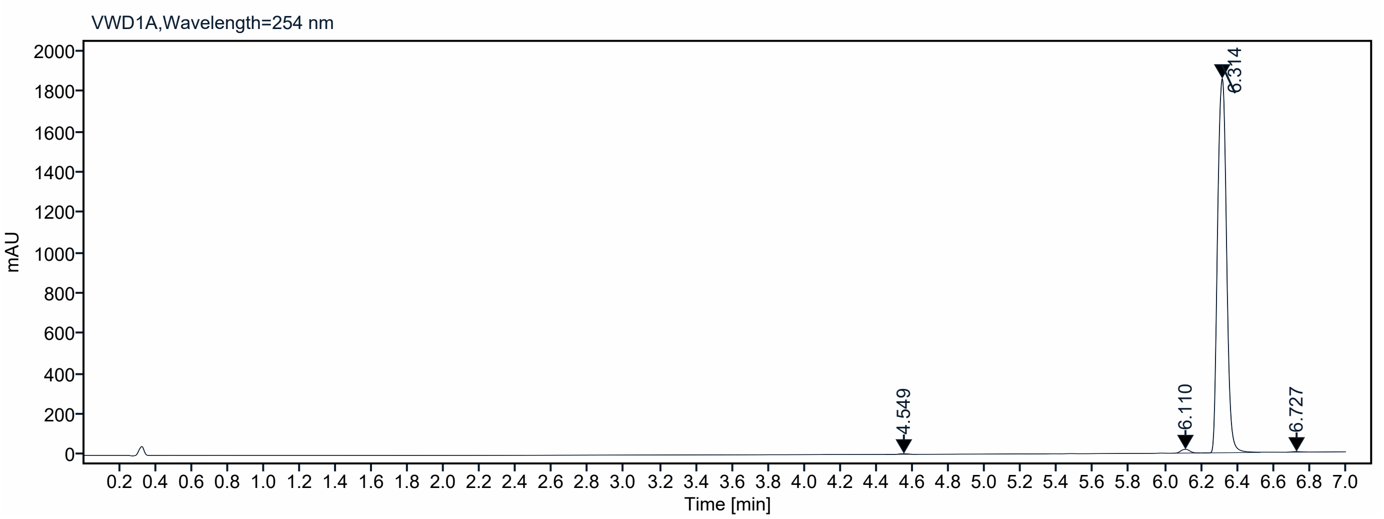

Signal: MS1 +TIC SCAN ESI Frag=110V Gain=1.0

RT [min]	Width [min]	Area	Height	Area%
6.378	0.3284	1564218.4903	195617.3957	100.0000
	**Sum**	**1564218.4903**		

Signal: VWD1A,Wavelength=254 nm

RT [min]	Width [min]	Area	Height	Area%
4.549	0.1769	10.7288	3.3151	0.1653
6.110	0.1510	64.1215	18.8523	0.9880
6.314	0.3191	6407.1884	1853.2975	98.7219
6.727	0.1166	8.1028	2.6349	0.1248
	**Sum**	**6490.1416**		

Single Injection Report

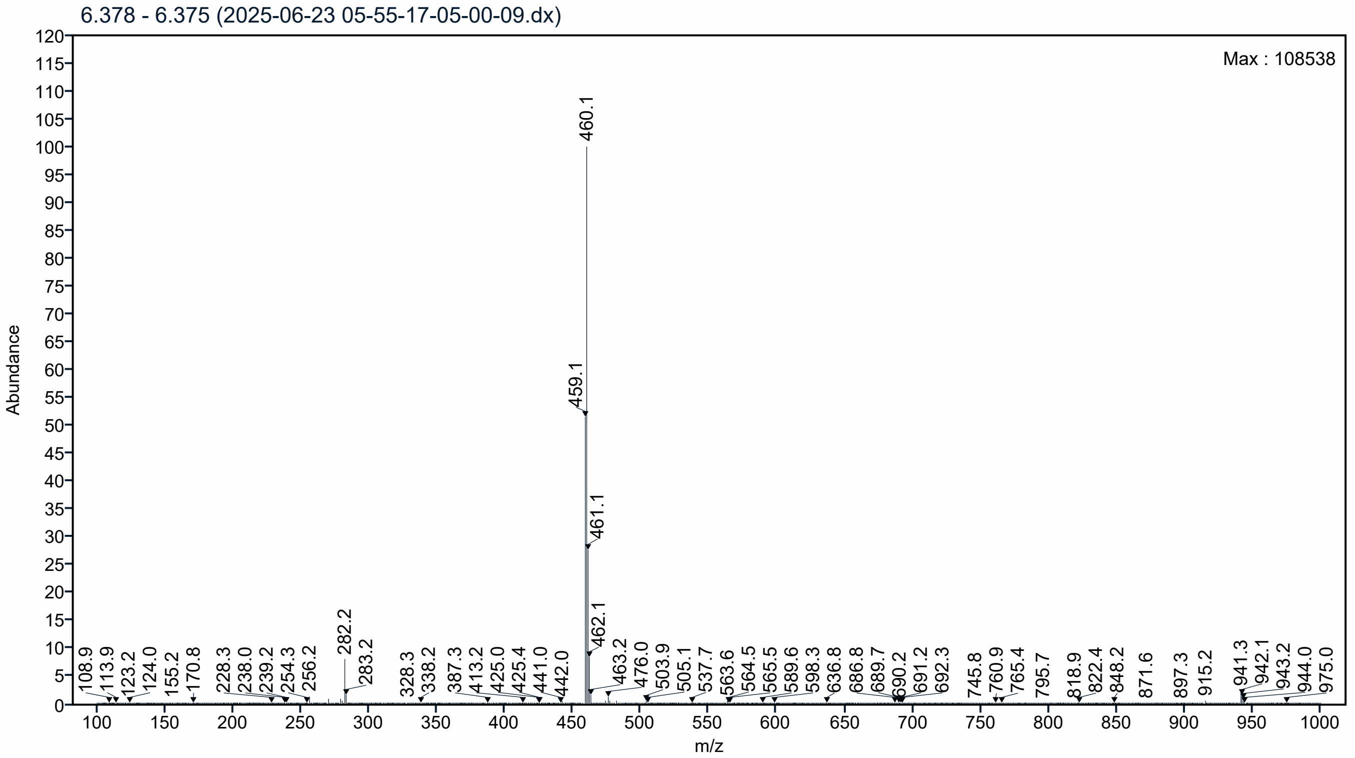

Mass Analysis Report

SAMPLE INFORMATION

Sample Name: SR211003A
Acq Method Set: Col2_MeCN_H2O_NH4HCO3
Acquired: 10/14/2021 6:25:53 PM CDT
InjVol: 7.50 uL

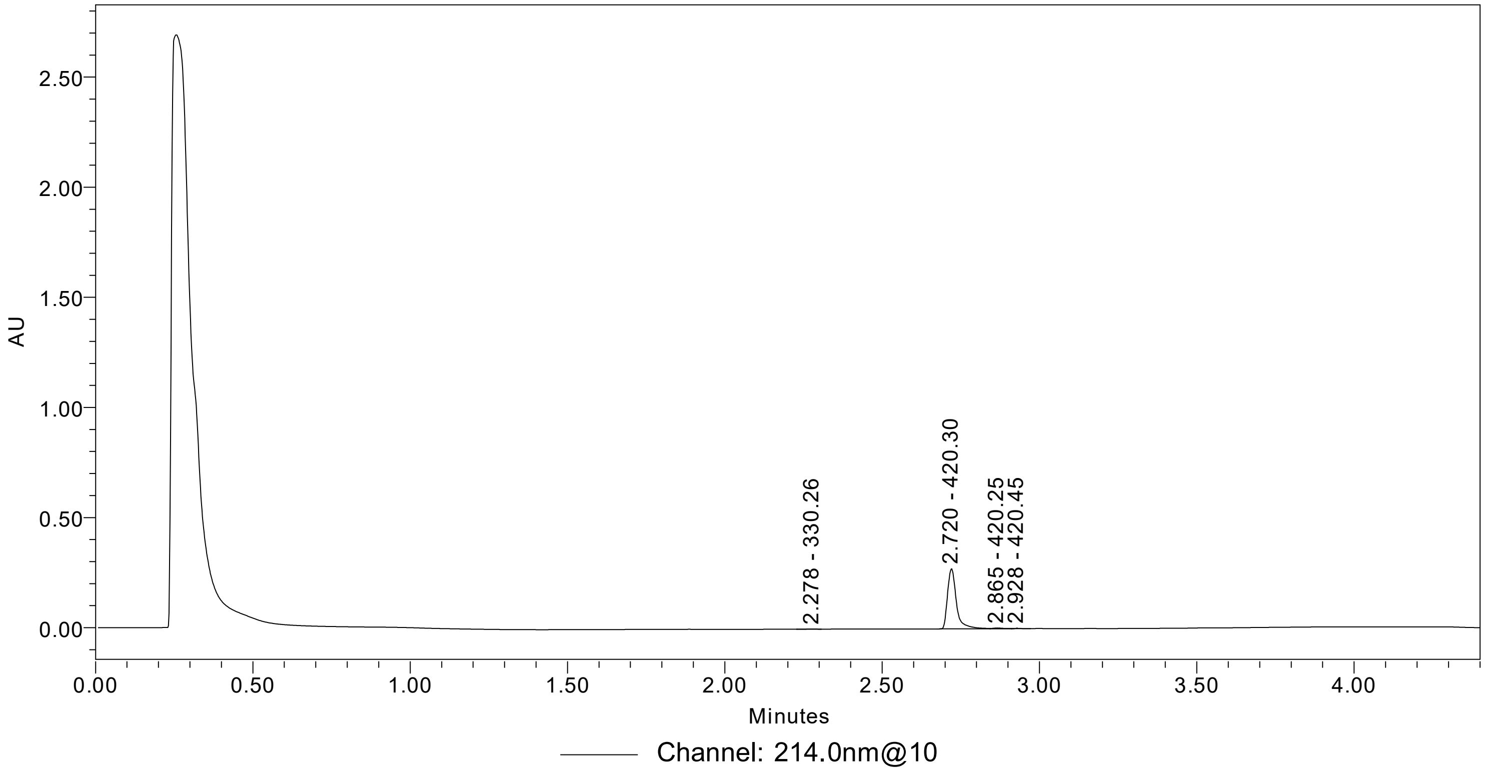

	RT	Area	% Area	Height	Base Peak (m/z)
1	2.278	1995	0.36	1032	330.26
2	2.720	536295	97.39	272270	420.30
3	2.865	8837	1.60	3448	420.25
4	2.928	3521	0.64	1780	420.45

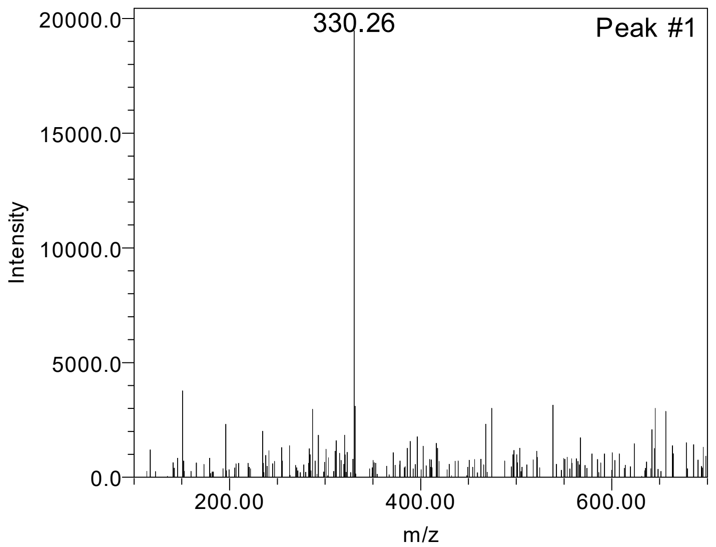

RT: 2.278

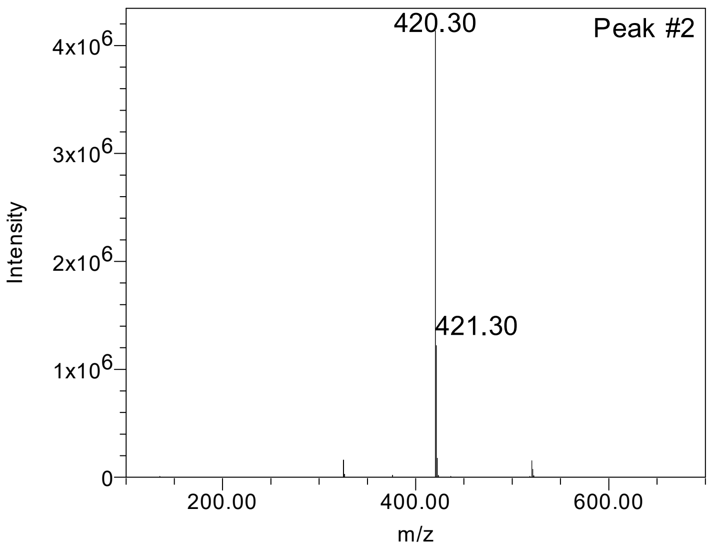

RT: 2.720

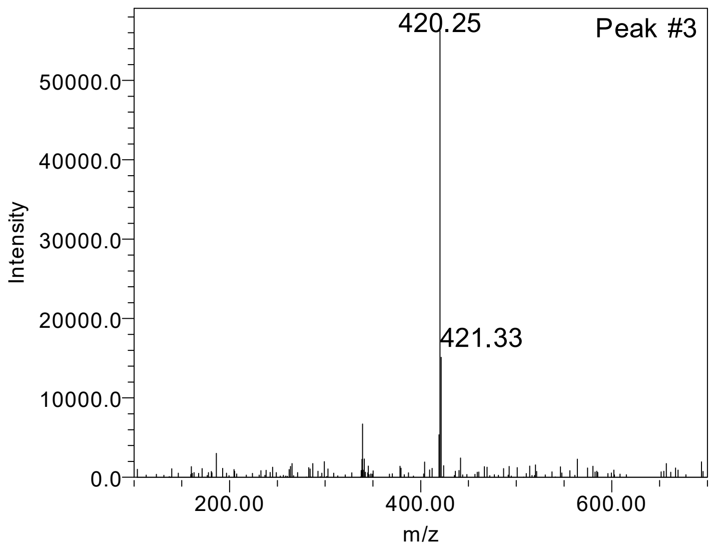

RT: 2.865

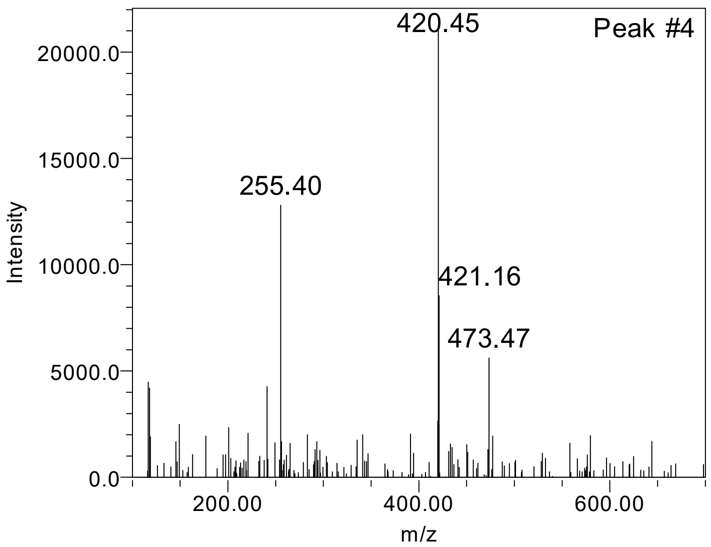

RT: 2.928

Mass Analysis Report

SAMPLE INFORMATION

Sample Name: SR211003A
Acq Method Set: Col1_MeOH_H2O_NH4HCO3
Acquired: 10/14/2021 10:38:29 PM CDT
InjVol: 7.50 uL

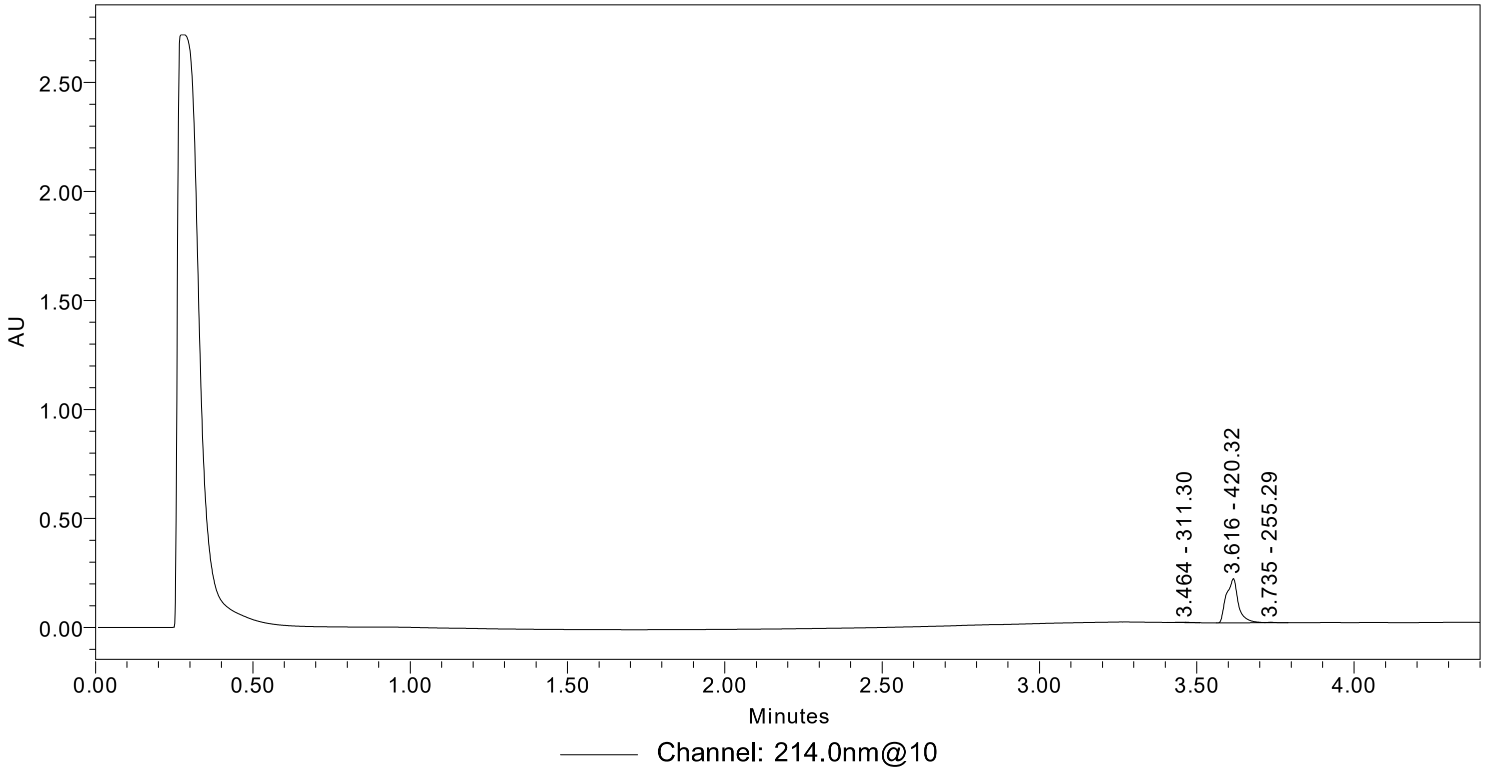

	RT	Area	% Area	Height	Base Peak (m/z)
1	3.464	3965	0.73	1238	311.30
2	3.616	532146	98.55	202609	420.32
3	3.735	3892	0.72	2097	255.29

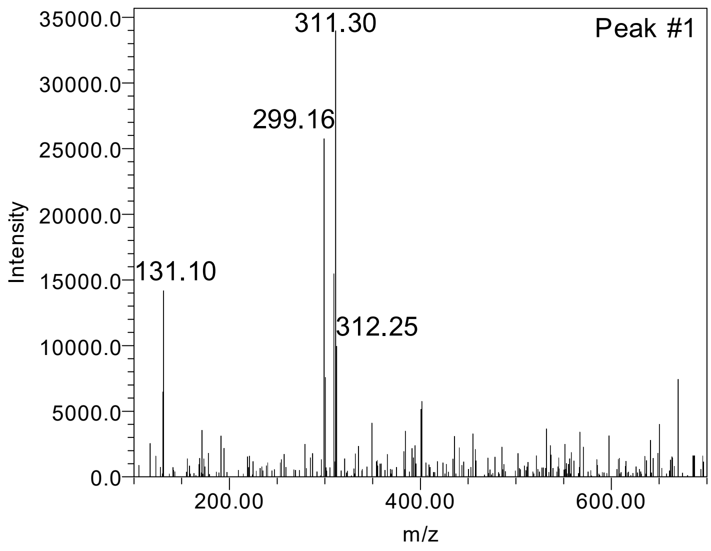

RT: 3.464

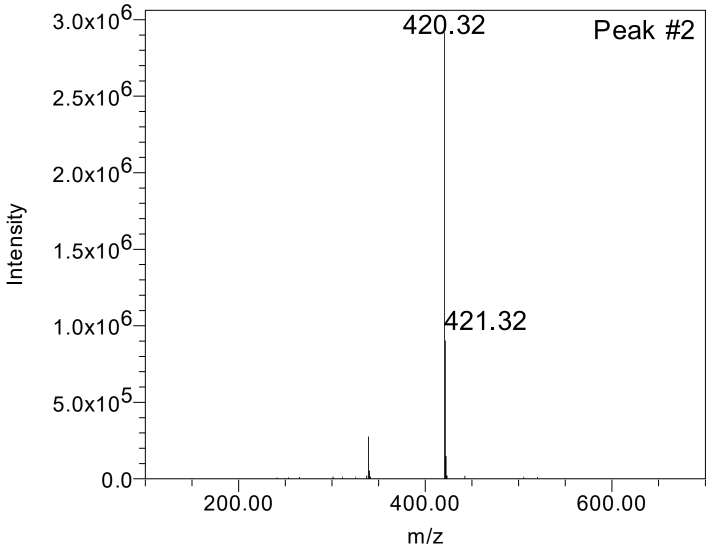

RT: 3.616

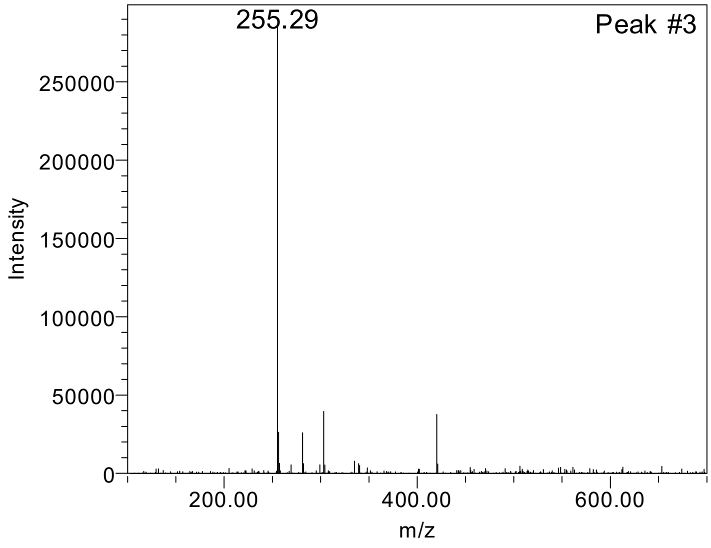

RT: 3.735

Mass Analysis Report

SAMPLE INFORMATION

Sample Name: SR210401A
Acq Method Set: Col2_MeCN_H2O_NH4HCO3
Acquired: 5/14/2021 6:20:40 PM CDT
InjVol: 7.50 uL

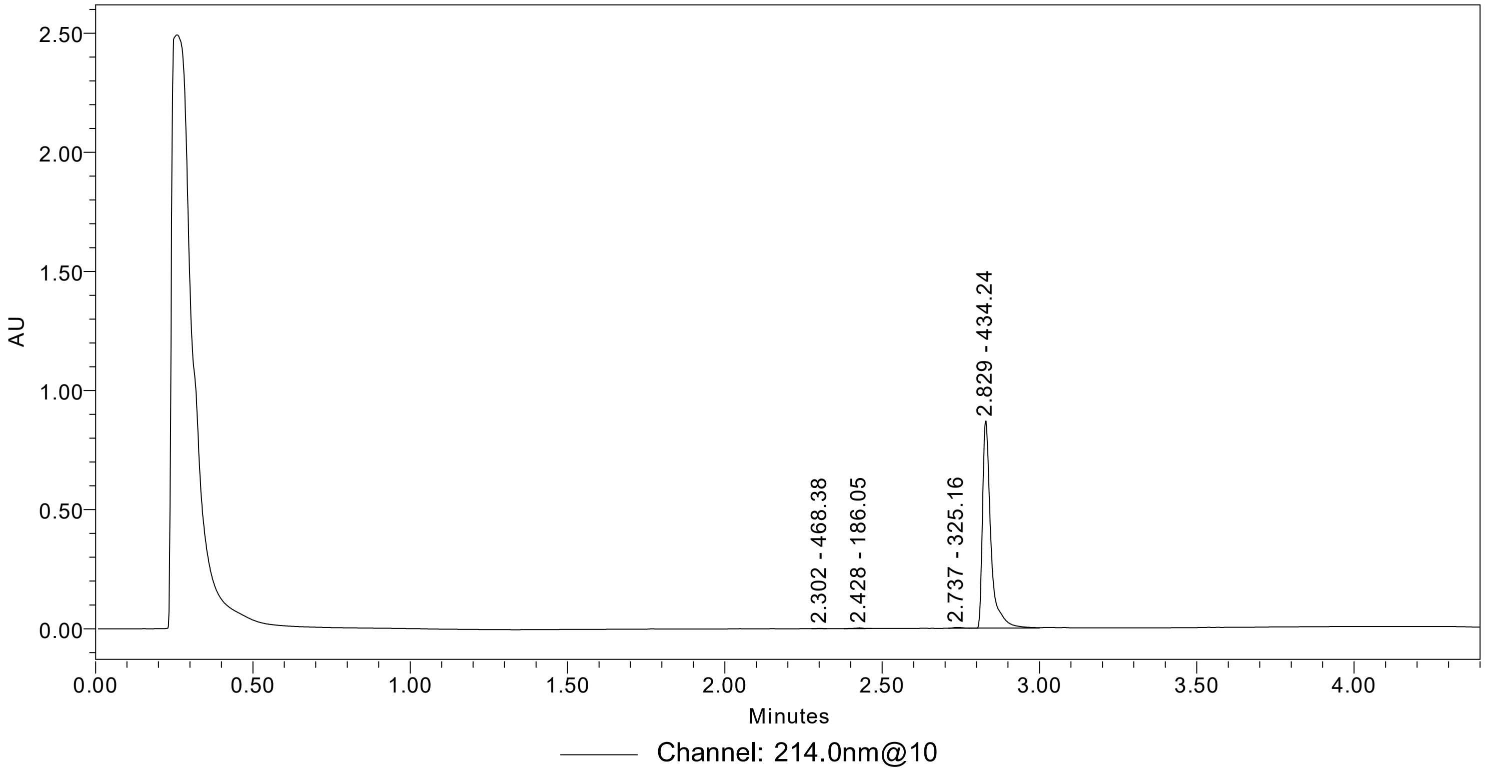

	RT	Area	% Area	Height	Base Peak (m/z)
1	2.302	1312	0.08	1168	468.38
2	2.428	4416	0.27	2577	186.05
3	2.737	6704	0.41	3303	325.16
4	2.829	1609979	99.23	871536	434.24

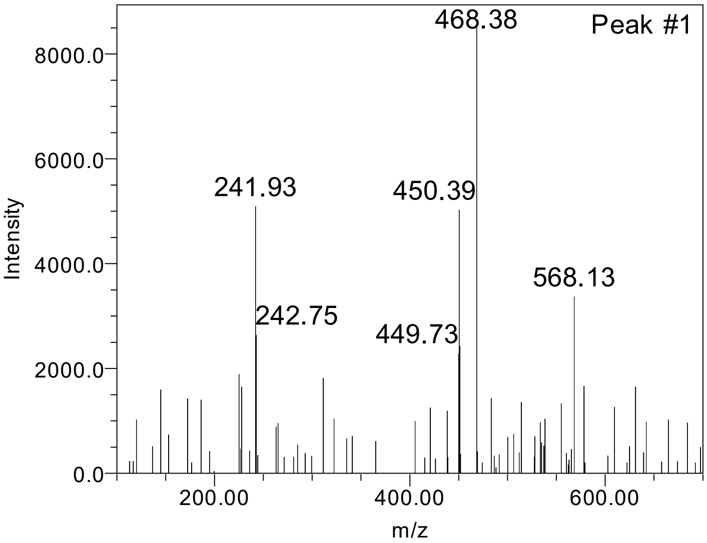

RT: 2.302

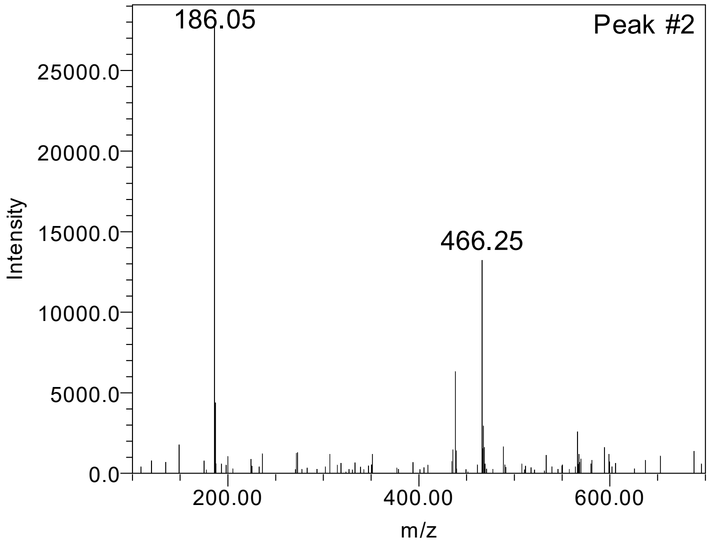

RT: 2.428

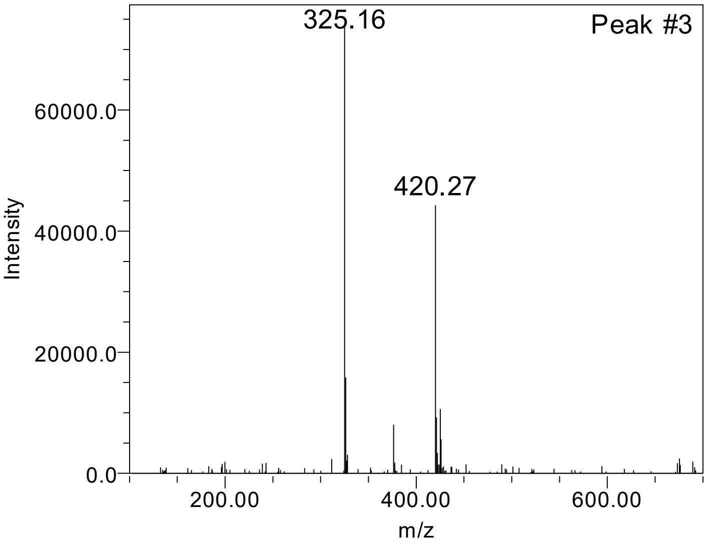

RT: 2.737

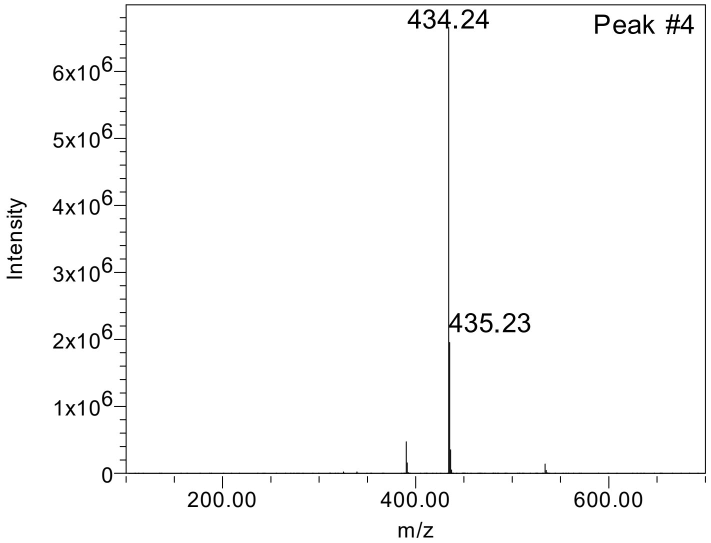

RT: 2.829

Mass Analysis Report

SAMPLE INFORMATION

Sample Name: SR210401A
Acq Method Set: Col1_MeOH_H2O_NH4HCO3
Acquired: 5/15/2021 1:01:12 AM CDT
InjVol: 7.50 uL

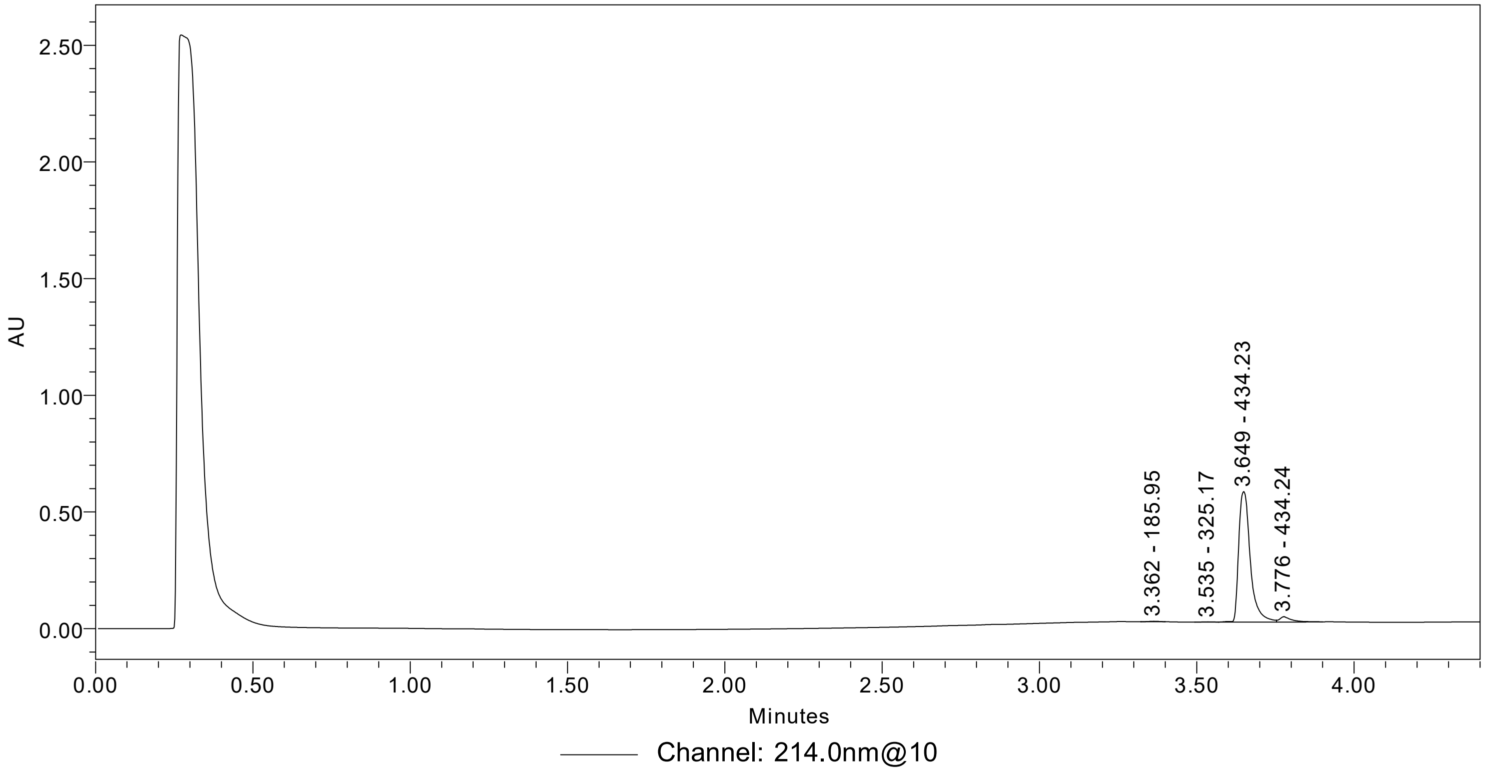

	RT	Area	% Area	Height	Base Peak (m/z)
1	3.362	4139	0.28	1868	185.95
2	3.535	2063	0.14	713	325.17
3	3.649	1434092	95.53	558548	434.23
4	3.776	60905	4.06	22739	434.24

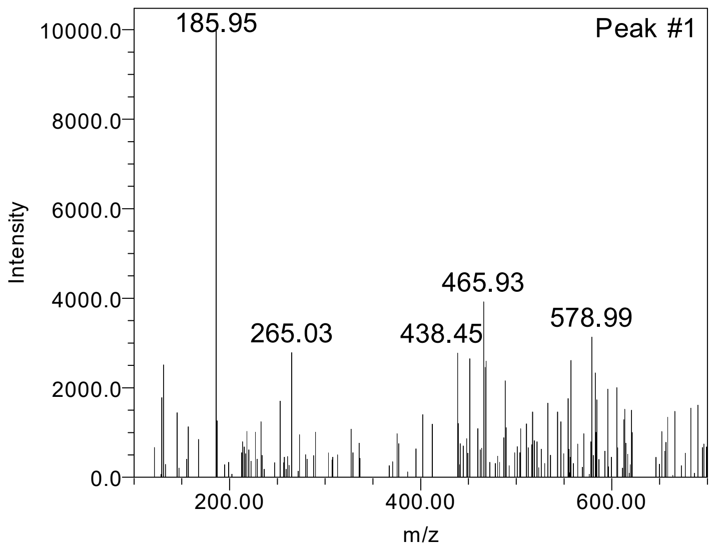

RT: 3.362

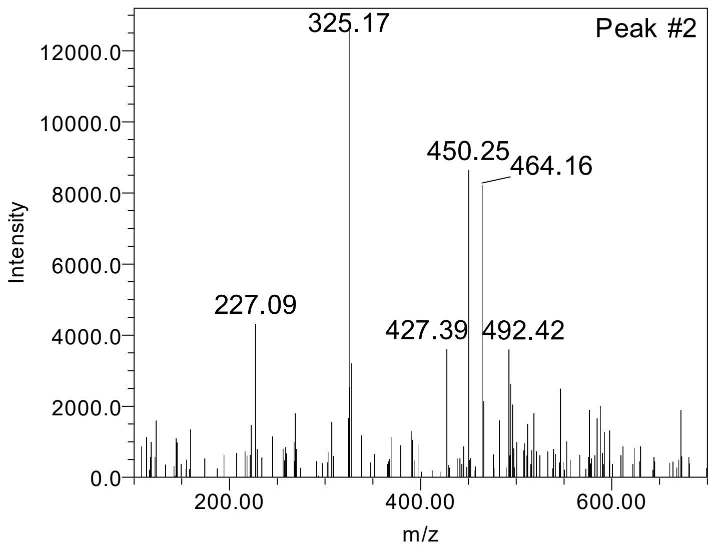

RT: 3.535

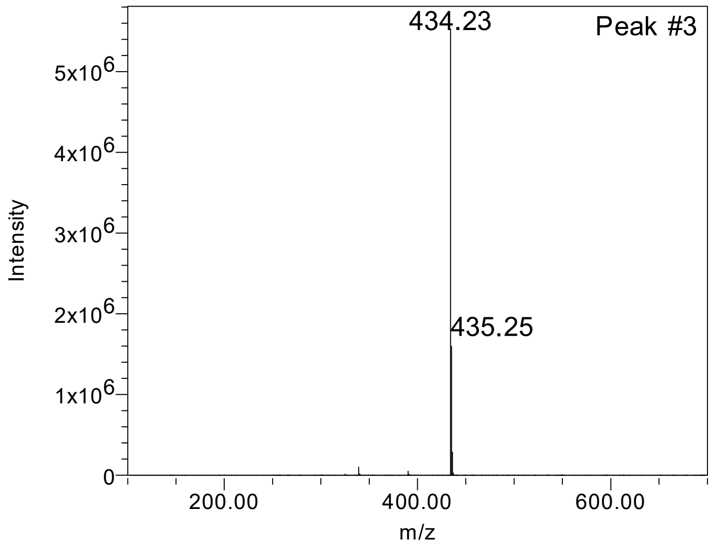

RT: 3.649

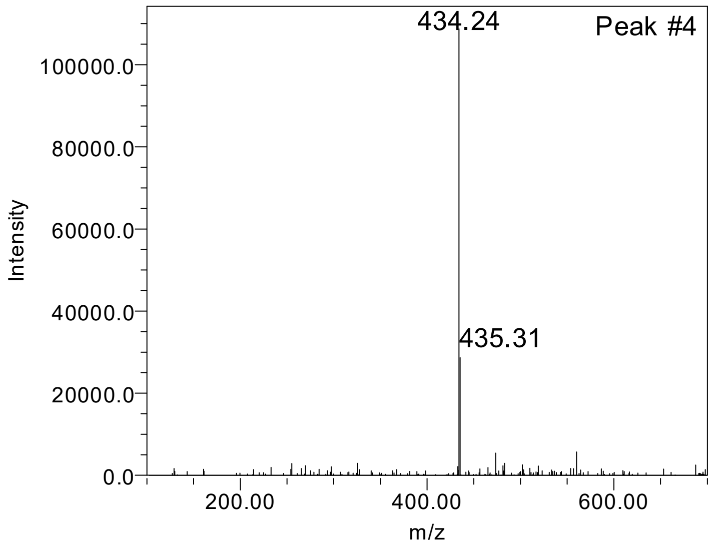

RT: 3.776

Single Injection Report

**Sample name:**	SR220908A		
**Description:**			
**Sample amount:**	0.000	**Sample type:**	Sample
**Instrument:**	LCMS	**Location:**	P1-A11
		**Injection:**	1 of 1
**Acq. method:**	Regular method.amx	**Injection volume:**	5.000 µL
**Analysis method:**	MS method-purity.pmx	**Acq. operator:**	SYSTEM

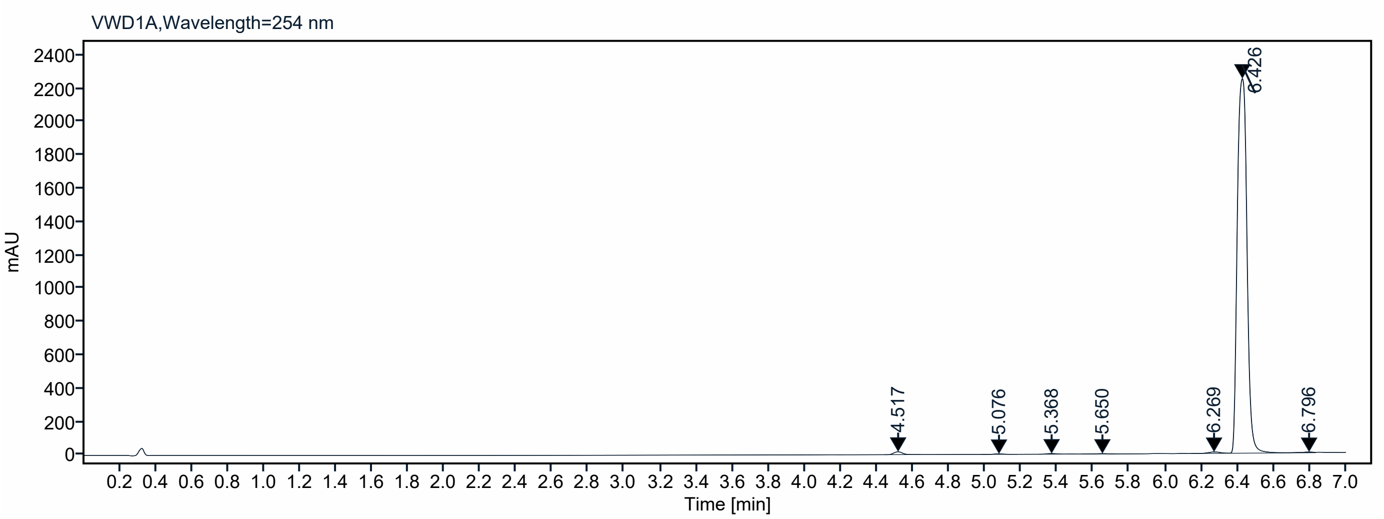

Signal: MS1 +TIC SCAN ESI Frag=110V Gain=1.0

RT [min]	Width [min]	Area	Height	Area%
6.460	0.0641	626258.9087	250227.0298	100.0000
	**Sum**	**626258.9087**		

Signal: VWD1A,Wavelength=254 nm

RT [min]	Width [min]	Area	Height	Area%
4.517	0.2008	56.6337	17.0737	0.6666
5.076	0.1977	10.1441	2.7287	0.1194
5.368	0.1299	13.1763	3.6142	0.1551
5.650	0.1914	9.0442	2.0490	0.1065
6.269	0.2125	39.6587	8.3527	0.4668
6.426	0.2989	8340.4316	2245.8686	98.1677
6.796	0.1818	27.0172	4.4072	0.3180
	**Sum**	**8496.1057**		

Single Injection Report

6.46 - 6.459 (2025-06-23 06-06-03-05-00-10.dx)

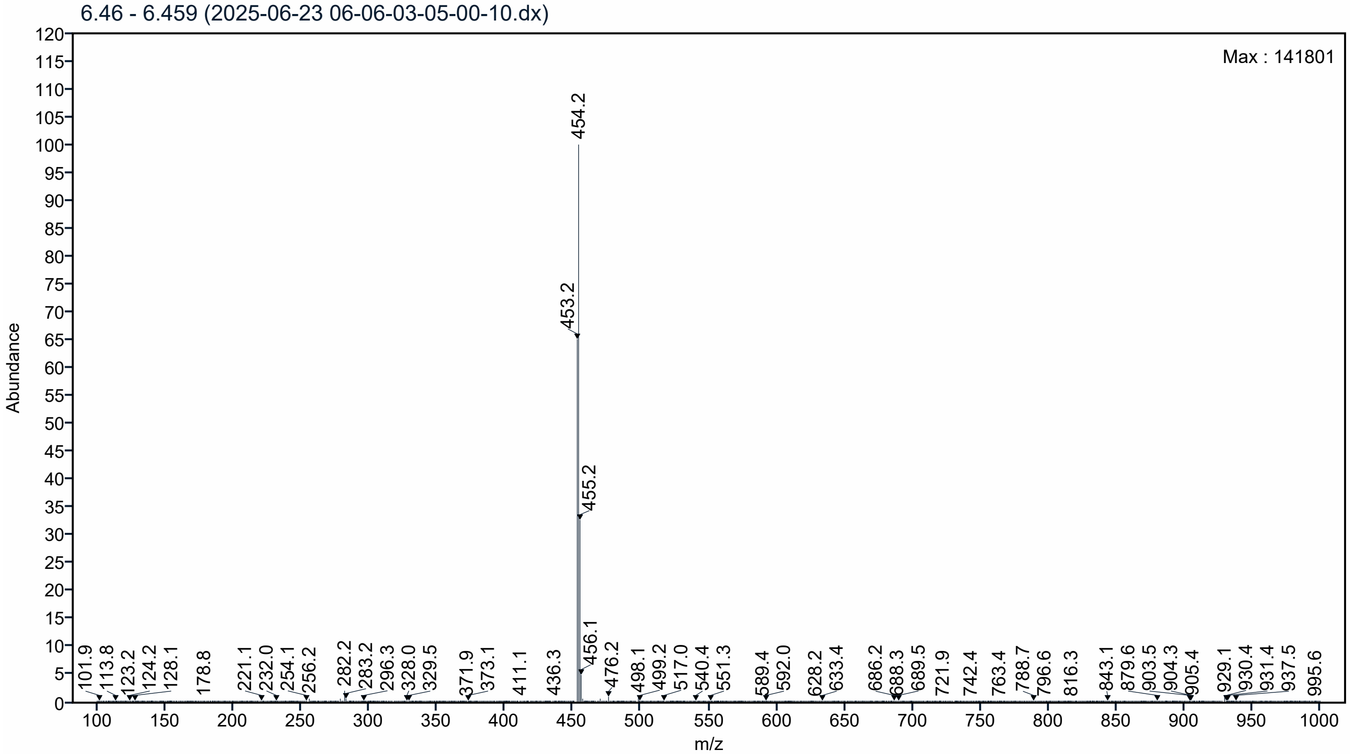

Single Injection Report

**Sample name:**	SR220908B		
**Description:**			
**Sample amount:**	0.000	**Sample type:**	Sample
**Instrument:**	LCMS	**Location:**	P1-B1
		**Injection:**	1 of 1
**Acq. method:**	Regular method.amx	**Injection volume:**	5.000 µL
**Analysis method:**	MS method-purity.pmx	**Acq. operator:**	SYSTEM

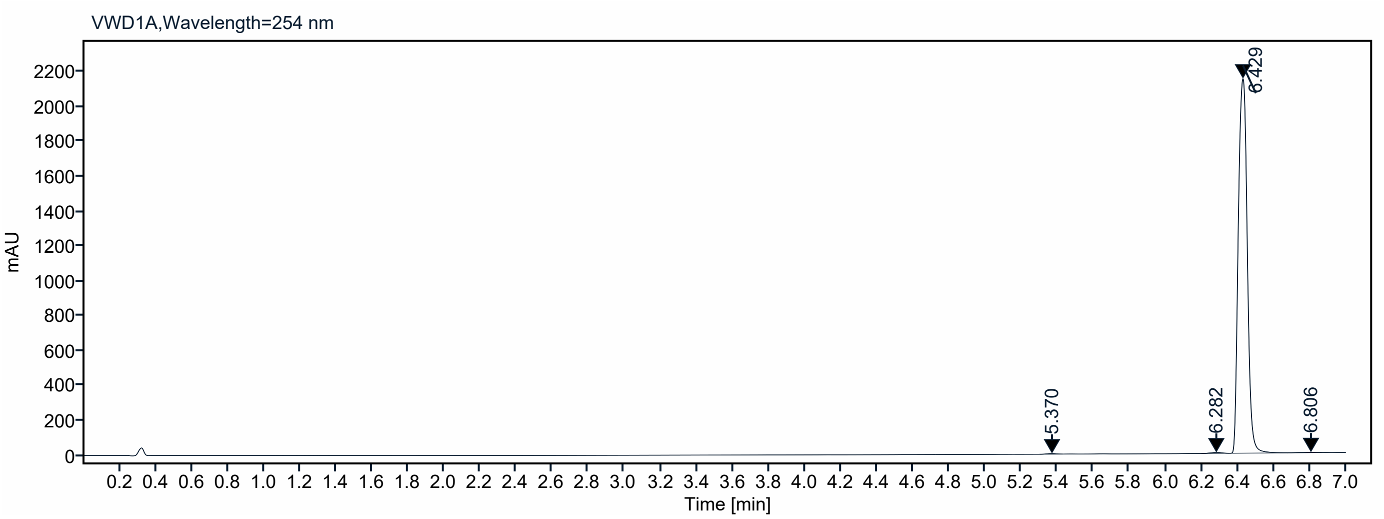

Signal: MS1 +TIC SCAN ESI Frag=110V Gain=1.0

RT [min]	Width [min]	Area	Height	Area%
6.467	0.2275	1546272.7920	241953.9556	100.0000
	**Sum**	**1546272.7920**		

Signal: VWD1A,Wavelength=254 nm

RT [min]	Width [min]	Area	Height	Area%
5.370	0.1272	13.6298	3.7590	0.1827
6.282	0.1573	22.0527	5.1019	0.2956
6.429	0.3192	7410.4632	2149.2801	99.3171
6.806	0.1882	15.2710	2.5750	0.2047
	**Sum**	**7461.4167**		

Single Injection Report

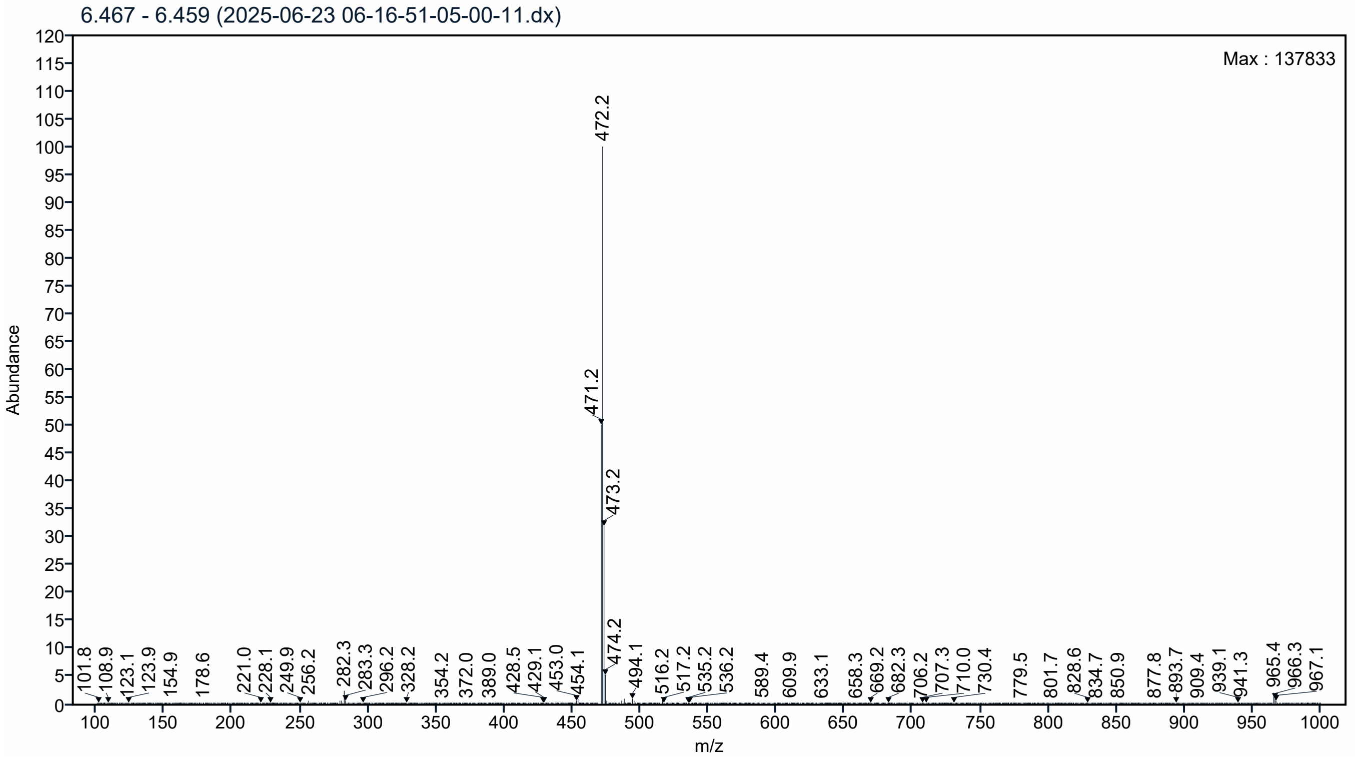

Single Injection Report

**Sample name:**	SR230329D		
**Description:**			
**Sample amount:**	0.000	**Sample type:**	Sample
**Instrument:**	LCMS	**Location:**	P1-B2
		**Injection:**	1 of 1
**Acq. method:**	Regular method.amx	**Injection volume:**	5.000 µL
**Analysis method:**	MS method-purity.pmx	**Acq. operator:**	SYSTEM

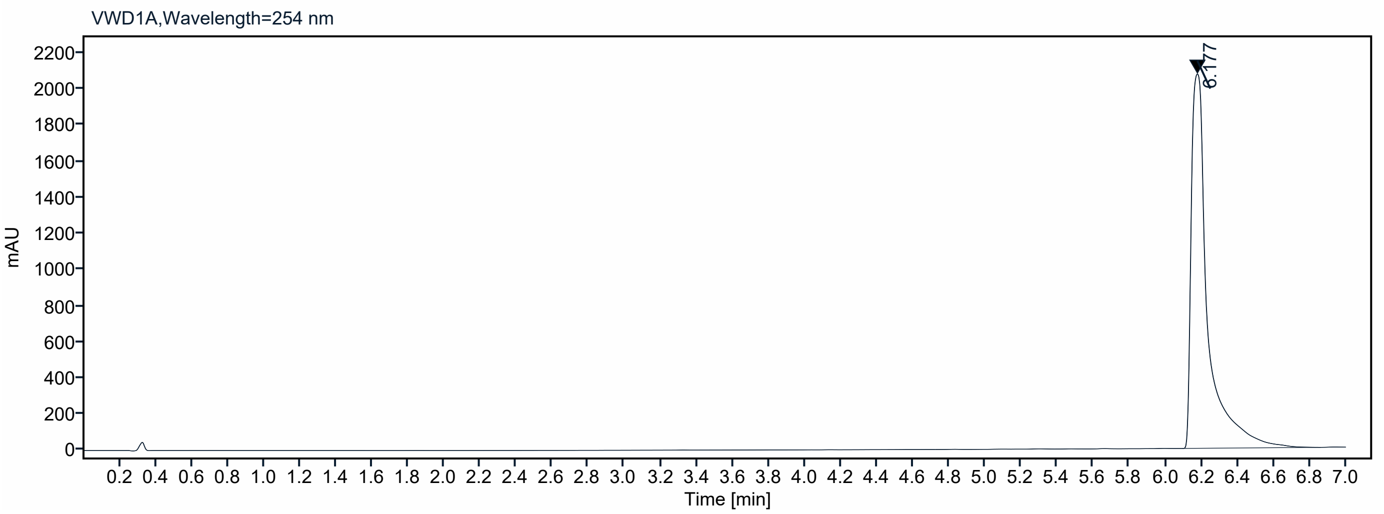

Signal: MS1 +TIC SCAN ESI Frag=110V Gain=1.0

RT [min]	Width [min]	Area	Height	Area%
6.234	0.5303	4085049.9294	308198.3338	100.0000
	**Sum**	**4085049.9294**		

Signal: VWD1A,Wavelength=254 nm

RT [min]	Width [min]	Area	Height	Area%
6.177	0.7658	13239.7178	2072.1568	100.0000
	**Sum**	**13239.7178**		

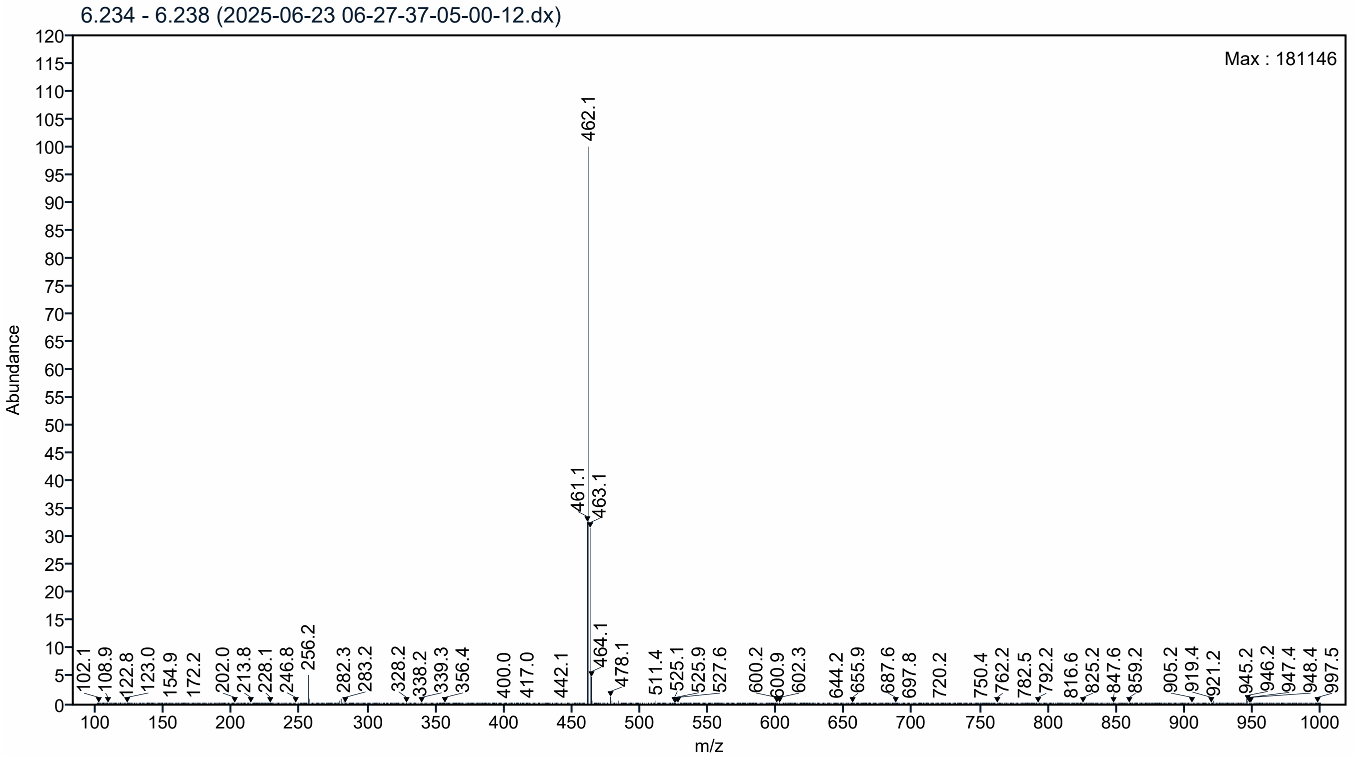
6.234 - 6.238 (2025-06-23 06-27-37-05-00-12.dx)
Max : 181146
Abundance
m/z
462.1
461.1
463.1
256.2
464.1
478.1

Single Injection Report

**Sample name:**	SR230329E		
**Description:**			
**Sample amount:**	0.000	**Sample type:**	Sample
**Instrument:**	LCMS	**Location:**	P1-B3
		**Injection:**	1 of 1
**Acq. method:**	Regular method.amx	**Injection volume:**	5.000 µL
**Analysis method:**	MS method-purity.pmx	**Acq. operator:**	SYSTEM

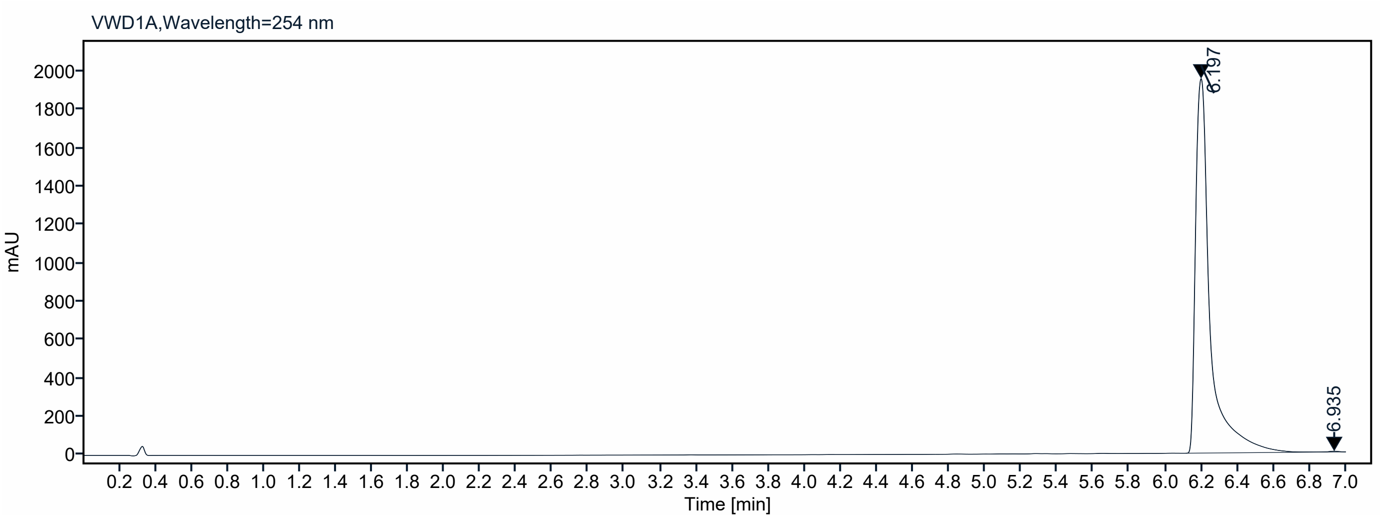

Signal: MS1 +TIC SCAN ESI Frag=110V Gain=1.0

RT [min]	Width [min]	Area	Height	Area%
6.254	0.5380	3984779.4481	376816.0057	100.0000
	**Sum**	**3984779.4481**		

Signal: VWD1A,Wavelength=254 nm

RT [min]	Width [min]	Area	Height	Area%
6.197	0.7246	10894.4371	1952.2007	99.8620
6.935	0.1627	15.0528	3.9925	0.1380
	**Sum**	**10909.4899**		

Single Injection Report

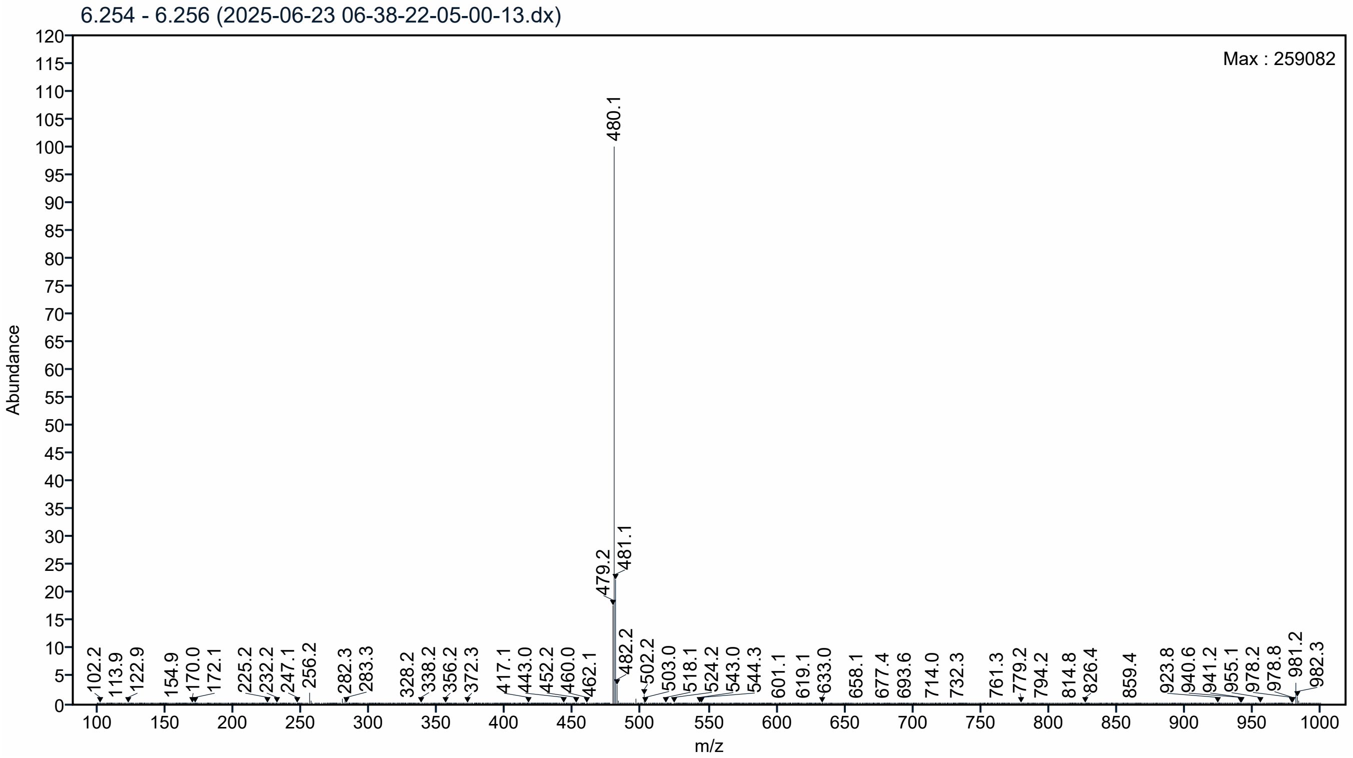

Single Injection Report

**Sample name:**	SR230329F		
**Description:**			
**Sample amount:**	0.000	**Sample type:**	Sample
**Instrument:**	LCMS	**Location:**	P1-B4
		**Injection:**	1 of 1
**Acq. method:**	Regular method.amx	**Injection volume:**	5.000 µL
**Analysis method:**	MS method-purity.pmx	**Acq. operator:**	SYSTEM

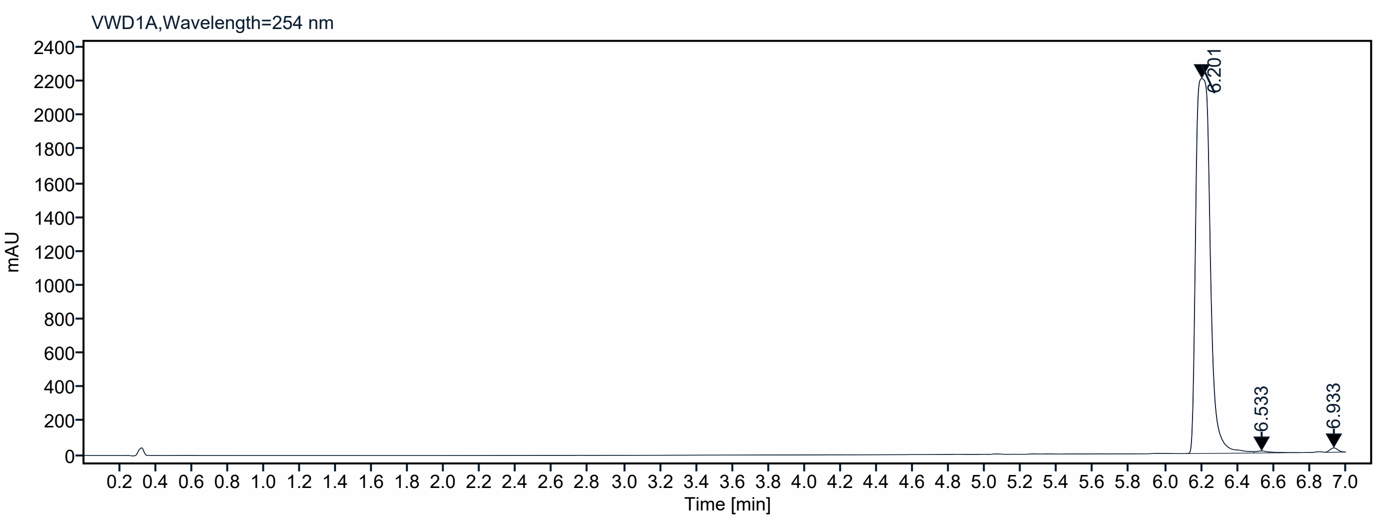

Signal: MS1 +TIC SCAN ESI Frag=110V Gain=1.0

RT [min]	Width [min]	Area	Height	Area%
6.284	0.1901	1885253.1736	308220.8720	100.0000
	**Sum**	**1885253.1736**		

Signal: VWD1A,Wavelength=254 nm

RT [min]	Width [min]	Area	Height	Area%
6.201	0.3788	12236.2438	2207.8991	98.8406
6.533	0.2548	66.6667	11.6277	0.5385
6.933	0.1166	76.8663	25.2686	0.6209
	**Sum**	**12379.7769**		

Single Injection Report

6.284 - 6.289 (2025-06-23 06-49-08-05-00-14.dx)

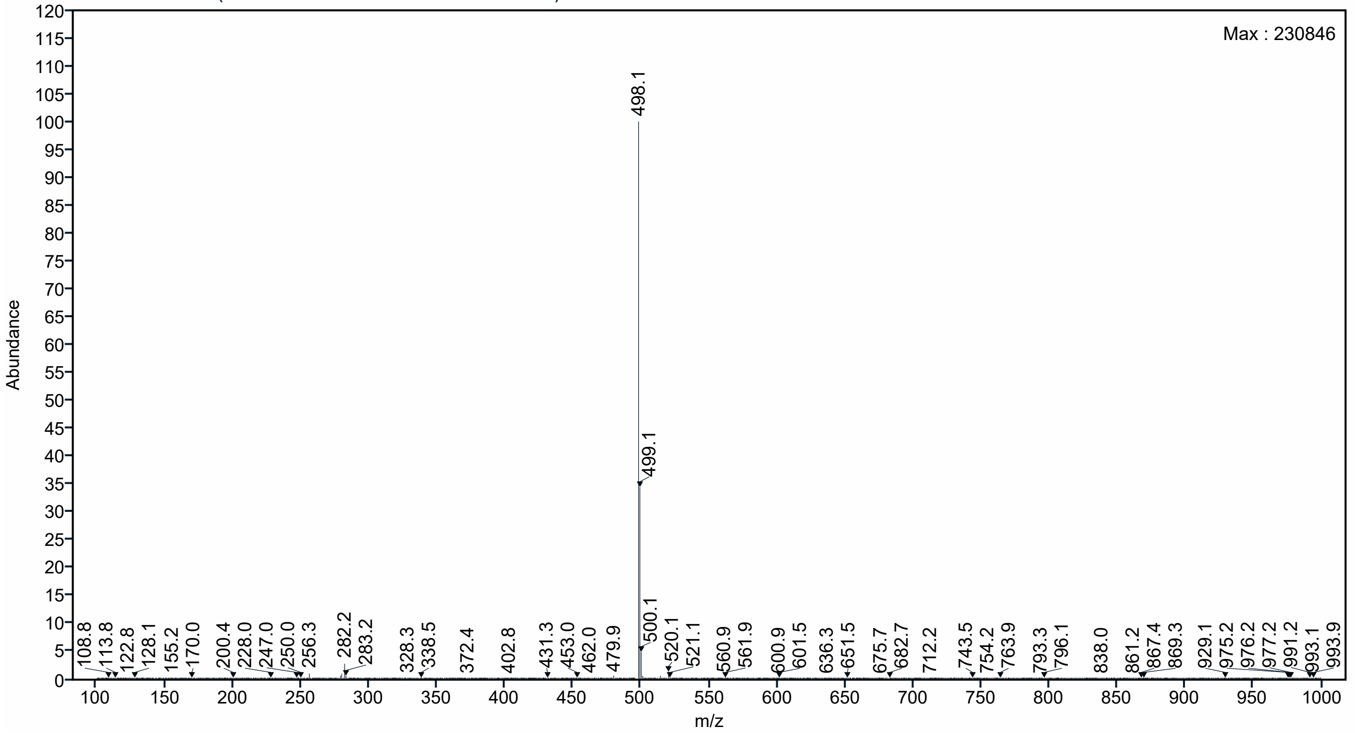

Supplement: Supplementary file 1 [file jm6c00011_si_001.pdf]
